# Estimated Number of Injection-Involved Overdose Deaths in US States From 2000 to 2020: Secondary Analysis of Surveillance Data

**DOI:** 10.2196/49527

**Published:** 2024-04-05

**Authors:** Eric William Hall, Patrick Sean Sullivan, Heather Bradley

**Affiliations:** 1 OHSU-PSU School of Public Health Oregon Health and Science University Portland, OR United States; 2 Department of Epidemiology Rollins School of Public Health Emory University Atlanta, GA United States

**Keywords:** death rate, death, drug abuse, drugs, injection drug use, injection, mortality, National Vital Statistics System, overdose death rate, overdose, state, substance abuse, Treatment Episode Dataset-Admission, treatment

## Abstract

**Background:**

In the United States, both drug overdose mortality and injection-involved drug overdose mortality have increased nationally over the past 25 years. Despite documented geographic differences in overdose mortality and substances implicated in overdose mortality trends, injection-involved overdose mortality has not been summarized at a subnational level.

**Objective:**

We aimed to estimate the annual number of injection-involved overdose deaths in each US state from 2000 to 2020.

**Methods:**

We conducted a stratified analysis that used data from drug treatment admissions (Treatment Episodes Data Set–Admissions; TEDS-A) and the National Vital Statistics System (NVSS) to estimate state-specific percentages of reported drug overdose deaths that were injection-involved from 2000 to 2020. TEDS-A collects data on the route of administration and the type of substance used upon treatment admission. We used these data to calculate the percentage of reported injections for each drug type by demographic group (race or ethnicity, sex, and age group), year, and state. Additionally, using NVSS mortality data, the annual number of overdose deaths involving selected drug types was identified by the following specific multiple-cause-of-death codes: heroin or synthetic opioids other than methadone (T40.1, T40.4), natural or semisynthetic opioids and methadone (T40.2, T40.3), cocaine (T40.5), psychostimulants with abuse potential (T43.6), sedatives (T42.3, T42.4), and others (T36-T59.0). We used the probabilities of injection with the annual number of overdose deaths, by year, primary substance, and demographic groups to estimate the number of overdose deaths that were injection-involved.

**Results:**

In 2020, there were 91,071 overdose deaths among adults recorded in the United States, and 93.1% (84,753/91,071) occurred in the 46 jurisdictions that reported data to TEDS-A. Slightly less than half (38,253/84,753, 45.1%; 95% CI 41.1%-49.8%) of those overdose deaths were estimated to be injection-involved, translating to 38,253 (95% CI 34,839-42,181) injection-involved overdose deaths in 2020. There was large variation among states in the estimated injection-involved overdose death rate (median 14.72, range 5.45-31.77 per 100,000 people). The national injection-involved overdose death rate increased by 323% (95% CI 255%-391%) from 2010 (3.78, 95% CI 3.33-4.31) to 2020 (15.97, 95% CI 14.55-17.61). States in which the estimated injection-involved overdose death rate increased faster than the national average were disproportionately concentrated in the Northeast region.

**Conclusions:**

Although overdose mortality and injection-involved overdose mortality have increased dramatically across the country, these trends have been more pronounced in some regions. A better understanding of state-level trends in injection-involved mortality can inform the prioritization of public health strategies that aim to reduce overdose mortality and prevent downstream consequences of injection drug use.

## Introduction

Drug overdose mortality has increased dramatically in the United States since 1999, with over 106,000 overdose deaths occurring in 2021 alone [1,2]. Although this trend is often largely attributed to an increase in opioid-involved overdose deaths [3,4], data from toxicology reports indicate that the specific drugs and drug classes that contribute to the burden of overdose mortality have changed over time [5-8]. In addition, primary routes of drug administration have evolved, and recent work suggests much of the recent increase in overdose mortality is likely attributable to increasing injection-involved overdose deaths. From 2007 to 2018, the estimated proportion of all overdose deaths that were injection-involved more than doubled from 18.5% to 42.2% [9]. Increases in injection-involved mortality may indicate an increase in injection behavior, which highlights additional public health concerns. Compared to other routes of substance use (eg, swallowing pills or smoking), injection drug use has the highest risk of overdose and the acquisition of bloodborne infections such as HIV, viral hepatitis, and skin infections [9-13].

National trends in injection-involved overdose mortality likely mask important geographic differences at subnational levels. Previous analysis of mortality data has identified regional differences in the substances implicated in overdose mortality trends [14-16]. Additionally, analyses of infectious disease surveillance data reveal geographic differences in both the rates of acute hepatitis B virus (HBV) and hepatitis C virus (HCV) infections [17] and the trends in HCV-related death rates among persons aged 40 years or younger [18], which are all likely related to the prevalence of injection drug use [19,20]. An understanding of subnational trends in injection-involved overdose mortality has implications both for local resource allocation and the types of interventions needed to prevent overdose mortality, as well as for public health strategies to address additional adverse health outcomes related to injection behavior. For example, the availability of naloxone [21-23] and the implementation of supervised injection sites [24] have both been shown to prevent overdoses, specifically among people who inject drugs. Syringe services programs can prevent infections through the provision of sterile needles and other injection equipment [25-27]. In this study, we aimed to estimate the annual number of injection-involved overdose deaths and the injection death rate within each US state from 2000 to 2020.

## Methods

### Overview

Our approach used and extended a previously published method for estimating the burden of injection-involved mortality [9]. Briefly, we conducted a stratified analysis that used data from drug treatment admissions to estimate the percentage of persons injecting by reported drug type within demographic strata. Using this estimated percentage along with counts of overdose deaths from the National Vital Statistics System (NVSS), we estimated the number of persons that died from an injection-involved drug overdose death within each state and year.

### Data Sources

Drug treatment admissions data were from the Treatment Episode Data Set–Admissions (TEDS-A; from 2000 to 2020) [28]. TEDS-A is a national data set that includes client-level data routinely collected from all publicly funded, and some privately funded, facilities that are licensed to provide substance abuse treatment in the United States. The national data set compiles data from individual state systems into a standardized format. Each record in TEDS-A represents an admission to a treatment program and includes demographic information, along with data on the substances used and the usual route of administration for each substance.

Data on the annual number of overdose deaths were from the NVSS detailed multiple cause of death mortality microdata files, obtained through a data request from the National Center for Health Statistics (NCHS) [29]. The NVSS microdata include a line-listing of all deaths that occurred in the United States from 2000 to 2020. Mortality data include the *International Classification of Diseases, Tenth Revision* (*ICD-10*) codes for multiple underlying causes of deaths. To align with previous definitions, overdose deaths were classified using the following *ICD-10* codes: X40-X44, X60-X64, X85, and Y10-Y14 [1,30]. Additionally, overdose deaths involving selected drug types were identified by the following specific multiple-cause-of-death codes: heroin or synthetic opioids other than methadone (T40.1, T40.4), natural or semisynthetic opioids and methadone (T40.2, T40.3), cocaine (T40.5), psychostimulants with abuse potential (T43.6), sedatives (T42.3, T42.4), and others (T36-T59.0). We used National Center for Health Statistics bridged-race annual population estimates [31] for denominators in all population death rates.

### Analysis

All analyses were limited to adults aged 18 years or older within 50 US states and the District of Columbia. Using TEDS-A data from 2000 to 2020, we identified all treatment admissions that included the following drugs: heroin or synthetic opioids (excluding methadone), stimulants (including methamphetamine, other amphetamines, and other stimulants), natural or semisynthetic opioids and methadone, cocaine (including crack), and sedatives (including benzodiazepines, other tranquilizers, barbiturates, and other sedatives). For each year and within each state, TEDS-A treatment admissions data were categorized into 16 strata defined by all combinations of sex (male and female), age group (18-39 years and ≥40 years), and race or ethnicity (Hispanic, non-Hispanic Black, non-Hispanic White, and non-Hispanic other). For each of the 5 drug types, within the analytic strata, we estimated the percentage of injection by calculating the percentage of admissions with data on the route of administration that reported injection as the usual route of administration. The number of treatment admissions and percentage of reported injections for each state, year, and drug type are reported in Table S1 in Multimedia Appendix 1.

Overdose deaths from NVSS that included toxicology data were categorized into the same 5 mutually exclusive drug categories. Deaths that indicated multiple drugs were categorized based on the drug that had the highest overall percentage of persons reporting injection, as estimated from the TEDS-A treatment data [9]. This approach was used because we were interested in the percentage of any drug having been injected rather than in classifying deaths as attributable to a single drug type. Overdose deaths that did not have a specific T-code listed (ie, only listed T50.9) were distributed to the 6 categories (the 5 defined drug use categories described above and any other T-codes) based on the nonmissing distribution within each year and demographic strata [32,33]. The percentage of all overdose deaths that were categorized into each drug category for each state and year is reported in Table S2 in Multimedia Appendix 2 [1,9,29,30,32,33].

To estimate the number of injection-involved overdose deaths, we multiplied the drug-specific probabilities of injection by the respective number of reported overdose deaths within each drug type, state, year, and demographic strata. We then collapsed the results to report the estimated number of injection-involved overdose deaths for each state and year. To estimate population-level rates, we summed the bridged-race population estimates as denominators. The CIs were estimated by calculating the Clopper-Pearson confidence limits for all estimates of drug-specific probabilities of injection. If the annual number of treatment admissions that reported any of the 5 drug types was less than 50 within any state, we suppressed the results for that state and year. Additionally, if more than 15% of treatment admissions that reported a drug of interest were missing, we suppressed the results for that state and year.

To demonstrate how changes in injection-involved overdose mortality differed by state and over time, we present state-level results from 3 years: 2000, 2010, and 2020. We also calculated the relative percentage change in the national injection-involved overdose death rate from 2010 to 2020 with CIs for multiyear percentage change [34]. If a state had a suppressed rate for either 2010 or 2020, we suppressed the estimated trend. We then compared the state-level trends to the national trend during the same time period to identify states that had more or less pronounced trends.

### Ethical Considerations

This study did not receive a review or a formal determination from an institutional review board because this analysis is not human-participant research, according to Regulation 45 CFR 46 [35].

## Results

In 2020, 90% (46/51) of jurisdictions reported treatment data and were included in the national TEDS-A data set (states not included were Idaho, Maryland, New Mexico, Oregon, and Washington). Overall, there were 91,071 overdose deaths among adults recorded in the United States in 2020. Of those, 93.1% (84,753/91,071) occurred in the 46 jurisdictions that reported data to TEDS-A. Slightly less than half (38,253/84,753, 45.1%, 95% CI 41.1%-49.8%) of those were estimated to be injection-involved, translating to 38,253 (95% CI 34,839-42,181) injection-involved overdose deaths (Table 1). In 2020, the national injection overdose death rate was 15.97 (95% CI 14.55-17.61) per 100,000 people.

**Table 1:** Estimated number of injection-involved overdose deaths among adults by US state, 2020. State-level results were suppressed if the annual number of treatment admissions that reported any of the 5 drug types was <50 or if ≥15% of treatment admissions that reported a drug of interest were missing data on route of administration.

State	Population, n	Overdose deaths, n	Injection-involved overdose deaths		
			Deaths, n (95% CI)	% of overdose deaths (95% CI)	Rate per 100,000 (95% CI)
Alabama	3,834,249	1023	456 (407-513)	44.6 (39.7-50.1)	11.89 (10.61-13.37)
Alaska	552,427	158	83 (68-100)	52.6 (42.8-63.3)	15.06 (12.25-18.11)
Arizona	5,774,978	2483	779 (539-1249)	31.4 (21.7-50.3)	13.49 (9.33-21.63)
Arkansas	2,330,808	540	248 (192-299)	46 (35.5-55.4)	10.66 (8.23-12.85)
California	30,576,844	8778	3475 (3343-3620)	39.6 (38.1-41.2)	11.37 (10.93-11.84)
Colorado	4,557,684	1461	553 (513-599)	37.9 (35.1-41)	12.14 (11.26-13.14)
Connecticut	2,838,054	1364	543 (510-578)	39.8 (37.4-42.4)	19.13 (17.99-20.36)
Delaware	782,153	444	175 (159-192)	39.3 (35.8-43.3)	22.33 (20.31-24.59)
District of Columbia	583,228	421	Suppressed	Suppressed	Suppressed
Florida	17,482,580	7202	3767 (3475-4073)	52.3 (48.3-56.6)	21.54 (19.88-23.30)
Georgia	8,210,067	1902	773 (683-868)	40.7 (35.9-45.7)	9.42 (8.32-10.58)
Hawaii	1,111,188	273	Suppressed	Suppressed	Suppressed
Idaho	1,375,870	284	—^a^	—	—
Illinois	9,809,562	3532	1063 (971-1169)	30.1 (27.5-33.1)	10.84 (9.90-11.92)
Indiana	5,188,514	2305	1250 (1161-1344)	54.2 (50.4-58.3)	24.10 (22.38-25.90)
Iowa	2,438,002	428	171 (150-194)	40 (35-45.3)	7.01 (6.14-7.95)
Kansas	2,217,059	474	222 (178-265)	46.8 (37.5-55.8)	10.01 (8.03-11.94)
Kentucky	3,475,334	2075	1104 (1025-1185)	53.2 (49.4-57.1)	31.77 (29.50-34.09)
Louisiana	3,564,038	1889	847 (718-986)	44.8 (38-52.2)	23.76 (20.16-27.67)
Maine	1,101,973	495	229 (202-257)	46.3 (40.8-52)	20.80 (18.34-23.34)
Maryland	4,721,883	2757	—	—	—
Massachusetts	5,552,051	2298	1375 (1335-1416)	59.8 (58.1-61.6)	24.77 (24.05-25.51)
Michigan	7,839,742	2742	1295 (1237-1355)	47.2 (45.1-49.4)	16.52 (15.78-17.29)
Minnesota	4,356,123	1035	413 (379-450)	39.9 (36.6-43.5)	9.47 (8.69-10.33)
Mississippi	2,273,653	582	256 (202-318)	44.1 (34.7-54.6)	11.28 (8.89-13.99)
Missouri	4,780,119	1859	921 (856-987)	49.5 (46.1-53.1)	19.26 (17.92-20.64)
Montana	850,894	160	Suppressed	Suppressed	Suppressed
Nebraska	1,462,537	209	94 (69-115)	45 (33.2-55.2)	6.44 (4.74-7.88)
Nevada	2,440,679	817	292 (185-440)	35.7 (22.6-53.8)	11.95 (7.58-18.01)
New Hampshire	1,113,141	391	Suppressed	Suppressed	Suppressed
New Jersey	6,947,836	2827	1175 (1130-1223)	41.6 (40-43.3)	16.91 (16.26-17.60)
New Mexico	1,633,828	778	—	—	—
New York	15,348,422	4950	2082 (2025-2142)	42.1 (40.9-43.3)	13.57 (13.20-13.95)
North Carolina	8,294,423	3129	1529 (1411-1654)	48.9 (45.1-52.9)	18.44 (17.01-19.94)
North Dakota	583,680	110	Suppressed	Suppressed	Suppressed
Ohio	9,124,576	5179	2688 (2485-2903)	51.9 (48-56)	29.46 (27.23-31.81)
Oklahoma	3,027,263	750	289 (245-336)	38.5 (32.7-44.7)	9.55 (8.09-11.09)
Oregon	3,380,729	794	—	—	—
Pennsylvania	10,162,497	5141	2877 (2684-3077)	56 (52.2-59.8)	28.31 (26.41-30.27)
Rhode Island	855,276	395	182 (162-203)	46 (41-51.4)	21.24 (18.95-23.73)
South Carolina	4,100,115	1730	487 (392-607)	28.2 (22.7-35.1)	11.89 (9.57-14.79)
South Dakota	674,238	81	37 (26-49)	45.4 (32-59.9)	5.45 (3.84-7.20)
Tennessee	5,373,433	3021	1587 (1446-1734)	52.5 (47.9-57.4)	29.53 (26.91-32.27)
Texas	21,925,627	4116	1677 (1548-1823)	40.7 (37.6-44.3)	7.65 (7.06-8.32)
Utah	2,320,603	619	217 (193-250)	35.1 (31.1-40.4)	9.36 (8.30-10.77)
Vermont	510,181	189	93 (82-104)	49.4 (43.6-55.2)	18.29 (16.14-20.44)
Virginia	6,724,143	2226	990 (888-1101)	44.5 (39.9-49.5)	14.72 (13.21-16.38)
Washington	6,027,818	1705	—	—	—
West Virginia	1,428,520	1328	Suppressed	Suppressed	Suppressed
Wisconsin	4,574,131	1523	771 (694-857)	50.6 (45.6-56.3)	16.86 (15.18-18.74)
Wyoming	449,237	99	Suppressed	Suppressed	Suppressed
National^b^	256,662,010	91,071	38,253 (34,839-42,181)	45.1 (41.1-49.8)	15.97 (14.55-17.61)

^a^Not available. These states did not report data to Treatment Episodes Data Set–Admissions.

^b^National rate does not include populations from states that did not report data to Treatment Episodes Data Set–Admissions (Idaho, Maryland, New Mexico, Oregon, and Washington).

**Figure 1:**
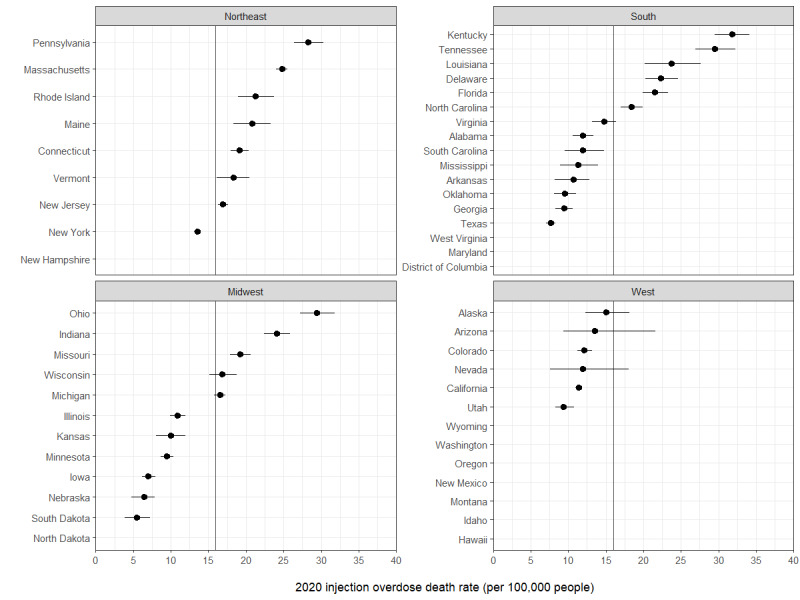
Estimated injection-involved overdose death rate among adults, by state and region, United States, 2020. The vertical line represents the national estimated injection overdose death rate for 2020. States without an estimate either did not report data to TEDS-A or were suppressed. State-level results were suppressed if the annual number of treatment admissions that reported any of the 5 drug types was <50 or if ≥15% of treatment admissions that reported a drug of interest were missing data on route of administration.

In addition to the 5 states that did not report TEDS-A data, we suppressed data from 7 states, resulting in 39 (77%) states with estimated results for 2020. In 2020, the estimated number of injection-involved overdose deaths by state ranged from 37 (95% CI 26-49) in South Dakota to 3767 (95% CI 3475-4073) in Florida (Table 2). There was large variation in the estimated injection-involved overdose death rate by state (median 14.72, range 5.45-31.77 per 100,000 people). Of the 10 states with the highest estimated injection overdose death rate, 5 were located in the South (Kentucky, Tennessee, Louisiana, Delaware, and Florida), 3 were in the Northeast (Pennsylvania, Massachusetts, and Rhode Island), and 2 (Ohio and Indiana) were in the Midwest Census regions. Of the 8 states in the Northeast that have results, 7 (86%) had an estimated injection overdose death rate higher than the national rate (Figure 1). All 6 states with results in the West had an estimated injection overdose death rate lower than the national rate. Over half (19682/38253, 51.5%) of the total number of estimated injection-involved overdoses occurred in 8 states (Florida, California, Pennsylvania, Ohio, New York, Texas, Tennessee, and North Carolina).

**Table 2:** Estimated number of injection-involved overdose deaths among adults, by US state and year, 2000-2020. State-level results were suppressed if the annual number of treatment admissions that reported any of the 5 drug types was <50 or if ≥15% of treatment admissions that reported a drug of interest were missing data on route of administration.

State	2000				2020		
	Overdose deaths, n	Injection-involved deaths		Overdose deaths, n		Injection-involved deaths	
		Deaths, n (95% CI)	% of overdose deaths (95% CI)			Deaths, n (95% CI)	% of overdose deaths (95% CI)
Alabama	192	31 (22-41)	16.1 (11.4-21.2)	1023		456 (407-513)	44.6 (39.7-50.1)
Alaska	47	Suppressed	Suppressed	158		83 (68-100)	52.6 (42.8-63.3)
Arizona	517	Suppressed	Suppressed	2483		779 (539-1249)	31.4 (21.7-50.3)
Arkansas	135	36 (30-42)	27 (22.6-31.1)	540		248 (192-299)	46 (35.5-55.4)
California	1921	565 (541-598)	29.4 (28.2-31.1)	8778		3475 (3343-3620)	39.6 (38.1-41.2)
Colorado	346	97 (85-112)	28 (24.7-32.5)	1461		553 (513-599)	37.9 (35.1-41)
Connecticut	317	—^a^	—	1364		543 (510-578)	39.8 (37.4-42.4)
Delaware	54	10 (8-16)	19.1 (14-28.8)	444		175 (159-192)	39.3 (35.8-43.3)
District of Columbia	76	Suppressed	Suppressed	421		Suppressed	Suppressed
Florida	1145	329 (305-356)	28.7 (26.7-31.1)	7202		3767 (3475-4073)	52.3 (48.3-56.6)
Georgia	355	25 (16-42)	7.2 (4.6-11.9)	1902		773 (683-868)	40.7 (35.9-45.7)
Hawaii	63	8 (5-17)	12.4 (7.6-27.5)	273		Suppressed	Suppressed
Idaho	63	7 (5-15)	11.3 (8.2-23.3)	284		—	—
Illinois	856	64 (51-83)	7.5 (5.9-9.7)	3532		1063 (971-1169)	30.1 (27.5-33.1)
Indiana	214	43 (38-48)	20.3 (17.9-22.6)	2305		1250 (1161-1344)	54.2 (50.4-58.3)
Iowa	71	11 (8-15)	16.2 (12-21.4)	428		171 (150-194)	40 (35-45.3)
Kansas	103	23 (17-27)	21.9 (16.7-26.7)	474		222 (178-265)	46.8 (37.5-55.8)
Kentucky	236	Suppressed	Suppressed	2075		1104 (1025-1185)	53.2 (49.4-57.1)
Louisiana	246	28 (21-37)	11.6 (8.6-15.2)	1889		847 (718-986)	44.8 (38-52.2)
Maine	59	13 (11-17)	22.5 (18.8-29.5)	495		229 (202-257)	46.3 (40.8-52)
Maryland	614	41 (37-47)	6.7 (6-7.6)	2757		—	—
Massachusetts	461	44 (41-48)	9.6 (8.9-10.5)	2298		1375 (1335-1416)	59.8 (58.1-61.6)
Michigan	554	164 (153-177)	29.6 (27.7-31.9)	2742		1295 (1237-1355)	47.2 (45.1-49.4)
Minnesota	128	17 (13-23)	13.5 (10.4-17.7)	1035		413 (379-450)	39.9 (36.6-43.5)
Mississippi	110	18 (12-25)	16.7 (10.7-22.6)	582		256 (202-318)	44.1 (34.7-54.6)
Missouri	299	88 (76-102)	29.4 (25.5-34.3)	1859		921 (856-987)	49.5 (46.1-53.1)
Montana	41	7 (4-11)	17.5 (10.6-26.2)	160		Suppressed	Suppressed
Nebraska	44	Suppressed	Suppressed	209		94 (69-115)	45 (33.2-55.2)
Nevada	273	74 (59-112)	26.9 (21.8-40.9)	817		292 (185-440)	35.7 (22.6-53.8)
New Hampshire	45	4 (3-9)	10 (6.6-19.2)	391		Suppressed	Suppressed
New Jersey	639	155 (145-169)	24.3 (22.7-26.5)	2827		1175 (1130-1223)	41.6 (40-43.3)
New Mexico	263	75 (55-112)	28.4 (21-42.6)	778		—	—
New York	762	55 (50-61)	7.2 (6.6-8)	4950		2082 (2025-2142)	42.1 (40.9-43.3)
North Carolina	485	135 (114-158)	27.9 (23.5-32.6)	3129		1529 (1411-1654)	48.9 (45.1-52.9)
North Dakota	14	Suppressed	Suppressed	110		Suppressed	Suppressed
Ohio	555	137 (123-157)	24.7 (22.1-28.3)	5179		2688 (2485-2903)	51.9 (48-56)
Oklahoma	233	63 (47-80)	27.1 (20.4-34.4)	750		289 (245-336)	38.5 (32.7-44.7)
Oregon	206	56 (51-62)	27.2 (25-30)	794		—	—
Pennsylvania	1134	306 (290-325)	27 (25.6-28.7)	5141		2877 (2684-3077)	56 (52.2-59.8)
Rhode Island	73	6 (5-9)	8.4 (6.8-12.5)	395		182 (162-203)	46 (41-51.4)
South Carolina	252	37 (26-52)	14.8 (10.5-20.7)	1730		487 (392-607)	28.2 (22.7-35.1)
South Dakota	19	Suppressed	Suppressed	81		37 (26-49)	45.4 (32-59.9)
Tennessee	388	48 (34-70)	12.5 (8.7-18)	3021		1587 (1446-1734)	52.5 (47.9-57.4)
Texas	1006	290 (263-325)	28.9 (26.1-32.3)	4116		1677 (1548-1823)	40.7 (37.6-44.3)
Utah	210	65 (58-77)	31.2 (27.7-36.6)	619		217 (193-250)	35.1 (31.1-40.4)
Vermont	36	9 (7-14)	24.2 (19.1-39.7)	189		93 (82-104)	49.4 (43.6-55.2)
Virginia	407	59 (44-81)	14.5 (10.9-19.9)	2226		990 (888-1101)	44.5 (39.9-49.5)
Washington	551	131 (118-149)	23.7 (21.5-27.1)	1705		—	—
West Virginia	112	—	—	1328		Suppressed	Suppressed
Wisconsin	244	47 (32-71)	19.3 (13.2-29.1)	1523		771 (694-857)	50.6 (45.6-56.3)
Wyoming	22	Suppressed	Suppressed	99		Suppressed	Suppressed

^a^Not available. These states did not report data to Treatment Episodes Data Set–Admissions.

**Figure 2:**
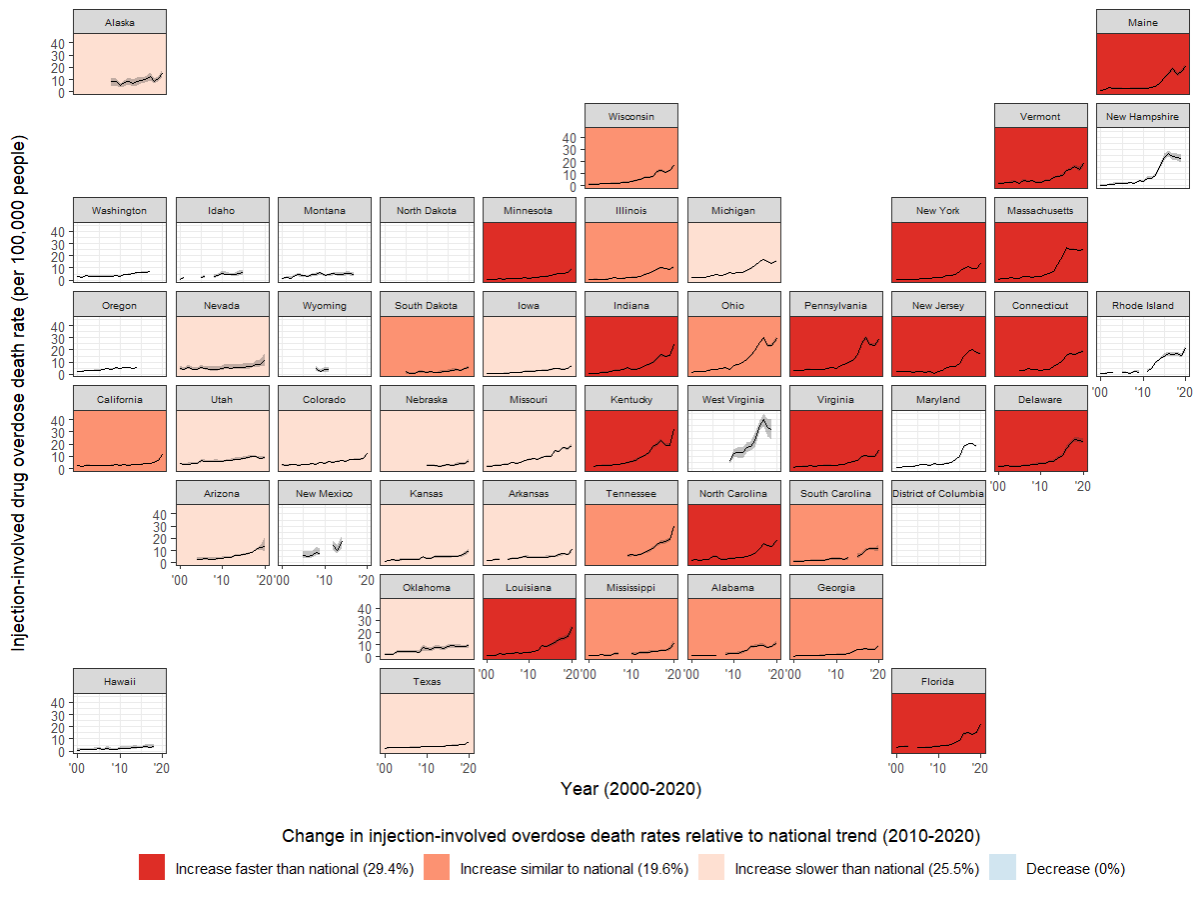
Estimated injection-involved overdose death rate among adults, by state and year, United States, from 2000 to 2020. The state-level results were suppressed if the annual number of treatment admissions that reported any of the 5 drug types was <50 or if ≥15% of treatment admissions that reported a drug of interest were missing data on route of administration.

The number of overdose deaths among adults increased more than 5-fold from 2000 (n=17,196) to 2020 (n=91,071; Table 2). Among jurisdictions that reported TEDS-A data, the estimated number of injection-involved overdose deaths increased more than 10-fold from 2000 (50/51 states reported TEDS-A data; n=3549, 95% CI 3103-4248) to 2020 (46/51 states reported TEDS-A data; n=38,253, 95% CI 34,839-42,181). Over the same time period, the national injection overdose death rate increased from 1.72 (95% CI 1.51-2.05) to 15.97 (95% CI 14.55-17.61). Much of that increase occurred from 2010 to 2020, in which the injection overdose death rate increased by 323% (95% CI 255%-391%). We estimated a state-level relative percentage change in the injection overdose death rate from 2010 to 2020 for 38 states. States that experienced the largest relative increase in injection overdose death rates were disproportionately concentrated in the Northeast (Figure 2). The injection overdose death rate increased more than 6-fold in Massachusetts (733% relative increase), Maine (678% relative increase), New York (610% relative increase), Virginia (600% relative increase), New Jersey (573% relative increase), Louisiana (545% relative increase), Florida (523% relative increase), and Connecticut (507% relative increase). There were 6 states (Utah, Texas, Arkansas, Missouri, Nebraska, and Iowa) that experienced a more gradual increase in the injection overdose death rate than the national increase. The estimated number of injection-involved overdose deaths and injection overdose death rate for each state and year are reported in Table S3 in Multimedia Appendix 3. Additionally, Figure S1 in Multimedia Appendix 4 visualizes the relationship between state-level overdose death rates and the percentage of overdose deaths that were estimated to be injection-involved for each year.

## Discussion

This work provides additional evidence that drug overdose mortality and other adverse outcomes related to injection drug use continue to be growing public health challenges across the United States. We previously demonstrated that much of the increase in overdose mortality since 2000 [1] has been attributable to injection-involved overdose deaths [9], and this analysis indicates that trend continued through 2020. We estimated that over half (34,704/67,986, 51%) of the increase in overdose deaths between 2000 and 2020 were injection-involved deaths. Importantly, these results provide additional granularity that reveals geographic differences in the magnitude of that trend. Although the injection-involved overdose death rate has increased dramatically in all states, the increase in injection overdose mortality is most pronounced in the Northeast and Appalachia regions, further emphasizing the need to prioritize harm reduction strategies in these areas. These geographic differences may be indicative of differences in the drug supply [36], an ongoing shift from prescription opioid mortality to injection-involved mortality [37], or geographic differences in access to overdose prevention interventions.

The estimated injection-involved overdose death rate for any jurisdiction is driven by both trends in overdose deaths (the total number and distribution of substances involved) and trends in reported injection behavior by substance type. Nationally, the likelihood of injecting heroin or synthetic opioids steadily increased before peaking in 2014 at 69.4% and declining to 58.3% in 2020 (Table S1 in Multimedia Appendix 1). However, during the same time period, the number of overdose deaths (46,764 in 2014 and 91,071 in 2020) and the proportion of overdose deaths attributable to heroin or synthetic opioids (39.6% in 2014 and 70.4% in 2020; Table S2 in Multimedia Appendix 2) continually increased, resulting in an increase in the overall injection-involved overdose death rate. As seen in Figure 2, some states have experienced a slight decrease in the estimated injection-involved overdose death rate in recent years due to slightly different temporal trends. For example, in Massachusetts, the injection-involved overdose death rate peaked in 2016. Similar to the national trend, the likelihood of injecting heroin or synthetic opioids has declined in recent years (79.6% in 2016 and 68.8% in 2020). This decrease outweighed the corresponding gradual increase in the percent of overdose deaths attributable to heroin or synthetic opioids (80.0% in 2016 and 85.8% in 2020), resulting in a decrease in the injection-involved overdose death rate. Similarly, recent trends in stimulant use and, subsequently, deaths attributed to stimulant use also influence these estimates. Generally, areas with a higher proportion of deaths attributable to stimulant use are estimated to have relatively fewer injection-involved overdose deaths, based on a lower probability of injecting stimulants compared to heroin or synthetic opioids. In Tables S1 and S2 in Multimedia Appendices 1 and 2, we present both the probability of injecting by substance type and the percentage of deaths attributable to each substance as components of trends in injection-involved overdose in each state. Disaggregating the drivers of state-level trends in injection-involved overdose death rates can help inform the composition of harm reduction strategies.

Understanding changes in injection-involved overdose mortality provides additional value for designing overdose prevention and intervention strategies that cannot be gained from overall trends in overdose mortality alone. For example, states that are experiencing a particularly sharp increase in injection-involved overdose mortality may prioritize strategies that focus on injection harm reduction, such as naloxone access or supervised injection sites. Although the Centers for Disease Control and Prevention (CDC) has started to implement efforts to expand overdose surveillance and collect better data on the evidence of injection through the development of the State Unintentional Drug Overdose Report System (SUDORS) [38], the system does not yet have complete data on the route of administration and is too new to investigate temporal trends in injection-drug use.

Beyond the implications for overdose mortality prevention, these results can inform efforts to reduce infectious disease transmission due to injection drug use. From a public health surveillance perspective, injection drug use behavior, which is associated with an increased risk of transmission of several viruses and bacteria, is extremely difficult to measure and monitor [39]. These results can help state health departments and researchers better understand the burden of injection drug use in their jurisdiction. Recently, our team developed an approach that built upon this methodology to estimate that there were 3.7 million “people who injected drugs” in the United States in 2018 [40]. Following that framework, researchers could use these results, along with their own local data, to estimate the population size of people who injected drugs at the state level. There has been an increase in federal and state-led efforts to eliminate HCV [41] and HIV [42] infections, both of which disproportionately occur among people who injected drugs in the United States. The results from this analysis can be interpreted alongside data on the prevalence of HCV [17,43] or HIV [44] to highlight areas in most need of infectious disease prevention and treatment efforts. The comparison across states can inform the allocation of federal funding to scale up infectious disease prevention interventions [25,26,45] (eg, syringe service programs, substance use treatment centers, and hepatitis B vaccination clinics) or screening and treatment resources (eg, testing for acute viral hepatitis or HIV infections and providing linkage to care) [46,47].

As previously described in the development of this method [9], we note that we assumed the likelihood of injection within demographic strata and drug type is the same among living persons that enter treatment and persons who died of a drug overdose. This is a conservative approach because if the injection of any of these substances is more lethal than other routes of administration, these results would be an underestimate of the true burden of injection-involved mortality. Additionally, the selection of TEDS-A data to estimate the likelihood of injection comes with strengths and limitations. TEDS-A is the most comprehensive and complete single data source on substance abuse treatment admissions across the country. TEDS-A data collection has been an ongoing systematic activity for more than 30 years, which enables us to look at changes in reported behaviors over time. However, there may be differences in the data collection systems or the use of public funding for substance use treatment within each state [48]. As a result, the degree to which TEDS-A captures all treatment admissions may differ between states. However, this methodology does not depend on the complete enumeration of substance use treatment admissions but rather on the probability of reporting injection among those that are captured by TEDS-A data systems. In 2020, 99.2% of all admissions for a drug of interest reported data on the route of administration. The state-level response rate ranged from 44.6% in Wyoming to >99% in many states. After Wyoming, the second-lowest response rate was 95.2% in North Dakota.

Additionally, the analytic decision to categorize overdose deaths with multiple substances recorded for the drug with the highest likelihood of injection could result in an overestimate if polydrug use is less likely to involve injection than just using one of the drugs independently. In general, this assumption becomes more important, and the use of these estimates may be more limited in time periods or areas in which there is a high proportion of polysubstance use. A future analysis could validate these estimates with an analysis of reported injections from SUDORS [38] as those data become available. Finally, we assumed overdose deaths without T-codes were missing at random within strata and followed the same distribution as overdose deaths with recorded T-codes. However, the potential for this assumption to bias results has been reduced in recent years, as the completeness of these data has increased substantially over time [49].

Measuring trends in injection behavior and understanding how those trends impact the ongoing overdose mortality epidemic remains a huge public health challenge. These estimates provide longitudinal data points that reveal additional understanding of geographic heterogeneity in recent trends of injection-involved overdose mortality. As state and local public health departments continue to implement programs aimed at reducing overdose mortality and preventing infectious disease transmission, innovative and timely data sources can help inform the development of strategies best suited for their particular setting.

## Appendix

Multimedia Appendix 1 Total number of treatment admissions among adults that reported injection as a route of administration, by drug type, state and year, United States, 2000-2020.

Multimedia Appendix 2 Percent of overdose deaths among adults attributed to each drug type, by state and year, United States, 2000-2020.

Multimedia Appendix 3 Estimated number of injection-involved overdose deaths among adults, by state and year, United States, 2000-2020.

Multimedia Appendix 4 Overdose death rate and the estimated percent of overdose deaths that were injection-involved among adults, by state and year, United States, 2000-2020. Note: Points represent the point estimate for the estimated percent of overdose deaths that were injection-involved.

## Abbreviations

CDC: Centers for Disease Control and Prevention
HBV: hepatitis B virus
HCV: hepatitis C virus
ICD-10: *International Classification of Diseases, Tenth Revision*
NCHS: National Center for Health Statistics
NVSS: National Vital Statistics System
SUDORS: State Unintentional Drug Overdose Report System
TEDS-A: Treatment Episodes Data Set–Admissions

## Data Availability

The data sets analyzed during this study are available in the National Center for Health Statistics (NCHS) Restricted-Use Vital Statistics Data repository [29] or the Treatment Episodes Data Set–Admissions (TEDS-A) web-based repository [50]. All data generated during this study are included in this published article and its supplementary information files.
