# Supplementary material for: Estimated Number of Injection-Involved Overdose Deaths in US States From 2000 to 2020: Secondary Analysis of Surveillance Data
Source: JMIR Public Health Surveill. 2024 Apr 5;10:e49527. doi: 10.2196/49527 (PMC11031697; doi:10.2196/49527)
Supplement: Multimedia Appendix 1 [file publichealth_v10i1e49527_app1.docx]

|  |  | Heroin/Synthetic opioids | |  | Cocaine | |  | Stimulants | |  | Natural/semi-synthetic opioids/methadone | |  | Sedatives | |
| --- | --- | --- | --- | --- | --- | --- | --- | --- | --- | --- | --- | --- | --- | --- | --- |
| **State** | **Year** | **n** | **% injection** |  | **n** | **% injection** |  | **n** | **% injection** |  | **n** | **% injection** |  | **n** | **% injection** |
| Alabama | 2000 | 288 | 54.9 |  | 5,605 | 2.3 |  | 848 | 19.6 |  | 1,217 | 22.6 |  | 644 | 0.8 |
| Alabama | 2001 | 315 | 50.5 |  | 5,578 | 3.3 |  | 1,342 | 17.7 |  | 1,495 | 22.8 |  | 718 | 1.3 |
| Alabama | 2002 | 274 | 48.9 |  | 6,405 | 3.9 |  | 1,866 | 23.2 |  | 1,905 | 21.1 |  | 848 | 1.1 |
| Alabama | 2003 | 274 | 58.0 |  | 7,052 | 4.6 |  | 2,335 | 25.1 |  | 2,132 | 20.7 |  | 1,012 | 1.3 |
| Alabama | 2004 | 297 | 64.0 |  | 6,807 | 4.1 |  | 2,682 | 23.1 |  | 2,238 | 18.3 |  | 1,027 | 1.4 |
| Alabama | 2005 | 248 | 60.9 |  | 6,420 | 3.3 |  | 2,540 | 18.3 |  | 2,416 | 15.4 |  | 902 | 2.0 |
| Alabama | 2006 | 88 | 27.3 |  | 5,673 | 4.9 |  | 1,977 | 7.2 |  | 1,947 | 7.8 |  | 547 | 4.4 |
| Alabama | 2007 | Data not reported | |  |  |  |  |  |  |  |  |  |  |  |  |
| Alabama | 2008 | 148 | 56.8 |  | 4,009 | 2.6 |  | 1,385 | 18.2 |  | 2,099 | 13.4 |  | 838 | 1.3 |
| Alabama | 2009 | 265 | 52.5 |  | 4,565 | 5.3 |  | 2,121 | 21.9 |  | 2,677 | 14.5 |  | 949 | 1.5 |
| Alabama | 2010 | 258 | 74.4 |  | 4,642 | 3.1 |  | 2,687 | 24.3 |  | 3,647 | 16.2 |  | 1,145 | 1.0 |
| Alabama | 2011 | 249 | 66.7 |  | 1,621 | 3.9 |  | 1,292 | 31.4 |  | 1,482 | 17.7 |  | 558 | 0.7 |
| Alabama | 2012 | 237 | 78.9 |  | 1,785 | 5.1 |  | 1,314 | 32.8 |  | 1,874 | 20.9 |  | 627 | 0.6 |
| Alabama | 2013 | 375 | 78.1 |  | 1,669 | 4.3 |  | 1,559 | 38.6 |  | 2,084 | 22.3 |  | 740 | 0.9 |
| Alabama | 2014 | 534 | 77.3 |  | 1,401 | 4.6 |  | 1,505 | 37.2 |  | 1,678 | 18.7 |  | 643 | 0.9 |
| Alabama | 2015 | 836 | 80.3 |  | 1,342 | 6.4 |  | 1,864 | 42.5 |  | 2,092 | 20.7 |  | 689 | 0.9 |
| Alabama | 2016 | 1,150 | 79.5 |  | 1,700 | 5.1 |  | 2,898 | 40.3 |  | 2,193 | 18.2 |  | 848 | 0.7 |
| Alabama | 2017 | 1,431 | 79.7 |  | 1,667 | 5.1 |  | 3,653 | 37.2 |  | 2,502 | 14.8 |  | 875 | 0.7 |
| Alabama | 2018 | 3,159 | 75.9 |  | 1,928 | 7.1 |  | 4,692 | 37.0 |  | 5,062 | 13.2 |  | 1,081 | 1.2 |
| Alabama | 2019 | 3,151 | 72.1 |  | 1,687 | 4.9 |  | 5,115 | 36.1 |  | 4,357 | 11.9 |  | 1,196 | 0.5 |
| Alabama | 2020 | 2,715 | 70.1 |  | 1,350 | 4.2 |  | 4,471 | 33.5 |  | 3,372 | 11.2 |  | 817 | 0.5 |
| Alaska | 2000 | 63 | 85.7 |  | 887 | 9.4 |  | 137 | 23.4 |  | 89 | 14.6 |  | 44 | 2.3 |
| Alaska | 2001 | 27 | 77.8 |  | 652 | 7.8 |  | 123 | 17.1 |  | 104 | 17.3 |  | 34 | 2.9 |
| Alaska | 2002 | 49 | 69.4 |  | 713 | 8.6 |  | 219 | 18.3 |  | 183 | 24.0 |  | 55 | 7.3 |
| Alaska | 2003 | 36 | 80.6 |  | 530 | 8.9 |  | 170 | 17.1 |  | 164 | 20.1 |  | 42 | 4.8 |
| Alaska | 2004 | Data not reported | |  |  |  |  |  |  |  |  |  |  |  |  |
| Alaska | 2005 | 20 | 95.0 |  | 191 | 9.4 |  | 51 | 17.6 |  | 100 | 40.0 |  | 13 | 7.7 |
| Alaska | 2006 | 31 | 87.1 |  | 250 | 10.8 |  | 124 | 18.5 |  | 136 | 23.5 |  | 26 | 0.0 |
| Alaska | 2007 | 100 | 70.0 |  | 518 | 10.6 |  | 226 | 17.3 |  | 221 | 26.7 |  | 42 | 2.4 |
| Alaska | 2008 | 172 | 80.2 |  | 709 | 10.2 |  | 269 | 29.0 |  | 343 | 25.7 |  | 79 | 7.6 |
| Alaska | 2009 | 292 | 84.6 |  | 613 | 12.9 |  | 332 | 24.4 |  | 519 | 23.7 |  | 80 | 10.0 |
| Alaska | 2010 | 323 | 82.7 |  | 672 | 11.5 |  | 446 | 32.1 |  | 636 | 24.1 |  | 95 | 4.2 |
| Alaska | 2011 | 342 | 82.7 |  | 639 | 11.6 |  | 557 | 32.9 |  | 678 | 27.0 |  | 114 | 7.0 |
| Alaska | 2012 | 415 | 74.5 |  | 501 | 10.2 |  | 688 | 34.7 |  | 702 | 28.8 |  | 97 | 5.2 |
| Alaska | 2013 | 572 | 72.4 |  | 390 | 11.8 |  | 783 | 35.6 |  | 518 | 23.4 |  | 97 | 3.1 |
| Alaska | 2014 | 717 | 66.2 |  | 364 | 8.5 |  | 918 | 35.3 |  | 545 | 23.9 |  | 91 | 4.4 |
| Alaska | 2015 | 922 | 71.3 |  | 399 | 8.5 |  | 1,240 | 37.1 |  | 579 | 20.2 |  | 160 | 2.5 |
| Alaska | 2016 | 1,264 | 68.9 |  | 353 | 9.1 |  | 1,600 | 36.5 |  | 639 | 22.1 |  | 144 | 4.9 |
| Alaska | 2017 | 1,395 | 71.8 |  | 375 | 11.2 |  | 1,892 | 44.0 |  | 520 | 19.4 |  | 107 | 6.5 |
| Alaska | 2018 | 1,786 | 74.0 |  | 382 | 9.7 |  | 2,183 | 42.4 |  | 551 | 20.9 |  | 134 | 6.0 |
| Alaska | 2019 | 1,656 | 69.3 |  | 305 | 9.2 |  | 2,200 | 42.7 |  | 422 | 19.2 |  | 122 | 6.6 |
| Alaska | 2020 | 1,361 | 68.3 |  | 235 | 9.4 |  | 1,637 | 40.3 |  | 335 | 18.5 |  | 75 | 6.7 |
| Arizona | 2000 | 378 | 81.0 |  | 550 | 6.4 |  | 709 | 21.7 |  | 48 | 6.3 |  | 6 | 0.0 |
| Arizona | 2001 | 1,045 | 87.5 |  | 2,043 | 10.9 |  | 2,330 | 17.8 |  | 257 | 4.7 |  | 119 | 1.7 |
| Arizona | 2002 | 435 | 81.1 |  | 1,421 | 9.1 |  | 1,569 | 20.1 |  | 175 | 11.4 |  | 87 | 0.0 |
| Arizona | 2003 | 613 | 85.2 |  | 1,223 | 10.3 |  | 1,908 | 14.4 |  | 75 | 10.7 |  | 71 | 0.0 |
| Arizona | 2004 | 734 | 77.7 |  | 1,791 | 6.6 |  | 4,168 | 12.2 |  | 165 | 6.7 |  | 182 | 0.5 |
| Arizona | 2005 | 1,425 | 76.2 |  | 2,614 | 7.1 |  | 5,653 | 10.7 |  | 235 | 6.4 |  | 176 | 0.0 |
| Arizona | 2006 | 1,196 | 75.6 |  | 2,581 | 6.5 |  | 4,692 | 12.3 |  | 367 | 4.9 |  | 210 | 0.0 |
| Arizona | 2007 | 1,340 | 71.9 |  | 2,673 | 7.2 |  | 3,957 | 11.5 |  | 527 | 6.6 |  | 248 | 0.0 |
| Arizona | 2008 | 1,826 | 69.2 |  | 2,994 | 6.3 |  | 4,057 | 13.2 |  | 768 | 5.1 |  | 286 | 1.0 |
| Arizona | 2009 | 2,065 | 66.1 |  | 1,946 | 6.9 |  | 3,523 | 14.7 |  | 1,103 | 5.5 |  | 362 | 0.3 |
| Arizona | 2010 | 3,527 | 65.0 |  | 3,087 | 7.0 |  | 5,772 | 13.4 |  | 2,106 | 6.3 |  | 716 | 0.6 |
| Arizona | 2011 | 3,197 | 66.8 |  | 3,168 | 5.7 |  | 6,096 | 14.0 |  | 2,331 | 7.1 |  | 623 | 0.8 |
| Arizona | 2012 | 3,610 | 67.9 |  | 2,927 | 6.3 |  | 7,085 | 14.7 |  | 2,717 | 7.4 |  | 710 | 1.1 |
| Arizona | 2013 | 4,408 | 68.0 |  | 2,395 | 6.0 |  | 7,224 | 19.9 |  | 2,589 | 7.6 |  | 589 | 0.8 |
| Arizona | 2014 | 4,971 | 67.5 |  | 2,220 | 6.9 |  | 8,037 | 18.9 |  | 2,647 | 7.2 |  | 552 | 1.8 |
| Arizona | 2015 | 4,886 | 64.2 |  | 2,147 | 6.4 |  | 7,964 | 19.1 |  | 2,293 | 8.3 |  | 468 | 0.6 |
| Arizona | 2016 | 6,884 | 61.6 |  | 2,389 | 5.3 |  | 11,245 | 20.2 |  | 2,628 | 6.5 |  | 664 | 0.6 |
| Arizona | 2017 | 11,319 | 61.5 |  | 3,117 | 5.9 |  | 17,065 | 21.2 |  | 3,747 | 6.6 |  | 854 | 0.9 |
| Arizona | 2018 | 21,027 | 60.1 |  | 6,024 | 5.7 |  | 30,768 | 20.3 |  | 8,050 | 7.0 |  | 1,540 | 1.0 |
| Arizona | 2019 | 6,920 | 57.7 |  | 2,276 | 4.9 |  | 12,023 | 19.7 |  | 3,222 | 6.5 |  | 570 | 1.1 |
| Arizona | 2020 | 1,184 | 52.1 |  | 333 | 4.8 |  | 2,627 | 17.9 |  | 660 | 7.4 |  | 88 | 0.0 |
| Arkansas | 2000 | 178 | 86.0 |  | 2,988 | 11.6 |  | 3,527 | 51.3 |  | 551 | 34.5 |  | 394 | 4.6 |
| Arkansas | 2001 | 96 | 86.5 |  | 2,675 | 10.2 |  | 3,342 | 50.3 |  | 540 | 33.3 |  | 392 | 6.6 |
| Arkansas | 2002 | 122 | 85.2 |  | 3,109 | 12.3 |  | 3,974 | 50.9 |  | 741 | 34.5 |  | 484 | 7.4 |
| Arkansas | 2003 | 156 | 88.5 |  | 3,453 | 12.0 |  | 4,079 | 47.9 |  | 833 | 28.8 |  | 509 | 4.9 |
| Arkansas | 2004 | Data not reported | |  |  |  |  |  |  |  |  |  |  |  |  |
| Arkansas | 2005 | 79 | 68.4 |  | 3,419 | 10.1 |  | 4,583 | 40.7 |  | 1,366 | 21.4 |  | 946 | 2.0 |
| Arkansas | 2006 | 84 | 78.6 |  | 3,581 | 10.6 |  | 4,206 | 40.5 |  | 1,394 | 21.4 |  | 981 | 2.1 |
| Arkansas | 2007 | 75 | 78.7 |  | 3,198 | 10.4 |  | 4,055 | 40.1 |  | 1,921 | 19.1 |  | 1,172 | 2.5 |
| Arkansas | 2008 | 173 | 82.7 |  | 5,629 | 9.8 |  | 6,715 | 44.2 |  | 4,502 | 22.4 |  | 2,735 | 2.1 |
| Arkansas | 2009 | 144 | 78.5 |  | 3,684 | 9.5 |  | 5,727 | 42.7 |  | 3,985 | 21.4 |  | 1,898 | 1.1 |
| Arkansas | 2010 | 96 | 71.9 |  | 1,874 | 8.8 |  | 3,698 | 43.2 |  | 2,467 | 26.1 |  | 1,179 | 2.0 |
| Arkansas | 2011 | 84 | 78.6 |  | 1,570 | 8.1 |  | 3,142 | 43.5 |  | 2,226 | 24.1 |  | 964 | 2.7 |
| Arkansas | 2012 | 130 | 86.9 |  | 1,427 | 7.1 |  | 3,270 | 43.0 |  | 2,883 | 23.7 |  | 1,076 | 1.9 |
| Arkansas | 2013 | 122 | 86.1 |  | 1,177 | 7.6 |  | 3,977 | 45.0 |  | 2,763 | 26.5 |  | 915 | 1.6 |
| Arkansas | 2014 | 135 | 75.6 |  | 709 | 6.9 |  | 2,949 | 41.3 |  | 1,899 | 28.8 |  | 672 | 3.0 |
| Arkansas | 2015 | 220 | 82.3 |  | 920 | 5.8 |  | 4,641 | 40.7 |  | 2,435 | 32.5 |  | 826 | 1.8 |
| Arkansas | 2016 | 295 | 76.3 |  | 1,076 | 7.1 |  | 4,998 | 39.6 |  | 2,367 | 27.9 |  | 791 | 1.9 |
| Arkansas | 2017 | 324 | 79.9 |  | 838 | 4.2 |  | 4,821 | 38.2 |  | 1,896 | 27.6 |  | 644 | 2.2 |
| Arkansas | 2018 | 389 | 77.4 |  | 795 | 4.7 |  | 4,888 | 39.8 |  | 1,897 | 28.3 |  | 680 | 2.4 |
| Arkansas | 2019 | 554 | 77.8 |  | 756 | 4.9 |  | 6,788 | 37.8 |  | 2,474 | 29.3 |  | 728 | 1.9 |
| Arkansas | 2020 | 457 | 75.7 |  | 574 | 6.3 |  | 5,370 | 36.7 |  | 2,015 | 27.8 |  | 542 | 3.1 |
| California | 2000 | 61,356 | 86.2 |  | 44,652 | 19.8 |  | 44,208 | 19.4 |  | 3,769 | 6.0 |  | 1,762 | 3.8 |
| California | 2001 | 50,923 | 87.0 |  | 40,201 | 17.0 |  | 50,453 | 17.3 |  | 3,735 | 5.7 |  | 1,732 | 4.6 |
| California | 2002 | 50,969 | 86.3 |  | 45,855 | 14.5 |  | 73,398 | 15.7 |  | 4,748 | 6.6 |  | 2,012 | 4.2 |
| California | 2003 | 41,857 | 85.2 |  | 41,973 | 11.9 |  | 74,832 | 14.1 |  | 5,286 | 7.3 |  | 1,994 | 3.9 |
| California | 2004 | 36,875 | 84.6 |  | 36,770 | 11.2 |  | 71,180 | 12.9 |  | 5,056 | 6.8 |  | 1,738 | 4.2 |
| California | 2005 | 32,173 | 84.0 |  | 31,713 | 9.5 |  | 77,514 | 11.7 |  | 4,911 | 6.6 |  | 1,638 | 2.7 |
| California | 2006 | 33,025 | 84.8 |  | 33,143 | 9.0 |  | 83,788 | 11.5 |  | 7,226 | 7.4 |  | 1,602 | 1.7 |
| California | 2007 | 32,496 | 83.7 |  | 32,051 | 7.7 |  | 83,198 | 11.4 |  | 8,768 | 6.9 |  | 1,645 | 2.0 |
| California | 2008 | 33,563 | 82.8 |  | 32,177 | 7.0 |  | 73,572 | 11.8 |  | 10,746 | 8.1 |  | 1,780 | 2.1 |
| California | 2009 | 31,304 | 81.0 |  | 25,155 | 6.8 |  | 61,317 | 12.3 |  | 11,951 | 8.4 |  | 1,897 | 1.8 |
| California | 2010 | 29,532 | 80.0 |  | 19,013 | 6.5 |  | 57,129 | 13.4 |  | 13,530 | 9.3 |  | 1,942 | 1.6 |
| California | 2011 | 30,245 | 77.7 |  | 16,317 | 6.6 |  | 53,105 | 14.6 |  | 13,499 | 9.1 |  | 2,159 | 2.3 |
| California | 2012 | 32,591 | 76.3 |  | 15,568 | 6.7 |  | 56,694 | 16.4 |  | 13,747 | 8.1 |  | 2,435 | 1.2 |
| California | 2013 | 35,552 | 75.1 |  | 13,969 | 5.8 |  | 62,193 | 17.9 |  | 13,107 | 7.7 |  | 2,298 | 1.5 |
| California | 2014 | 42,791 | 73.7 |  | 12,481 | 6.0 |  | 67,901 | 18.7 |  | 13,415 | 6.3 |  | 2,228 | 1.0 |
| California | 2015 | 40,889 | 70.7 |  | 10,281 | 6.5 |  | 65,932 | 18.5 |  | 12,490 | 6.1 |  | 2,357 | 1.1 |
| California | 2016 | 42,038 | 69.9 |  | 9,538 | 6.5 |  | 71,676 | 19.3 |  | 11,671 | 4.6 |  | 2,706 | 1.1 |
| California | 2017 | 40,877 | 68.6 |  | 9,095 | 6.4 |  | 70,625 | 18.3 |  | 9,851 | 4.1 |  | 2,759 | 1.1 |
| California | 2018 | 48,071 | 68.3 |  | 10,577 | 5.0 |  | 78,672 | 17.7 |  | 9,433 | 3.7 |  | 2,828 | 0.9 |
| California | 2019 | 40,784 | 64.7 |  | 9,293 | 4.0 |  | 73,285 | 16.5 |  | 7,289 | 4.4 |  | 2,393 | 1.0 |
| California | 2020 | 32,906 | 62.8 |  | 6,969 | 3.7 |  | 57,968 | 15.5 |  | 7,917 | 6.7 |  | 2,049 | 0.9 |
| Colorado | 2000 | 2,212 | 83.9 |  | 5,553 | 18.0 |  | 2,960 | 30.5 |  | 685 | 15.0 |  | 363 | 2.5 |
| Colorado | 2001 | 1,452 | 81.3 |  | 4,098 | 18.5 |  | 2,466 | 30.1 |  | 561 | 13.2 |  | 259 | 3.1 |
| Colorado | 2002 | 2,091 | 81.3 |  | 6,011 | 17.1 |  | 4,111 | 27.8 |  | 862 | 10.9 |  | 806 | 2.9 |
| Colorado | 2003 | 2,311 | 83.0 |  | 6,236 | 16.7 |  | 4,888 | 23.2 |  | 1,007 | 8.9 |  | 819 | 1.8 |
| Colorado | 2004 | 1,990 | 84.7 |  | 7,843 | 12.2 |  | 6,951 | 20.1 |  | 1,196 | 9.5 |  | 637 | 2.5 |
| Colorado | 2005 | 2,190 | 81.8 |  | 8,626 | 11.2 |  | 9,007 | 19.6 |  | 1,393 | 8.4 |  | 841 | 2.1 |
| Colorado | 2006 | 2,360 | 80.3 |  | 9,768 | 10.2 |  | 8,909 | 18.8 |  | 1,781 | 8.5 |  | 919 | 2.3 |
| Colorado | 2007 | 2,164 | 79.6 |  | 9,836 | 10.2 |  | 8,944 | 18.6 |  | 2,114 | 8.6 |  | 1,005 | 2.2 |
| Colorado | 2008 | 2,393 | 77.3 |  | 9,770 | 9.1 |  | 8,166 | 21.2 |  | 2,557 | 8.1 |  | 1,122 | 1.9 |
| Colorado | 2009 | 2,846 | 75.9 |  | 8,811 | 10.9 |  | 7,896 | 19.9 |  | 3,203 | 7.7 |  | 1,193 | 1.0 |
| Colorado | 2010 | 3,167 | 76.3 |  | 7,996 | 10.9 |  | 8,448 | 21.5 |  | 3,849 | 10.5 |  | 1,283 | 1.5 |
| Colorado | 2011 | 3,737 | 75.6 |  | 7,701 | 13.0 |  | 8,249 | 23.2 |  | 4,123 | 10.6 |  | 1,349 | 1.8 |
| Colorado | 2012 | 5,083 | 72.8 |  | 8,441 | 12.9 |  | 10,457 | 24.6 |  | 5,371 | 11.2 |  | 1,660 | 1.6 |
| Colorado | 2013 | 5,675 | 73.1 |  | 6,960 | 13.5 |  | 12,236 | 26.4 |  | 5,227 | 11.5 |  | 1,730 | 2.5 |
| Colorado | 2014 | 7,518 | 67.3 |  | 6,393 | 13.0 |  | 14,035 | 28.4 |  | 5,114 | 10.4 |  | 1,724 | 2.1 |
| Colorado | 2015 | 7,652 | 63.0 |  | 5,310 | 13.1 |  | 13,674 | 28.4 |  | 3,820 | 11.8 |  | 1,477 | 2.9 |
| Colorado | 2016 | 11,302 | 61.3 |  | 6,427 | 12.8 |  | 19,953 | 30.1 |  | 4,896 | 11.2 |  | 2,099 | 1.3 |
| Colorado | 2017 | 12,414 | 61.2 |  | 6,895 | 10.9 |  | 22,348 | 30.8 |  | 4,958 | 10.9 |  | 2,073 | 1.8 |
| Colorado | 2018 | 13,704 | 59.1 |  | 6,772 | 10.1 |  | 24,083 | 31.1 |  | 4,598 | 9.1 |  | 1,984 | 2.1 |
| Colorado | 2019 | 13,364 | 56.6 |  | 6,136 | 8.6 |  | 25,483 | 28.5 |  | 4,564 | 8.3 |  | 1,762 | 1.4 |
| Colorado | 2020 | 11,660 | 54.4 |  | 5,176 | 7.7 |  | 22,005 | 27.5 |  | 4,569 | 8.0 |  | 1,797 | 1.3 |
| Connecticut | 2000 | Data not reported | |  |  |  |  |  |  |  |  |  |  |  |  |
| Connecticut | 2001 | Data not reported | |  |  |  |  |  |  |  |  |  |  |  |  |
| Connecticut | 2002 | Data not reported | |  |  |  |  |  |  |  |  |  |  |  |  |
| Connecticut | 2003 | Data not reported | |  |  |  |  |  |  |  |  |  |  |  |  |
| Connecticut | 2004 | Data not reported | |  |  |  |  |  |  |  |  |  |  |  |  |
| Connecticut | 2005 | 18,057 | 62.8 |  | 17,834 | 12.8 |  | 291 | 8.2 |  | 3,663 | 3.0 |  | 1,561 | 1.7 |
| Connecticut | 2006 | 17,716 | 63.6 |  | 19,718 | 13.2 |  | 301 | 9.6 |  | 3,858 | 4.0 |  | 1,727 | 1.9 |
| Connecticut | 2007 | 17,321 | 64.0 |  | 19,766 | 13.3 |  | 380 | 5.8 |  | 4,381 | 3.7 |  | 1,748 | 1.1 |
| Connecticut | 2008 | 17,597 | 63.3 |  | 18,932 | 11.5 |  | 382 | 8.4 |  | 4,851 | 4.3 |  | 2,083 | 1.3 |
| Connecticut | 2009 | 16,842 | 65.0 |  | 16,751 | 12.5 |  | 400 | 5.3 |  | 5,038 | 4.8 |  | 2,264 | 1.4 |
| Connecticut | 2010 | 16,785 | 64.1 |  | 16,693 | 14.6 |  | 452 | 6.0 |  | 5,436 | 7.4 |  | 2,687 | 1.3 |
| Connecticut | 2011 | 19,996 | 61.7 |  | 19,221 | 14.7 |  | 498 | 5.6 |  | 6,350 | 6.7 |  | 3,373 | 1.3 |
| Connecticut | 2012 | 20,586 | 61.7 |  | 18,608 | 14.5 |  | 501 | 5.8 |  | 6,595 | 6.2 |  | 3,568 | 1.1 |
| Connecticut | 2013 | 23,661 | 63.5 |  | 17,080 | 14.4 |  | 487 | 6.8 |  | 6,826 | 4.6 |  | 3,435 | 1.4 |
| Connecticut | 2014 | 26,203 | 63.8 |  | 17,277 | 12.3 |  | 492 | 8.1 |  | 7,088 | 4.2 |  | 3,871 | 1.4 |
| Connecticut | 2015 | 28,193 | 62.8 |  | 17,113 | 12.2 |  | 455 | 7.7 |  | 6,763 | 3.7 |  | 3,762 | 1.3 |
| Connecticut | 2016 | 29,235 | 61.5 |  | 17,881 | 11.3 |  | 457 | 8.3 |  | 6,467 | 4.3 |  | 3,957 | 1.0 |
| Connecticut | 2017 | 28,620 | 57.6 |  | 18,485 | 10.0 |  | 481 | 8.1 |  | 6,281 | 4.6 |  | 4,061 | 0.5 |
| Connecticut | 2018 | 26,274 | 56.1 |  | 19,528 | 9.2 |  | 540 | 8.0 |  | 6,026 | 5.6 |  | 3,792 | 0.6 |
| Connecticut | 2019 | 23,533 | 50.9 |  | 18,847 | 8.5 |  | 640 | 9.2 |  | 6,229 | 11.9 |  | 3,378 | 0.4 |
| Connecticut | 2020 | 16,873 | 47.3 |  | 12,969 | 8.5 |  | 535 | 10.8 |  | 5,001 | 15.6 |  | 2,192 | 0.8 |
| Delaware | 2000 | 2,177 | 44.7 |  | 2,677 | 7.4 |  | 55 | 29.1 |  | 178 | 10.7 |  | 108 | 0.9 |
| Delaware | 2001 | 2,075 | 42.4 |  | 2,746 | 6.3 |  | 68 | 14.7 |  | 222 | 4.5 |  | 126 | 1.6 |
| Delaware | 2002 | 1,898 | 44.8 |  | 2,922 | 5.3 |  | 85 | 15.3 |  | 325 | 7.4 |  | 133 | 1.5 |
| Delaware | 2003 | 2,216 | 45.8 |  | 3,211 | 6.0 |  | 82 | 26.8 |  | 445 | 3.8 |  | 149 | 1.3 |
| Delaware | 2004 | 2,388 | 51.5 |  | 3,231 | 7.5 |  | 75 | 22.7 |  | 574 | 4.7 |  | 180 | 1.7 |
| Delaware | 2005 | 2,336 | 49.5 |  | 3,458 | 6.6 |  | 96 | 13.5 |  | 706 | 3.4 |  | 230 | 0.9 |
| Delaware | 2006 | 2,320 | 51.8 |  | 3,511 | 6.8 |  | 105 | 14.3 |  | 1,080 | 4.5 |  | 291 | 1.4 |
| Delaware | 2007 | 2,270 | 56.1 |  | 3,391 | 6.0 |  | 88 | 18.2 |  | 1,393 | 7.8 |  | 371 | 1.1 |
| Delaware | 2008 | 2,145 | 53.6 |  | 2,915 | 5.2 |  | 88 | 17.0 |  | 1,707 | 7.4 |  | 506 | 0.2 |
| Delaware | 2009 | 1,957 | 55.9 |  | 2,227 | 6.1 |  | 55 | 14.5 |  | 2,090 | 10.2 |  | 536 | 1.3 |
| Delaware | 2010 | 1,593 | 59.4 |  | 1,599 | 8.3 |  | 53 | 17.0 |  | 1,960 | 11.4 |  | 543 | 2.4 |
| Delaware | 2011 | 1,590 | 63.5 |  | 1,475 | 8.7 |  | 67 | 7.5 |  | 2,294 | 12.0 |  | 483 | 1.9 |
| Delaware | 2012 | 2,517 | 63.2 |  | 1,602 | 10.7 |  | 79 | 7.6 |  | 2,290 | 12.0 |  | 522 | 1.5 |
| Delaware | 2013 | 2,682 | 60.7 |  | 1,243 | 12.1 |  | 81 | 12.3 |  | 1,754 | 9.5 |  | 419 | 0.5 |
| Delaware | 2014 | 3,685 | 62.1 |  | 1,458 | 12.3 |  | 96 | 16.7 |  | 1,614 | 9.8 |  | 456 | 1.5 |
| Delaware | 2015 | 2,969 | 64.8 |  | 1,074 | 15.5 |  | 76 | 18.4 |  | 1,052 | 7.5 |  | 318 | 1.9 |
| Delaware | 2016 | 2,596 | 65.1 |  | 1,241 | 15.1 |  | 86 | 30.2 |  | 728 | 8.8 |  | 310 | 1.0 |
| Delaware | 2017 | 5,810 | 62.9 |  | 3,351 | 13.9 |  | 259 | 25.1 |  | 1,283 | 7.5 |  | 502 | 0.4 |
| Delaware | 2018 | 9,515 | 57.0 |  | 5,683 | 13.9 |  | 481 | 29.7 |  | 2,343 | 8.1 |  | 717 | 1.5 |
| Delaware | 2019 | 8,437 | 49.7 |  | 4,792 | 11.9 |  | 585 | 38.8 |  | 1,951 | 11.1 |  | 515 | 0.0 |
| Delaware | 2020 | 6,837 | 48.3 |  | 3,603 | 10.6 |  | 673 | 38.3 |  | 1,398 | 15.9 |  | 434 | 2.5 |
| District of Columbia | 2000 | 2,436 | 45.2 |  | 3,816 | 9.1 |  | 131 | 4.6 |  | 33 | 3.0 |  | 11 | 0.0 |
| District of Columbia | 2001 | 2,348 | 28.5 |  | 3,482 | 6.3 |  | 51 | 21.6 |  | 36 | 8.3 |  | 12 | 0.0 |
| District of Columbia | 2002 | 2,407 | 25.1 |  | 3,353 | 7.2 |  | 34 | 35.3 |  | 31 | 9.7 |  | 0 |  |
| District of Columbia | 2003 | 2,158 | 28.1 |  | 2,493 | 5.3 |  | 11 | 27.3 |  | 33 | 3.0 |  | 0 |  |
| District of Columbia | 2004 | Data not reported | |  |  |  |  |  |  |  |  |  |  |  |  |
| District of Columbia | 2005 | Data not reported | |  |  |  |  |  |  |  |  |  |  |  |  |
| District of Columbia | 2006 | Data not reported | |  |  |  |  |  |  |  |  |  |  |  |  |
| District of Columbia | 2007 | 46 | 34.8 |  | 71 | 5.6 |  | 0 |  |  | 0 |  |  | 0 |  |
| District of Columbia | 2008 | 528 | 36.0 |  | 780 | 1.2 |  | 11 | 9.1 |  | 7 | 28.6 |  | 0 |  |
| District of Columbia | 2009 | Data not reported | |  |  |  |  |  |  |  |  |  |  |  |  |
| District of Columbia | 2010 | 1,284 | 47.9 |  | 1,207 | 3.8 |  | 16 | 6.3 |  | 71 | 16.9 |  | 14 | 0.0 |
| District of Columbia | 2011 | 2,043 | 34.8 |  | 3,164 | 1.3 |  | 55 | 16.4 |  | 140 | 5.7 |  | 28 | 3.6 |
| District of Columbia | 2012 | 1,460 | 32.2 |  | 2,448 | 1.2 |  | 47 | 21.3 |  | 116 | 2.6 |  | 16 | 6.3 |
| District of Columbia | 2013 | 1,454 | 35.1 |  | 2,226 | 1.5 |  | 42 | 19.0 |  | 124 | 7.3 |  | 15 | 0.0 |
| District of Columbia | 2014 | 1,620 | 33.0 |  | 2,102 | 2.0 |  | 61 | 6.6 |  | 141 | 7.1 |  | 26 | 0.0 |
| District of Columbia | 2015 | 1,929 | 30.3 |  | 2,201 | 1.8 |  | 67 | 6.0 |  | 268 | 4.9 |  | 42 | 0.0 |
| District of Columbia | 2016 | 2,002 | 31.1 |  | 1,854 | 1.2 |  | 50 | 18.0 |  | 223 | 9.4 |  | 40 | 0.0 |
| District of Columbia | 2017 | 1,979 | 25.0 |  | 2,227 | 1.9 |  | 89 | 11.2 |  | 249 | 5.2 |  | 33 | 0.0 |
| District of Columbia | 2018 | 1,825 | 22.1 |  | 2,250 | 1.1 |  | 88 | 5.7 |  | 202 | 0.0 |  | 18 | 0.0 |
| District of Columbia | 2019 | 1,800 | 16.2 |  | 2,166 | 1.1 |  | 92 | 21.7 |  | 210 | 2.4 |  | 33 | 0.0 |
| District of Columbia | 2020 | 877 | 15.5 |  | 1,161 | 0.7 |  | 78 | 9.0 |  | 131 | 1.5 |  | 17 | 0.0 |
| Florida | 2000 | 4,970 | 73.9 |  | 25,803 | 2.9 |  | 916 | 12.2 |  | 4,033 | 16.9 |  | 2,067 | 0.8 |
| Florida | 2001 | 4,328 | 75.1 |  | 19,584 | 3.0 |  | 975 | 12.4 |  | 4,523 | 17.3 |  | 2,031 | 1.0 |
| Florida | 2002 | 5,919 | 74.8 |  | 25,087 | 3.5 |  | 1,375 | 16.3 |  | 5,999 | 17.4 |  | 2,544 | 1.1 |
| Florida | 2003 | 4,830 | 73.3 |  | 23,985 | 3.6 |  | 1,776 | 19.8 |  | 5,071 | 15.8 |  | 2,389 | 0.7 |
| Florida | 2004 | Data not reported | |  |  |  |  |  |  |  |  |  |  |  |  |
| Florida | 2005 | 2,083 | 70.6 |  | 17,180 | 3.4 |  | 1,954 | 14.9 |  | 3,995 | 11.9 |  | 2,095 | 0.9 |
| Florida | 2006 | 2,562 | 69.8 |  | 18,382 | 4.0 |  | 2,016 | 11.0 |  | 5,701 | 12.2 |  | 2,427 | 1.4 |
| Florida | 2007 | 2,061 | 70.4 |  | 18,321 | 4.3 |  | 1,878 | 11.9 |  | 6,475 | 12.3 |  | 2,672 | 1.2 |
| Florida | 2008 | 2,799 | 74.1 |  | 24,340 | 3.3 |  | 2,378 | 12.1 |  | 11,834 | 12.1 |  | 4,620 | 0.7 |
| Florida | 2009 | 2,670 | 72.3 |  | 19,611 | 3.6 |  | 2,224 | 13.7 |  | 16,333 | 16.4 |  | 6,020 | 1.5 |
| Florida | 2010 | 2,231 | 70.8 |  | 12,561 | 4.4 |  | 2,125 | 14.2 |  | 18,416 | 20.4 |  | 5,942 | 2.1 |
| Florida | 2011 | 3,126 | 78.1 |  | 19,071 | 6.4 |  | 3,249 | 16.9 |  | 31,526 | 26.7 |  | 12,919 | 3.5 |
| Florida | 2012 | 2,810 | 78.1 |  | 11,762 | 8.4 |  | 2,524 | 22.7 |  | 20,181 | 35.4 |  | 8,069 | 6.5 |
| Florida | 2013 | 3,442 | 80.9 |  | 8,171 | 14.2 |  | 2,344 | 28.0 |  | 15,642 | 46.0 |  | 5,700 | 7.0 |
| Florida | 2014 | 3,941 | 80.8 |  | 5,871 | 16.5 |  | 1,960 | 35.2 |  | 10,528 | 45.3 |  | 3,718 | 4.9 |
| Florida | 2015 | 5,191 | 77.2 |  | 4,937 | 16.7 |  | 1,935 | 33.9 |  | 8,658 | 44.9 |  | 3,247 | 4.7 |
| Florida | 2016 | 6,106 | 73.0 |  | 5,259 | 18.2 |  | 2,144 | 33.8 |  | 8,187 | 42.9 |  | 3,277 | 3.3 |
| Florida | 2017 | 11,312 | 75.8 |  | 9,766 | 16.2 |  | 4,921 | 33.8 |  | 11,972 | 37.2 |  | 5,391 | 3.7 |
| Florida | 2018 | 19,283 | 75.6 |  | 18,350 | 11.9 |  | 9,814 | 31.7 |  | 20,384 | 32.6 |  | 6,708 | 3.0 |
| Florida | 2019 | 7,142 | 71.2 |  | 5,453 | 5.8 |  | 3,722 | 25.1 |  | 6,308 | 31.5 |  | 980 | 1.8 |
| Florida | 2020 | 5,773 | 64.8 |  | 4,199 | 7.4 |  | 2,837 | 22.7 |  | 4,921 | 35.1 |  | 710 | 3.0 |
| Georgia | 2000 | 577 | 57.7 |  | 10,221 | 2.5 |  | 978 | 19.8 |  | 496 | 8.9 |  | 618 | 1.9 |
| Georgia | 2001 | 831 | 57.9 |  | 12,233 | 2.4 |  | 1,478 | 17.9 |  | 877 | 12.0 |  | 759 | 0.4 |
| Georgia | 2002 | 759 | 61.8 |  | 10,691 | 2.8 |  | 2,114 | 16.9 |  | 1,001 | 14.0 |  | 831 | 1.7 |
| Georgia | 2003 | 791 | 56.9 |  | 12,237 | 2.7 |  | 3,439 | 15.1 |  | 1,348 | 12.5 |  | 1,090 | 0.9 |
| Georgia | 2004 | 665 | 57.1 |  | 11,070 | 2.6 |  | 3,688 | 13.8 |  | 1,176 | 11.1 |  | 1,007 | 1.1 |
| Georgia | 2005 | 876 | 60.6 |  | 16,728 | 2.4 |  | 6,927 | 14.0 |  | 3,300 | 7.8 |  | 1,759 | 0.6 |
| Georgia | 2006 | 1,056 | 61.4 |  | 17,157 | 2.8 |  | 4,651 | 13.7 |  | 3,117 | 8.1 |  | 1,689 | 0.5 |
| Georgia | 2007 | 864 | 58.1 |  | 16,209 | 2.5 |  | 4,541 | 12.2 |  | 2,811 | 5.5 |  | 2,084 | 0.5 |
| Georgia | 2008 | 854 | 61.5 |  | 14,564 | 2.4 |  | 3,489 | 14.2 |  | 3,107 | 6.8 |  | 2,153 | 0.7 |
| Georgia | 2009 | 854 | 63.3 |  | 12,856 | 2.2 |  | 3,762 | 14.3 |  | 3,791 | 7.8 |  | 2,560 | 0.4 |
| Georgia | 2010 | 882 | 70.5 |  | 11,476 | 2.8 |  | 4,138 | 16.5 |  | 4,803 | 10.7 |  | 3,083 | 1.1 |
| Georgia | 2011 | 844 | 70.6 |  | 11,596 | 2.9 |  | 4,194 | 16.7 |  | 5,153 | 10.4 |  | 3,413 | 1.1 |
| Georgia | 2012 | 1,098 | 70.8 |  | 11,311 | 2.5 |  | 5,183 | 19.1 |  | 5,551 | 11.0 |  | 3,504 | 1.6 |
| Georgia | 2013 | 1,426 | 75.2 |  | 10,928 | 2.9 |  | 6,498 | 20.3 |  | 5,455 | 11.9 |  | 3,457 | 1.7 |
| Georgia | 2014 | 1,824 | 77.5 |  | 10,874 | 2.8 |  | 7,888 | 23.3 |  | 5,447 | 13.0 |  | 3,274 | 1.8 |
| Georgia | 2015 | 2,271 | 76.5 |  | 10,511 | 3.4 |  | 9,347 | 24.4 |  | 5,576 | 14.3 |  | 3,199 | 1.7 |
| Georgia | 2016 | 1,664 | 72.4 |  | 9,222 | 3.3 |  | 8,672 | 23.0 |  | 5,478 | 21.6 |  | 2,301 | 2.0 |
| Georgia | 2017 | 1,916 | 72.2 |  | 8,643 | 4.3 |  | 8,945 | 24.2 |  | 4,972 | 22.4 |  | 2,206 | 1.7 |
| Georgia | 2018 | 2,378 | 71.2 |  | 8,215 | 4.7 |  | 9,840 | 24.9 |  | 4,677 | 19.4 |  | 1,964 | 1.4 |
| Georgia | 2019 | 2,604 | 66.4 |  | 7,794 | 3.6 |  | 10,449 | 24.0 |  | 4,237 | 18.6 |  | 1,666 | 1.4 |
| Georgia | 2020 | 2,534 | 64.5 |  | 6,389 | 2.9 |  | 9,621 | 22.8 |  | 3,560 | 19.5 |  | 1,322 | 1.7 |
| Hawaii | 2000 | 449 | 86.0 |  | 1,303 | 7.1 |  | 2,311 | 0.9 |  | 176 | 5.7 |  | 110 | 0.0 |
| Hawaii | 2001 | 274 | 79.9 |  | 1,055 | 6.3 |  | 2,578 | 1.4 |  | 193 | 11.9 |  | 117 | 3.4 |
| Hawaii | 2002 | 307 | 77.5 |  | 910 | 6.9 |  | 2,783 | 1.9 |  | 183 | 13.1 |  | 79 | 0.0 |
| Hawaii | 2003 | 236 | 81.4 |  | 839 | 5.7 |  | 3,052 | 2.1 |  | 187 | 14.4 |  | 68 | 1.5 |
| Hawaii | 2004 | 213 | 85.9 |  | 686 | 7.3 |  | 2,771 | 2.2 |  | 223 | 13.5 |  | 58 | 0.0 |
| Hawaii | 2005 | 250 | 87.6 |  | 637 | 6.3 |  | 3,099 | 3.2 |  | 262 | 16.0 |  | 63 | 4.8 |
| Hawaii | 2006 | 212 | 85.8 |  | 829 | 4.5 |  | 2,746 | 2.3 |  | 249 | 13.3 |  | 56 | 0.0 |
| Hawaii | 2007 | 200 | 88.5 |  | 776 | 6.7 |  | 2,707 | 2.7 |  | 302 | 21.2 |  | 82 | 0.0 |
| Hawaii | 2008 | 195 | 82.6 |  | 722 | 3.3 |  | 2,443 | 2.5 |  | 338 | 13.9 |  | 65 | 0.0 |
| Hawaii | 2009 | 165 | 83.6 |  | 644 | 5.7 |  | 2,480 | 4.0 |  | 359 | 17.5 |  | 91 | 0.0 |
| Hawaii | 2010 | 130 | 87.7 |  | 509 | 6.1 |  | 2,295 | 3.4 |  | 409 | 19.8 |  | 139 | 2.9 |
| Hawaii | 2011 | 148 | 85.1 |  | 535 | 7.7 |  | 2,423 | 5.7 |  | 478 | 17.4 |  | 131 | 1.5 |
| Hawaii | 2012 | 163 | 92.0 |  | 514 | 5.4 |  | 2,573 | 5.8 |  | 536 | 19.0 |  | 105 | 0.0 |
| Hawaii | 2013 | 173 | 80.9 |  | 421 | 5.5 |  | 2,623 | 7.6 |  | 493 | 21.9 |  | 99 | 9.1 |
| Hawaii | 2014 | 241 | 73.0 |  | 425 | 5.9 |  | 2,765 | 7.6 |  | 512 | 20.9 |  | 101 | 5.9 |
| Hawaii | 2015 | 290 | 74.1 |  | 415 | 5.1 |  | 2,942 | 9.0 |  | 493 | 17.2 |  | 85 | 8.2 |
| Hawaii | 2016 | 309 | 74.8 |  | 386 | 3.4 |  | 3,063 | 9.9 |  | 466 | 20.4 |  | 94 | 8.5 |
| Hawaii | 2017 | 345 | 68.1 |  | 370 | 5.9 |  | 2,739 | 11.3 |  | 406 | 19.5 |  | 89 | 6.7 |
| Hawaii | 2018 | 266 | 71.8 |  | 219 | 4.1 |  | 1,768 | 15.3 |  | 251 | 17.5 |  | 61 | 1.6 |
| Hawaii | 2019 | 265 | 66.8 |  | 169 | 5.9 |  | 1,470 | 18.6 |  | 187 | 17.1 |  | 26 | 0.0 |
| Hawaii | 2020 | 228 | 55.3 |  | 102 | 3.9 |  | 970 | 17.6 |  | 121 | 18.2 |  | 31 | 0.0 |
| Idaho | 2000 | 189 | 89.9 |  | 595 | 33.9 |  | 2,104 | 31.3 |  | 110 | 2.7 |  | 59 | 3.4 |
| Idaho | 2001 | 198 | 90.4 |  | 695 | 33.5 |  | 3,097 | 35.4 |  | 174 | 5.2 |  | 72 | 1.4 |
| Idaho | 2002 | 92 | 89.1 |  | 385 | 31.2 |  | 2,024 | 36.5 |  | 127 | 8.7 |  | 34 | 0.0 |
| Idaho | 2003 | 57 | 80.7 |  | 220 | 24.1 |  | 1,189 | 29.9 |  | 85 | 8.2 |  | 15 | 0.0 |
| Idaho | 2004 | 115 | 79.1 |  | 402 | 23.6 |  | 2,702 | 30.1 |  | 244 | 14.8 |  | 38 | 0.0 |
| Idaho | 2005 | 103 | 89.3 |  | 288 | 19.8 |  | 2,949 | 31.0 |  | 274 | 14.2 |  | 53 | 1.9 |
| Idaho | 2006 | 140 | 91.4 |  | 445 | 28.8 |  | 3,757 | 31.0 |  | 417 | 17.0 |  | 61 | 1.6 |
| Idaho | 2007 | 219 | 87.2 |  | 429 | 36.8 |  | 3,274 | 46.8 |  | 409 | 35.7 |  | 47 | 6.4 |
| Idaho | 2008 | 156 | 76.9 |  | 404 | 25.7 |  | 2,370 | 32.9 |  | 444 | 21.4 |  | 51 | 11.8 |
| Idaho | 2009 | 235 | 85.5 |  | 396 | 21.2 |  | 2,407 | 39.0 |  | 608 | 24.2 |  | 86 | 5.8 |
| Idaho | 2010 | 193 | 90.2 |  | 208 | 37.0 |  | 1,711 | 49.6 |  | 602 | 39.4 |  | 79 | 2.5 |
| Idaho | 2011 | 210 | 87.6 |  | 209 | 33.5 |  | 1,753 | 46.8 |  | 691 | 35.3 |  | 71 | 4.2 |
| Idaho | 2012 | 328 | 84.5 |  | 253 | 26.1 |  | 2,446 | 43.1 |  | 949 | 35.4 |  | 119 | 3.4 |
| Idaho | 2013 | 388 | 61.9 |  | 188 | 20.7 |  | 2,205 | 38.0 |  | 629 | 30.8 |  | 109 | 6.4 |
| Idaho | 2014 | 338 | 75.1 |  | 87 | 24.1 |  | 1,295 | 50.3 |  | 309 | 39.5 |  | 64 | 6.3 |
| Idaho | 2015 | 345 | 74.5 |  | 117 | 29.1 |  | 1,314 | 50.7 |  | 314 | 29.9 |  | 52 | 3.8 |
| Idaho | 2016 | 402 | 76.6 |  | 83 | 26.5 |  | 1,518 | 49.7 |  | 265 | 34.0 |  | 48 | 8.3 |
| Idaho | 2017 | 447 | 81.0 |  | 65 | 20.0 |  | 1,538 | 50.2 |  | 215 | 23.3 |  | 49 | 8.2 |
| Idaho | 2018 | 228 | 71.9 |  | 38 | 26.3 |  | 843 | 42.0 |  | 120 | 20.0 |  | 8 | 12.5 |
| Idaho | 2019 | 128 | 73.4 |  | 20 | 25.0 |  | 365 | 42.5 |  | 47 | 25.5 |  | 5 | 20.0 |
| Idaho | 2020 | Data not reported | |  |  |  |  |  |  |  |  |  |  |  |  |
| Illinois | 2000 | 10,748 | 21.5 |  | 22,753 | 3.2 |  | 2,461 | 4.3 |  | 877 | 13.0 |  | 401 | 1.0 |
| Illinois | 2001 | 12,310 | 20.5 |  | 26,731 | 2.9 |  | 2,914 | 4.5 |  | 929 | 12.1 |  | 432 | 0.7 |
| Illinois | 2002 | 13,537 | 18.9 |  | 27,664 | 2.7 |  | 3,416 | 6.7 |  | 987 | 12.6 |  | 418 | 0.7 |
| Illinois | 2003 | 16,546 | 20.9 |  | 29,040 | 2.4 |  | 4,012 | 8.3 |  | 1,101 | 12.2 |  | 479 | 0.8 |
| Illinois | 2004 | 14,294 | 20.5 |  | 26,933 | 2.3 |  | 4,423 | 10.5 |  | 1,108 | 12.6 |  | 512 | 1.0 |
| Illinois | 2005 | 13,608 | 21.8 |  | 25,590 | 2.2 |  | 4,402 | 8.4 |  | 1,187 | 13.6 |  | 489 | 0.4 |
| Illinois | 2006 | 20,434 | 23.8 |  | 28,574 | 2.3 |  | 4,486 | 9.9 |  | 2,068 | 11.8 |  | 629 | 0.6 |
| Illinois | 2007 | 18,217 | 23.5 |  | 27,265 | 2.2 |  | 2,857 | 10.6 |  | 2,319 | 11.0 |  | 718 | 0.8 |
| Illinois | 2008 | 21,102 | 27.4 |  | 26,061 | 2.1 |  | 2,269 | 11.8 |  | 2,714 | 10.0 |  | 925 | 0.9 |
| Illinois | 2009 | 21,002 | 30.7 |  | 22,490 | 2.1 |  | 2,297 | 11.8 |  | 2,961 | 9.6 |  | 1,237 | 0.8 |
| Illinois | 2010 | 19,996 | 38.1 |  | 20,425 | 2.4 |  | 2,959 | 13.4 |  | 3,938 | 10.8 |  | 1,627 | 0.6 |
| Illinois | 2011 | 17,068 | 39.3 |  | 16,355 | 2.8 |  | 2,600 | 17.7 |  | 3,701 | 9.6 |  | 1,650 | 0.6 |
| Illinois | 2012 | 10,272 | 40.6 |  | 8,955 | 2.8 |  | 1,793 | 21.4 |  | 2,698 | 10.8 |  | 1,223 | 0.4 |
| Illinois | 2013 | 9,348 | 42.7 |  | 7,617 | 2.7 |  | 1,779 | 22.7 |  | 2,648 | 13.1 |  | 1,137 | 1.2 |
| Illinois | 2014 | 8,530 | 43.9 |  | 6,113 | 3.0 |  | 1,814 | 23.2 |  | 2,227 | 11.3 |  | 1,031 | 0.8 |
| Illinois | 2015 | 17,729 | 41.5 |  | 10,888 | 3.1 |  | 7,098 | 12.0 |  | 2,935 | 10.7 |  | 1,648 | 4.3 |
| Illinois | 2016 | 9,915 | 45.0 |  | 5,706 | 3.3 |  | 4,483 | 16.7 |  | 2,064 | 11.0 |  | 1,119 | 3.4 |
| Illinois | 2017 | 17,487 | 42.4 |  | 10,502 | 4.1 |  | 7,736 | 19.3 |  | 2,727 | 11.8 |  | 1,670 | 3.7 |
| Illinois | 2018 | 19,165 | 37.3 |  | 12,744 | 3.6 |  | 9,903 | 19.7 |  | 3,139 | 10.7 |  | 1,843 | 3.6 |
| Illinois | 2019 | 16,395 | 37.2 |  | 12,209 | 3.2 |  | 12,137 | 20.5 |  | 3,163 | 15.2 |  | 1,933 | 0.7 |
| Illinois | 2020 | 10,755 | 35.8 |  | 7,844 | 2.7 |  | 9,619 | 20.6 |  | 2,598 | 17.7 |  | 1,433 | 0.5 |
| Indiana | 2000 | 1,745 | 73.9 |  | 8,971 | 7.4 |  | 1,765 | 16.9 |  | 2,028 | 17.4 |  | 1,802 | 1.3 |
| Indiana | 2001 | 753 | 72.2 |  | 6,229 | 6.0 |  | 1,740 | 16.8 |  | 1,663 | 13.8 |  | 1,349 | 1.5 |
| Indiana | 2002 | 840 | 65.5 |  | 6,524 | 6.4 |  | 2,426 | 13.7 |  | 1,881 | 12.0 |  | 1,467 | 1.3 |
| Indiana | 2003 | 825 | 69.8 |  | 6,999 | 6.1 |  | 2,808 | 13.1 |  | 2,285 | 11.4 |  | 1,580 | 1.5 |
| Indiana | 2004 | 1,148 | 67.1 |  | 8,428 | 5.4 |  | 3,781 | 11.8 |  | 2,911 | 10.4 |  | 1,910 | 1.5 |
| Indiana | 2005 | 933 | 68.9 |  | 6,738 | 4.9 |  | 3,332 | 12.2 |  | 2,541 | 11.0 |  | 1,667 | 1.4 |
| Indiana | 2006 | 1,233 | 68.4 |  | 9,503 | 5.3 |  | 4,353 | 12.3 |  | 3,962 | 9.7 |  | 2,324 | 0.9 |
| Indiana | 2007 | 1,091 | 71.5 |  | 8,371 | 4.6 |  | 3,474 | 12.6 |  | 4,054 | 7.9 |  | 2,337 | 0.6 |
| Indiana | 2008 | 1,470 | 70.1 |  | 7,067 | 4.3 |  | 3,388 | 14.9 |  | 4,577 | 8.0 |  | 2,332 | 0.5 |
| Indiana | 2009 | 1,629 | 72.4 |  | 5,289 | 4.0 |  | 3,219 | 13.7 |  | 4,054 | 9.8 |  | 2,253 | 0.3 |
| Indiana | 2010 | 1,534 | 76.0 |  | 3,580 | 4.3 |  | 2,668 | 16.0 |  | 3,813 | 8.4 |  | 1,848 | 1.0 |
| Indiana | 2011 | 2,175 | 66.3 |  | 3,827 | 4.8 |  | 3,332 | 16.5 |  | 4,639 | 10.3 |  | 2,006 | 1.3 |
| Indiana | 2012 | 2,522 | 70.8 |  | 3,597 | 4.4 |  | 3,439 | 17.9 |  | 5,412 | 10.0 |  | 2,246 | 0.6 |
| Indiana | 2013 | 3,081 | 79.1 |  | 3,082 | 5.7 |  | 3,730 | 21.3 |  | 5,610 | 11.4 |  | 2,035 | 0.8 |
| Indiana | 2014 | 4,099 | 77.5 |  | 2,799 | 8.3 |  | 4,371 | 26.0 |  | 5,816 | 11.5 |  | 2,037 | 1.0 |
| Indiana | 2015 | 4,218 | 74.5 |  | 2,400 | 9.2 |  | 4,169 | 27.2 |  | 4,740 | 12.8 |  | 1,654 | 0.7 |
| Indiana | 2016 | 4,849 | 72.3 |  | 2,595 | 10.6 |  | 5,357 | 30.0 |  | 4,538 | 12.3 |  | 1,541 | 1.8 |
| Indiana | 2017 | 6,000 | 72.2 |  | 3,158 | 9.2 |  | 7,606 | 33.3 |  | 5,169 | 10.3 |  | 1,618 | 1.7 |
| Indiana | 2018 | 7,511 | 71.8 |  | 4,120 | 8.1 |  | 11,402 | 34.4 |  | 6,010 | 10.3 |  | 1,994 | 1.0 |
| Indiana | 2019 | 6,744 | 71.3 |  | 3,438 | 7.9 |  | 11,475 | 33.9 |  | 5,058 | 10.0 |  | 1,730 | 0.8 |
| Indiana | 2020 | 5,673 | 69.4 |  | 2,508 | 7.8 |  | 9,992 | 34.9 |  | 3,763 | 11.0 |  | 1,449 | 1.0 |
| Iowa | 2000 | 369 | 56.1 |  | 3,654 | 9.1 |  | 5,632 | 19.0 |  | 333 | 18.0 |  | 287 | 2.1 |
| Iowa | 2001 | 361 | 52.6 |  | 3,600 | 8.6 |  | 6,741 | 18.4 |  | 429 | 18.4 |  | 304 | 3.6 |
| Iowa | 2002 | 395 | 51.1 |  | 3,535 | 7.8 |  | 7,350 | 18.1 |  | 575 | 18.6 |  | 328 | 2.4 |
| Iowa | 2003 | 375 | 48.5 |  | 3,616 | 7.6 |  | 7,937 | 18.2 |  | 536 | 18.5 |  | 313 | 2.2 |
| Iowa | 2004 | 379 | 63.6 |  | 3,990 | 8.8 |  | 8,403 | 22.5 |  | 723 | 16.7 |  | 333 | 3.3 |
| Iowa | 2005 | 338 | 67.5 |  | 3,993 | 8.1 |  | 8,538 | 20.2 |  | 756 | 15.1 |  | 358 | 1.7 |
| Iowa | 2006 | 338 | 60.1 |  | 4,523 | 8.4 |  | 7,332 | 20.6 |  | 883 | 16.9 |  | 311 | 3.5 |
| Iowa | 2007 | 303 | 67.0 |  | 4,232 | 6.2 |  | 6,196 | 18.3 |  | 863 | 15.6 |  | 342 | 0.9 |
| Iowa | 2008 | 288 | 61.8 |  | 3,663 | 6.3 |  | 5,195 | 20.5 |  | 1,076 | 12.7 |  | 423 | 1.4 |
| Iowa | 2009 | 386 | 64.2 |  | 3,460 | 7.4 |  | 6,371 | 22.0 |  | 1,732 | 17.6 |  | 671 | 0.9 |
| Iowa | 2010 | 568 | 66.0 |  | 3,290 | 8.5 |  | 7,633 | 22.1 |  | 2,438 | 18.9 |  | 832 | 0.6 |
| Iowa | 2011 | 445 | 75.1 |  | 2,434 | 6.2 |  | 6,416 | 20.5 |  | 2,134 | 21.3 |  | 762 | 1.6 |
| Iowa | 2012 | 629 | 75.2 |  | 2,206 | 8.3 |  | 7,496 | 22.4 |  | 2,436 | 22.7 |  | 832 | 1.0 |
| Iowa | 2013 | 847 | 75.6 |  | 2,072 | 6.1 |  | 8,817 | 22.1 |  | 2,765 | 21.5 |  | 989 | 1.3 |
| Iowa | 2014 | 941 | 76.1 |  | 1,877 | 6.3 |  | 9,242 | 22.2 |  | 2,676 | 22.6 |  | 880 | 1.7 |
| Iowa | 2015 | 1,234 | 76.9 |  | 1,799 | 7.7 |  | 9,836 | 23.3 |  | 2,763 | 23.3 |  | 987 | 1.0 |
| Iowa | 2016 | 1,314 | 77.0 |  | 1,555 | 5.9 |  | 10,410 | 23.5 |  | 2,659 | 22.5 |  | 964 | 1.8 |
| Iowa | 2017 | 1,324 | 73.1 |  | 1,667 | 5.6 |  | 11,272 | 24.0 |  | 2,503 | 21.3 |  | 935 | 1.4 |
| Iowa | 2018 | 1,527 | 72.1 |  | 1,701 | 6.5 |  | 11,965 | 24.6 |  | 2,401 | 17.8 |  | 997 | 0.7 |
| Iowa | 2019 | 1,505 | 73.8 |  | 1,424 | 5.8 |  | 12,015 | 24.4 |  | 2,255 | 18.6 |  | 820 | 0.9 |
| Iowa | 2020 | 1,412 | 71.0 |  | 1,293 | 5.7 |  | 10,268 | 26.3 |  | 1,707 | 15.5 |  | 651 | 1.2 |
| Kansas | 2000 | 210 | 87.1 |  | 4,792 | 11.9 |  | 2,323 | 42.3 |  | 229 | 20.5 |  | 236 | 1.3 |
| Kansas | 2001 | 179 | 85.5 |  | 4,590 | 11.3 |  | 2,613 | 39.5 |  | 255 | 18.8 |  | 292 | 3.8 |
| Kansas | 2002 | 182 | 81.9 |  | 4,813 | 11.3 |  | 2,945 | 35.6 |  | 324 | 20.7 |  | 286 | 4.9 |
| Kansas | 2003 | 192 | 80.2 |  | 4,742 | 12.1 |  | 3,033 | 36.8 |  | 423 | 18.9 |  | 270 | 3.7 |
| Kansas | 2004 | 202 | 82.7 |  | 4,896 | 11.5 |  | 3,575 | 34.9 |  | 565 | 21.6 |  | 324 | 3.7 |
| Kansas | 2005 | 204 | 75.0 |  | 4,901 | 10.2 |  | 3,961 | 31.9 |  | 625 | 16.6 |  | 288 | 3.1 |
| Kansas | 2006 | 183 | 76.0 |  | 4,269 | 9.3 |  | 3,037 | 31.4 |  | 534 | 17.8 |  | 265 | 2.3 |
| Kansas | 2007 | 221 | 82.4 |  | 4,634 | 9.1 |  | 3,663 | 32.9 |  | 727 | 15.8 |  | 298 | 1.7 |
| Kansas | 2008 | 264 | 82.6 |  | 4,684 | 10.9 |  | 3,714 | 34.0 |  | 1,088 | 14.2 |  | 449 | 2.0 |
| Kansas | 2009 | 289 | 86.2 |  | 4,320 | 9.3 |  | 4,137 | 35.7 |  | 1,549 | 17.6 |  | 550 | 1.1 |
| Kansas | 2010 | 285 | 85.3 |  | 3,020 | 9.0 |  | 3,597 | 36.8 |  | 1,399 | 18.8 |  | 496 | 1.8 |
| Kansas | 2011 | 254 | 86.2 |  | 2,489 | 8.6 |  | 3,612 | 35.7 |  | 1,495 | 20.5 |  | 529 | 1.7 |
| Kansas | 2012 | 262 | 84.7 |  | 2,137 | 8.3 |  | 3,931 | 38.1 |  | 1,523 | 23.0 |  | 488 | 2.0 |
| Kansas | 2013 | 266 | 86.8 |  | 1,370 | 8.5 |  | 3,758 | 38.2 |  | 1,399 | 23.9 |  | 436 | 2.1 |
| Kansas | 2014 | 149 | 84.6 |  | 714 | 7.0 |  | 2,513 | 38.2 |  | 800 | 22.4 |  | 276 | 1.4 |
| Kansas | 2015 | 164 | 86.6 |  | 648 | 7.3 |  | 2,581 | 34.5 |  | 802 | 21.4 |  | 265 | 0.8 |
| Kansas | 2016 | 450 | 87.6 |  | 1,279 | 6.8 |  | 6,266 | 38.7 |  | 1,583 | 22.5 |  | 478 | 1.7 |
| Kansas | 2017 | 496 | 85.5 |  | 1,272 | 7.2 |  | 6,488 | 37.3 |  | 1,476 | 23.0 |  | 451 | 0.9 |
| Kansas | 2018 | 455 | 83.7 |  | 920 | 6.5 |  | 5,316 | 36.2 |  | 1,082 | 23.3 |  | 357 | 1.4 |
| Kansas | 2019 | 308 | 87.0 |  | 580 | 5.5 |  | 4,028 | 38.1 |  | 609 | 28.9 |  | 174 | 2.3 |
| Kansas | 2020 | 363 | 84.8 |  | 632 | 3.8 |  | 4,605 | 36.0 |  | 636 | 20.4 |  | 235 | 1.7 |
| Kentucky | 2000 | 192 | 72.4 |  | 1,829 | 9.6 |  | 492 | 12.8 |  | 738 | 14.9 |  | 781 | 0.5 |
| Kentucky | 2001 | 279 | 69.5 |  | 3,179 | 6.9 |  | 834 | 10.9 |  | 1,432 | 12.1 |  | 1,428 | 0.6 |
| Kentucky | 2002 | 385 | 69.4 |  | 4,189 | 5.9 |  | 970 | 10.2 |  | 1,817 | 11.3 |  | 1,546 | 0.8 |
| Kentucky | 2003 | 482 | 69.7 |  | 5,736 | 4.4 |  | 1,327 | 9.5 |  | 2,804 | 10.2 |  | 2,053 | 0.4 |
| Kentucky | 2004 | 318 | 71.1 |  | 3,447 | 6.2 |  | 1,059 | 10.0 |  | 2,202 | 10.1 |  | 1,536 | 0.5 |
| Kentucky | 2005 | 697 | 67.1 |  | 6,903 | 4.7 |  | 2,202 | 10.9 |  | 3,592 | 10.3 |  | 2,175 | 1.0 |
| Kentucky | 2006 | 809 | 64.2 |  | 7,807 | 4.2 |  | 2,323 | 9.9 |  | 4,740 | 9.8 |  | 2,735 | 0.7 |
| Kentucky | 2007 | 825 | 64.4 |  | 7,387 | 4.4 |  | 2,019 | 9.8 |  | 5,543 | 9.7 |  | 2,714 | 0.7 |
| Kentucky | 2008 | 1,061 | 64.5 |  | 5,952 | 5.1 |  | 1,605 | 9.7 |  | 6,426 | 11.4 |  | 2,929 | 0.5 |
| Kentucky | 2009 | 1,324 | 69.0 |  | 4,566 | 7.2 |  | 1,590 | 12.2 |  | 7,129 | 13.9 |  | 3,204 | 1.1 |
| Kentucky | 2010 | 1,525 | 71.5 |  | 4,017 | 8.1 |  | 1,988 | 14.4 |  | 8,611 | 14.9 |  | 3,658 | 1.9 |
| Kentucky | 2011 | 1,650 | 76.9 |  | 3,414 | 8.1 |  | 2,036 | 16.6 |  | 8,834 | 16.1 |  | 3,401 | 1.5 |
| Kentucky | 2012 | 2,524 | 77.3 |  | 2,486 | 7.8 |  | 1,725 | 20.3 |  | 6,720 | 17.0 |  | 2,402 | 1.8 |
| Kentucky | 2013 | 4,114 | 78.9 |  | 2,674 | 7.5 |  | 2,109 | 22.6 |  | 6,138 | 18.8 |  | 2,294 | 2.1 |
| Kentucky | 2014 | 5,039 | 79.0 |  | 2,650 | 10.0 |  | 2,488 | 26.6 |  | 6,343 | 17.7 |  | 2,413 | 2.6 |
| Kentucky | 2015 | 5,660 | 78.0 |  | 2,751 | 11.1 |  | 3,589 | 35.5 |  | 6,131 | 19.4 |  | 2,451 | 2.5 |
| Kentucky | 2016 | 5,880 | 75.6 |  | 2,782 | 9.2 |  | 4,870 | 35.7 |  | 6,106 | 17.7 |  | 2,364 | 1.5 |
| Kentucky | 2017 | 5,983 | 72.2 |  | 2,781 | 10.4 |  | 6,729 | 38.9 |  | 6,091 | 16.4 |  | 2,234 | 1.9 |
| Kentucky | 2018 | 5,542 | 69.6 |  | 2,764 | 8.8 |  | 8,063 | 38.3 |  | 5,365 | 16.3 |  | 1,854 | 2.0 |
| Kentucky | 2019 | 5,038 | 65.9 |  | 2,675 | 9.0 |  | 9,753 | 35.2 |  | 5,080 | 16.4 |  | 1,437 | 2.4 |
| Kentucky | 2020 | 4,409 | 64.2 |  | 2,103 | 6.1 |  | 8,273 | 34.7 |  | 4,015 | 14.4 |  | 1,131 | 1.8 |
| Louisiana | 2000 | 803 | 62.8 |  | 13,073 | 5.1 |  | 864 | 34.5 |  | 1,633 | 14.3 |  | 1,473 | 2.0 |
| Louisiana | 2001 | 922 | 57.3 |  | 12,313 | 5.1 |  | 1,069 | 30.4 |  | 2,344 | 15.9 |  | 1,586 | 2.5 |
| Louisiana | 2002 | 927 | 64.8 |  | 12,564 | 7.2 |  | 1,474 | 28.1 |  | 2,985 | 16.3 |  | 1,871 | 2.2 |
| Louisiana | 2003 | 697 | 69.0 |  | 11,661 | 7.3 |  | 1,643 | 28.6 |  | 3,447 | 16.8 |  | 1,879 | 1.8 |
| Louisiana | 2004 | 625 | 74.2 |  | 11,795 | 8.0 |  | 2,164 | 26.7 |  | 4,223 | 13.6 |  | 2,526 | 1.6 |
| Louisiana | 2005 | 403 | 72.5 |  | 10,496 | 8.4 |  | 2,373 | 25.8 |  | 4,055 | 12.2 |  | 2,239 | 1.3 |
| Louisiana | 2006 | 413 | 75.5 |  | 9,527 | 9.3 |  | 1,867 | 22.7 |  | 3,720 | 9.9 |  | 2,092 | 0.6 |
| Louisiana | 2007 | 600 | 75.5 |  | 10,205 | 9.2 |  | 1,943 | 24.4 |  | 4,746 | 8.3 |  | 2,861 | 0.6 |
| Louisiana | 2008 | 879 | 77.8 |  | 9,453 | 7.5 |  | 1,553 | 26.4 |  | 5,130 | 8.2 |  | 2,876 | 0.8 |
| Louisiana | 2009 | 1,866 | 81.5 |  | 9,122 | 8.2 |  | 1,596 | 27.0 |  | 5,843 | 9.9 |  | 3,137 | 0.4 |
| Louisiana | 2010 | 2,220 | 85.5 |  | 7,084 | 10.4 |  | 1,679 | 27.8 |  | 6,038 | 12.3 |  | 3,416 | 0.8 |
| Louisiana | 2011 | 2,668 | 85.5 |  | 6,812 | 11.4 |  | 1,985 | 33.6 |  | 6,865 | 19.3 |  | 3,388 | 1.6 |
| Louisiana | 2012 | 1,824 | 86.1 |  | 4,172 | 15.3 |  | 1,503 | 35.5 |  | 4,838 | 19.7 |  | 2,465 | 1.2 |
| Louisiana | 2013 | 2,345 | 85.8 |  | 2,950 | 18.0 |  | 2,136 | 30.2 |  | 3,786 | 21.2 |  | 2,001 | 2.0 |
| Louisiana | 2014 | 2,012 | 82.9 |  | 1,982 | 21.7 |  | 1,872 | 32.3 |  | 2,340 | 20.3 |  | 1,370 | 2.7 |
| Louisiana | 2015 | 1,236 | 82.6 |  | 1,544 | 15.2 |  | 2,225 | 33.7 |  | 1,834 | 18.4 |  | 936 | 3.5 |
| Louisiana | 2016 | 1,272 | 83.6 |  | 1,304 | 19.5 |  | 1,587 | 41.5 |  | 1,375 | 15.1 |  | 821 | 2.7 |
| Louisiana | 2017 | 1,412 | 79.2 |  | 1,940 | 13.7 |  | 2,464 | 38.7 |  | 1,596 | 14.2 |  | 902 | 1.9 |
| Louisiana | 2018 | 1,827 | 75.3 |  | 2,098 | 13.3 |  | 3,150 | 40.1 |  | 1,726 | 12.5 |  | 841 | 1.9 |
| Louisiana | 2019 | 1,805 | 73.7 |  | 1,843 | 13.3 |  | 3,421 | 36.7 |  | 1,419 | 8.9 |  | 826 | 1.0 |
| Louisiana | 2020 | 1,361 | 64.4 |  | 1,396 | 7.4 |  | 3,261 | 30.7 |  | 1,084 | 7.6 |  | 635 | 1.1 |
| Maine | 2000 | 797 | 70.1 |  | 1,104 | 15.4 |  | 158 | 8.9 |  | 1,294 | 27.4 |  | 418 | 1.7 |
| Maine | 2001 | 1,140 | 68.6 |  | 1,086 | 15.7 |  | 167 | 9.6 |  | 1,767 | 27.2 |  | 405 | 2.2 |
| Maine | 2002 | 1,599 | 67.1 |  | 1,337 | 16.1 |  | 144 | 12.5 |  | 2,117 | 25.2 |  | 413 | 2.2 |
| Maine | 2003 | 1,659 | 69.6 |  | 1,821 | 18.3 |  | 188 | 9.6 |  | 2,649 | 25.6 |  | 659 | 2.0 |
| Maine | 2004 | 1,921 | 70.0 |  | 1,930 | 18.0 |  | 214 | 14.0 |  | 3,149 | 26.4 |  | 713 | 2.0 |
| Maine | 2005 | 2,136 | 68.7 |  | 2,180 | 20.7 |  | 242 | 12.0 |  | 3,930 | 26.6 |  | 785 | 2.0 |
| Maine | 2006 | 2,255 | 66.8 |  | 2,684 | 20.4 |  | 328 | 11.0 |  | 4,719 | 22.5 |  | 902 | 1.8 |
| Maine | 2007 | 2,242 | 64.2 |  | 3,067 | 20.8 |  | 362 | 12.7 |  | 5,706 | 22.2 |  | 1,078 | 2.6 |
| Maine | 2008 | 2,434 | 67.5 |  | 2,718 | 24.0 |  | 349 | 16.6 |  | 6,565 | 22.5 |  | 933 | 1.9 |
| Maine | 2009 | 2,445 | 65.4 |  | 2,138 | 21.3 |  | 342 | 12.0 |  | 6,385 | 22.4 |  | 1,005 | 2.6 |
| Maine | 2010 | 1,812 | 68.2 |  | 1,689 | 23.4 |  | 340 | 17.9 |  | 5,932 | 23.2 |  | 924 | 3.9 |
| Maine | 2011 | 2,240 | 69.7 |  | 2,041 | 26.3 |  | 472 | 23.7 |  | 7,188 | 25.4 |  | 1,244 | 2.5 |
| Maine | 2012 | 2,790 | 69.8 |  | 2,141 | 25.3 |  | 543 | 28.2 |  | 7,495 | 26.4 |  | 1,148 | 4.6 |
| Maine | 2013 | 3,979 | 71.4 |  | 2,323 | 25.4 |  | 522 | 24.3 |  | 7,506 | 26.4 |  | 1,127 | 4.6 |
| Maine | 2014 | 5,090 | 70.8 |  | 2,232 | 21.9 |  | 543 | 27.6 |  | 6,365 | 25.1 |  | 1,047 | 3.3 |
| Maine | 2015 | 4,974 | 68.6 |  | 1,785 | 23.1 |  | 483 | 28.6 |  | 4,963 | 24.3 |  | 980 | 3.2 |
| Maine | 2016 | 4,128 | 65.5 |  | 1,689 | 21.8 |  | 475 | 28.8 |  | 3,891 | 22.1 |  | 749 | 3.1 |
| Maine | 2017 | 3,657 | 63.2 |  | 1,798 | 22.5 |  | 455 | 28.4 |  | 3,086 | 20.4 |  | 647 | 2.6 |
| Maine | 2018 | 3,713 | 60.3 |  | 1,941 | 20.2 |  | 547 | 38.0 |  | 2,625 | 20.1 |  | 584 | 2.4 |
| Maine | 2019 | 3,598 | 59.3 |  | 1,905 | 19.1 |  | 858 | 44.6 |  | 2,241 | 17.6 |  | 538 | 1.7 |
| Maine | 2020 | 3,045 | 57.1 |  | 1,529 | 16.8 |  | 821 | 41.9 |  | 1,530 | 19.8 |  | 327 | 1.8 |
| Maryland | 2000 | 17,945 | 47.0 |  | 19,223 | 19.9 |  | 192 | 9.4 |  | 1,656 | 9.2 |  | 889 | 1.3 |
| Maryland | 2001 | 21,309 | 45.9 |  | 22,746 | 19.2 |  | 310 | 5.5 |  | 2,757 | 7.0 |  | 1,304 | 0.9 |
| Maryland | 2002 | 24,206 | 46.7 |  | 26,796 | 20.2 |  | 434 | 7.1 |  | 3,554 | 5.8 |  | 1,752 | 1.1 |
| Maryland | 2003 | 25,681 | 48.3 |  | 27,977 | 20.6 |  | 492 | 11.6 |  | 3,997 | 6.7 |  | 1,889 | 1.0 |
| Maryland | 2004 | 26,098 | 48.6 |  | 29,448 | 18.0 |  | 901 | 6.0 |  | 5,019 | 6.1 |  | 2,075 | 1.3 |
| Maryland | 2005 | 25,914 | 49.8 |  | 29,327 | 17.4 |  | 676 | 6.8 |  | 5,926 | 7.3 |  | 2,343 | 0.6 |
| Maryland | 2006 | 21,394 | 50.5 |  | 26,994 | 14.9 |  | 711 | 4.5 |  | 6,760 | 6.8 |  | 2,338 | 0.9 |
| Maryland | 2007 | 20,781 | 52.1 |  | 26,375 | 14.1 |  | 733 | 6.3 |  | 7,604 | 7.1 |  | 2,544 | 1.1 |
| Maryland | 2008 | 21,129 | 51.8 |  | 23,495 | 14.0 |  | 596 | 5.5 |  | 8,230 | 7.6 |  | 2,441 | 1.4 |
| Maryland | 2009 | 20,304 | 55.8 |  | 20,101 | 14.2 |  | 595 | 4.9 |  | 9,524 | 9.6 |  | 2,747 | 1.3 |
| Maryland | 2010 | 15,650 | 58.9 |  | 14,842 | 17.3 |  | 520 | 4.2 |  | 8,681 | 12.2 |  | 2,906 | 1.2 |
| Maryland | 2011 | 20,255 | 60.5 |  | 19,242 | 15.8 |  | 646 | 6.7 |  | 13,315 | 13.7 |  | 4,255 | 2.0 |
| Maryland | 2012 | 20,219 | 60.6 |  | 17,315 | 16.0 |  | 626 | 9.7 |  | 13,481 | 13.6 |  | 4,403 | 1.3 |
| Maryland | 2013 | 19,239 | 61.9 |  | 12,411 | 15.4 |  | 680 | 8.8 |  | 10,095 | 11.7 |  | 3,463 | 1.3 |
| Maryland | 2014 | 16,552 | 61.1 |  | 10,061 | 15.6 |  | 515 | 10.1 |  | 6,820 | 9.0 |  | 2,899 | 0.9 |
| Maryland | 2015 | 60,531 | 56.6 |  | 18,765 | 14.7 |  | 1,042 | 13.2 |  | 18,417 | 6.3 |  | 5,845 | 1.0 |
| Maryland | 2016 | 46,630 | 55.5 |  | 16,472 | 15.7 |  | 901 | 17.4 |  | 14,049 | 6.2 |  | 4,501 | 0.9 |
| Maryland | 2017 | 85,855 | 50.8 |  | 30,734 | 15.1 |  | 1,709 | 18.3 |  | 24,984 | 5.5 |  | 7,161 | 1.1 |
| Maryland | 2018 | 92,509 | 47.8 |  | 39,914 | 12.7 |  | 2,617 | 24.0 |  | 26,073 | 6.1 |  | 7,692 | 1.1 |
| Maryland | 2019 | 61,539 | 42.7 |  | 31,866 | 10.8 |  | 2,708 | 29.5 |  | 15,660 | 5.7 |  | 5,577 | 1.1 |
| Maryland | 2020 | Data not reported | |  | 0 |  |  | 0 |  |  | 0 |  |  | 0 |  |
| Massachusetts | 2000 | 33,009 | 69.1 |  | 21,846 | 17.2 |  | 368 | 7.6 |  | 4,000 | 3.0 |  | 4,830 | 0.6 |
| Massachusetts | 2001 | 40,803 | 68.9 |  | 22,764 | 20.7 |  | 353 | 6.5 |  | 7,347 | 3.0 |  | 5,757 | 0.7 |
| Massachusetts | 2002 | 39,183 | 70.7 |  | 20,858 | 23.1 |  | 281 | 7.8 |  | 8,437 | 3.5 |  | 5,460 | 0.9 |
| Massachusetts | 2003 | 27,717 | 71.5 |  | 15,916 | 20.3 |  | 308 | 7.1 |  | 6,856 | 3.2 |  | 4,398 | 0.6 |
| Massachusetts | 2004 | 28,234 | 75.1 |  | 18,033 | 20.0 |  | 380 | 5.3 |  | 7,360 | 3.7 |  | 4,561 | 0.8 |
| Massachusetts | 2005 | 42,650 | 76.0 |  | 32,433 | 18.4 |  | 722 | 7.1 |  | 12,686 | 3.9 |  | 7,313 | 0.8 |
| Massachusetts | 2006 | 41,063 | 76.9 |  | 33,487 | 19.4 |  | 689 | 11.3 |  | 12,971 | 3.8 |  | 7,689 | 0.9 |
| Massachusetts | 2007 | 41,701 | 78.5 |  | 31,873 | 20.1 |  | 619 | 10.5 |  | 13,540 | 5.6 |  | 8,071 | 1.3 |
| Massachusetts | 2008 | 39,173 | 79.5 |  | 24,459 | 22.3 |  | 395 | 12.2 |  | 13,090 | 7.6 |  | 7,701 | 1.4 |
| Massachusetts | 2009 | 42,346 | 80.5 |  | 21,574 | 24.8 |  | 390 | 14.4 |  | 14,861 | 10.5 |  | 8,708 | 1.9 |
| Massachusetts | 2010 | 41,014 | 82.2 |  | 19,643 | 28.8 |  | 344 | 11.0 |  | 16,982 | 14.6 |  | 9,478 | 2.0 |
| Massachusetts | 2011 | 42,546 | 82.4 |  | 18,601 | 32.5 |  | 368 | 17.7 |  | 17,230 | 15.7 |  | 10,743 | 2.1 |
| Massachusetts | 2012 | 47,017 | 82.1 |  | 18,573 | 34.7 |  | 487 | 23.0 |  | 15,522 | 14.4 |  | 10,461 | 2.1 |
| Massachusetts | 2013 | 52,024 | 82.0 |  | 18,209 | 36.4 |  | 585 | 26.3 |  | 12,984 | 12.3 |  | 10,449 | 1.7 |
| Massachusetts | 2014 | 53,461 | 83.6 |  | 16,876 | 39.3 |  | 567 | 28.6 |  | 10,135 | 9.9 |  | 11,034 | 1.8 |
| Massachusetts | 2015 | 50,550 | 81.9 |  | 14,917 | 38.1 |  | 634 | 29.8 |  | 8,124 | 7.0 |  | 8,568 | 1.6 |
| Massachusetts | 2016 | 50,399 | 79.6 |  | 17,151 | 40.0 |  | 914 | 33.2 |  | 7,953 | 7.6 |  | 8,751 | 1.4 |
| Massachusetts | 2017 | 49,797 | 76.6 |  | 20,690 | 37.3 |  | 1,044 | 43.8 |  | 7,413 | 9.5 |  | 9,558 | 1.3 |
| Massachusetts | 2018 | 47,867 | 73.4 |  | 21,046 | 34.1 |  | 1,426 | 54.1 |  | 6,718 | 9.6 |  | 9,678 | 1.6 |
| Massachusetts | 2019 | 46,900 | 69.8 |  | 22,243 | 29.5 |  | 2,040 | 54.8 |  | 6,776 | 12.6 |  | 9,965 | 1.5 |
| Massachusetts | 2020 | 37,780 | 68.8 |  | 17,004 | 28.7 |  | 1,976 | 56.2 |  | 5,685 | 21.0 |  | 8,275 | 1.4 |
| Michigan | 2000 | 7,862 | 59.3 |  | 17,396 | 2.0 |  | 537 | 7.4 |  | 2,630 | 9.2 |  | 710 | 0.6 |
| Michigan | 2001 | 8,082 | 61.0 |  | 16,056 | 2.5 |  | 669 | 11.1 |  | 3,324 | 9.7 |  | 799 | 0.5 |
| Michigan | 2002 | 9,106 | 62.6 |  | 20,288 | 2.0 |  | 1,069 | 6.8 |  | 4,390 | 8.9 |  | 1,249 | 0.2 |
| Michigan | 2003 | 9,028 | 62.5 |  | 20,575 | 2.1 |  | 1,262 | 7.3 |  | 4,609 | 9.4 |  | 1,345 | 0.3 |
| Michigan | 2004 | 9,011 | 63.3 |  | 20,613 | 2.4 |  | 1,503 | 7.1 |  | 5,399 | 10.6 |  | 1,403 | 0.1 |
| Michigan | 2005 | 8,692 | 65.2 |  | 18,635 | 2.7 |  | 1,479 | 7.6 |  | 5,624 | 11.3 |  | 1,485 | 0.1 |
| Michigan | 2006 | 10,744 | 64.0 |  | 22,289 | 2.4 |  | 1,304 | 7.0 |  | 7,687 | 11.6 |  | 1,948 | 0.1 |
| Michigan | 2007 | 10,695 | 65.6 |  | 21,825 | 2.2 |  | 1,013 | 8.0 |  | 8,641 | 12.0 |  | 2,444 | 0.1 |
| Michigan | 2008 | 11,900 | 67.3 |  | 18,221 | 2.4 |  | 1,254 | 8.9 |  | 10,139 | 12.6 |  | 2,859 | 0.3 |
| Michigan | 2009 | 12,653 | 69.3 |  | 15,110 | 2.5 |  | 1,426 | 14.0 |  | 11,457 | 14.3 |  | 3,652 | 0.2 |
| Michigan | 2010 | 12,210 | 71.9 |  | 13,128 | 2.8 |  | 1,597 | 13.3 |  | 12,226 | 15.4 |  | 4,193 | 0.2 |
| Michigan | 2011 | 12,781 | 72.6 |  | 12,752 | 3.1 |  | 1,778 | 15.5 |  | 12,866 | 15.2 |  | 4,362 | 0.2 |
| Michigan | 2012 | 13,370 | 72.6 |  | 11,188 | 2.8 |  | 1,800 | 19.6 |  | 12,564 | 14.9 |  | 5,137 | 0.3 |
| Michigan | 2013 | 13,620 | 75.1 |  | 10,873 | 2.8 |  | 1,787 | 18.6 |  | 12,345 | 15.0 |  | 5,161 | 0.4 |
| Michigan | 2014 | 17,969 | 71.4 |  | 12,221 | 3.6 |  | 2,697 | 21.4 |  | 14,296 | 14.9 |  | 5,680 | 0.4 |
| Michigan | 2015 | 21,931 | 73.5 |  | 13,011 | 5.8 |  | 2,765 | 24.8 |  | 14,925 | 14.4 |  | 6,639 | 0.9 |
| Michigan | 2016 | 27,197 | 72.9 |  | 18,276 | 8.1 |  | 4,333 | 27.4 |  | 15,857 | 14.7 |  | 7,740 | 0.6 |
| Michigan | 2017 | 27,855 | 71.0 |  | 21,252 | 9.9 |  | 5,639 | 31.6 |  | 16,136 | 14.3 |  | 7,343 | 1.0 |
| Michigan | 2018 | 26,274 | 68.0 |  | 22,544 | 9.7 |  | 7,764 | 36.3 |  | 13,860 | 13.5 |  | 6,092 | 0.5 |
| Michigan | 2019 | 25,637 | 65.8 |  | 22,759 | 8.0 |  | 10,374 | 39.1 |  | 12,700 | 13.6 |  | 5,016 | 0.6 |
| Michigan | 2020 | 20,379 | 64.4 |  | 17,503 | 7.4 |  | 10,662 | 38.0 |  | 9,371 | 14.6 |  | 3,437 | 0.8 |
| Minnesota | 2000 | 1,105 | 57.4 |  | 5,184 | 12.5 |  | 2,420 | 20.6 |  | 900 | 16.4 |  | 740 | 1.2 |
| Minnesota | 2001 | 1,090 | 56.7 |  | 5,195 | 12.5 |  | 3,305 | 20.2 |  | 1,017 | 15.0 |  | 694 | 1.7 |
| Minnesota | 2002 | 1,022 | 59.0 |  | 5,052 | 11.2 |  | 3,909 | 18.7 |  | 1,101 | 15.6 |  | 637 | 1.3 |
| Minnesota | 2003 | 1,117 | 60.8 |  | 5,614 | 10.3 |  | 4,862 | 17.7 |  | 1,551 | 14.0 |  | 807 | 1.9 |
| Minnesota | 2004 | 1,291 | 64.2 |  | 6,105 | 10.5 |  | 6,435 | 14.8 |  | 1,924 | 13.6 |  | 928 | 2.3 |
| Minnesota | 2005 | 1,549 | 62.2 |  | 6,350 | 9.8 |  | 7,353 | 13.8 |  | 2,235 | 12.6 |  | 1,007 | 1.1 |
| Minnesota | 2006 | 1,874 | 62.1 |  | 10,486 | 1.7 |  | 6,550 | 14.8 |  | 2,565 | 11.5 |  | 1,338 | 0.1 |
| Minnesota | 2007 | 2,249 | 63.0 |  | 10,062 | 5.5 |  | 7,765 | 13.9 |  | 4,073 | 11.4 |  | 1,331 | 0.8 |
| Minnesota | 2008 | 2,415 | 64.1 |  | 9,004 | 5.5 |  | 6,460 | 15.2 |  | 4,824 | 11.0 |  | 1,599 | 1.1 |
| Minnesota | 2009 | 2,792 | 63.8 |  | 7,088 | 5.3 |  | 6,532 | 14.8 |  | 6,135 | 11.8 |  | 1,825 | 1.5 |
| Minnesota | 2010 | 2,934 | 64.6 |  | 6,053 | 6.4 |  | 7,079 | 18.1 |  | 7,091 | 14.2 |  | 2,111 | 1.1 |
| Minnesota | 2011 | 4,161 | 64.9 |  | 5,962 | 7.9 |  | 7,763 | 18.7 |  | 7,899 | 14.8 |  | 2,298 | 1.5 |
| Minnesota | 2012 | 5,426 | 62.8 |  | 5,610 | 6.8 |  | 9,154 | 21.4 |  | 8,140 | 15.1 |  | 2,395 | 2.0 |
| Minnesota | 2013 | 6,247 | 65.5 |  | 4,857 | 5.9 |  | 11,626 | 23.9 |  | 8,028 | 14.1 |  | 2,290 | 1.9 |
| Minnesota | 2014 | 6,816 | 64.3 |  | 4,613 | 5.5 |  | 13,796 | 25.9 |  | 7,505 | 13.8 |  | 2,354 | 2.1 |
| Minnesota | 2015 | 8,377 | 65.5 |  | 4,635 | 6.0 |  | 16,246 | 28.2 |  | 7,585 | 13.7 |  | 2,590 | 1.5 |
| Minnesota | 2016 | 9,501 | 65.6 |  | 5,079 | 6.0 |  | 19,271 | 30.1 |  | 7,064 | 12.5 |  | 3,047 | 2.3 |
| Minnesota | 2017 | 10,985 | 64.9 |  | 6,307 | 6.7 |  | 22,577 | 31.8 |  | 6,777 | 12.3 |  | 3,094 | 1.6 |
| Minnesota | 2018 | 10,061 | 64.0 |  | 6,412 | 6.0 |  | 24,464 | 31.6 |  | 5,828 | 10.4 |  | 2,813 | 1.3 |
| Minnesota | 2019 | 10,302 | 61.6 |  | 6,024 | 4.3 |  | 26,221 | 31.8 |  | 5,289 | 12.5 |  | 2,551 | 1.5 |
| Minnesota | 2020 | 8,763 | 59.4 |  | 4,128 | 3.9 |  | 21,097 | 32.0 |  | 4,442 | 11.0 |  | 1,900 | 1.6 |
| Mississippi | 2000 | 133 | 73.7 |  | 3,343 | 3.2 |  | 568 | 16.2 |  | 471 | 23.4 |  | 336 | 3.6 |
| Mississippi | 2001 | 134 | 73.9 |  | 3,518 | 3.8 |  | 882 | 17.6 |  | 746 | 27.7 |  | 451 | 3.1 |
| Mississippi | 2002 | 200 | 62.0 |  | 3,928 | 4.6 |  | 1,049 | 19.6 |  | 946 | 29.0 |  | 570 | 2.6 |
| Mississippi | 2003 | 175 | 70.9 |  | 3,682 | 5.4 |  | 1,086 | 19.8 |  | 945 | 23.5 |  | 473 | 3.2 |
| Mississippi | 2004 | 130 | 64.6 |  | 3,392 | 6.1 |  | 950 | 20.1 |  | 1,022 | 21.6 |  | 451 | 2.2 |
| Mississippi | 2005 | 95 | 72.6 |  | 3,018 | 5.0 |  | 935 | 15.4 |  | 979 | 19.3 |  | 464 | 1.7 |
| Mississippi | 2006 | 97 | 64.9 |  | 2,795 | 5.3 |  | 780 | 15.5 |  | 942 | 18.8 |  | 434 | 1.4 |
| Mississippi | 2007 | 120 | 64.2 |  | 2,538 | 5.1 |  | 745 | 16.2 |  | 922 | 16.3 |  | 463 | 1.5 |
| Mississippi | 2008 | 39 | 74.4 |  | 1,347 | 4.9 |  | 355 | 14.1 |  | 466 | 12.7 |  | 197 | 1.0 |
| Mississippi | 2009 | Data not reported | |  |  |  |  |  |  |  |  |  |  |  |  |
| Mississippi | 2010 | 78 | 75.6 |  | 830 | 6.1 |  | 454 | 16.3 |  | 774 | 16.3 |  | 354 | 1.4 |
| Mississippi | 2011 | 143 | 76.9 |  | 1,717 | 5.3 |  | 874 | 19.7 |  | 1,542 | 15.9 |  | 745 | 2.4 |
| Mississippi | 2012 | 162 | 85.8 |  | 1,546 | 6.7 |  | 798 | 23.1 |  | 1,456 | 17.0 |  | 677 | 1.9 |
| Mississippi | 2013 | 160 | 78.8 |  | 1,150 | 5.7 |  | 838 | 26.0 |  | 1,236 | 18.0 |  | 552 | 1.4 |
| Mississippi | 2014 | 231 | 71.4 |  | 979 | 3.7 |  | 833 | 21.1 |  | 867 | 17.6 |  | 434 | 4.4 |
| Mississippi | 2015 | 338 | 67.8 |  | 1,427 | 4.1 |  | 1,571 | 24.9 |  | 1,338 | 16.0 |  | 606 | 1.5 |
| Mississippi | 2016 | 452 | 72.6 |  | 1,426 | 5.0 |  | 2,085 | 30.0 |  | 1,249 | 17.3 |  | 636 | 1.6 |
| Mississippi | 2017 | 760 | 75.9 |  | 1,423 | 5.8 |  | 2,707 | 33.8 |  | 1,813 | 15.9 |  | 838 | 1.6 |
| Mississippi | 2018 | 774 | 73.6 |  | 1,211 | 4.5 |  | 2,594 | 32.6 |  | 1,765 | 17.1 |  | 682 | 1.5 |
| Mississippi | 2019 | 802 | 69.3 |  | 1,218 | 4.1 |  | 2,716 | 30.3 |  | 1,285 | 12.3 |  | 569 | 0.0 |
| Mississippi | 2020 | 741 | 66.7 |  | 1,111 | 3.5 |  | 2,406 | 29.1 |  | 1,251 | 16.1 |  | 480 | 1.0 |
| Missouri | 2000 | 2,696 | 55.5 |  | 12,748 | 6.4 |  | 6,553 | 35.9 |  | 432 | 19.0 |  | 840 | 2.4 |
| Missouri | 2001 | 2,492 | 55.7 |  | 13,259 | 6.0 |  | 7,041 | 36.3 |  | 755 | 22.1 |  | 929 | 3.3 |
| Missouri | 2002 | 2,085 | 56.2 |  | 12,035 | 5.6 |  | 6,966 | 35.6 |  | 1,149 | 23.3 |  | 921 | 3.1 |
| Missouri | 2003 | 1,898 | 55.1 |  | 11,220 | 5.6 |  | 6,505 | 35.0 |  | 1,235 | 19.8 |  | 964 | 3.3 |
| Missouri | 2004 | 2,175 | 54.2 |  | 11,794 | 5.6 |  | 7,812 | 33.2 |  | 1,720 | 22.5 |  | 1,066 | 3.0 |
| Missouri | 2005 | 2,667 | 57.6 |  | 12,348 | 4.9 |  | 9,134 | 32.1 |  | 2,037 | 22.6 |  | 1,176 | 2.6 |
| Missouri | 2006 | 2,613 | 53.7 |  | 12,603 | 5.6 |  | 8,440 | 33.2 |  | 2,133 | 21.8 |  | 1,310 | 3.4 |
| Missouri | 2007 | 2,817 | 55.0 |  | 11,015 | 5.7 |  | 7,524 | 33.8 |  | 2,267 | 19.3 |  | 1,349 | 1.7 |
| Missouri | 2008 | 3,917 | 58.3 |  | 11,678 | 5.9 |  | 8,558 | 36.2 |  | 3,793 | 20.5 |  | 2,003 | 1.5 |
| Missouri | 2009 | 4,844 | 58.5 |  | 10,030 | 6.3 |  | 9,548 | 38.0 |  | 4,966 | 24.3 |  | 2,388 | 2.1 |
| Missouri | 2010 | 4,599 | 62.9 |  | 7,110 | 6.4 |  | 8,262 | 38.2 |  | 4,625 | 24.6 |  | 2,270 | 1.9 |
| Missouri | 2011 | 3,967 | 64.9 |  | 5,236 | 6.7 |  | 7,030 | 37.6 |  | 3,937 | 22.9 |  | 1,797 | 1.7 |
| Missouri | 2012 | 4,709 | 69.3 |  | 4,961 | 6.6 |  | 8,959 | 42.9 |  | 4,710 | 23.5 |  | 2,122 | 2.4 |
| Missouri | 2013 | 5,064 | 70.7 |  | 4,514 | 7.2 |  | 10,097 | 41.6 |  | 4,984 | 24.4 |  | 2,014 | 1.8 |
| Missouri | 2014 | 5,702 | 69.7 |  | 4,269 | 7.4 |  | 11,105 | 41.8 |  | 4,893 | 23.8 |  | 1,898 | 1.3 |
| Missouri | 2015 | 6,925 | 70.2 |  | 4,085 | 7.7 |  | 11,922 | 41.6 |  | 5,032 | 22.2 |  | 2,102 | 1.9 |
| Missouri | 2016 | 6,911 | 72.9 |  | 3,911 | 7.9 |  | 13,366 | 41.3 |  | 4,924 | 21.5 |  | 2,091 | 1.3 |
| Missouri | 2017 | 6,879 | 68.6 |  | 3,658 | 7.8 |  | 13,999 | 39.9 |  | 4,625 | 22.3 |  | 2,023 | 2.4 |
| Missouri | 2018 | 8,765 | 67.8 |  | 3,832 | 8.5 |  | 16,077 | 39.8 |  | 6,017 | 27.8 |  | 2,011 | 1.9 |
| Missouri | 2019 | 6,833 | 63.6 |  | 3,374 | 6.8 |  | 15,183 | 39.3 |  | 5,634 | 31.9 |  | 1,623 | 2.0 |
| Missouri | 2020 | 5,192 | 61.3 |  | 2,457 | 6.2 |  | 11,622 | 38.7 |  | 5,207 | 31.4 |  | 1,212 | 1.0 |
| Montana | 2000 | 136 | 88.2 |  | 680 | 30.6 |  | 1,576 | 42.1 |  | 327 | 23.5 |  | 98 | 4.1 |
| Montana | 2001 | 146 | 85.6 |  | 739 | 34.1 |  | 1,872 | 42.6 |  | 416 | 24.5 |  | 123 | 5.7 |
| Montana | 2002 | 123 | 84.6 |  | 691 | 29.5 |  | 1,844 | 40.7 |  | 451 | 22.4 |  | 160 | 5.0 |
| Montana | 2003 | 139 | 87.8 |  | 714 | 25.5 |  | 2,256 | 40.8 |  | 626 | 27.5 |  | 169 | 2.4 |
| Montana | 2004 | 140 | 79.3 |  | 640 | 26.3 |  | 2,160 | 38.9 |  | 628 | 25.6 |  | 180 | 3.3 |
| Montana | 2005 | 152 | 84.2 |  | 733 | 25.5 |  | 2,602 | 38.1 |  | 705 | 27.5 |  | 204 | 6.4 |
| Montana | 2006 | 174 | 84.5 |  | 854 | 21.7 |  | 2,460 | 35.0 |  | 882 | 23.9 |  | 191 | 1.6 |
| Montana | 2007 | 233 | 83.7 |  | 1,228 | 16.8 |  | 2,732 | 32.7 |  | 1,310 | 24.0 |  | 283 | 2.5 |
| Montana | 2008 | 199 | 80.4 |  | 801 | 20.2 |  | 1,780 | 35.7 |  | 1,014 | 25.7 |  | 199 | 4.0 |
| Montana | 2009 | 179 | 84.9 |  | 613 | 23.5 |  | 1,557 | 37.4 |  | 1,239 | 27.5 |  | 267 | 2.6 |
| Montana | 2010 | 190 | 84.2 |  | 619 | 24.4 |  | 1,696 | 39.1 |  | 1,398 | 29.6 |  | 231 | 3.0 |
| Montana | 2011 | 272 | 82.4 |  | 689 | 24.7 |  | 2,217 | 41.8 |  | 1,691 | 33.0 |  | 256 | 4.3 |
| Montana | 2012 | 335 | 78.8 |  | 603 | 21.7 |  | 2,298 | 45.1 |  | 1,693 | 34.8 |  | 217 | 6.5 |
| Montana | 2013 | 319 | 75.9 |  | 378 | 22.2 |  | 2,178 | 41.6 |  | 1,335 | 35.4 |  | 190 | 5.8 |
| Montana | 2014 | 213 | 78.9 |  | 242 | 14.9 |  | 1,943 | 41.6 |  | 1,022 | 32.4 |  | 160 | 3.8 |
| Montana | 2015 | 246 | 72.8 |  | 215 | 17.7 |  | 2,043 | 42.3 |  | 975 | 30.1 |  | 146 | 3.4 |
| Montana | 2016 | 251 | 72.1 |  | 177 | 18.1 |  | 2,224 | 42.3 |  | 803 | 32.0 |  | 144 | 19.4 |
| Montana | 2017 | 135 | 73.3 |  | 96 | 19.8 |  | 1,189 | 43.7 |  | 350 | 29.4 |  | 54 | 20.4 |
| Montana | 2018 | 72 | 70.8 |  | 41 | 22.0 |  | 691 | 45.3 |  | 165 | 21.8 |  | 20 | 25.0 |
| Montana | 2019 | 121 | 72.7 |  | 31 | 19.4 |  | 605 | 46.4 |  | 136 | 32.4 |  | 22 | 9.1 |
| Montana | 2020 | 136 | 61.8 |  | 35 | 17.1 |  | 585 | 51.3 |  | 107 | 19.6 |  | 19 | 0.0 |
| Nebraska | 2000 | 29 | 75.9 |  | 1,523 | 14.7 |  | 1,605 | 41.0 |  | 101 | 38.6 |  | 81 | 7.4 |
| Nebraska | 2001 | 34 | 79.4 |  | 1,570 | 15.0 |  | 2,081 | 39.8 |  | 148 | 31.8 |  | 90 | 4.4 |
| Nebraska | 2002 | 35 | 80.0 |  | 1,468 | 13.6 |  | 2,338 | 38.4 |  | 125 | 31.2 |  | 86 | 2.3 |
| Nebraska | 2003 | 21 | 76.2 |  | 1,457 | 13.2 |  | 2,644 | 33.7 |  | 156 | 32.7 |  | 94 | 2.1 |
| Nebraska | 2004 | 0 |  |  | 1,788 | 13.1 |  | 3,361 | 36.3 |  | 358 | 37.7 |  | 157 | 4.5 |
| Nebraska | 2005 | 0 |  |  | 1,386 | 13.9 |  | 2,649 | 32.7 |  | 301 | 32.9 |  | 107 | 0.9 |
| Nebraska | 2006 | 0 |  |  | 1,306 | 15.0 |  | 2,366 | 31.7 |  | 318 | 35.5 |  | 117 | 5.1 |
| Nebraska | 2007 | 20 | 50.0 |  | 1,231 | 8.0 |  | 2,063 | 28.8 |  | 318 | 31.1 |  | 110 | 11.8 |
| Nebraska | 2008 | 62 | 85.5 |  | 1,062 | 11.3 |  | 1,573 | 34.9 |  | 330 | 22.1 |  | 122 | 2.5 |
| Nebraska | 2009 | 47 | 80.9 |  | 566 | 16.6 |  | 1,211 | 36.7 |  | 276 | 18.8 |  | 80 | 3.8 |
| Nebraska | 2010 | 61 | 77.0 |  | 979 | 15.4 |  | 2,204 | 34.8 |  | 519 | 19.7 |  | 170 | 3.5 |
| Nebraska | 2011 | 141 | 87.9 |  | 1,270 | 17.2 |  | 2,777 | 36.8 |  | 1,038 | 21.8 |  | 280 | 3.6 |
| Nebraska | 2012 | 141 | 83.0 |  | 949 | 13.7 |  | 2,839 | 37.0 |  | 800 | 23.8 |  | 306 | 5.2 |
| Nebraska | 2013 | 150 | 84.7 |  | 805 | 12.4 |  | 3,349 | 38.3 |  | 803 | 24.8 |  | 282 | 3.2 |
| Nebraska | 2014 | 112 | 82.1 |  | 639 | 12.4 |  | 3,289 | 38.1 |  | 687 | 28.2 |  | 206 | 2.9 |
| Nebraska | 2015 | 209 | 84.2 |  | 626 | 12.5 |  | 3,683 | 38.3 |  | 969 | 25.8 |  | 244 | 2.0 |
| Nebraska | 2016 | 234 | 84.6 |  | 575 | 13.4 |  | 4,646 | 37.9 |  | 876 | 26.4 |  | 277 | 4.3 |
| Nebraska | 2017 | 226 | 75.2 |  | 683 | 10.8 |  | 5,061 | 35.9 |  | 869 | 25.9 |  | 271 | 3.7 |
| Nebraska | 2018 | 231 | 81.4 |  | 701 | 8.8 |  | 4,945 | 39.9 |  | 847 | 29.2 |  | 286 | 2.4 |
| Nebraska | 2019 | 289 | 85.1 |  | 728 | 8.9 |  | 5,153 | 38.2 |  | 717 | 28.2 |  | 265 | 3.0 |
| Nebraska | 2020 | 273 | 82.8 |  | 619 | 8.6 |  | 4,246 | 35.3 |  | 490 | 25.7 |  | 183 | 2.2 |
| Nevada | 2000 | 985 | 86.0 |  | 2,773 | 20.8 |  | 3,273 | 26.9 |  | 131 | 9.9 |  | 62 | 0.0 |
| Nevada | 2001 | 928 | 85.9 |  | 2,456 | 18.0 |  | 3,234 | 28.1 |  | 177 | 9.6 |  | 91 | 2.2 |
| Nevada | 2002 | 853 | 88.2 |  | 2,010 | 19.1 |  | 3,495 | 26.2 |  | 209 | 11.0 |  | 85 | 2.4 |
| Nevada | 2003 | 850 | 85.9 |  | 2,168 | 14.8 |  | 4,106 | 23.2 |  | 403 | 3.7 |  | 113 | 1.8 |
| Nevada | 2004 | 821 | 82.7 |  | 2,033 | 13.1 |  | 4,065 | 21.2 |  | 464 | 7.8 |  | 114 | 0.0 |
| Nevada | 2005 | 685 | 83.5 |  | 1,501 | 17.7 |  | 4,206 | 16.6 |  | 485 | 8.7 |  | 110 | 1.8 |
| Nevada | 2006 | 731 | 82.2 |  | 1,707 | 15.8 |  | 3,875 | 18.8 |  | 429 | 4.7 |  | 131 | 1.5 |
| Nevada | 2007 | 689 | 77.1 |  | 1,844 | 14.9 |  | 3,383 | 19.9 |  | 555 | 5.0 |  | 152 | 0.0 |
| Nevada | 2008 | 776 | 64.0 |  | 1,723 | 12.4 |  | 2,538 | 20.1 |  | 680 | 6.5 |  | 186 | 1.6 |
| Nevada | 2009 | 1,033 | 58.9 |  | 1,595 | 12.5 |  | 2,491 | 20.8 |  | 869 | 4.7 |  | 189 | 1.1 |
| Nevada | 2010 | 909 | 66.6 |  | 1,107 | 14.0 |  | 2,486 | 24.1 |  | 881 | 7.9 |  | 252 | 4.0 |
| Nevada | 2011 | 1,018 | 64.7 |  | 1,086 | 16.3 |  | 2,782 | 23.0 |  | 977 | 11.5 |  | 361 | 0.8 |
| Nevada | 2012 | 1,102 | 64.3 |  | 952 | 17.3 |  | 3,195 | 25.3 |  | 1,035 | 11.0 |  | 378 | 2.4 |
| Nevada | 2013 | 1,103 | 61.3 |  | 668 | 15.1 |  | 3,036 | 25.1 |  | 886 | 8.7 |  | 305 | 0.7 |
| Nevada | 2014 | 1,184 | 62.5 |  | 586 | 13.5 |  | 2,957 | 27.4 |  | 786 | 10.9 |  | 204 | 1.5 |
| Nevada | 2015 | 1,091 | 61.5 |  | 408 | 12.3 |  | 2,659 | 26.9 |  | 490 | 8.6 |  | 185 | 0.0 |
| Nevada | 2016 | 1,455 | 52.4 |  | 389 | 16.5 |  | 2,081 | 25.3 |  | 488 | 8.0 |  | 314 | 1.0 |
| Nevada | 2017 | 2,120 | 58.9 |  | 899 | 10.3 |  | 5,201 | 23.7 |  | 772 | 6.9 |  | 553 | 1.6 |
| Nevada | 2018 | 943 | 62.1 |  | 469 | 6.4 |  | 2,954 | 29.9 |  | 339 | 7.4 |  | 168 | 1.8 |
| Nevada | 2019 | 838 | 60.7 |  | 462 | 10.8 |  | 2,528 | 29.2 |  | 266 | 6.4 |  | 133 | 1.5 |
| Nevada | 2020 | 474 | 62.7 |  | 282 | 7.4 |  | 1,529 | 25.2 |  | 194 | 7.7 |  | 63 | 0.0 |
| New Hampshire | 2000 | 532 | 72.4 |  | 1,161 | 8.4 |  | 165 | 7.9 |  | 185 | 10.3 |  | 90 | 1.1 |
| New Hampshire | 2001 | 631 | 69.3 |  | 890 | 7.2 |  | 159 | 5.0 |  | 274 | 3.6 |  | 91 | 0.0 |
| New Hampshire | 2002 | 655 | 63.1 |  | 975 | 9.5 |  | 141 | 5.7 |  | 309 | 6.5 |  | 111 | 0.0 |
| New Hampshire | 2003 | 861 | 74.6 |  | 1,165 | 11.9 |  | 81 | 7.4 |  | 367 | 6.3 |  | 115 | 3.5 |
| New Hampshire | 2004 | 992 | 73.6 |  | 1,443 | 16.9 |  | 105 | 10.5 |  | 510 | 5.7 |  | 134 | 2.2 |
| New Hampshire | 2005 | 885 | 79.1 |  | 1,511 | 20.3 |  | 142 | 14.8 |  | 506 | 8.3 |  | 154 | 3.2 |
| New Hampshire | 2006 | 1,022 | 76.8 |  | 2,020 | 19.1 |  | 180 | 15.6 |  | 689 | 7.4 |  | 139 | 2.9 |
| New Hampshire | 2007 | 916 | 75.3 |  | 1,772 | 18.5 |  | 138 | 15.2 |  | 828 | 7.0 |  | 153 | 3.3 |
| New Hampshire | 2008 | 1,128 | 75.2 |  | 1,790 | 21.1 |  | 143 | 12.6 |  | 1,223 | 9.8 |  | 185 | 2.2 |
| New Hampshire | 2009 | 1,257 | 75.5 |  | 1,499 | 22.9 |  | 148 | 12.2 |  | 1,530 | 12.5 |  | 246 | 2.4 |
| New Hampshire | 2010 | 1,267 | 79.3 |  | 1,397 | 24.6 |  | 165 | 14.5 |  | 1,774 | 19.1 |  | 335 | 2.4 |
| New Hampshire | 2011 | 1,153 | 80.7 |  | 1,191 | 30.4 |  | 131 | 16.8 |  | 1,494 | 18.5 |  | 241 | 1.2 |
| New Hampshire | 2012 | 998 | 86.2 |  | 941 | 34.2 |  | 128 | 19.5 |  | 1,344 | 23.2 |  | 174 | 2.9 |
| New Hampshire | 2013 | 1,454 | 83.9 |  | 928 | 39.5 |  | 137 | 27.7 |  | 1,617 | 21.6 |  | 252 | 4.8 |
| New Hampshire | 2014 | 1,772 | 80.8 |  | 832 | 39.7 |  | 154 | 28.6 |  | 1,240 | 21.5 |  | 198 | 3.0 |
| New Hampshire | 2015 | 1,906 | 76.7 |  | 768 | 38.4 |  | 231 | 42.9 |  | 838 | 19.0 |  | 159 | 6.3 |
| New Hampshire | 2016 | 2,412 | 74.2 |  | 893 | 40.0 |  | 355 | 50.4 |  | 749 | 21.1 |  | 158 | 5.1 |
| New Hampshire | 2017 | 2,531 | 68.4 |  | 1,239 | 36.4 |  | 675 | 56.6 |  | 691 | 21.0 |  | 188 | 1.6 |
| New Hampshire | 2018 | 1,675 | 65.8 |  | 857 | 29.2 |  | 536 | 61.9 |  | 423 | 23.4 |  | 124 | 1.6 |
| New Hampshire | 2019 | 1,085 | 68.2 |  | 630 | 29.0 |  | 477 | 59.1 |  | 268 | 33.2 |  | 71 | 1.4 |
| New Hampshire | 2020 | 775 | 66.8 |  | 416 | 24.8 |  | 469 | 57.1 |  | 159 | 34.0 |  | 49 | 0.0 |
| New Jersey | 2000 | 25,648 | 39.0 |  | 18,353 | 13.5 |  | 371 | 14.6 |  | 1,593 | 5.0 |  | 1,550 | 0.7 |
| New Jersey | 2001 | 28,083 | 38.9 |  | 18,470 | 13.8 |  | 359 | 16.2 |  | 2,050 | 4.9 |  | 1,713 | 0.6 |
| New Jersey | 2002 | 27,974 | 39.8 |  | 19,300 | 14.4 |  | 509 | 13.6 |  | 2,440 | 4.0 |  | 1,824 | 0.7 |
| New Jersey | 2003 | 27,302 | 41.6 |  | 19,490 | 13.2 |  | 488 | 8.4 |  | 2,702 | 4.9 |  | 1,754 | 0.3 |
| New Jersey | 2004 | 25,343 | 44.6 |  | 19,958 | 12.6 |  | 685 | 7.9 |  | 3,528 | 3.3 |  | 2,051 | 0.5 |
| New Jersey | 2005 | 25,337 | 44.9 |  | 20,739 | 12.1 |  | 725 | 7.4 |  | 4,378 | 2.9 |  | 2,163 | 0.2 |
| New Jersey | 2006 | 23,505 | 46.2 |  | 20,820 | 11.7 |  | 834 | 6.1 |  | 5,162 | 2.8 |  | 2,409 | 0.2 |
| New Jersey | 2007 | 24,819 | 45.0 |  | 22,213 | 11.0 |  | 828 | 5.9 |  | 6,322 | 2.4 |  | 2,762 | 0.3 |
| New Jersey | 2008 | 25,814 | 49.9 |  | 21,150 | 11.7 |  | 904 | 4.0 |  | 7,741 | 2.8 |  | 3,300 | 0.3 |
| New Jersey | 2009 | 27,142 | 56.1 |  | 18,827 | 12.8 |  | 1,124 | 5.9 |  | 9,495 | 4.5 |  | 3,933 | 0.4 |
| New Jersey | 2010 | 25,836 | 60.6 |  | 17,521 | 14.0 |  | 1,188 | 3.5 |  | 12,282 | 4.9 |  | 5,324 | 0.5 |
| New Jersey | 2011 | 26,602 | 63.2 |  | 17,018 | 14.8 |  | 1,133 | 4.9 |  | 14,431 | 5.7 |  | 6,154 | 0.7 |
| New Jersey | 2012 | 30,172 | 63.1 |  | 16,938 | 16.1 |  | 1,230 | 6.6 |  | 14,482 | 4.9 |  | 6,347 | 0.5 |
| New Jersey | 2013 | 35,653 | 63.4 |  | 18,235 | 15.2 |  | 1,405 | 7.6 |  | 14,584 | 4.5 |  | 6,758 | 0.4 |
| New Jersey | 2014 | 24,869 | 59.6 |  | 10,823 | 17.7 |  | 803 | 11.0 |  | 7,079 | 5.5 |  | 3,412 | 0.6 |
| New Jersey | 2015 | 29,464 | 61.9 |  | 12,661 | 16.7 |  | 1,131 | 9.9 |  | 8,461 | 5.0 |  | 4,390 | 0.7 |
| New Jersey | 2016 | 36,523 | 64.9 |  | 16,450 | 17.0 |  | 1,605 | 14.8 |  | 10,361 | 5.6 |  | 6,841 | 0.6 |
| New Jersey | 2017 | 40,832 | 63.3 |  | 20,000 | 18.1 |  | 2,125 | 20.2 |  | 10,795 | 5.2 |  | 7,479 | 0.7 |
| New Jersey | 2018 | 44,088 | 58.2 |  | 23,225 | 16.5 |  | 2,933 | 22.9 |  | 11,172 | 5.3 |  | 7,879 | 0.6 |
| New Jersey | 2019 | 47,052 | 54.0 |  | 24,988 | 15.3 |  | 4,131 | 25.2 |  | 11,540 | 5.1 |  | 8,472 | 0.3 |
| New Jersey | 2020 | 37,829 | 51.1 |  | 19,374 | 14.0 |  | 4,434 | 25.0 |  | 9,757 | 6.7 |  | 7,475 | 0.4 |
| New Mexico | 2000 | 681 | 89.3 |  | 902 | 13.1 |  | 233 | 35.6 |  | 275 | 4.4 |  | 59 | 8.5 |
| New Mexico | 2001 | 626 | 89.8 |  | 966 | 13.0 |  | 368 | 31.5 |  | 240 | 2.9 |  | 42 | 4.8 |
| New Mexico | 2002 | 664 | 90.5 |  | 1,045 | 15.5 |  | 387 | 29.2 |  | 230 | 7.4 |  | 49 | 10.2 |
| New Mexico | 2003 | 542 | 86.7 |  | 1,174 | 13.2 |  | 513 | 20.7 |  | 178 | 3.9 |  | 49 | 4.1 |
| New Mexico | 2004 | 441 | 85.3 |  | 801 | 13.9 |  | 468 | 23.7 |  | 163 | 8.0 |  | 41 | 7.3 |
| New Mexico | 2005 | 814 | 85.5 |  | 1,233 | 10.5 |  | 974 | 18.5 |  | 272 | 6.6 |  | 69 | 4.3 |
| New Mexico | 2006 | 804 | 83.3 |  | 1,482 | 8.5 |  | 1,205 | 18.4 |  | 339 | 9.7 |  | 62 | 0.0 |
| New Mexico | 2007 | 761 | 84.9 |  | 1,475 | 9.7 |  | 1,205 | 21.7 |  | 348 | 8.3 |  | 65 | 0.0 |
| New Mexico | 2008 | 780 | 82.3 |  | 1,454 | 9.3 |  | 1,051 | 20.3 |  | 355 | 11.3 |  | 101 | 0.0 |
| New Mexico | 2009 | 652 | 78.1 |  | 1,001 | 9.2 |  | 765 | 22.5 |  | 426 | 11.3 |  | 103 | 1.0 |
| New Mexico | 2010 | 429 | 83.7 |  | 501 | 11.8 |  | 586 | 17.2 |  | 439 | 25.5 |  | 42 | 0.0 |
| New Mexico | 2011 | 497 | 86.9 |  | 537 | 12.5 |  | 904 | 18.9 |  | 635 | 33.9 |  | 65 | 0.0 |
| New Mexico | 2012 | 625 | 86.6 |  | 540 | 11.5 |  | 939 | 21.4 |  | 696 | 40.2 |  | 67 | 0.0 |
| New Mexico | 2013 | 767 | 82.1 |  | 565 | 14.0 |  | 933 | 24.1 |  | 652 | 32.5 |  | 58 | 1.7 |
| New Mexico | 2014 | 598 | 78.4 |  | 373 | 9.1 |  | 589 | 29.7 |  | 580 | 48.1 |  | 61 | 11.5 |
| New Mexico | 2015 | 510 | 80.6 |  | 286 | 13.3 |  | 348 | 35.9 |  | 317 | 32.5 |  | 36 | 0.0 |
| New Mexico | 2016 | 467 | 76.7 |  | 192 | 13.5 |  | 466 | 42.3 |  | 349 | 40.7 |  | 34 | 2.9 |
| New Mexico | 2017 | 606 | 71.9 |  | 286 | 8.7 |  | 712 | 28.5 |  | 270 | 24.8 |  | 48 | 2.1 |
| New Mexico | 2018 | 853 | 61.1 |  | 473 | 4.9 |  | 1,173 | 23.4 |  | 285 | 5.3 |  | 39 | 0.0 |
| New Mexico | 2019 | 943 | 54.4 |  | 441 | 4.3 |  | 1,640 | 22.3 |  | 496 | 3.4 |  | 53 | 0.0 |
| New Mexico | 2020 | Data not reported | |  |  |  |  |  |  |  |  |  |  |  |  |
| New York | 2000 | 73,894 | 43.1 |  | 131,750 | 5.8 |  | 2,289 | 5.7 |  | 7,638 | 4.5 |  | 8,075 | 0.9 |
| New York | 2001 | 77,755 | 43.8 |  | 130,096 | 5.9 |  | 2,564 | 5.0 |  | 8,806 | 5.3 |  | 8,820 | 0.8 |
| New York | 2002 | 80,023 | 45.1 |  | 136,684 | 6.4 |  | 2,946 | 6.4 |  | 10,626 | 5.2 |  | 9,358 | 0.8 |
| New York | 2003 | 79,663 | 45.6 |  | 135,824 | 6.3 |  | 2,950 | 8.2 |  | 11,277 | 4.5 |  | 9,070 | 0.9 |
| New York | 2004 | 71,654 | 47.0 |  | 129,155 | 6.5 |  | 2,706 | 7.1 |  | 11,671 | 3.8 |  | 8,835 | 1.0 |
| New York | 2005 | 78,461 | 48.7 |  | 148,303 | 6.4 |  | 2,591 | 7.2 |  | 13,908 | 3.6 |  | 8,681 | 0.8 |
| New York | 2006 | 75,888 | 49.2 |  | 149,583 | 6.9 |  | 2,339 | 8.3 |  | 15,179 | 3.6 |  | 9,803 | 0.7 |
| New York | 2007 | 73,371 | 48.5 |  | 145,178 | 7.0 |  | 3,509 | 6.1 |  | 20,458 | 3.4 |  | 12,641 | 0.7 |
| New York | 2008 | 77,556 | 49.1 |  | 135,825 | 7.2 |  | 3,657 | 5.0 |  | 23,974 | 3.8 |  | 14,471 | 0.5 |
| New York | 2009 | 78,582 | 52.2 |  | 124,275 | 7.4 |  | 3,948 | 5.1 |  | 28,254 | 4.9 |  | 17,627 | 0.7 |
| New York | 2010 | 74,888 | 55.0 |  | 118,644 | 8.0 |  | 4,474 | 5.5 |  | 34,791 | 6.5 |  | 21,233 | 0.8 |
| New York | 2011 | 73,971 | 54.9 |  | 114,325 | 8.4 |  | 4,647 | 7.5 |  | 41,164 | 7.4 |  | 23,472 | 0.7 |
| New York | 2012 | 79,122 | 57.8 |  | 104,866 | 9.4 |  | 5,021 | 10.1 |  | 42,917 | 8.7 |  | 25,226 | 0.8 |
| New York | 2013 | 87,267 | 59.0 |  | 97,578 | 10.4 |  | 5,200 | 13.1 |  | 40,989 | 9.2 |  | 26,320 | 0.7 |
| New York | 2014 | 96,818 | 60.7 |  | 92,403 | 11.3 |  | 5,643 | 16.0 |  | 38,141 | 8.9 |  | 28,986 | 0.7 |
| New York | 2015 | 104,269 | 60.8 |  | 89,130 | 13.0 |  | 6,719 | 20.6 |  | 37,454 | 8.6 |  | 30,425 | 0.8 |
| New York | 2016 | 108,093 | 60.9 |  | 92,886 | 14.8 |  | 7,785 | 25.0 |  | 35,991 | 8.4 |  | 31,524 | 0.6 |
| New York | 2017 | 105,966 | 59.4 |  | 97,530 | 14.8 |  | 9,059 | 28.1 |  | 32,946 | 8.2 |  | 29,417 | 0.8 |
| New York | 2018 | 99,263 | 56.9 |  | 101,441 | 14.8 |  | 11,438 | 34.0 |  | 29,490 | 7.9 |  | 26,549 | 0.8 |
| New York | 2019 | 96,054 | 53.5 |  | 101,999 | 14.3 |  | 13,704 | 38.0 |  | 26,097 | 8.1 |  | 24,547 | 1.0 |
| New York | 2020 | 71,305 | 52.0 |  | 75,067 | 13.7 |  | 13,398 | 37.5 |  | 19,254 | 8.8 |  | 18,285 | 1.1 |
| North Carolina | 2000 | 1,237 | 66.9 |  | 10,587 | 3.1 |  | 344 | 10.5 |  | 1,099 | 27.7 |  | 1,034 | 1.1 |
| North Carolina | 2001 | 1,120 | 64.9 |  | 9,665 | 3.0 |  | 546 | 9.2 |  | 1,455 | 21.6 |  | 1,052 | 0.7 |
| North Carolina | 2002 | 1,109 | 65.7 |  | 9,744 | 3.3 |  | 556 | 7.4 |  | 1,463 | 15.7 |  | 1,029 | 1.1 |
| North Carolina | 2003 | 1,178 | 71.8 |  | 9,072 | 3.5 |  | 598 | 8.7 |  | 1,590 | 12.1 |  | 901 | 0.7 |
| North Carolina | 2004 | 1,349 | 70.5 |  | 9,151 | 4.3 |  | 565 | 12.7 |  | 1,944 | 12.2 |  | 830 | 1.0 |
| North Carolina | 2005 | 983 | 71.7 |  | 11,577 | 2.8 |  | 783 | 14.4 |  | 2,554 | 39.7 |  | 905 | 1.0 |
| North Carolina | 2006 | 972 | 73.6 |  | 8,787 | 3.8 |  | 519 | 15.8 |  | 2,373 | 38.6 |  | 698 | 0.7 |
| North Carolina | 2007 | 1,483 | 69.7 |  | 11,067 | 4.4 |  | 575 | 12.3 |  | 3,082 | 9.3 |  | 976 | 1.2 |
| North Carolina | 2008 | 1,704 | 70.8 |  | 12,195 | 2.9 |  | 774 | 12.9 |  | 4,427 | 8.4 |  | 1,526 | 0.9 |
| North Carolina | 2009 | 2,152 | 74.6 |  | 13,270 | 2.9 |  | 1,253 | 16.0 |  | 6,908 | 9.6 |  | 2,776 | 1.9 |
| North Carolina | 2010 | 2,246 | 77.0 |  | 13,727 | 3.4 |  | 1,486 | 17.8 |  | 9,697 | 12.7 |  | 3,699 | 2.1 |
| North Carolina | 2011 | 3,055 | 79.7 |  | 15,877 | 4.2 |  | 1,987 | 22.7 |  | 12,647 | 13.5 |  | 4,457 | 1.1 |
| North Carolina | 2012 | 3,488 | 80.0 |  | 14,174 | 4.9 |  | 1,810 | 23.3 |  | 11,920 | 14.9 |  | 3,757 | 1.2 |
| North Carolina | 2013 | 4,031 | 80.9 |  | 11,938 | 5.4 |  | 2,250 | 24.4 |  | 11,869 | 17.7 |  | 3,199 | 1.3 |
| North Carolina | 2014 | 3,469 | 80.5 |  | 8,521 | 5.9 |  | 2,139 | 25.5 |  | 8,759 | 18.9 |  | 2,849 | 1.7 |
| North Carolina | 2015 | 5,929 | 79.2 |  | 10,873 | 7.6 |  | 3,155 | 30.3 |  | 11,377 | 20.8 |  | 3,733 | 2.4 |
| North Carolina | 2016 | 12,679 | 79.2 |  | 19,803 | 9.9 |  | 7,435 | 33.3 |  | 20,758 | 23.0 |  | 7,572 | 1.7 |
| North Carolina | 2017 | 12,459 | 76.3 |  | 17,110 | 10.6 |  | 7,628 | 35.5 |  | 17,196 | 20.0 |  | 5,935 | 1.6 |
| North Carolina | 2018 | 8,101 | 73.0 |  | 10,285 | 10.2 |  | 5,835 | 35.5 |  | 9,828 | 18.1 |  | 2,541 | 1.3 |
| North Carolina | 2019 | 9,428 | 67.3 |  | 10,220 | 9.0 |  | 7,920 | 33.9 |  | 9,528 | 17.0 |  | 2,385 | 2.5 |
| North Carolina | 2020 | 7,642 | 64.3 |  | 6,698 | 8.9 |  | 6,763 | 33.8 |  | 6,900 | 17.8 |  | 2,206 | 1.6 |
| North Dakota | 2000 | 20 | 60.0 |  | 106 | 16.0 |  | 281 | 29.5 |  | 107 | 2.8 |  | 29 | 20.7 |
| North Dakota | 2001 | 25 | 72.0 |  | 99 | 22.2 |  | 370 | 24.6 |  | 157 | 7.6 |  | 41 | 24.4 |
| North Dakota | 2002 | 35 | 60.0 |  | 190 | 30.0 |  | 847 | 26.9 |  | 159 | 8.8 |  | 42 | 4.8 |
| North Dakota | 2003 | 16 | 56.3 |  | 101 | 15.8 |  | 490 | 25.9 |  | 48 | 29.2 |  | 13 | 0.0 |
| North Dakota | 2004 | 30 | 83.3 |  | 132 | 29.5 |  | 804 | 28.7 |  | 112 | 27.7 |  | 30 | 6.7 |
| North Dakota | 2005 | 12 | 66.7 |  | 129 | 18.6 |  | 738 | 25.2 |  | 77 | 31.2 |  | 23 | 0.0 |
| North Dakota | 2006 | 9 | 88.9 |  | 160 | 13.8 |  | 752 | 24.6 |  | 137 | 26.3 |  | 21 | 0.0 |
| North Dakota | 2007 | 12 | 83.3 |  | 147 | 17.0 |  | 574 | 22.3 |  | 134 | 17.9 |  | 15 | 0.0 |
| North Dakota | 2008 | 17 | 82.4 |  | 156 | 20.5 |  | 505 | 27.1 |  | 175 | 25.1 |  | 22 | 0.0 |
| North Dakota | 2009 | 20 | 70.0 |  | 104 | 12.5 |  | 415 | 29.2 |  | 209 | 24.9 |  | 24 | 4.2 |
| North Dakota | 2010 | 18 | 88.9 |  | 109 | 13.8 |  | 393 | 30.3 |  | 253 | 24.9 |  | 20 | 0.0 |
| North Dakota | 2011 | 11 | 72.7 |  | 65 | 15.4 |  | 284 | 34.9 |  | 195 | 29.7 |  | 16 | 0.0 |
| North Dakota | 2012 | 34 | 82.4 |  | 95 | 12.6 |  | 557 | 38.1 |  | 346 | 32.1 |  | 41 | 0.0 |
| North Dakota | 2013 | 51 | 74.5 |  | 80 | 15.0 |  | 809 | 39.1 |  | 373 | 34.6 |  | 43 | 4.7 |
| North Dakota | 2014 | 109 | 77.1 |  | 115 | 4.3 |  | 1,236 | 38.0 |  | 468 | 41.2 |  | 46 | 4.3 |
| North Dakota | 2015 | 154 | 72.7 |  | 73 | 19.2 |  | 1,161 | 38.2 |  | 355 | 34.9 |  | 45 | 2.2 |
| North Dakota | 2016 | 464 | 79.1 |  | 99 | 9.1 |  | 2,818 | 41.4 |  | 749 | 30.0 |  | 67 | 7.5 |
| North Dakota | 2017 | 514 | 76.1 |  | 92 | 13.0 |  | 2,647 | 44.5 |  | 533 | 34.3 |  | 44 | 0.0 |
| North Dakota | 2018 | 343 | 77.3 |  | 42 | 19.0 |  | 1,929 | 44.9 |  | 295 | 37.6 |  | 38 | 5.3 |
| North Dakota | 2019 | 78 | 76.9 |  | 19 | 26.3 |  | 371 | 43.1 |  | 66 | 21.2 |  | 8 | 12.5 |
| North Dakota | 2020 | 123 | 71.5 |  | 46 | 10.9 |  | 805 | 45.1 |  | 136 | 31.6 |  | 15 | 0.0 |
| Ohio | 2000 | 3,369 | 79.8 |  | 13,990 | 2.2 |  | 493 | 5.9 |  | 1,032 | 9.3 |  | 866 | 0.8 |
| Ohio | 2001 | 4,150 | 77.7 |  | 16,229 | 2.7 |  | 628 | 7.0 |  | 1,503 | 10.1 |  | 1,220 | 0.7 |
| Ohio | 2002 | 5,041 | 78.6 |  | 18,766 | 3.1 |  | 914 | 8.0 |  | 2,678 | 8.7 |  | 1,490 | 0.3 |
| Ohio | 2003 | 4,673 | 78.4 |  | 16,676 | 3.7 |  | 885 | 9.4 |  | 2,685 | 9.5 |  | 1,268 | 0.6 |
| Ohio | 2004 | 4,855 | 78.2 |  | 17,023 | 4.0 |  | 1,096 | 9.3 |  | 2,964 | 12.0 |  | 1,251 | 1.1 |
| Ohio | 2005 | 7,823 | 78.0 |  | 23,659 | 5.1 |  | 1,835 | 8.7 |  | 4,759 | 10.5 |  | 1,986 | 0.8 |
| Ohio | 2006 | 8,225 | 76.2 |  | 24,838 | 5.6 |  | 1,803 | 7.5 |  | 5,319 | 10.2 |  | 2,119 | 0.8 |
| Ohio | 2007 | 10,063 | 75.9 |  | 28,085 | 5.2 |  | 1,788 | 8.2 |  | 7,260 | 12.1 |  | 2,669 | 0.5 |
| Ohio | 2008 | 10,439 | 75.6 |  | 22,538 | 4.5 |  | 1,466 | 7.0 |  | 7,368 | 12.6 |  | 2,543 | 0.9 |
| Ohio | 2009 | 10,305 | 78.2 |  | 16,841 | 2.5 |  | 1,374 | 9.1 |  | 8,153 | 13.1 |  | 2,742 | 0.9 |
| Ohio | 2010 | 12,184 | 79.8 |  | 18,517 | 1.0 |  | 1,752 | 9.6 |  | 13,353 | 11.2 |  | 3,871 | 0.8 |
| Ohio | 2011 | 13,591 | 81.5 |  | 16,767 | 1.0 |  | 1,992 | 11.9 |  | 14,412 | 10.4 |  | 4,206 | 1.0 |
| Ohio | 2012 | 14,514 | 79.5 |  | 13,178 | 1.5 |  | 1,884 | 12.6 |  | 12,782 | 10.8 |  | 3,257 | 0.9 |
| Ohio | 2013 | 13,928 | 77.8 |  | 10,319 | 0.7 |  | 1,800 | 17.2 |  | 11,013 | 11.6 |  | 2,634 | 1.1 |
| Ohio | 2014 | 16,041 | 74.7 |  | 9,996 | 1.3 |  | 1,863 | 19.0 |  | 9,770 | 10.3 |  | 2,539 | 1.3 |
| Ohio | 2015 | 17,783 | 74.3 |  | 10,561 | 2.0 |  | 2,258 | 22.9 |  | 10,031 | 10.3 |  | 2,522 | 1.3 |
| Ohio | 2016 | 17,977 | 73.2 |  | 10,776 | 2.3 |  | 3,029 | 31.0 |  | 8,933 | 11.3 |  | 2,479 | 2.0 |
| Ohio | 2017 | 17,844 | 70.3 |  | 11,298 | 1.9 |  | 5,092 | 33.8 |  | 8,458 | 11.3 |  | 2,572 | 1.6 |
| Ohio | 2018 | 13,437 | 69.6 |  | 9,314 | 1.5 |  | 6,653 | 34.1 |  | 6,399 | 11.8 |  | 2,015 | 1.3 |
| Ohio | 2019 | 8,281 | 66.5 |  | 5,593 | 2.2 |  | 5,602 | 36.4 |  | 4,048 | 15.4 |  | 1,193 | 2.1 |
| Ohio | 2020 | 6,280 | 63.3 |  | 3,922 | 4.7 |  | 5,133 | 33.2 |  | 3,297 | 17.9 |  | 964 | 5.5 |
| Oklahoma | 2000 | 245 | 88.2 |  | 2,762 | 18.3 |  | 4,290 | 54.5 |  | 450 | 16.0 |  | 567 | 3.4 |
| Oklahoma | 2001 | 309 | 79.6 |  | 3,283 | 16.6 |  | 5,521 | 54.4 |  | 732 | 17.1 |  | 770 | 3.9 |
| Oklahoma | 2002 | 244 | 85.2 |  | 3,463 | 14.4 |  | 5,523 | 51.2 |  | 861 | 16.8 |  | 858 | 3.7 |
| Oklahoma | 2003 | 266 | 79.7 |  | 3,276 | 15.0 |  | 5,448 | 49.7 |  | 1,006 | 18.7 |  | 833 | 3.8 |
| Oklahoma | 2004 | 262 | 81.3 |  | 3,728 | 13.8 |  | 5,937 | 47.4 |  | 1,341 | 15.2 |  | 1,035 | 2.4 |
| Oklahoma | 2005 | 206 | 73.3 |  | 3,649 | 12.3 |  | 6,218 | 45.4 |  | 1,457 | 13.5 |  | 950 | 2.4 |
| Oklahoma | 2006 | 156 | 78.8 |  | 3,260 | 13.3 |  | 5,571 | 45.8 |  | 1,525 | 14.4 |  | 981 | 2.9 |
| Oklahoma | 2007 | 187 | 71.1 |  | 3,235 | 13.3 |  | 5,347 | 41.2 |  | 1,890 | 12.9 |  | 1,021 | 2.4 |
| Oklahoma | 2008 | 296 | 74.7 |  | 2,787 | 14.0 |  | 4,471 | 42.8 |  | 2,260 | 13.7 |  | 1,075 | 2.4 |
| Oklahoma | 2009 | 367 | 77.9 |  | 2,234 | 11.9 |  | 4,847 | 42.0 |  | 2,546 | 16.3 |  | 1,104 | 4.0 |
| Oklahoma | 2010 | 312 | 77.2 |  | 1,420 | 11.8 |  | 4,248 | 38.8 |  | 2,570 | 16.8 |  | 1,015 | 2.2 |
| Oklahoma | 2011 | 323 | 81.1 |  | 1,164 | 11.9 |  | 4,216 | 40.8 |  | 2,427 | 17.6 |  | 1,074 | 2.3 |
| Oklahoma | 2012 | 373 | 87.4 |  | 934 | 10.5 |  | 4,633 | 43.5 |  | 2,609 | 19.0 |  | 1,116 | 1.5 |
| Oklahoma | 2013 | 454 | 90.5 |  | 840 | 14.6 |  | 5,193 | 44.8 |  | 2,549 | 21.4 |  | 1,107 | 2.1 |
| Oklahoma | 2014 | 513 | 87.5 |  | 894 | 9.7 |  | 6,093 | 41.6 |  | 2,728 | 21.0 |  | 1,079 | 2.0 |
| Oklahoma | 2015 | 640 | 85.5 |  | 777 | 11.3 |  | 6,738 | 43.5 |  | 2,766 | 22.9 |  | 1,158 | 2.2 |
| Oklahoma | 2016 | 913 | 79.5 |  | 734 | 10.2 |  | 7,400 | 42.9 |  | 2,712 | 21.9 |  | 1,075 | 1.8 |
| Oklahoma | 2017 | 1,101 | 76.7 |  | 780 | 9.4 |  | 7,903 | 40.3 |  | 2,475 | 20.8 |  | 982 | 2.1 |
| Oklahoma | 2018 | 1,296 | 75.8 |  | 770 | 8.1 |  | 8,003 | 41.3 |  | 2,669 | 18.6 |  | 967 | 1.4 |
| Oklahoma | 2019 | 1,681 | 74.9 |  | 697 | 9.9 |  | 7,922 | 39.9 |  | 2,394 | 17.8 |  | 882 | 1.6 |
| Oklahoma | 2020 | 1,567 | 69.4 |  | 658 | 6.8 |  | 7,163 | 35.0 |  | 1,781 | 13.4 |  | 639 | 1.3 |
| Oregon | 2000 | 5,879 | 89.1 |  | 5,118 | 31.5 |  | 12,591 | 37.7 |  | 851 | 10.1 |  | 421 | 3.1 |
| Oregon | 2001 | 5,455 | 86.0 |  | 5,553 | 30.5 |  | 14,004 | 36.5 |  | 1,152 | 8.1 |  | 502 | 3.8 |
| Oregon | 2002 | 5,498 | 86.8 |  | 5,573 | 30.1 |  | 14,924 | 36.2 |  | 1,329 | 9.3 |  | 588 | 3.7 |
| Oregon | 2003 | 3,887 | 86.2 |  | 3,798 | 30.4 |  | 11,311 | 32.7 |  | 1,123 | 8.6 |  | 391 | 2.8 |
| Oregon | 2004 | 3,436 | 85.5 |  | 3,470 | 26.2 |  | 12,330 | 30.1 |  | 1,421 | 8.4 |  | 395 | 2.0 |
| Oregon | 2005 | 3,767 | 81.4 |  | 3,619 | 20.6 |  | 13,912 | 26.2 |  | 1,812 | 8.1 |  | 468 | 4.5 |
| Oregon | 2006 | 4,118 | 80.7 |  | 4,170 | 23.1 |  | 13,563 | 26.7 |  | 2,215 | 8.3 |  | 550 | 4.0 |
| Oregon | 2007 | 4,556 | 81.4 |  | 4,357 | 20.8 |  | 13,479 | 25.7 |  | 3,022 | 7.1 |  | 689 | 0.9 |
| Oregon | 2008 | 4,682 | 79.0 |  | 4,160 | 18.2 |  | 12,175 | 26.9 |  | 3,704 | 8.3 |  | 867 | 2.5 |
| Oregon | 2009 | 5,146 | 77.1 |  | 3,512 | 18.3 |  | 11,533 | 28.1 |  | 4,460 | 8.5 |  | 949 | 3.3 |
| Oregon | 2010 | 5,905 | 78.4 |  | 3,232 | 19.3 |  | 11,963 | 28.6 |  | 4,852 | 9.9 |  | 1,094 | 2.4 |
| Oregon | 2011 | 6,856 | 76.3 |  | 2,919 | 21.5 |  | 11,929 | 30.7 |  | 5,197 | 10.7 |  | 1,156 | 2.8 |
| Oregon | 2012 | 7,022 | 75.4 |  | 2,354 | 19.2 |  | 12,010 | 31.3 |  | 5,112 | 9.8 |  | 1,028 | 3.3 |
| Oregon | 2013 | 7,228 | 72.1 |  | 1,756 | 15.4 |  | 12,598 | 32.9 |  | 4,803 | 10.0 |  | 904 | 2.7 |
| Oregon | 2014 | 6,168 | 72.3 |  | 1,079 | 17.3 |  | 9,701 | 32.9 |  | 3,326 | 10.8 |  | 570 | 2.3 |
| Oregon | 2015 | Data not reported | |  |  |  |  |  |  |  |  |  |  |  |  |
| Oregon | 2016 | Data not reported | |  |  |  |  |  |  |  |  |  |  |  |  |
| Oregon | 2017 | Data not reported | |  |  |  |  |  |  |  |  |  |  |  |  |
| Oregon | 2018 | Data not reported | |  |  |  |  |  |  |  |  |  |  |  |  |
| Oregon | 2019 | Data not reported | |  |  |  |  |  |  |  |  |  |  |  |  |
| Oregon | 2020 | Data not reported | |  |  |  |  |  |  |  |  |  |  |  |  |
| Pennsylvania | 2000 | 11,885 | 65.2 |  | 22,552 | 6.2 |  | 892 | 20.3 |  | 2,661 | 6.1 |  | 2,499 | 1.5 |
| Pennsylvania | 2001 | 12,605 | 64.8 |  | 20,062 | 6.5 |  | 909 | 16.9 |  | 3,859 | 9.3 |  | 2,477 | 1.5 |
| Pennsylvania | 2002 | 14,216 | 66.7 |  | 20,053 | 6.7 |  | 1,030 | 13.4 |  | 4,325 | 9.1 |  | 2,177 | 1.3 |
| Pennsylvania | 2003 | 17,490 | 69.5 |  | 21,782 | 7.0 |  | 991 | 12.7 |  | 5,676 | 11.3 |  | 2,667 | 1.4 |
| Pennsylvania | 2004 | 21,809 | 70.7 |  | 29,268 | 6.8 |  | 1,832 | 10.8 |  | 7,928 | 9.4 |  | 3,341 | 1.3 |
| Pennsylvania | 2005 | 18,266 | 70.0 |  | 26,700 | 6.7 |  | 1,643 | 10.6 |  | 7,444 | 11.1 |  | 3,215 | 1.2 |
| Pennsylvania | 2006 | 15,984 | 70.8 |  | 24,837 | 7.2 |  | 1,531 | 8.6 |  | 7,467 | 7.4 |  | 3,180 | 0.9 |
| Pennsylvania | 2007 | 13,995 | 74.0 |  | 21,518 | 7.6 |  | 1,186 | 11.6 |  | 7,945 | 9.6 |  | 2,719 | 0.8 |
| Pennsylvania | 2008 | 17,919 | 75.7 |  | 21,730 | 8.3 |  | 1,135 | 10.3 |  | 10,426 | 10.2 |  | 3,521 | 1.0 |
| Pennsylvania | 2009 | 14,375 | 74.7 |  | 14,899 | 8.0 |  | 810 | 11.4 |  | 9,261 | 11.0 |  | 2,951 | 0.8 |
| Pennsylvania | 2010 | 14,615 | 77.2 |  | 14,296 | 10.0 |  | 931 | 14.1 |  | 11,466 | 13.1 |  | 3,503 | 1.7 |
| Pennsylvania | 2011 | 12,912 | 77.4 |  | 11,505 | 11.5 |  | 841 | 17.4 |  | 10,339 | 13.9 |  | 3,271 | 1.9 |
| Pennsylvania | 2012 | 12,251 | 82.8 |  | 7,143 | 12.3 |  | 711 | 22.1 |  | 7,835 | 15.7 |  | 2,464 | 1.7 |
| Pennsylvania | 2013 | 15,410 | 85.5 |  | 5,343 | 14.3 |  | 749 | 27.4 |  | 6,585 | 16.5 |  | 2,059 | 1.7 |
| Pennsylvania | 2014 | 16,528 | 85.3 |  | 4,397 | 13.9 |  | 918 | 27.2 |  | 6,042 | 17.6 |  | 1,821 | 1.4 |
| Pennsylvania | 2015 | 12,337 | 82.7 |  | 3,455 | 14.2 |  | 891 | 29.9 |  | 4,610 | 16.7 |  | 1,416 | 1.4 |
| Pennsylvania | 2016 | 9,566 | 79.4 |  | 3,219 | 15.7 |  | 1,118 | 32.0 |  | 3,563 | 13.0 |  | 1,407 | 1.2 |
| Pennsylvania | 2017 | 9,719 | 75.0 |  | 3,722 | 16.4 |  | 1,589 | 37.5 |  | 3,652 | 14.8 |  | 1,288 | 1.0 |
| Pennsylvania | 2018 | 9,853 | 74.6 |  | 4,502 | 17.7 |  | 2,924 | 38.6 |  | 3,870 | 16.7 |  | 1,420 | 1.8 |
| Pennsylvania | 2019 | 10,169 | 72.4 |  | 5,238 | 16.4 |  | 4,951 | 41.1 |  | 4,286 | 13.8 |  | 1,624 | 1.5 |
| Pennsylvania | 2020 | 7,895 | 69.1 |  | 3,973 | 16.8 |  | 4,636 | 41.1 |  | 3,059 | 14.6 |  | 1,467 | 1.2 |
| Rhode Island | 2000 | 5,248 | 68.4 |  | 3,710 | 14.4 |  | 69 | 11.6 |  | 774 | 3.0 |  | 511 | 0.4 |
| Rhode Island | 2001 | 5,530 | 72.3 |  | 3,903 | 11.1 |  | 90 | 5.6 |  | 999 | 3.3 |  | 427 | 0.9 |
| Rhode Island | 2002 | 4,900 | 70.9 |  | 3,963 | 10.2 |  | 92 | 5.4 |  | 1,206 | 3.9 |  | 465 | 0.2 |
| Rhode Island | 2003 | 4,905 | 71.2 |  | 4,081 | 8.5 |  | 50 | 12.0 |  | 1,399 | 3.1 |  | 475 | 0.6 |
| Rhode Island | 2004 | 4,660 | 72.5 |  | 4,035 | 7.9 |  | 34 | 5.9 |  | 1,572 | 3.8 |  | 478 | 1.0 |
| Rhode Island | 2005 | 4,004 | 74.0 |  | 3,976 | 6.3 |  | 58 | 6.9 |  | 1,601 | 3.6 |  | 497 | 0.8 |
| Rhode Island | 2006 | 3,435 | 73.5 |  | 3,626 | 5.1 |  | 96 | 6.3 |  | 1,760 | 3.4 |  | 454 | 0.9 |
| Rhode Island | 2007 | 2,768 | 75.8 |  | 3,569 | 4.4 |  | 113 | 0.0 |  | 1,806 | 3.7 |  | 503 | 0.6 |
| Rhode Island | 2008 | 3,011 | 74.5 |  | 3,540 | 6.6 |  | 130 | 6.2 |  | 1,973 | 4.8 |  | 551 | 0.2 |
| Rhode Island | 2009 | 2,761 | 75.3 |  | 3,223 | 8.1 |  | 124 | 4.0 |  | 1,882 | 7.2 |  | 527 | 0.9 |
| Rhode Island | 2010 | 2,411 | 75.3 |  | 2,422 | 7.9 |  | 49 | 8.2 |  | 2,083 | 6.9 |  | 553 | 0.9 |
| Rhode Island | 2011 | 2,864 | 77.3 |  | 2,413 | 8.9 |  | 71 | 7.0 |  | 2,320 | 5.8 |  | 605 | 0.5 |
| Rhode Island | 2012 | 2,981 | 76.4 |  | 2,377 | 8.0 |  | 87 | 13.8 |  | 2,230 | 5.9 |  | 659 | 0.6 |
| Rhode Island | 2013 | 4,015 | 78.4 |  | 2,390 | 8.0 |  | 137 | 15.3 |  | 2,343 | 5.0 |  | 639 | 1.7 |
| Rhode Island | 2014 | 4,179 | 78.2 |  | 2,364 | 9.3 |  | 157 | 14.6 |  | 1,958 | 5.7 |  | 716 | 0.3 |
| Rhode Island | 2015 | 4,379 | 72.5 |  | 2,418 | 10.4 |  | 194 | 12.4 |  | 1,775 | 5.5 |  | 656 | 0.6 |
| Rhode Island | 2016 | 5,009 | 69.3 |  | 3,060 | 11.7 |  | 287 | 10.1 |  | 1,841 | 5.5 |  | 957 | 0.5 |
| Rhode Island | 2017 | 4,683 | 63.6 |  | 3,325 | 11.4 |  | 338 | 11.5 |  | 2,071 | 8.8 |  | 926 | 0.9 |
| Rhode Island | 2018 | 4,523 | 62.8 |  | 4,003 | 12.3 |  | 414 | 18.4 |  | 2,949 | 16.8 |  | 895 | 2.0 |
| Rhode Island | 2019 | 4,163 | 59.0 |  | 4,123 | 12.2 |  | 549 | 28.1 |  | 3,403 | 23.2 |  | 921 | 1.8 |
| Rhode Island | 2020 | 3,381 | 57.9 |  | 3,794 | 10.2 |  | 665 | 26.3 |  | 4,285 | 26.2 |  | 1,050 | 2.2 |
| South Carolina | 2000 | 466 | 66.7 |  | 5,923 | 1.9 |  | 283 | 10.2 |  | 647 | 9.9 |  | 537 | 0.2 |
| South Carolina | 2001 | 514 | 64.2 |  | 6,582 | 2.1 |  | 341 | 12.3 |  | 1,099 | 15.4 |  | 668 | 1.2 |
| South Carolina | 2002 | 545 | 69.4 |  | 5,907 | 2.0 |  | 454 | 11.2 |  | 1,309 | 15.9 |  | 679 | 0.4 |
| South Carolina | 2003 | 556 | 69.1 |  | 5,755 | 2.7 |  | 526 | 7.8 |  | 1,273 | 12.7 |  | 649 | 0.6 |
| South Carolina | 2004 | 426 | 66.0 |  | 6,266 | 2.2 |  | 716 | 8.8 |  | 1,297 | 10.6 |  | 674 | 0.6 |
| South Carolina | 2005 | 590 | 65.8 |  | 8,179 | 2.6 |  | 1,189 | 10.8 |  | 1,894 | 10.5 |  | 758 | 0.4 |
| South Carolina | 2006 | 397 | 66.0 |  | 8,165 | 2.7 |  | 1,132 | 7.1 |  | 1,936 | 8.4 |  | 853 | 0.7 |
| South Carolina | 2007 | 334 | 64.7 |  | 7,731 | 1.8 |  | 1,043 | 8.6 |  | 2,008 | 7.6 |  | 903 | 0.4 |
| South Carolina | 2008 | 361 | 63.7 |  | 6,959 | 1.9 |  | 985 | 7.4 |  | 2,382 | 6.7 |  | 1,090 | 0.1 |
| South Carolina | 2009 | 383 | 71.5 |  | 5,707 | 2.3 |  | 952 | 8.0 |  | 2,760 | 6.9 |  | 1,354 | 0.7 |
| South Carolina | 2010 | 337 | 75.1 |  | 4,631 | 2.3 |  | 1,044 | 11.8 |  | 2,878 | 9.7 |  | 1,318 | 0.1 |
| South Carolina | 2011 | 351 | 77.8 |  | 3,962 | 2.6 |  | 1,112 | 12.6 |  | 2,919 | 9.9 |  | 1,318 | 0.5 |
| South Carolina | 2012 | 396 | 78.8 |  | 3,824 | 3.0 |  | 1,413 | 15.1 |  | 3,297 | 11.8 |  | 1,430 | 0.6 |
| South Carolina | 2013 | 303 | 83.8 |  | 2,676 | 3.4 |  | 1,268 | 15.1 |  | 2,670 | 13.2 |  | 1,138 | 0.9 |
| South Carolina | 2014 | Data not reported | |  |  |  |  |  |  |  |  |  |  |  |  |
| South Carolina | 2015 | 272 | 75.0 |  | 954 | 4.9 |  | 657 | 20.4 |  | 672 | 12.1 |  | 413 | 0.7 |
| South Carolina | 2016 | 882 | 75.5 |  | 2,822 | 4.3 |  | 2,196 | 17.5 |  | 2,107 | 9.0 |  | 1,240 | 0.4 |
| South Carolina | 2017 | 2,589 | 72.2 |  | 5,041 | 5.5 |  | 3,819 | 21.9 |  | 4,192 | 10.1 |  | 2,424 | 0.5 |
| South Carolina | 2018 | 3,080 | 71.2 |  | 5,171 | 5.7 |  | 4,429 | 26.6 |  | 4,487 | 10.3 |  | 2,530 | 0.2 |
| South Carolina | 2019 | 3,295 | 66.8 |  | 4,660 | 5.3 |  | 4,847 | 24.4 |  | 4,200 | 9.0 |  | 1,975 | 0.6 |
| South Carolina | 2020 | 1,717 | 40.9 |  | 3,242 | 1.5 |  | 3,248 | 10.5 |  | 3,356 | 3.5 |  | 1,492 | 0.1 |
| South Dakota | 2000 | 22 | 40.9 |  | 474 | 24.7 |  | 724 | 28.6 |  | 52 | 15.4 |  | 43 | 2.3 |
| South Dakota | 2001 | 23 | 60.9 |  | 344 | 16.9 |  | 618 | 21.7 |  | 47 | 25.5 |  | 23 | 8.7 |
| South Dakota | 2002 | 29 | 79.3 |  | 446 | 24.4 |  | 1,091 | 28.1 |  | 101 | 13.9 |  | 38 | 5.3 |
| South Dakota | 2003 | 34 | 73.5 |  | 549 | 22.2 |  | 1,211 | 31.3 |  | 110 | 11.8 |  | 44 | 0.0 |
| South Dakota | 2004 | 37 | 62.2 |  | 607 | 20.6 |  | 1,422 | 29.1 |  | 152 | 12.5 |  | 44 | 4.5 |
| South Dakota | 2005 | 72 | 72.2 |  | 958 | 25.3 |  | 2,595 | 32.6 |  | 271 | 17.7 |  | 62 | 3.2 |
| South Dakota | 2006 | 79 | 55.7 |  | 1,030 | 20.6 |  | 2,598 | 29.6 |  | 320 | 12.5 |  | 83 | 2.4 |
| South Dakota | 2007 | 64 | 65.6 |  | 1,065 | 20.8 |  | 2,313 | 29.2 |  | 374 | 13.4 |  | 117 | 1.7 |
| South Dakota | 2008 | 62 | 71.0 |  | 941 | 23.9 |  | 1,938 | 31.1 |  | 488 | 14.5 |  | 134 | 2.2 |
| South Dakota | 2009 | 70 | 72.9 |  | 848 | 25.2 |  | 1,847 | 31.6 |  | 561 | 17.3 |  | 160 | 0.6 |
| South Dakota | 2010 | 82 | 64.6 |  | 749 | 22.0 |  | 1,905 | 32.2 |  | 776 | 18.2 |  | 209 | 1.0 |
| South Dakota | 2011 | 131 | 67.9 |  | 751 | 23.6 |  | 2,030 | 37.0 |  | 834 | 21.9 |  | 158 | 2.5 |
| South Dakota | 2012 | 173 | 64.2 |  | 694 | 20.9 |  | 2,194 | 32.3 |  | 863 | 21.6 |  | 184 | 2.7 |
| South Dakota | 2013 | 175 | 64.0 |  | 504 | 23.6 |  | 2,189 | 32.9 |  | 846 | 16.1 |  | 185 | 2.2 |
| South Dakota | 2014 | 144 | 67.4 |  | 477 | 22.4 |  | 2,321 | 36.8 |  | 626 | 22.2 |  | 113 | 1.8 |
| South Dakota | 2015 | 135 | 68.1 |  | 367 | 18.3 |  | 2,587 | 36.1 |  | 657 | 20.4 |  | 125 | 2.4 |
| South Dakota | 2016 | 178 | 75.8 |  | 366 | 24.3 |  | 3,178 | 42.4 |  | 731 | 24.8 |  | 163 | 1.8 |
| South Dakota | 2017 | 223 | 75.3 |  | 414 | 18.6 |  | 3,735 | 43.5 |  | 700 | 22.1 |  | 137 | 1.5 |
| South Dakota | 2018 | 359 | 75.8 |  | 414 | 17.6 |  | 4,467 | 45.4 |  | 690 | 19.6 |  | 152 | 0.7 |
| South Dakota | 2019 | 427 | 62.8 |  | 454 | 15.4 |  | 5,060 | 44.7 |  | 674 | 19.9 |  | 138 | 5.1 |
| South Dakota | 2020 | 290 | 67.2 |  | 353 | 15.6 |  | 4,350 | 43.9 |  | 478 | 20.7 |  | 107 | 2.8 |
| Tennessee | 2000 | 0 |  |  | 2,447 | 2.8 |  | 237 | 13.9 |  | 592 | 34.3 |  | 306 | 2.3 |
| Tennessee | 2001 | 0 |  |  | 3,503 | 4.6 |  | 320 | 14.1 |  | 1,054 | 36.1 |  | 472 | 4.7 |
| Tennessee | 2002 | 0 |  |  | 3,544 | 4.4 |  | 447 | 17.9 |  | 1,391 | 33.1 |  | 519 | 2.7 |
| Tennessee | 2003 | 0 |  |  | 3,923 | 4.7 |  | 551 | 12.7 |  | 1,721 | 32.8 |  | 564 | 1.8 |
| Tennessee | 2004 | 0 |  |  | 3,885 | 4.9 |  | 793 | 20.3 |  | 1,749 | 33.6 |  | 608 | 4.8 |
| Tennessee | 2005 | 0 |  |  | 3,950 | 4.2 |  | 734 | 16.5 |  | 2,003 | 33.4 |  | 705 | 3.7 |
| Tennessee | 2006 | 0 |  |  | 3,882 | 5.5 |  | 626 | 17.7 |  | 2,060 | 35.0 |  | 724 | 3.5 |
| Tennessee | 2007 | 0 |  |  | 3,481 | 6.4 |  | 568 | 12.3 |  | 2,334 | 27.5 |  | 900 | 9.4 |
| Tennessee | 2008 | 0 |  |  | 2,955 | 5.4 |  | 530 | 16.4 |  | 2,482 | 29.0 |  | 840 | 4.4 |
| Tennessee | 2009 | 150 | 84.0 |  | 2,808 | 7.0 |  | 694 | 25.9 |  | 3,119 | 32.0 |  | 1,346 | 6.6 |
| Tennessee | 2010 | 281 | 80.1 |  | 2,953 | 7.1 |  | 968 | 34.5 |  | 4,477 | 33.3 |  | 2,033 | 3.8 |
| Tennessee | 2011 | 344 | 80.8 |  | 3,277 | 7.8 |  | 1,549 | 35.2 |  | 5,282 | 34.1 |  | 2,251 | 3.4 |
| Tennessee | 2012 | 517 | 77.0 |  | 3,069 | 10.3 |  | 1,531 | 40.0 |  | 5,710 | 38.4 |  | 2,420 | 4.7 |
| Tennessee | 2013 | 719 | 84.7 |  | 3,015 | 8.7 |  | 1,812 | 46.2 |  | 6,094 | 42.3 |  | 2,291 | 5.6 |
| Tennessee | 2014 | 973 | 83.0 |  | 3,042 | 10.1 |  | 2,099 | 47.8 |  | 6,347 | 42.0 |  | 2,375 | 4.2 |
| Tennessee | 2015 | 1,418 | 80.5 |  | 2,911 | 10.4 |  | 2,623 | 50.8 |  | 6,305 | 39.9 |  | 2,369 | 5.3 |
| Tennessee | 2016 | 2,094 | 79.2 |  | 3,068 | 11.4 |  | 3,705 | 53.7 |  | 6,381 | 38.0 |  | 2,380 | 4.5 |
| Tennessee | 2017 | 2,719 | 76.0 |  | 3,682 | 10.2 |  | 5,035 | 52.8 |  | 6,663 | 34.3 |  | 2,312 | 4.0 |
| Tennessee | 2018 | 3,578 | 70.8 |  | 3,826 | 9.6 |  | 7,013 | 54.1 |  | 7,114 | 35.4 |  | 2,363 | 3.9 |
| Tennessee | 2019 | 4,192 | 68.1 |  | 3,393 | 8.6 |  | 7,758 | 50.4 |  | 6,344 | 30.5 |  | 2,108 | 3.4 |
| Tennessee | 2020 | 3,955 | 66.0 |  | 2,593 | 6.8 |  | 6,772 | 49.2 |  | 4,791 | 27.5 |  | 1,619 | 3.4 |
| Texas | 2000 | 3,961 | 88.6 |  | 12,789 | 17.1 |  | 2,410 | 55.7 |  | 1,177 | 11.9 |  | 1,079 | 2.1 |
| Texas | 2001 | 3,917 | 87.9 |  | 12,785 | 15.9 |  | 3,023 | 54.4 |  | 1,715 | 10.5 |  | 1,238 | 2.7 |
| Texas | 2002 | 4,499 | 89.1 |  | 14,948 | 15.4 |  | 3,543 | 52.0 |  | 2,239 | 10.2 |  | 1,291 | 2.2 |
| Texas | 2003 | 3,900 | 89.6 |  | 13,985 | 14.5 |  | 4,110 | 50.1 |  | 2,448 | 10.6 |  | 1,350 | 1.0 |
| Texas | 2004 | 4,072 | 87.5 |  | 14,162 | 13.4 |  | 4,946 | 44.0 |  | 3,044 | 11.0 |  | 1,428 | 1.0 |
| Texas | 2005 | 4,255 | 84.9 |  | 14,967 | 11.7 |  | 7,416 | 36.0 |  | 3,178 | 9.5 |  | 1,690 | 1.2 |
| Texas | 2006 | 4,163 | 85.3 |  | 15,922 | 11.8 |  | 7,174 | 35.3 |  | 3,640 | 9.3 |  | 2,033 | 0.5 |
| Texas | 2007 | 4,295 | 86.5 |  | 16,661 | 11.5 |  | 6,746 | 35.1 |  | 4,310 | 9.5 |  | 2,593 | 0.7 |
| Texas | 2008 | 4,827 | 84.3 |  | 16,081 | 10.0 |  | 5,717 | 35.4 |  | 4,918 | 8.2 |  | 2,508 | 0.8 |
| Texas | 2009 | 5,598 | 85.4 |  | 13,713 | 9.8 |  | 5,765 | 37.5 |  | 5,594 | 8.1 |  | 2,973 | 1.2 |
| Texas | 2010 | 5,199 | 81.6 |  | 11,245 | 10.1 |  | 5,623 | 35.1 |  | 5,050 | 9.7 |  | 3,155 | 0.4 |
| Texas | 2011 | 6,641 | 78.3 |  | 11,282 | 9.9 |  | 6,077 | 31.6 |  | 5,502 | 8.8 |  | 3,394 | 0.2 |
| Texas | 2012 | 6,310 | 77.9 |  | 10,347 | 9.4 |  | 7,214 | 33.7 |  | 5,444 | 10.2 |  | 3,474 | 0.5 |
| Texas | 2013 | 6,668 | 78.0 |  | 9,115 | 9.0 |  | 8,533 | 33.0 |  | 4,804 | 8.3 |  | 3,088 | 0.3 |
| Texas | 2014 | 6,615 | 79.5 |  | 8,307 | 8.9 |  | 9,504 | 32.8 |  | 4,266 | 8.7 |  | 3,049 | 0.7 |
| Texas | 2015 | 7,333 | 80.2 |  | 7,600 | 9.7 |  | 9,885 | 32.5 |  | 3,743 | 10.9 |  | 3,170 | 0.4 |
| Texas | 2016 | 6,789 | 83.3 |  | 6,816 | 8.9 |  | 10,454 | 31.8 |  | 3,395 | 10.2 |  | 3,000 | 0.4 |
| Texas | 2017 | 6,784 | 83.3 |  | 6,972 | 7.8 |  | 11,126 | 29.5 |  | 3,026 | 9.7 |  | 3,009 | 0.3 |
| Texas | 2018 | 6,597 | 82.6 |  | 6,836 | 7.9 |  | 10,812 | 27.7 |  | 2,880 | 9.9 |  | 2,558 | 0.2 |
| Texas | 2019 | 6,833 | 80.3 |  | 5,829 | 6.9 |  | 11,630 | 25.4 |  | 2,621 | 9.3 |  | 2,297 | 0.4 |
| Texas | 2020 | 6,612 | 78.2 |  | 4,949 | 6.4 |  | 10,737 | 23.6 |  | 2,444 | 9.4 |  | 2,125 | 0.3 |
| Utah | 2000 | 2,056 | 84.4 |  | 4,010 | 31.1 |  | 4,678 | 20.6 |  | 731 | 6.3 |  | 449 | 2.2 |
| Utah | 2001 | 1,604 | 83.2 |  | 2,913 | 32.3 |  | 3,999 | 23.0 |  | 685 | 5.8 |  | 357 | 2.2 |
| Utah | 2002 | 1,158 | 85.1 |  | 2,002 | 31.2 |  | 2,978 | 23.6 |  | 588 | 5.1 |  | 262 | 1.5 |
| Utah | 2003 | 1,366 | 79.6 |  | 2,317 | 29.7 |  | 4,312 | 23.9 |  | 820 | 6.3 |  | 286 | 2.8 |
| Utah | 2004 | 1,345 | 78.3 |  | 2,187 | 29.2 |  | 4,462 | 25.2 |  | 1,053 | 7.5 |  | 335 | 1.2 |
| Utah | 2005 | 1,580 | 74.2 |  | 2,282 | 26.7 |  | 4,366 | 27.8 |  | 979 | 7.6 |  | 293 | 2.7 |
| Utah | 2006 | 1,885 | 72.1 |  | 2,851 | 27.8 |  | 5,100 | 26.8 |  | 1,247 | 6.2 |  | 315 | 2.2 |
| Utah | 2007 | 2,223 | 73.0 |  | 3,232 | 28.1 |  | 4,778 | 26.1 |  | 1,364 | 4.9 |  | 306 | 1.0 |
| Utah | 2008 | 2,358 | 69.1 |  | 3,239 | 25.4 |  | 4,090 | 26.9 |  | 1,511 | 6.1 |  | 257 | 1.6 |
| Utah | 2009 | 2,378 | 68.3 |  | 2,638 | 25.0 |  | 3,625 | 27.2 |  | 1,690 | 6.4 |  | 363 | 1.1 |
| Utah | 2010 | 2,552 | 70.4 |  | 2,409 | 25.9 |  | 3,758 | 28.3 |  | 1,742 | 5.2 |  | 506 | 0.8 |
| Utah | 2011 | 2,651 | 69.6 |  | 2,159 | 27.5 |  | 3,949 | 28.3 |  | 1,649 | 5.9 |  | 566 | 2.7 |
| Utah | 2012 | 2,686 | 66.6 |  | 1,865 | 26.2 |  | 4,026 | 29.9 |  | 1,633 | 7.5 |  | 549 | 1.8 |
| Utah | 2013 | 3,011 | 63.4 |  | 1,644 | 20.4 |  | 4,394 | 28.6 |  | 1,579 | 5.7 |  | 477 | 2.7 |
| Utah | 2014 | 2,992 | 61.0 |  | 1,340 | 24.6 |  | 4,284 | 31.1 |  | 1,318 | 6.2 |  | 397 | 2.3 |
| Utah | 2015 | 3,390 | 61.9 |  | 1,333 | 22.4 |  | 4,956 | 35.1 |  | 1,384 | 6.4 |  | 425 | 1.2 |
| Utah | 2016 | 3,825 | 63.0 |  | 1,193 | 19.3 |  | 5,368 | 36.3 |  | 1,297 | 5.6 |  | 408 | 1.2 |
| Utah | 2017 | 4,329 | 63.1 |  | 1,298 | 18.4 |  | 5,917 | 39.0 |  | 1,112 | 7.1 |  | 402 | 1.2 |
| Utah | 2018 | 5,643 | 63.1 |  | 1,308 | 19.3 |  | 8,201 | 38.2 |  | 1,446 | 7.1 |  | 519 | 2.3 |
| Utah | 2019 | 5,637 | 60.6 |  | 1,096 | 14.6 |  | 9,160 | 36.3 |  | 1,365 | 5.5 |  | 492 | 2.6 |
| Utah | 2020 | 3,771 | 58.3 |  | 652 | 12.9 |  | 6,245 | 33.9 |  | 857 | 7.0 |  | 337 | 2.4 |
| Vermont | 2000 | 531 | 73.6 |  | 838 | 12.1 |  | 76 | 7.9 |  | 314 | 13.1 |  | 125 | 0.8 |
| Vermont | 2001 | 670 | 70.9 |  | 836 | 13.4 |  | 59 | 8.5 |  | 442 | 9.5 |  | 128 | 2.3 |
| Vermont | 2002 | 1,098 | 77.9 |  | 1,116 | 17.8 |  | 75 | 6.7 |  | 664 | 12.7 |  | 149 | 1.3 |
| Vermont | 2003 | 990 | 77.2 |  | 1,119 | 14.9 |  | 73 | 17.8 |  | 958 | 15.7 |  | 174 | 2.9 |
| Vermont | 2004 | 723 | 75.8 |  | 988 | 18.2 |  | 57 | 7.0 |  | 937 | 20.6 |  | 118 | 3.4 |
| Vermont | 2005 | 1,092 | 79.1 |  | 1,714 | 20.9 |  | 97 | 10.3 |  | 1,790 | 22.5 |  | 224 | 0.9 |
| Vermont | 2006 | 802 | 73.6 |  | 1,960 | 18.4 |  | 75 | 12.0 |  | 1,996 | 24.8 |  | 209 | 1.4 |
| Vermont | 2007 | 681 | 70.0 |  | 1,850 | 20.6 |  | 110 | 14.5 |  | 2,147 | 19.7 |  | 267 | 1.9 |
| Vermont | 2008 | 787 | 69.8 |  | 1,766 | 20.0 |  | 93 | 12.9 |  | 2,545 | 21.1 |  | 304 | 1.3 |
| Vermont | 2009 | 520 | 73.7 |  | 948 | 15.8 |  | 66 | 10.6 |  | 1,865 | 19.4 |  | 170 | 0.6 |
| Vermont | 2010 | 484 | 73.1 |  | 981 | 14.1 |  | 84 | 14.3 |  | 2,246 | 21.0 |  | 169 | 4.1 |
| Vermont | 2011 | 765 | 72.8 |  | 1,067 | 12.8 |  | 68 | 14.7 |  | 2,698 | 22.1 |  | 171 | 1.8 |
| Vermont | 2012 | 1,420 | 72.3 |  | 1,455 | 20.6 |  | 163 | 17.8 |  | 3,247 | 24.8 |  | 355 | 3.4 |
| Vermont | 2013 | 2,489 | 74.6 |  | 1,473 | 22.6 |  | 191 | 24.1 |  | 3,502 | 22.0 |  | 379 | 2.1 |
| Vermont | 2014 | 3,721 | 72.6 |  | 1,907 | 24.1 |  | 264 | 22.3 |  | 4,075 | 22.9 |  | 476 | 2.7 |
| Vermont | 2015 | 3,875 | 69.7 |  | 1,993 | 23.9 |  | 281 | 21.4 |  | 3,330 | 20.2 |  | 379 | 4.5 |
| Vermont | 2016 | 4,271 | 71.9 |  | 2,121 | 22.4 |  | 264 | 28.8 |  | 3,170 | 19.4 |  | 389 | 2.1 |
| Vermont | 2017 | 3,758 | 72.1 |  | 2,321 | 22.2 |  | 269 | 24.2 |  | 2,636 | 17.6 |  | 337 | 2.4 |
| Vermont | 2018 | 3,188 | 67.6 |  | 2,622 | 19.8 |  | 307 | 25.4 |  | 2,411 | 17.8 |  | 313 | 2.6 |
| Vermont | 2019 | 2,706 | 65.9 |  | 2,376 | 17.8 |  | 330 | 30.9 |  | 1,872 | 14.9 |  | 224 | 4.9 |
| Vermont | 2020 | 1,825 | 62.6 |  | 1,420 | 16.4 |  | 221 | 32.6 |  | 959 | 13.0 |  | 120 | 0.8 |
| Virginia | 2000 | 1,789 | 24.0 |  | 6,549 | 8.4 |  | 503 | 8.7 |  | 1,231 | 25.1 |  | 800 | 3.1 |
| Virginia | 2001 | 1,793 | 28.1 |  | 6,691 | 8.2 |  | 607 | 6.1 |  | 1,601 | 26.3 |  | 1,077 | 3.2 |
| Virginia | 2002 | 2,550 | 39.6 |  | 9,347 | 7.0 |  | 828 | 6.3 |  | 1,915 | 18.4 |  | 1,138 | 4.7 |
| Virginia | 2003 | 4,767 | 51.7 |  | 16,230 | 4.8 |  | 1,493 | 11.3 |  | 3,410 | 14.0 |  | 1,910 | 3.6 |
| Virginia | 2004 | 6,033 | 55.3 |  | 21,008 | 4.8 |  | 1,798 | 12.5 |  | 4,613 | 15.7 |  | 2,379 | 2.2 |
| Virginia | 2005 | 3,337 | 58.7 |  | 12,909 | 4.9 |  | 1,498 | 11.5 |  | 3,013 | 13.3 |  | 1,390 | 2.7 |
| Virginia | 2006 | 2,820 | 53.5 |  | 11,411 | 4.2 |  | 1,190 | 8.8 |  | 2,632 | 15.0 |  | 1,138 | 1.4 |
| Virginia | 2007 | 2,585 | 59.1 |  | 10,272 | 4.5 |  | 1,008 | 9.7 |  | 2,689 | 13.2 |  | 1,060 | 1.0 |
| Virginia | 2008 | 2,942 | 59.5 |  | 10,169 | 3.8 |  | 905 | 11.5 |  | 3,137 | 12.0 |  | 1,177 | 0.9 |
| Virginia | 2009 | 3,093 | 61.3 |  | 8,472 | 4.9 |  | 959 | 13.3 |  | 3,842 | 12.7 |  | 1,396 | 1.6 |
| Virginia | 2010 | 2,657 | 63.3 |  | 7,055 | 5.2 |  | 867 | 11.3 |  | 4,420 | 16.0 |  | 1,538 | 1.0 |
| Virginia | 2011 | 2,718 | 65.3 |  | 7,061 | 6.2 |  | 1,033 | 12.6 |  | 4,737 | 18.4 |  | 1,728 | 1.2 |
| Virginia | 2012 | 2,992 | 69.1 |  | 6,168 | 7.1 |  | 1,116 | 17.2 |  | 4,756 | 21.7 |  | 1,729 | 2.0 |
| Virginia | 2013 | 2,983 | 70.0 |  | 5,135 | 7.3 |  | 1,096 | 18.5 |  | 4,293 | 22.6 |  | 1,594 | 2.1 |
| Virginia | 2014 | 3,338 | 71.0 |  | 5,027 | 7.1 |  | 1,154 | 16.8 |  | 4,489 | 23.3 |  | 1,526 | 1.7 |
| Virginia | 2015 | 3,772 | 69.0 |  | 4,708 | 7.4 |  | 1,522 | 23.3 |  | 4,579 | 23.7 |  | 1,518 | 1.8 |
| Virginia | 2016 | 4,137 | 66.0 |  | 5,054 | 9.7 |  | 1,968 | 27.4 |  | 4,583 | 21.3 |  | 1,474 | 2.4 |
| Virginia | 2017 | 4,710 | 63.8 |  | 5,699 | 10.1 |  | 2,638 | 31.4 |  | 4,689 | 19.4 |  | 1,448 | 2.3 |
| Virginia | 2018 | 5,172 | 59.1 |  | 6,278 | 8.7 |  | 3,777 | 29.7 |  | 4,659 | 16.9 |  | 1,399 | 1.9 |
| Virginia | 2019 | 5,439 | 56.6 |  | 6,561 | 7.7 |  | 4,643 | 29.5 |  | 4,718 | 15.7 |  | 1,241 | 1.5 |
| Virginia | 2020 | 3,896 | 56.3 |  | 4,525 | 7.9 |  | 4,407 | 29.6 |  | 3,742 | 15.7 |  | 888 | 1.8 |
| Washington | 2000 | 3,548 | 91.1 |  | 7,463 | 24.5 |  | 6,268 | 27.9 |  | 1,140 | 4.8 |  | 645 | 1.7 |
| Washington | 2001 | 3,215 | 91.5 |  | 6,771 | 22.1 |  | 6,781 | 27.4 |  | 1,205 | 4.4 |  | 762 | 2.0 |
| Washington | 2002 | 3,047 | 89.9 |  | 5,964 | 21.2 |  | 6,269 | 27.1 |  | 1,384 | 5.1 |  | 767 | 2.1 |
| Washington | 2003 | 2,940 | 89.1 |  | 6,230 | 18.4 |  | 6,837 | 26.5 |  | 1,662 | 4.5 |  | 762 | 0.5 |
| Washington | 2004 | 3,483 | 89.1 |  | 6,986 | 17.7 |  | 7,802 | 24.7 |  | 2,347 | 4.2 |  | 874 | 1.9 |
| Washington | 2005 | 3,951 | 87.5 |  | 7,944 | 14.7 |  | 9,748 | 22.7 |  | 2,832 | 4.2 |  | 1,000 | 1.8 |
| Washington | 2006 | 3,659 | 85.8 |  | 8,792 | 13.8 |  | 10,750 | 21.9 |  | 2,432 | 6.1 |  | 1,046 | 2.0 |
| Washington | 2007 | 3,527 | 84.3 |  | 8,645 | 11.9 |  | 10,567 | 21.9 |  | 2,922 | 5.0 |  | 959 | 2.6 |
| Washington | 2008 | 4,225 | 84.5 |  | 8,851 | 11.4 |  | 9,748 | 21.4 |  | 4,797 | 4.7 |  | 1,073 | 1.8 |
| Washington | 2009 | 4,267 | 80.6 |  | 7,535 | 10.6 |  | 9,018 | 22.0 |  | 5,494 | 5.2 |  | 1,117 | 2.1 |
| Washington | 2010 | 4,929 | 79.8 |  | 6,389 | 10.4 |  | 9,117 | 23.6 |  | 5,896 | 5.3 |  | 1,186 | 1.7 |
| Washington | 2011 | 5,965 | 75.5 |  | 5,509 | 10.1 |  | 9,269 | 24.1 |  | 5,576 | 5.2 |  | 1,231 | 1.6 |
| Washington | 2012 | 6,484 | 74.6 |  | 4,592 | 9.8 |  | 9,354 | 26.2 |  | 5,501 | 5.6 |  | 1,194 | 1.5 |
| Washington | 2013 | 6,902 | 73.2 |  | 3,771 | 10.2 |  | 10,388 | 28.4 |  | 5,299 | 5.5 |  | 1,176 | 1.7 |
| Washington | 2014 | 9,176 | 69.3 |  | 3,518 | 10.4 |  | 12,048 | 28.4 |  | 5,495 | 5.8 |  | 1,270 | 2.0 |
| Washington | 2015 | 10,096 | 65.0 |  | 3,033 | 10.3 |  | 12,888 | 29.5 |  | 5,133 | 5.5 |  | 1,297 | 2.1 |
| Washington | 2016 | 28,691 | 65.2 |  | 5,979 | 9.6 |  | 36,326 | 31.8 |  | 12,529 | 6.9 |  | 2,460 | 2.1 |
| Washington | 2017 | 16,879 | 61.0 |  | 3,776 | 9.9 |  | 27,445 | 34.5 |  | 6,383 | 8.1 |  | 1,863 | 2.4 |
| Washington | 2018 | Data not reported | |  |  |  |  |  |  |  |  |  |  |  |  |
| Washington | 2019 | Data not reported | |  |  |  |  |  |  |  |  |  |  |  |  |
| Washington | 2020 | Data not reported | |  |  |  |  |  |  |  |  |  |  |  |  |
| West Virginia | 2000 | Data not reported | |  |  |  |  |  |  |  |  |  |  |  |  |
| West Virginia | 2001 | Data not reported | |  |  |  |  |  |  |  |  |  |  |  |  |
| West Virginia | 2002 | Data not reported | |  |  |  |  |  |  |  |  |  |  |  |  |
| West Virginia | 2003 | 1 | 100.0 |  | 12 | 0.0 |  | 3 | 0.0 |  | 8 | 25.0 |  | 3 | 33.3 |
| West Virginia | 2004 | 23 | 78.3 |  | 67 | 9.0 |  | 12 | 16.7 |  | 45 | 24.4 |  | 20 | 0.0 |
| West Virginia | 2005 | 244 | 78.7 |  | 814 | 14.3 |  | 210 | 15.7 |  | 1,085 | 22.9 |  | 394 | 3.6 |
| West Virginia | 2006 | 382 | 78.3 |  | 1,908 | 11.7 |  | 363 | 16.0 |  | 2,100 | 23.5 |  | 755 | 2.8 |
| West Virginia | 2007 | 266 | 82.7 |  | 1,433 | 11.8 |  | 296 | 19.6 |  | 2,176 | 18.8 |  | 693 | 0.9 |
| West Virginia | 2008 | 388 | 83.8 |  | 1,237 | 10.8 |  | 321 | 15.6 |  | 3,319 | 19.8 |  | 872 | 2.5 |
| West Virginia | 2009 | 383 | 85.4 |  | 732 | 12.7 |  | 226 | 16.8 |  | 2,953 | 25.3 |  | 832 | 2.4 |
| West Virginia | 2010 | 244 | 84.0 |  | 374 | 16.0 |  | 166 | 23.5 |  | 1,614 | 27.4 |  | 555 | 2.7 |
| West Virginia | 2011 | 296 | 82.4 |  | 262 | 13.7 |  | 172 | 26.7 |  | 1,344 | 25.1 |  | 434 | 1.4 |
| West Virginia | 2012 | 208 | 85.6 |  | 153 | 15.7 |  | 120 | 33.3 |  | 834 | 27.1 |  | 194 | 1.0 |
| West Virginia | 2013 | 486 | 80.2 |  | 204 | 20.1 |  | 181 | 30.9 |  | 1,083 | 28.4 |  | 146 | 2.1 |
| West Virginia | 2014 | 695 | 77.7 |  | 269 | 17.1 |  | 253 | 38.3 |  | 1,371 | 26.7 |  | 184 | 3.8 |
| West Virginia | 2015 | 540 | 76.1 |  | 242 | 18.6 |  | 225 | 37.3 |  | 1,020 | 28.2 |  | 154 | 0.6 |
| West Virginia | 2016 | 1,502 | 79.4 |  | 589 | 23.8 |  | 756 | 44.3 |  | 2,240 | 30.7 |  | 729 | 3.4 |
| West Virginia | 2017 | 1,068 | 78.0 |  | 465 | 23.9 |  | 886 | 46.0 |  | 1,677 | 30.1 |  | 450 | 2.7 |
| West Virginia | 2018 | 563 | 69.6 |  | 219 | 17.8 |  | 581 | 45.4 |  | 848 | 29.7 |  | 172 | 5.2 |
| West Virginia | 2019 | 442 | 65.2 |  | 122 | 22.1 |  | 500 | 41.2 |  | 639 | 28.3 |  | 91 | 4.4 |
| West Virginia | 2020 | 448 | 57.1 |  | 63 | 23.8 |  | 390 | 45.4 |  | 350 | 22.9 |  | 47 | 0.0 |
| Wisconsin | 2000 | 411 | 46.2 |  | 2,851 | 4.3 |  | 212 | 14.2 |  | 303 | 17.2 |  | 133 | 0.0 |
| Wisconsin | 2001 | 548 | 50.0 |  | 3,038 | 4.9 |  | 261 | 7.3 |  | 388 | 12.1 |  | 129 | 0.8 |
| Wisconsin | 2002 | 691 | 51.8 |  | 3,274 | 4.8 |  | 332 | 13.6 |  | 573 | 9.9 |  | 167 | 0.6 |
| Wisconsin | 2003 | 1,027 | 51.6 |  | 4,876 | 3.8 |  | 440 | 11.4 |  | 771 | 10.8 |  | 173 | 1.2 |
| Wisconsin | 2004 | 879 | 56.5 |  | 4,369 | 3.8 |  | 511 | 7.2 |  | 987 | 7.6 |  | 205 | 1.5 |
| Wisconsin | 2005 | 1,208 | 57.0 |  | 5,224 | 4.2 |  | 767 | 9.5 |  | 1,305 | 7.7 |  | 217 | 0.0 |
| Wisconsin | 2006 | 1,350 | 61.3 |  | 7,213 | 4.1 |  | 861 | 4.9 |  | 1,845 | 6.0 |  | 308 | 4.5 |
| Wisconsin | 2007 | 1,329 | 57.6 |  | 6,611 | 4.0 |  | 750 | 9.1 |  | 2,093 | 7.7 |  | 319 | 2.8 |
| Wisconsin | 2008 | 1,350 | 66.1 |  | 5,254 | 3.7 |  | 616 | 9.9 |  | 2,267 | 8.6 |  | 318 | 1.9 |
| Wisconsin | 2009 | 1,791 | 65.3 |  | 5,170 | 3.7 |  | 695 | 5.9 |  | 2,593 | 9.3 |  | 450 | 1.3 |
| Wisconsin | 2010 | 1,916 | 66.2 |  | 4,693 | 3.8 |  | 730 | 8.9 |  | 2,905 | 13.0 |  | 474 | 1.3 |
| Wisconsin | 2011 | 2,340 | 73.1 |  | 4,267 | 5.1 |  | 862 | 12.2 |  | 2,968 | 13.0 |  | 468 | 3.0 |
| Wisconsin | 2012 | 2,662 | 72.8 |  | 4,474 | 4.8 |  | 1,047 | 17.2 |  | 3,366 | 11.4 |  | 501 | 2.0 |
| Wisconsin | 2013 | 3,200 | 75.9 |  | 3,356 | 6.2 |  | 1,008 | 17.1 |  | 3,027 | 13.6 |  | 493 | 0.2 |
| Wisconsin | 2014 | 2,643 | 74.3 |  | 2,196 | 6.4 |  | 991 | 17.9 |  | 2,073 | 11.3 |  | 440 | 1.4 |
| Wisconsin | 2015 | 2,829 | 74.7 |  | 2,251 | 9.1 |  | 1,288 | 23.7 |  | 2,181 | 13.9 |  | 467 | 0.9 |
| Wisconsin | 2016 | 3,127 | 77.2 |  | 1,992 | 10.4 |  | 1,535 | 31.5 |  | 2,061 | 13.6 |  | 371 | 1.6 |
| Wisconsin | 2017 | 4,376 | 74.9 |  | 2,967 | 10.1 |  | 2,060 | 33.2 |  | 2,430 | 11.4 |  | 482 | 1.9 |
| Wisconsin | 2018 | 4,870 | 73.2 |  | 3,872 | 10.0 |  | 2,629 | 39.4 |  | 2,381 | 10.2 |  | 431 | 2.8 |
| Wisconsin | 2019 | 5,264 | 70.8 |  | 4,450 | 10.3 |  | 3,250 | 40.0 |  | 2,462 | 11.9 |  | 513 | 1.2 |
| Wisconsin | 2020 | 3,676 | 68.4 |  | 3,437 | 8.5 |  | 2,410 | 37.8 |  | 1,701 | 11.5 |  | 457 | 2.0 |
| Wyoming | 2000 | 43 | 55.8 |  | 354 | 19.2 |  | 712 | 36.4 |  | 55 | 5.5 |  | 31 | 6.5 |
| Wyoming | 2001 | 42 | 73.8 |  | 410 | 25.6 |  | 1,045 | 33.8 |  | 93 | 4.3 |  | 78 | 3.8 |
| Wyoming | 2002 | 35 | 62.9 |  | 380 | 22.4 |  | 1,159 | 33.5 |  | 119 | 11.8 |  | 80 | 5.0 |
| Wyoming | 2003 | 58 | 63.8 |  | 430 | 25.6 |  | 1,452 | 39.0 |  | 158 | 13.3 |  | 63 | 1.6 |
| Wyoming | 2004 | 46 | 67.4 |  | 383 | 18.0 |  | 1,516 | 33.6 |  | 166 | 9.6 |  | 64 | 3.1 |
| Wyoming | 2005 | 34 | 35.3 |  | 300 | 13.0 |  | 1,706 | 14.8 |  | 171 | 7.6 |  | 58 | 3.4 |
| Wyoming | 2006 | 30 | 10.0 |  | 265 | 5.3 |  | 1,099 | 12.6 |  | 122 | 4.1 |  | 49 | 4.1 |
| Wyoming | 2007 | 41 | 43.9 |  | 300 | 8.3 |  | 930 | 24.3 |  | 175 | 14.9 |  | 72 | 1.4 |
| Wyoming | 2008 | 56 | 82.1 |  | 427 | 17.6 |  | 1,108 | 32.1 |  | 269 | 16.0 |  | 72 | 0.0 |
| Wyoming | 2009 | 98 | 81.6 |  | 404 | 17.6 |  | 1,320 | 34.5 |  | 402 | 19.7 |  | 94 | 4.3 |
| Wyoming | 2010 | 92 | 84.8 |  | 376 | 14.6 |  | 1,318 | 35.2 |  | 450 | 16.7 |  | 115 | 5.2 |
| Wyoming | 2011 | 90 | 75.6 |  | 328 | 14.0 |  | 1,120 | 36.7 |  | 425 | 15.5 |  | 109 | 3.7 |
| Wyoming | 2012 | 85 | 71.8 |  | 155 | 15.5 |  | 809 | 36.2 |  | 393 | 18.6 |  | 94 | 5.3 |
| Wyoming | 2013 | 134 | 64.9 |  | 111 | 21.6 |  | 776 | 30.9 |  | 393 | 16.5 |  | 89 | 6.7 |
| Wyoming | 2014 | 109 | 74.3 |  | 92 | 6.5 |  | 665 | 33.2 |  | 308 | 16.9 |  | 61 | 4.9 |
| Wyoming | 2015 | 84 | 66.7 |  | 59 | 15.3 |  | 664 | 32.5 |  | 223 | 17.0 |  | 43 | 11.6 |
| Wyoming | 2016 | 119 | 73.9 |  | 48 | 2.1 |  | 687 | 31.4 |  | 261 | 16.1 |  | 45 | 6.7 |
| Wyoming | 2017 | 176 | 69.9 |  | 54 | 7.4 |  | 805 | 35.9 |  | 260 | 17.7 |  | 37 | 0.0 |
| Wyoming | 2018 | 200 | 73.5 |  | 62 | 9.7 |  | 828 | 36.1 |  | 285 | 20.7 |  | 59 | 3.4 |
| Wyoming | 2019 | 148 | 60.8 |  | 53 | 15.1 |  | 834 | 30.3 |  | 264 | 12.5 |  | 43 | 0.0 |
| Wyoming | 2020 | 134 | 70.9 |  | 37 | 10.8 |  | 585 | 38.1 |  | 116 | 12.9 |  | 25 | 0.0 |
| United States | 2000 | 295,502 | 60.7 |  | 500,231 | 9.5 |  | 127,792 | 25.9 |  | 52,072 | 11.4 |  | 40,019 | 1.4 |
| United States | 2001 | 303,489 | 59.2 |  | 494,162 | 9.3 |  | 146,700 | 24.8 |  | 67,779 | 11.4 |  | 44,492 | 1.6 |
| United States | 2002 | 314,011 | 59.6 |  | 527,298 | 9.2 |  | 178,707 | 22.8 |  | 83,237 | 11.2 |  | 48,243 | 1.6 |
| United States | 2003 | 299,359 | 58.5 |  | 528,255 | 8.5 |  | 187,043 | 21.1 |  | 91,679 | 11.1 |  | 49,157 | 1.4 |
| United States | 2004 | 281,962 | 60.1 |  | 502,937 | 8.6 |  | 196,000 | 19.8 |  | 99,191 | 10.5 |  | 48,826 | 1.5 |
| United States | 2005 | 317,484 | 61.3 |  | 579,976 | 8.3 |  | 229,415 | 18.7 |  | 127,574 | 10.6 |  | 58,389 | 1.2 |
| United States | 2006 | 314,830 | 61.2 |  | 601,428 | 8.3 |  | 227,534 | 18.1 |  | 145,081 | 10.1 |  | 63,219 | 1.1 |
| United States | 2007 | 310,236 | 61.7 |  | 584,288 | 8.3 |  | 218,190 | 18.1 |  | 168,197 | 9.4 |  | 70,613 | 1.1 |
| United States | 2008 | 330,359 | 62.3 |  | 554,537 | 8.0 |  | 199,369 | 19.2 |  | 205,186 | 10.1 |  | 81,299 | 1.1 |
| United States | 2009 | 334,268 | 64.2 |  | 473,981 | 8.3 |  | 187,054 | 20.4 |  | 234,634 | 11.5 |  | 93,086 | 1.3 |
| United States | 2010 | 324,470 | 66.7 |  | 422,594 | 9.0 |  | 187,291 | 20.9 |  | 269,292 | 13.0 |  | 105,560 | 1.3 |
| United States | 2011 | 341,722 | 67.2 |  | 413,920 | 9.7 |  | 186,744 | 22.0 |  | 305,517 | 14.5 |  | 120,807 | 1.6 |
| United States | 2012 | 359,845 | 68.5 |  | 364,874 | 10.6 |  | 201,444 | 24.0 |  | 291,352 | 15.2 |  | 115,668 | 1.7 |
| United States | 2013 | 399,164 | 69.4 |  | 323,746 | 11.3 |  | 222,326 | 25.4 |  | 270,726 | 15.8 |  | 109,357 | 1.7 |
| United States | 2014 | 424,088 | 69.4 |  | 288,487 | 11.8 |  | 233,326 | 26.1 |  | 234,974 | 15.3 |  | 103,511 | 1.5 |
| United States | 2015 | 493,121 | 66.5 |  | 293,124 | 12.2 |  | 244,399 | 26.6 |  | 236,067 | 14.4 |  | 107,428 | 1.5 |
| United States | 2016 | 525,803 | 66.8 |  | 315,935 | 13.3 |  | 310,364 | 28.7 |  | 246,448 | 14.5 |  | 117,589 | 1.3 |
| United States | 2017 | 583,026 | 63.4 |  | 361,825 | 13.4 |  | 342,755 | 29.3 |  | 248,094 | 13.7 |  | 120,775 | 1.4 |
| United States | 2018 | 591,823 | 61.0 |  | 388,739 | 12.6 |  | 367,420 | 29.2 |  | 242,135 | 13.9 |  | 112,390 | 1.3 |
| United States | 2019 | 512,309 | 57.9 |  | 355,415 | 11.7 |  | 358,711 | 29.3 |  | 197,070 | 13.4 |  | 95,831 | 1.2 |
| United States | 2020 | 347,282 | 58.3 |  | 238,723 | 11.3 |  | 292,585 | 28.9 |  | 143,502 | 15.0 |  | 70,871 | 1.2 |
