# Supplementary material for: Estimated Number of Injection-Involved Overdose Deaths in US States From 2000 to 2020: Secondary Analysis of Surveillance Data
Source: JMIR Public Health Surveill. 2024 Apr 5;10:e49527. doi: 10.2196/49527 (PMC11031697; doi:10.2196/49527)
Supplement: Multimedia Appendix 2 [file publichealth_v10i1e49527_app2.docx]

**Supplemental Table 2 Notes**

Data on the annual number of overdose deaths were from the National Vital Statistics System detailed multiple cause of death mortality microdata files^1^. Overdose deaths were identified using the following ICD-10 codes: X40-X44, X60-X64, X85, Y10-Y14^2,3^. Overdose deaths that included toxicology data were categorized into five mutually exclusive drug categories by the following specific multiple-cause-of-death codes: heroin/synthetic opioids other than methadone (T40.1, T40.4), natural or semi-synthetic opioids, and methadone (T40.2, T40.3), cocaine (T40.5), psychostimulants with abuse potential (T43.6), sedatives (T42.3, T42.4) and other (T36-T59.0). Deaths that indicated multiple drugs were categorized based on the drug that had the highest overall percent of injection as estimated from Treatment Episode Data Set - Admissions (TEDS-A) data^4^. Overdose deaths that did not have a specific T-code listed (i.e. only listed T50.9) were distributed to the six categories (five defined drug use categories descried above, and any other T-codes) based on the non-missing distribution within each year and demographic strata^5,6^.

**References**

1. National Center for Health Statistics (NCHS). Data from: Multiple Cause of Death Mortality Microdata (1999-2020), as compiled from data provided by the 57 vital statistics jurisdictions through the Vital Statistics Cooperative Program. 2022.

2. Hedegaard H, Miniño AM, Warner M. Drug Overdose Deaths in the United States, 1999-2019. *NCHS Data Brief*. Dec 2020;(394):1-8.

3. Statistics NCfH. Guide to State Implementation of ICD-10 for Mortality. Part II: Applying Comparability Ratios. *Guide to State Implementation of ICD-10 for Mortality Part II: Applying Comparability Ratios/AHIMA, American Health Information Management Association*. 2000;

4. Hall EW, Rosenberg ES, Jones CM, Asher A, Valverde E, Bradley H. Estimated number of injection-involved drug overdose deaths, United States, 2000 – 2018. *Drug Alcohol Depend*. 2022/05/01/ 2022;234:109428. doi:<https://doi.org/10.1016/j.drugalcdep.2022.109428>

5. Boslett AJ, Denham A, Hill EL. Using contributing causes of death improves prediction of opioid involvement in unclassified drug overdoses in US death records. *Addiction*. Jul 2020;115(7):1308-1317. doi:10.1111/add.14943

6. Ruhm CJ. Drug involvement in fatal overdoses. *SSM Popul Health*. Dec 2017;3:219-226. doi:10.1016/j.ssmph.2017.01.009

Supplemental Table 2. Percent of overdose deaths among adults attributed to each drug type, by state and year, United States, 2000-2020.

| **State** | **Year** | **Heroin/Synthetic opioids (%)** | **Cocaine (%)** | **Stimulants (%)** | **Natural/semi-synthetic opioids/methadone (%)** | **Sedatives (%)** | **Other (%)** |
| --- | --- | --- | --- | --- | --- | --- | --- |
| Alabama | 2000 | 11.5 | 12.9 | 2.1 | 36.7 | 3.0 | 33.8 |
| Alabama | 2001 | 6.9 | 9.8 | 1.0 | 50.7 | 2.0 | 28.7 |
| Alabama | 2002 | 9.6 | 13.7 | 3.0 | 49.9 | 3.7 | 20.1 |
| Alabama | 2003 | 7.1 | 9.7 | 6.8 | 48.4 | 3.1 | 24.9 |
| Alabama | 2004 | 9.4 | 10.3 | 6.8 | 47.6 | 2.1 | 23.8 |
| Alabama | 2005 | 9.3 | 15.2 | 6.8 | 45.7 | 0.8 | 21.1 |
| Alabama | 2006 | 13.9 | 13.6 | 2.6 | 52.7 | 3.8 | 13.1 |
| Alabama | 2007 | 11.1 | 6.5 | 3.0 | 58.1 | 2.7 | 18.4 |
| Alabama | 2008 | 9.5 | 9.7 | 4.8 | 58.3 | 3.2 | 14.1 |
| Alabama | 2009 | 16.0 | 5.4 | 2.7 | 58.5 | 3.2 | 14.0 |
| Alabama | 2010 | 16.3 | 7.2 | 8.9 | 46.8 | 2.0 | 18.6 |
| Alabama | 2011 | 14.8 | 6.6 | 4.4 | 57.0 | 2.8 | 14.4 |
| Alabama | 2012 | 22.5 | 2.6 | 6.1 | 45.9 | 5.9 | 16.5 |
| Alabama | 2013 | 29.0 | 5.0 | 8.5 | 36.0 | 6.2 | 15.0 |
| Alabama | 2014 | 46.9 | 3.4 | 5.1 | 31.3 | 3.4 | 9.5 |
| Alabama | 2015 | 44.9 | 3.8 | 9.2 | 26.8 | 2.7 | 12.7 |
| Alabama | 2016 | 53.1 | 5.8 | 9.8 | 21.2 | 2.3 | 7.8 |
| Alabama | 2017 | 48.7 | 5.7 | 12.9 | 19.3 | 3.3 | 10.1 |
| Alabama | 2018 | 43.6 | 5.7 | 17.5 | 18.1 | 2.8 | 12.4 |
| Alabama | 2019 | 47.4 | 4.4 | 23.7 | 14.4 | 1.5 | 8.2 |
| Alabama | 2020 | 59.0 | 6.3 | 17.3 | 9.2 | 1.3 | 7.0 |
| Alaska | 2000 | 4.3 | 17.0 | 0.0 | 56.9 | 13.8 | 8.0 |
| Alaska | 2001 | 17.6 | 22.7 | 0.0 | 22.2 | 9.7 | 16.4 |
| Alaska | 2002 | 10.3 | 35.5 | 0.0 | 22.2 | 3.0 | 8.7 |
| Alaska | 2003 | 0.0 | 26.3 | 3.3 | 33.2 | 15.8 | 9.0 |
| Alaska | 2004 | 0.0 | 13.3 | 0.0 | 21.6 | 3.7 | 47.8 |
| Alaska | 2005 | 7.4 | 32.3 | 0.0 | 45.0 | 1.6 | 11.0 |
| Alaska | 2006 | 2.3 | 16.0 | 2.9 | 55.4 | 5.5 | 18.0 |
| Alaska | 2007 | 12.9 | 20.3 | 7.6 | 48.6 | 0.0 | 2.3 |
| Alaska | 2008 | 17.8 | 7.8 | 4.4 | 52.8 | 6.2 | 10.1 |
| Alaska | 2009 | 24.0 | 6.9 | 10.8 | 41.1 | 3.3 | 14.0 |
| Alaska | 2010 | 17.5 | 9.8 | 7.9 | 57.2 | 1.2 | 6.3 |
| Alaska | 2011 | 17.8 | 8.0 | 18.0 | 48.3 | 1.6 | 6.4 |
| Alaska | 2012 | 27.2 | 11.4 | 15.7 | 26.3 | 7.9 | 11.5 |
| Alaska | 2013 | 36.1 | 2.0 | 19.7 | 23.5 | 2.9 | 14.8 |
| Alaska | 2014 | 34.4 | 6.3 | 16.9 | 29.0 | 3.6 | 9.9 |
| Alaska | 2015 | 44.6 | 4.4 | 11.4 | 30.9 | 2.6 | 6.2 |
| Alaska | 2016 | 43.8 | 1.6 | 20.0 | 26.6 | 1.6 | 5.6 |
| Alaska | 2017 | 45.4 | 5.0 | 23.8 | 19.3 | 4.3 | 2.2 |
| Alaska | 2018 | 38.0 | 4.6 | 28.7 | 15.7 | 6.5 | 6.5 |
| Alaska | 2019 | 48.5 | 2.4 | 21.6 | 13.6 | 1.7 | 11.5 |
| Alaska | 2020 | 61.2 | 1.9 | 23.8 | 7.1 | 1.4 | 4.6 |
| Arizona | 2000 | 8.7 | 25.8 | 6.8 | 25.4 | 4.7 | 28.4 |
| Arizona | 2001 | 10.6 | 20.4 | 12.5 | 23.5 | 3.4 | 29.8 |
| Arizona | 2002 | 9.3 | 19.0 | 9.4 | 34.1 | 5.5 | 22.7 |
| Arizona | 2003 | 17.0 | 13.3 | 11.9 | 36.6 | 4.5 | 16.6 |
| Arizona | 2004 | 12.8 | 13.7 | 14.2 | 31.6 | 4.9 | 22.7 |
| Arizona | 2005 | 10.9 | 12.4 | 15.0 | 36.9 | 5.1 | 19.6 |
| Arizona | 2006 | 14.9 | 13.5 | 13.7 | 32.7 | 4.3 | 21.0 |
| Arizona | 2007 | 12.7 | 10.3 | 13.8 | 37.1 | 4.1 | 22.0 |
| Arizona | 2008 | 16.6 | 5.6 | 10.2 | 45.7 | 4.3 | 17.5 |
| Arizona | 2009 | 16.8 | 6.5 | 10.0 | 42.9 | 5.3 | 18.5 |
| Arizona | 2010 | 18.9 | 3.5 | 11.3 | 42.8 | 5.5 | 18.0 |
| Arizona | 2011 | 19.5 | 4.1 | 13.1 | 40.8 | 4.0 | 18.5 |
| Arizona | 2012 | 17.5 | 3.2 | 14.0 | 43.6 | 4.0 | 17.7 |
| Arizona | 2013 | 24.3 | 3.4 | 19.0 | 34.6 | 3.3 | 15.2 |
| Arizona | 2014 | 28.6 | 3.4 | 20.3 | 34.0 | 3.6 | 10.2 |
| Arizona | 2015 | 30.6 | 2.8 | 23.7 | 28.6 | 4.3 | 10.1 |
| Arizona | 2016 | 34.6 | 2.5 | 26.0 | 23.7 | 3.6 | 9.6 |
| Arizona | 2017 | 39.7 | 3.1 | 27.1 | 19.1 | 3.0 | 8.0 |
| Arizona | 2018 | 53.1 | 3.1 | 21.1 | 13.1 | 2.9 | 6.8 |
| Arizona | 2019 | 56.6 | 2.1 | 23.0 | 10.2 | 2.2 | 5.8 |
| Arizona | 2020 | 65.8 | 1.7 | 22.0 | 6.4 | 1.0 | 3.0 |
| Arkansas | 2000 | 11.6 | 11.7 | 19.4 | 15.6 | 0.0 | 40.9 |
| Arkansas | 2001 | 5.7 | 15.2 | 15.1 | 39.3 | 1.8 | 22.1 |
| Arkansas | 2002 | 19.2 | 9.4 | 9.3 | 34.8 | 5.9 | 20.2 |
| Arkansas | 2003 | 18.4 | 8.4 | 13.3 | 37.3 | 2.9 | 19.7 |
| Arkansas | 2004 | 17.3 | 8.1 | 8.5 | 47.6 | 6.3 | 12.3 |
| Arkansas | 2005 | 12.8 | 10.9 | 11.5 | 42.5 | 6.5 | 15.9 |
| Arkansas | 2006 | 15.1 | 7.9 | 11.3 | 41.6 | 6.6 | 17.2 |
| Arkansas | 2007 | 13.2 | 9.2 | 10.1 | 46.6 | 3.5 | 16.8 |
| Arkansas | 2008 | 17.2 | 5.2 | 7.9 | 52.9 | 5.3 | 11.3 |
| Arkansas | 2009 | 22.2 | 3.2 | 10.7 | 44.1 | 4.1 | 15.1 |
| Arkansas | 2010 | 22.3 | 2.5 | 10.0 | 41.7 | 5.9 | 17.7 |
| Arkansas | 2011 | 14.3 | 1.2 | 13.5 | 46.1 | 6.7 | 18.3 |
| Arkansas | 2012 | 10.1 | 2.7 | 16.6 | 50.7 | 4.9 | 15.0 |
| Arkansas | 2013 | 13.5 | 2.9 | 16.4 | 45.1 | 9.2 | 12.6 |
| Arkansas | 2014 | 15.0 | 1.7 | 21.1 | 42.6 | 8.3 | 11.0 |
| Arkansas | 2015 | 17.3 | 2.3 | 16.6 | 44.4 | 7.2 | 11.9 |
| Arkansas | 2016 | 17.4 | 1.4 | 20.4 | 37.2 | 8.2 | 14.9 |
| Arkansas | 2017 | 24.1 | 3.4 | 23.9 | 26.5 | 6.3 | 15.8 |
| Arkansas | 2018 | 29.8 | 2.0 | 26.0 | 21.9 | 4.9 | 15.4 |
| Arkansas | 2019 | 38.6 | 1.5 | 26.5 | 15.2 | 5.4 | 12.8 |
| Arkansas | 2020 | 45.1 | 1.5 | 29.9 | 11.8 | 3.1 | 8.5 |
| California | 2000 | 26.0 | 9.3 | 11.7 | 27.2 | 5.3 | 20.5 |
| California | 2001 | 28.0 | 10.3 | 11.0 | 23.3 | 5.3 | 22.1 |
| California | 2002 | 20.9 | 9.4 | 14.6 | 31.7 | 4.0 | 19.4 |
| California | 2003 | 20.3 | 9.5 | 18.2 | 30.8 | 4.4 | 16.8 |
| California | 2004 | 18.5 | 9.7 | 17.8 | 32.2 | 4.6 | 17.3 |
| California | 2005 | 15.7 | 10.2 | 20.8 | 31.7 | 4.0 | 17.6 |
| California | 2006 | 16.7 | 11.8 | 18.4 | 32.0 | 4.8 | 16.3 |
| California | 2007 | 15.7 | 10.0 | 16.4 | 36.1 | 4.5 | 17.3 |
| California | 2008 | 17.7 | 6.5 | 15.7 | 40.2 | 4.2 | 15.8 |
| California | 2009 | 18.6 | 5.3 | 16.3 | 40.3 | 3.8 | 15.7 |
| California | 2010 | 17.7 | 4.6 | 17.3 | 39.8 | 4.0 | 16.7 |
| California | 2011 | 17.2 | 4.2 | 19.9 | 40.0 | 4.0 | 14.8 |
| California | 2012 | 17.5 | 4.2 | 23.6 | 34.7 | 3.9 | 16.0 |
| California | 2013 | 19.5 | 3.6 | 27.2 | 31.2 | 4.2 | 14.3 |
| California | 2014 | 21.3 | 3.8 | 27.7 | 29.8 | 4.1 | 13.2 |
| California | 2015 | 22.0 | 4.3 | 32.8 | 26.4 | 3.8 | 10.8 |
| California | 2016 | 24.5 | 4.3 | 34.9 | 22.2 | 4.1 | 10.0 |
| California | 2017 | 28.3 | 4.5 | 37.0 | 18.1 | 2.9 | 9.3 |
| California | 2018 | 32.5 | 4.4 | 38.7 | 13.6 | 3.3 | 7.5 |
| California | 2019 | 42.8 | 4.9 | 34.0 | 10.0 | 2.5 | 5.9 |
| California | 2020 | 55.7 | 3.0 | 29.5 | 6.0 | 1.6 | 4.1 |
| Colorado | 2000 | 22.2 | 23.9 | 5.7 | 16.4 | 3.6 | 27.9 |
| Colorado | 2001 | 12.3 | 20.3 | 6.5 | 28.1 | 8.4 | 24.4 |
| Colorado | 2002 | 17.1 | 23.3 | 3.7 | 24.7 | 4.4 | 26.6 |
| Colorado | 2003 | 12.2 | 26.1 | 9.0 | 25.2 | 3.0 | 24.0 |
| Colorado | 2004 | 11.3 | 24.4 | 7.5 | 27.0 | 3.4 | 26.0 |
| Colorado | 2005 | 17.0 | 19.0 | 10.1 | 30.5 | 3.6 | 19.8 |
| Colorado | 2006 | 13.5 | 24.3 | 6.7 | 34.5 | 3.5 | 17.6 |
| Colorado | 2007 | 16.0 | 16.1 | 7.1 | 41.6 | 2.5 | 16.6 |
| Colorado | 2008 | 16.4 | 12.4 | 6.8 | 38.7 | 4.2 | 21.5 |
| Colorado | 2009 | 26.6 | 8.3 | 6.7 | 37.1 | 5.9 | 15.3 |
| Colorado | 2010 | 24.4 | 9.1 | 7.4 | 35.7 | 5.1 | 18.3 |
| Colorado | 2011 | 27.2 | 7.6 | 9.1 | 38.7 | 3.5 | 13.8 |
| Colorado | 2012 | 24.9 | 4.9 | 8.3 | 41.5 | 2.3 | 17.9 |
| Colorado | 2013 | 29.8 | 3.3 | 11.5 | 33.8 | 4.2 | 17.5 |
| Colorado | 2014 | 32.4 | 4.4 | 11.2 | 33.8 | 3.7 | 14.5 |
| Colorado | 2015 | 31.8 | 3.4 | 15.3 | 34.2 | 4.9 | 10.4 |
| Colorado | 2016 | 38.8 | 5.5 | 16.1 | 26.8 | 4.3 | 8.6 |
| Colorado | 2017 | 37.5 | 4.3 | 22.5 | 24.9 | 2.1 | 8.7 |
| Colorado | 2018 | 39.1 | 5.8 | 23.8 | 19.5 | 2.7 | 9.1 |
| Colorado | 2019 | 46.4 | 5.1 | 21.8 | 16.3 | 2.2 | 8.2 |
| Colorado | 2020 | 56.2 | 4.4 | 20.0 | 11.2 | 1.7 | 6.5 |
| Connecticut | 2000 | 49.1 | 12.8 | 0.0 | 10.5 | 3.6 | 23.6 |
| Connecticut | 2001 | 50.6 | 13.5 | 0.0 | 13.5 | 3.3 | 18.5 |
| Connecticut | 2002 | 39.9 | 14.6 | 0.9 | 19.3 | 2.3 | 23.1 |
| Connecticut | 2003 | 51.0 | 13.2 | 0.0 | 17.0 | 1.3 | 17.5 |
| Connecticut | 2004 | 39.6 | 13.7 | 0.0 | 27.0 | 0.4 | 19.2 |
| Connecticut | 2005 | 37.5 | 20.7 | 0.5 | 24.5 | 1.9 | 14.2 |
| Connecticut | 2006 | 31.4 | 19.0 | 1.0 | 28.3 | 2.3 | 18.0 |
| Connecticut | 2007 | 41.9 | 13.6 | 0.5 | 22.0 | 1.6 | 20.2 |
| Connecticut | 2008 | 42.2 | 11.6 | 0.6 | 28.5 | 1.5 | 15.1 |
| Connecticut | 2009 | 41.0 | 14.2 | 0.3 | 23.8 | 1.9 | 17.3 |
| Connecticut | 2010 | 34.4 | 9.8 | 0.3 | 25.7 | 7.7 | 22.2 |
| Connecticut | 2011 | 34.1 | 16.0 | 0.4 | 25.7 | 1.5 | 22.4 |
| Connecticut | 2012 | 40.2 | 13.3 | 1.3 | 20.1 | 0.9 | 24.3 |
| Connecticut | 2013 | 49.0 | 9.2 | 1.1 | 27.6 | 4.2 | 8.8 |
| Connecticut | 2014 | 58.4 | 4.9 | 1.8 | 22.3 | 4.9 | 7.7 |
| Connecticut | 2015 | 63.7 | 5.1 | 1.2 | 21.0 | 3.7 | 5.4 |
| Connecticut | 2016 | 73.2 | 4.0 | 0.6 | 14.9 | 2.5 | 4.8 |
| Connecticut | 2017 | 76.9 | 3.3 | 1.0 | 12.6 | 3.1 | 3.0 |
| Connecticut | 2018 | 78.7 | 3.4 | 2.2 | 9.3 | 2.6 | 3.8 |
| Connecticut | 2019 | 82.8 | 3.0 | 1.2 | 8.0 | 2.4 | 2.6 |
| Connecticut | 2020 | 85.6 | 2.8 | 0.9 | 6.2 | 2.1 | 2.4 |
| Delaware | 2000 | 24.9 | 19.1 | 2.3 | 16.8 | 11.4 | 25.5 |
| Delaware | 2001 | 22.4 | 18.1 | 0.0 | 22.5 | 9.3 | 26.1 |
| Delaware | 2002 | 24.5 | 18.9 | 2.6 | 33.0 | 5.8 | 12.8 |
| Delaware | 2003 | 32.7 | 3.3 | 0.0 | 33.3 | 9.1 | 17.6 |
| Delaware | 2004 | 21.2 | 17.4 | 0.0 | 35.3 | 7.1 | 19.0 |
| Delaware | 2005 | 20.3 | 13.0 | 0.0 | 42.6 | 8.6 | 13.9 |
| Delaware | 2006 | 35.0 | 14.4 | 1.5 | 28.5 | 1.5 | 16.5 |
| Delaware | 2007 | 23.7 | 8.5 | 1.5 | 40.0 | 2.8 | 19.3 |
| Delaware | 2008 | 19.9 | 2.8 | 3.7 | 55.0 | 5.8 | 11.1 |
| Delaware | 2009 | 24.2 | 5.0 | 1.9 | 42.1 | 6.6 | 19.4 |
| Delaware | 2010 | 25.3 | 8.4 | 1.6 | 46.7 | 5.5 | 12.5 |
| Delaware | 2011 | 22.3 | 2.1 | 0.0 | 51.3 | 1.6 | 22.0 |
| Delaware | 2012 | 37.5 | 10.7 | 0.0 | 36.6 | 2.1 | 13.1 |
| Delaware | 2013 | 42.1 | 4.2 | 3.7 | 25.6 | 7.3 | 17.1 |
| Delaware | 2014 | 50.0 | 5.2 | 1.4 | 29.3 | 0.6 | 13.4 |
| Delaware | 2015 | 58.0 | 3.9 | 0.7 | 24.0 | 0.0 | 12.0 |
| Delaware | 2016 | 63.9 | 6.7 | 1.5 | 15.6 | 1.0 | 10.9 |
| Delaware | 2017 | 73.3 | 10.4 | 0.0 | 9.9 | 0.4 | 5.7 |
| Delaware | 2018 | 81.3 | 5.6 | 1.8 | 5.4 | 1.3 | 4.6 |
| Delaware | 2019 | 83.3 | 4.4 | 1.9 | 6.4 | 0.2 | 3.8 |
| Delaware | 2020 | 82.3 | 4.5 | 2.3 | 7.5 | 0.0 | 3.4 |
| District of Columbia | 2000 | 9.7 | 51.0 | 0.0 | 11.3 | 1.3 | 26.6 |
| District of Columbia | 2001 | 0.0 | 55.0 | 0.0 | 6.6 | 1.1 | 36.3 |
| District of Columbia | 2002 | 6.7 | 55.0 | 3.3 | 10.8 | 4.1 | 20.2 |
| District of Columbia | 2003 | 3.1 | 54.5 | 1.0 | 13.0 | 1.0 | 27.4 |
| District of Columbia | 2004 | 12.1 | 32.9 | 1.5 | 26.3 | 7.3 | 18.9 |
| District of Columbia | 2005 | 5.5 | 43.2 | 1.3 | 21.8 | 2.6 | 25.6 |
| District of Columbia | 2006 | 7.2 | 48.1 | 0.0 | 19.8 | 2.0 | 22.8 |
| District of Columbia | 2007 | 6.8 | 42.0 | 0.0 | 17.2 | 6.6 | 27.5 |
| District of Columbia | 2008 | 10.7 | 32.1 | 1.8 | 26.8 | 1.8 | 26.8 |
| District of Columbia | 2009 | 11.5 | 38.5 | 0.0 | 19.2 | 0.0 | 30.8 |
| District of Columbia | 2010 | 25.4 | 38.3 | 1.3 | 10.7 | 0.0 | 24.3 |
| District of Columbia | 2011 | 31.2 | 25.6 | 0.0 | 22.2 | 0.0 | 19.9 |
| District of Columbia | 2012 | 40.0 | 24.6 | 0.0 | 24.1 | 1.3 | 10.0 |
| District of Columbia | 2013 | 43.4 | 19.8 | 1.0 | 17.3 | 1.1 | 16.3 |
| District of Columbia | 2014 | 48.2 | 17.0 | 1.3 | 15.0 | 1.0 | 16.4 |
| District of Columbia | 2015 | 63.7 | 12.9 | 1.7 | 15.3 | 0.8 | 5.6 |
| District of Columbia | 2016 | 66.5 | 11.6 | 1.1 | 12.0 | 1.5 | 7.2 |
| District of Columbia | 2017 | 69.8 | 12.9 | 1.6 | 8.2 | 1.0 | 6.6 |
| District of Columbia | 2018 | 68.8 | 13.2 | 3.9 | 5.2 | 0.0 | 8.8 |
| District of Columbia | 2019 | 73.8 | 11.0 | 1.6 | 3.9 | 0.3 | 9.4 |
| District of Columbia | 2020 | 75.2 | 7.6 | 1.0 | 3.3 | 0.2 | 12.7 |
| Florida | 2000 | 29.3 | 12.2 | 1.8 | 29.9 | 7.8 | 18.9 |
| Florida | 2001 | 25.2 | 13.1 | 2.9 | 37.7 | 6.2 | 14.9 |
| Florida | 2002 | 24.9 | 13.3 | 1.4 | 41.1 | 4.0 | 15.3 |
| Florida | 2003 | 25.2 | 15.8 | 1.1 | 42.6 | 3.9 | 11.4 |
| Florida | 2004 | 17.8 | 14.2 | 1.8 | 50.7 | 4.2 | 11.2 |
| Florida | 2005 | 15.4 | 18.3 | 2.0 | 49.1 | 4.8 | 10.3 |
| Florida | 2006 | 12.1 | 17.7 | 1.6 | 53.0 | 5.0 | 10.6 |
| Florida | 2007 | 10.7 | 15.2 | 1.7 | 59.0 | 4.4 | 9.1 |
| Florida | 2008 | 12.0 | 9.6 | 1.8 | 59.1 | 8.4 | 9.1 |
| Florida | 2009 | 12.9 | 7.6 | 1.9 | 61.9 | 6.0 | 9.6 |
| Florida | 2010 | 9.4 | 6.7 | 2.6 | 68.0 | 5.0 | 8.2 |
| Florida | 2011 | 9.8 | 7.8 | 2.8 | 65.4 | 4.6 | 9.5 |
| Florida | 2012 | 14.3 | 7.8 | 3.4 | 56.2 | 5.7 | 12.5 |
| Florida | 2013 | 20.9 | 10.2 | 4.2 | 48.8 | 4.2 | 11.9 |
| Florida | 2014 | 32.2 | 9.7 | 3.4 | 39.8 | 4.2 | 10.6 |
| Florida | 2015 | 42.8 | 8.7 | 4.4 | 30.6 | 5.2 | 8.3 |
| Florida | 2016 | 54.0 | 9.6 | 4.9 | 22.2 | 2.5 | 6.8 |
| Florida | 2017 | 58.6 | 10.4 | 5.2 | 17.3 | 2.7 | 5.7 |
| Florida | 2018 | 56.8 | 8.4 | 6.6 | 17.8 | 2.6 | 7.6 |
| Florida | 2019 | 64.5 | 7.3 | 7.5 | 13.2 | 2.0 | 5.5 |
| Florida | 2020 | 74.7 | 5.3 | 6.7 | 8.0 | 1.6 | 3.7 |
| Georgia | 2000 | 5.6 | 26.4 | 3.8 | 29.2 | 7.8 | 26.9 |
| Georgia | 2001 | 9.3 | 26.0 | 3.3 | 36.1 | 5.1 | 20.1 |
| Georgia | 2002 | 10.8 | 22.5 | 5.0 | 36.3 | 5.8 | 19.4 |
| Georgia | 2003 | 11.1 | 24.9 | 4.1 | 37.5 | 5.6 | 16.6 |
| Georgia | 2004 | 8.9 | 21.2 | 8.7 | 40.1 | 4.3 | 16.8 |
| Georgia | 2005 | 10.7 | 20.5 | 8.3 | 42.3 | 4.8 | 13.1 |
| Georgia | 2006 | 11.6 | 20.2 | 7.4 | 45.8 | 2.8 | 12.3 |
| Georgia | 2007 | 12.9 | 18.8 | 4.3 | 45.9 | 3.2 | 14.9 |
| Georgia | 2008 | 13.8 | 13.8 | 4.4 | 49.5 | 4.5 | 14.0 |
| Georgia | 2009 | 14.5 | 12.4 | 5.8 | 50.0 | 3.2 | 14.0 |
| Georgia | 2010 | 14.8 | 9.2 | 8.2 | 48.4 | 4.0 | 15.2 |
| Georgia | 2011 | 15.3 | 8.7 | 6.9 | 51.7 | 4.7 | 12.5 |
| Georgia | 2012 | 13.0 | 8.2 | 9.5 | 50.7 | 5.1 | 13.5 |
| Georgia | 2013 | 18.6 | 7.9 | 13.0 | 42.9 | 4.1 | 13.6 |
| Georgia | 2014 | 28.3 | 6.8 | 10.6 | 39.0 | 3.0 | 12.2 |
| Georgia | 2015 | 37.4 | 6.1 | 13.2 | 30.9 | 3.0 | 9.5 |
| Georgia | 2016 | 37.0 | 8.6 | 13.4 | 28.9 | 2.2 | 9.9 |
| Georgia | 2017 | 41.3 | 6.6 | 16.6 | 25.5 | 1.9 | 8.2 |
| Georgia | 2018 | 41.5 | 8.3 | 18.3 | 20.6 | 2.8 | 8.5 |
| Georgia | 2019 | 44.1 | 8.8 | 20.1 | 17.8 | 2.2 | 6.9 |
| Georgia | 2020 | 58.1 | 7.2 | 15.1 | 12.9 | 1.8 | 5.0 |
| Hawaii | 2000 | 9.9 | 5.2 | 16.5 | 43.9 | 0.0 | 24.5 |
| Hawaii | 2001 | 12.2 | 6.5 | 22.7 | 38.1 | 5.3 | 15.2 |
| Hawaii | 2002 | 12.6 | 5.2 | 18.3 | 45.0 | 1.5 | 17.4 |
| Hawaii | 2003 | 9.6 | 6.7 | 24.0 | 31.9 | 2.2 | 23.5 |
| Hawaii | 2004 | 12.5 | 3.1 | 28.6 | 39.2 | 0.0 | 16.6 |
| Hawaii | 2005 | 7.5 | 1.7 | 30.5 | 46.2 | 0.0 | 13.3 |
| Hawaii | 2006 | 5.2 | 4.0 | 24.9 | 49.5 | 3.9 | 12.6 |
| Hawaii | 2007 | 7.4 | 7.5 | 22.8 | 52.2 | 2.5 | 7.7 |
| Hawaii | 2008 | 5.0 | 5.3 | 29.9 | 40.7 | 5.2 | 13.9 |
| Hawaii | 2009 | 4.3 | 7.8 | 32.1 | 41.9 | 4.5 | 9.4 |
| Hawaii | 2010 | 8.1 | 4.4 | 34.3 | 34.9 | 4.5 | 13.8 |
| Hawaii | 2011 | 8.6 | 3.0 | 30.9 | 38.0 | 7.9 | 11.6 |
| Hawaii | 2012 | 7.9 | 2.3 | 30.9 | 30.3 | 9.8 | 18.1 |
| Hawaii | 2013 | 13.1 | 0.7 | 36.9 | 31.6 | 5.5 | 12.2 |
| Hawaii | 2014 | 16.2 | 0.7 | 37.8 | 27.7 | 6.4 | 10.0 |
| Hawaii | 2015 | 19.8 | 3.3 | 49.9 | 17.2 | 2.0 | 7.9 |
| Hawaii | 2016 | 16.2 | 2.7 | 50.2 | 23.5 | 2.2 | 5.2 |
| Hawaii | 2017 | 9.3 | 2.7 | 56.1 | 17.5 | 6.8 | 7.6 |
| Hawaii | 2018 | 14.5 | 3.9 | 66.8 | 9.0 | 1.2 | 4.6 |
| Hawaii | 2019 | 15.5 | 3.6 | 65.6 | 7.1 | 2.2 | 6.0 |
| Hawaii | 2020 | 20.4 | 2.3 | 62.3 | 6.0 | 2.2 | 6.8 |
| Idaho | 2000 | 5.6 | 10.9 | 5.6 | 39.8 | 8.3 | 29.8 |
| Idaho | 2001 | 18.0 | 3.8 | 7.6 | 41.9 | 2.6 | 25.2 |
| Idaho | 2002 | 13.6 | 1.3 | 6.8 | 35.6 | 8.6 | 34.0 |
| Idaho | 2003 | 9.7 | 3.4 | 10.4 | 53.0 | 5.5 | 17.0 |
| Idaho | 2004 | 15.9 | 0.0 | 10.4 | 48.4 | 1.4 | 21.7 |
| Idaho | 2005 | 9.8 | 2.6 | 21.3 | 40.4 | 7.3 | 16.8 |
| Idaho | 2006 | 15.1 | 1.0 | 8.5 | 55.5 | 4.6 | 13.8 |
| Idaho | 2007 | 16.5 | 1.3 | 9.9 | 52.4 | 1.3 | 17.8 |
| Idaho | 2008 | 11.5 | 2.2 | 0.0 | 59.9 | 4.9 | 19.5 |
| Idaho | 2009 | 12.1 | 0.0 | 14.0 | 48.5 | 4.8 | 19.5 |
| Idaho | 2010 | 14.8 | 0.0 | 11.1 | 49.7 | 5.2 | 16.4 |
| Idaho | 2011 | 8.9 | 0.9 | 13.0 | 51.9 | 6.5 | 15.8 |
| Idaho | 2012 | 13.7 | 2.0 | 9.9 | 47.6 | 5.7 | 17.2 |
| Idaho | 2013 | 18.3 | 0.0 | 18.8 | 39.2 | 6.4 | 15.9 |
| Idaho | 2014 | 18.0 | 0.7 | 18.0 | 38.6 | 4.0 | 20.7 |
| Idaho | 2015 | 18.9 | 0.0 | 20.4 | 34.7 | 6.0 | 20.0 |
| Idaho | 2016 | 23.5 | 1.0 | 21.7 | 35.2 | 3.5 | 13.8 |
| Idaho | 2017 | 25.9 | 1.1 | 22.5 | 25.8 | 4.5 | 20.2 |
| Idaho | 2018 | 26.1 | 1.1 | 26.1 | 26.9 | 5.1 | 14.2 |
| Idaho | 2019 | 27.9 | 0.4 | 27.4 | 24.1 | 6.9 | 13.3 |
| Idaho | 2020 | 35.1 | 0.8 | 26.3 | 21.3 | 4.0 | 12.2 |
| Illinois | 2000 | 7.6 | 38.2 | 1.1 | 11.8 | 1.9 | 39.2 |
| Illinois | 2001 | 9.5 | 36.7 | 0.7 | 16.1 | 2.4 | 34.7 |
| Illinois | 2002 | 10.7 | 36.5 | 1.0 | 16.1 | 1.7 | 33.9 |
| Illinois | 2003 | 7.5 | 40.2 | 0.8 | 17.4 | 3.4 | 30.7 |
| Illinois | 2004 | 8.6 | 42.8 | 1.7 | 15.2 | 1.7 | 30.0 |
| Illinois | 2005 | 11.7 | 39.1 | 1.1 | 17.7 | 2.9 | 27.5 |
| Illinois | 2006 | 34.3 | 29.2 | 0.5 | 15.4 | 1.8 | 18.7 |
| Illinois | 2007 | 16.4 | 32.6 | 1.0 | 20.9 | 2.7 | 26.4 |
| Illinois | 2008 | 16.7 | 27.6 | 1.2 | 20.8 | 2.5 | 31.2 |
| Illinois | 2009 | 19.9 | 22.0 | 1.1 | 21.6 | 2.7 | 32.6 |
| Illinois | 2010 | 24.5 | 20.2 | 1.0 | 20.8 | 2.1 | 31.4 |
| Illinois | 2011 | 22.0 | 20.5 | 0.9 | 19.7 | 2.5 | 34.3 |
| Illinois | 2012 | 25.0 | 18.3 | 1.1 | 17.8 | 3.2 | 34.6 |
| Illinois | 2013 | 48.4 | 10.1 | 1.3 | 18.4 | 4.3 | 17.5 |
| Illinois | 2014 | 53.7 | 8.4 | 1.9 | 19.5 | 3.6 | 12.8 |
| Illinois | 2015 | 61.2 | 8.0 | 2.0 | 16.1 | 3.6 | 9.1 |
| Illinois | 2016 | 68.0 | 6.4 | 3.0 | 13.2 | 2.5 | 6.9 |
| Illinois | 2017 | 69.4 | 7.7 | 2.9 | 12.6 | 2.4 | 5.0 |
| Illinois | 2018 | 71.2 | 7.3 | 4.5 | 9.8 | 2.4 | 4.8 |
| Illinois | 2019 | 71.6 | 7.9 | 4.4 | 8.9 | 2.4 | 4.7 |
| Illinois | 2020 | 75.6 | 6.7 | 4.3 | 8.4 | 1.3 | 3.8 |
| Indiana | 2000 | 17.1 | 20.2 | 2.9 | 17.5 | 9.3 | 32.6 |
| Indiana | 2001 | 16.6 | 17.3 | 0.7 | 29.3 | 4.3 | 31.7 |
| Indiana | 2002 | 9.1 | 19.2 | 3.5 | 33.2 | 5.6 | 28.7 |
| Indiana | 2003 | 18.3 | 11.1 | 2.2 | 38.5 | 3.8 | 25.7 |
| Indiana | 2004 | 15.6 | 15.7 | 2.4 | 36.0 | 3.3 | 26.9 |
| Indiana | 2005 | 18.6 | 16.3 | 2.6 | 39.2 | 4.8 | 18.4 |
| Indiana | 2006 | 22.0 | 15.7 | 4.3 | 33.4 | 2.9 | 21.2 |
| Indiana | 2007 | 20.4 | 12.0 | 1.8 | 43.0 | 5.3 | 17.2 |
| Indiana | 2008 | 29.0 | 7.4 | 1.5 | 41.3 | 4.9 | 15.6 |
| Indiana | 2009 | 31.4 | 4.6 | 2.7 | 45.8 | 3.5 | 11.7 |
| Indiana | 2010 | 24.8 | 6.3 | 4.3 | 39.2 | 4.8 | 20.5 |
| Indiana | 2011 | 23.8 | 3.0 | 2.7 | 45.2 | 4.9 | 20.3 |
| Indiana | 2012 | 32.4 | 4.3 | 3.0 | 36.1 | 5.1 | 18.9 |
| Indiana | 2013 | 38.4 | 4.3 | 3.4 | 25.2 | 4.1 | 24.3 |
| Indiana | 2014 | 40.3 | 3.1 | 5.7 | 29.1 | 3.5 | 18.1 |
| Indiana | 2015 | 49.3 | 3.6 | 5.5 | 20.0 | 4.9 | 16.6 |
| Indiana | 2016 | 55.8 | 3.9 | 7.2 | 16.9 | 4.2 | 12.0 |
| Indiana | 2017 | 57.5 | 5.6 | 8.5 | 15.5 | 3.3 | 9.6 |
| Indiana | 2018 | 60.1 | 4.1 | 11.9 | 12.5 | 2.9 | 8.4 |
| Indiana | 2019 | 60.7 | 3.8 | 13.9 | 11.4 | 2.3 | 8.0 |
| Indiana | 2020 | 75.5 | 2.9 | 9.2 | 6.6 | 1.5 | 4.3 |
| Iowa | 2000 | 17.2 | 19.8 | 5.3 | 12.6 | 12.6 | 32.6 |
| Iowa | 2001 | 10.5 | 4.7 | 12.0 | 21.6 | 8.7 | 42.4 |
| Iowa | 2002 | 17.6 | 11.2 | 9.6 | 23.4 | 4.0 | 34.3 |
| Iowa | 2003 | 15.7 | 10.3 | 14.2 | 20.6 | 10.7 | 28.5 |
| Iowa | 2004 | 16.0 | 9.5 | 5.5 | 37.3 | 2.8 | 28.9 |
| Iowa | 2005 | 9.1 | 4.9 | 11.8 | 41.2 | 10.6 | 21.7 |
| Iowa | 2006 | 11.8 | 8.4 | 11.2 | 39.0 | 3.6 | 26.1 |
| Iowa | 2007 | 11.6 | 6.5 | 6.5 | 42.2 | 9.3 | 23.9 |
| Iowa | 2008 | 18.5 | 4.2 | 4.7 | 41.5 | 9.7 | 21.0 |
| Iowa | 2009 | 23.4 | 6.1 | 5.2 | 44.3 | 4.3 | 16.7 |
| Iowa | 2010 | 22.2 | 4.6 | 9.8 | 35.0 | 8.0 | 20.4 |
| Iowa | 2011 | 18.4 | 0.4 | 4.8 | 43.7 | 5.1 | 27.6 |
| Iowa | 2012 | 21.0 | 4.1 | 10.5 | 42.9 | 3.0 | 18.6 |
| Iowa | 2013 | 25.2 | 1.6 | 15.5 | 37.4 | 6.4 | 13.9 |
| Iowa | 2014 | 28.1 | 2.5 | 13.4 | 29.9 | 9.0 | 17.1 |
| Iowa | 2015 | 31.5 | 2.6 | 19.6 | 24.3 | 6.3 | 15.7 |
| Iowa | 2016 | 34.3 | 1.0 | 22.0 | 21.2 | 7.1 | 14.0 |
| Iowa | 2017 | 38.9 | 1.5 | 21.2 | 21.9 | 4.7 | 11.6 |
| Iowa | 2018 | 36.1 | 1.5 | 28.0 | 15.1 | 4.2 | 15.1 |
| Iowa | 2019 | 35.2 | 1.8 | 37.6 | 11.1 | 2.5 | 11.8 |
| Iowa | 2020 | 45.1 | 1.7 | 29.2 | 8.7 | 3.3 | 11.7 |
| Kansas | 2000 | 11.4 | 23.9 | 9.5 | 14.5 | 6.1 | 34.6 |
| Kansas | 2001 | 15.1 | 14.7 | 5.0 | 34.2 | 2.4 | 27.1 |
| Kansas | 2002 | 14.9 | 8.6 | 8.9 | 45.3 | 2.6 | 19.2 |
| Kansas | 2003 | 7.7 | 17.2 | 4.6 | 41.3 | 5.3 | 21.8 |
| Kansas | 2004 | 13.9 | 12.2 | 7.1 | 49.0 | 3.0 | 14.7 |
| Kansas | 2005 | 11.3 | 13.1 | 9.3 | 43.6 | 2.2 | 20.1 |
| Kansas | 2006 | 10.9 | 13.9 | 7.9 | 48.6 | 1.6 | 16.7 |
| Kansas | 2007 | 12.8 | 13.0 | 6.3 | 50.4 | 3.5 | 13.8 |
| Kansas | 2008 | 20.3 | 3.8 | 2.3 | 50.2 | 1.5 | 20.5 |
| Kansas | 2009 | 24.9 | 1.7 | 8.4 | 47.1 | 0.5 | 15.7 |
| Kansas | 2010 | 17.1 | 5.7 | 11.0 | 47.6 | 2.1 | 15.7 |
| Kansas | 2011 | 16.8 | 5.4 | 11.9 | 43.4 | 4.1 | 17.0 |
| Kansas | 2012 | 26.0 | 2.2 | 11.0 | 42.5 | 2.2 | 15.2 |
| Kansas | 2013 | 22.6 | 2.9 | 12.9 | 44.7 | 4.3 | 12.4 |
| Kansas | 2014 | 24.1 | 3.4 | 11.7 | 46.0 | 2.9 | 11.9 |
| Kansas | 2015 | 22.2 | 3.5 | 24.2 | 32.2 | 3.3 | 14.0 |
| Kansas | 2016 | 23.0 | 2.2 | 28.1 | 29.8 | 3.0 | 13.6 |
| Kansas | 2017 | 21.0 | 5.4 | 26.8 | 28.1 | 3.6 | 15.2 |
| Kansas | 2018 | 29.5 | 5.1 | 23.4 | 22.5 | 5.0 | 14.5 |
| Kansas | 2019 | 30.5 | 4.2 | 34.4 | 16.3 | 1.6 | 12.7 |
| Kansas | 2020 | 41.5 | 3.5 | 31.9 | 10.5 | 2.9 | 9.7 |
| Kentucky | 2000 | 4.2 | 7.0 | 2.2 | 50.8 | 8.5 | 26.9 |
| Kentucky | 2001 | 10.2 | 7.6 | 1.0 | 52.9 | 6.5 | 21.1 |
| Kentucky | 2002 | 13.8 | 7.1 | 4.6 | 51.3 | 5.7 | 17.5 |
| Kentucky | 2003 | 9.4 | 7.6 | 3.5 | 58.0 | 3.3 | 18.1 |
| Kentucky | 2004 | 11.0 | 6.7 | 4.7 | 57.5 | 3.2 | 16.8 |
| Kentucky | 2005 | 8.2 | 8.8 | 3.3 | 61.7 | 1.5 | 16.5 |
| Kentucky | 2006 | 12.1 | 8.9 | 2.1 | 58.8 | 3.3 | 14.8 |
| Kentucky | 2007 | 10.0 | 7.4 | 1.7 | 68.6 | 3.4 | 8.5 |
| Kentucky | 2008 | 12.5 | 5.3 | 1.1 | 65.4 | 4.9 | 10.6 |
| Kentucky | 2009 | 17.6 | 3.1 | 2.3 | 62.8 | 6.9 | 7.0 |
| Kentucky | 2010 | 12.8 | 2.5 | 2.1 | 68.6 | 7.0 | 7.0 |
| Kentucky | 2011 | 17.7 | 1.5 | 3.7 | 61.8 | 8.2 | 7.2 |
| Kentucky | 2012 | 27.7 | 2.6 | 2.4 | 50.2 | 7.1 | 10.0 |
| Kentucky | 2013 | 36.9 | 4.6 | 4.2 | 40.5 | 5.1 | 8.6 |
| Kentucky | 2014 | 46.2 | 2.7 | 5.1 | 33.8 | 5.6 | 6.5 |
| Kentucky | 2015 | 54.9 | 2.9 | 5.4 | 28.4 | 2.3 | 6.0 |
| Kentucky | 2016 | 57.4 | 3.3 | 8.4 | 22.1 | 3.3 | 5.4 |
| Kentucky | 2017 | 64.3 | 3.1 | 10.9 | 14.3 | 2.0 | 5.2 |
| Kentucky | 2018 | 65.6 | 2.5 | 13.0 | 13.0 | 2.1 | 3.6 |
| Kentucky | 2019 | 66.4 | 2.1 | 14.8 | 11.1 | 1.6 | 3.9 |
| Kentucky | 2020 | 77.0 | 1.4 | 11.6 | 6.7 | 0.9 | 2.3 |
| Louisiana | 2000 | 8.5 | 18.3 | 1.1 | 28.2 | 11.4 | 30.6 |
| Louisiana | 2001 | 8.2 | 22.1 | 2.6 | 40.4 | 4.0 | 21.8 |
| Louisiana | 2002 | 5.8 | 21.4 | 1.2 | 46.4 | 6.7 | 18.4 |
| Louisiana | 2003 | 6.5 | 22.7 | 2.5 | 43.8 | 6.3 | 17.6 |
| Louisiana | 2004 | 7.2 | 15.2 | 1.3 | 54.2 | 8.3 | 12.7 |
| Louisiana | 2005 | 5.9 | 23.2 | 3.2 | 50.3 | 1.9 | 13.8 |
| Louisiana | 2006 | 8.4 | 23.9 | 1.5 | 51.4 | 4.8 | 10.1 |
| Louisiana | 2007 | 7.5 | 25.5 | 3.6 | 51.1 | 2.8 | 9.6 |
| Louisiana | 2008 | 11.9 | 23.3 | 2.5 | 42.5 | 4.7 | 14.4 |
| Louisiana | 2009 | 14.1 | 20.2 | 5.6 | 35.2 | 3.3 | 20.9 |
| Louisiana | 2010 | 17.2 | 16.0 | 3.7 | 37.9 | 5.1 | 19.2 |
| Louisiana | 2011 | 19.4 | 21.3 | 2.4 | 31.9 | 6.0 | 18.4 |
| Louisiana | 2012 | 29.6 | 10.4 | 2.6 | 36.5 | 4.5 | 15.5 |
| Louisiana | 2013 | 37.4 | 12.1 | 7.7 | 22.5 | 2.7 | 17.0 |
| Louisiana | 2014 | 35.1 | 13.2 | 7.3 | 22.7 | 2.8 | 18.9 |
| Louisiana | 2015 | 39.9 | 10.9 | 9.3 | 24.2 | 2.0 | 13.4 |
| Louisiana | 2016 | 42.1 | 12.9 | 13.0 | 18.1 | 1.7 | 11.9 |
| Louisiana | 2017 | 47.4 | 12.3 | 11.3 | 17.2 | 2.6 | 9.1 |
| Louisiana | 2018 | 54.5 | 7.6 | 12.6 | 14.0 | 1.9 | 9.4 |
| Louisiana | 2019 | 55.4 | 7.2 | 14.5 | 12.4 | 1.2 | 9.2 |
| Louisiana | 2020 | 65.8 | 7.7 | 12.7 | 8.6 | 0.6 | 4.5 |
| Maine | 2000 | 13.1 | 0.0 | 0.0 | 49.9 | 8.6 | 28.3 |
| Maine | 2001 | 12.5 | 2.8 | 1.4 | 40.1 | 8.4 | 34.8 |
| Maine | 2002 | 13.3 | 2.0 | 0.8 | 54.3 | 9.9 | 19.6 |
| Maine | 2003 | 11.9 | 3.7 | 0.0 | 59.6 | 3.4 | 21.3 |
| Maine | 2004 | 7.9 | 11.1 | 0.9 | 65.4 | 5.3 | 9.4 |
| Maine | 2005 | 6.8 | 5.5 | 1.4 | 62.7 | 4.1 | 19.6 |
| Maine | 2006 | 3.9 | 7.4 | 0.7 | 68.2 | 3.8 | 16.0 |
| Maine | 2007 | 9.1 | 7.4 | 1.5 | 62.1 | 2.6 | 16.7 |
| Maine | 2008 | 15.2 | 1.6 | 3.6 | 56.6 | 1.5 | 21.5 |
| Maine | 2009 | 12.2 | 1.3 | 3.1 | 55.4 | 5.7 | 22.2 |
| Maine | 2010 | 17.6 | 0.8 | 1.6 | 53.2 | 7.8 | 19.0 |
| Maine | 2011 | 17.4 | 4.6 | 3.7 | 47.8 | 5.6 | 20.9 |
| Maine | 2012 | 20.8 | 2.3 | 5.4 | 49.7 | 5.3 | 16.4 |
| Maine | 2013 | 23.7 | 0.6 | 5.0 | 47.5 | 7.1 | 16.1 |
| Maine | 2014 | 42.0 | 2.3 | 4.2 | 34.0 | 5.2 | 12.2 |
| Maine | 2015 | 57.3 | 1.5 | 4.9 | 27.6 | 3.4 | 5.3 |
| Maine | 2016 | 63.4 | 2.9 | 2.6 | 21.8 | 2.0 | 7.3 |
| Maine | 2017 | 71.8 | 2.7 | 2.4 | 13.8 | 2.4 | 7.0 |
| Maine | 2018 | 70.2 | 3.6 | 4.2 | 11.6 | 3.0 | 7.4 |
| Maine | 2019 | 78.4 | 3.0 | 2.2 | 8.2 | 3.3 | 4.9 |
| Maine | 2020 | 75.2 | 1.8 | 6.3 | 7.7 | 1.8 | 7.1 |
| Maryland | 2000 | 2.6 | 19.8 | 0.4 | 6.5 | 0.6 | 69.8 |
| Maryland | 2001 | 1.7 | 19.5 | 0.0 | 7.4 | 2.3 | 69.2 |
| Maryland | 2002 | 5.6 | 19.2 | 0.2 | 7.4 | 0.3 | 67.3 |
| Maryland | 2003 | 6.0 | 22.5 | 0.0 | 9.8 | 0.8 | 60.6 |
| Maryland | 2004 | 14.2 | 18.2 | 0.3 | 23.8 | 1.8 | 41.7 |
| Maryland | 2005 | 15.8 | 18.2 | 0.8 | 30.8 | 0.5 | 33.7 |
| Maryland | 2006 | 22.5 | 15.2 | 1.3 | 32.9 | 1.7 | 26.5 |
| Maryland | 2007 | 22.4 | 11.8 | 0.8 | 36.9 | 1.2 | 26.8 |
| Maryland | 2008 | 21.5 | 10.2 | 0.6 | 42.8 | 3.0 | 21.9 |
| Maryland | 2009 | 28.8 | 7.5 | 0.1 | 39.0 | 2.4 | 22.0 |
| Maryland | 2010 | 23.2 | 7.4 | 0.3 | 49.5 | 3.1 | 16.5 |
| Maryland | 2011 | 23.0 | 6.3 | 0.6 | 50.5 | 1.9 | 17.6 |
| Maryland | 2012 | 27.6 | 5.3 | 1.0 | 45.0 | 2.1 | 18.9 |
| Maryland | 2013 | 34.0 | 4.1 | 1.2 | 42.0 | 1.7 | 16.9 |
| Maryland | 2014 | 48.6 | 2.6 | 1.1 | 36.8 | 2.1 | 8.9 |
| Maryland | 2015 | 54.1 | 3.1 | 1.4 | 28.7 | 1.3 | 11.4 |
| Maryland | 2016 | 69.3 | 3.5 | 1.0 | 19.0 | 0.8 | 6.5 |
| Maryland | 2017 | 76.8 | 4.5 | 1.1 | 12.4 | 1.0 | 4.2 |
| Maryland | 2018 | 81.9 | 4.1 | 0.7 | 8.1 | 0.7 | 4.6 |
| Maryland | 2019 | 83.6 | 3.9 | 0.9 | 6.7 | 0.6 | 4.4 |
| Maryland | 2020 | 86.7 | 2.5 | 1.0 | 5.7 | 0.4 | 3.7 |
| Massachusetts | 2000 | 5.0 | 31.1 | 0.7 | 8.5 | 3.9 | 50.6 |
| Massachusetts | 2001 | 4.7 | 30.1 | 0.5 | 14.0 | 3.9 | 46.9 |
| Massachusetts | 2002 | 5.6 | 27.2 | 1.0 | 14.3 | 3.7 | 48.2 |
| Massachusetts | 2003 | 6.6 | 34.1 | 0.0 | 15.8 | 2.4 | 41.1 |
| Massachusetts | 2004 | 8.0 | 28.7 | 0.5 | 18.4 | 2.8 | 41.6 |
| Massachusetts | 2005 | 7.9 | 25.9 | 1.1 | 22.8 | 5.5 | 36.8 |
| Massachusetts | 2006 | 13.2 | 24.5 | 1.3 | 27.4 | 3.7 | 29.8 |
| Massachusetts | 2007 | 16.2 | 21.0 | 1.1 | 28.6 | 4.5 | 28.5 |
| Massachusetts | 2008 | 15.0 | 17.7 | 1.3 | 25.6 | 5.5 | 34.9 |
| Massachusetts | 2009 | 16.1 | 15.1 | 1.6 | 30.1 | 3.4 | 33.7 |
| Massachusetts | 2010 | 19.1 | 16.0 | 1.1 | 31.3 | 4.1 | 28.4 |
| Massachusetts | 2011 | 24.9 | 13.9 | 1.9 | 24.2 | 5.5 | 29.6 |
| Massachusetts | 2012 | 37.2 | 9.8 | 1.8 | 23.1 | 3.3 | 24.8 |
| Massachusetts | 2013 | 35.2 | 11.2 | 2.0 | 20.0 | 4.7 | 26.9 |
| Massachusetts | 2014 | 61.6 | 6.4 | 0.9 | 13.1 | 2.9 | 15.1 |
| Massachusetts | 2015 | 75.0 | 4.2 | 0.8 | 8.6 | 1.9 | 9.4 |
| Massachusetts | 2016 | 80.0 | 4.7 | 0.9 | 7.2 | 1.8 | 5.4 |
| Massachusetts | 2017 | 81.9 | 4.8 | 0.6 | 6.2 | 2.2 | 4.4 |
| Massachusetts | 2018 | 84.3 | 4.1 | 0.8 | 5.9 | 2.4 | 2.5 |
| Massachusetts | 2019 | 86.5 | 4.2 | 1.4 | 3.3 | 1.5 | 3.2 |
| Massachusetts | 2020 | 85.8 | 4.6 | 1.5 | 4.4 | 1.0 | 2.7 |
| Michigan | 2000 | 36.9 | 16.0 | 0.5 | 21.3 | 3.7 | 21.2 |
| Michigan | 2001 | 31.1 | 16.0 | 2.6 | 23.9 | 6.2 | 19.9 |
| Michigan | 2002 | 28.5 | 16.0 | 0.3 | 30.7 | 3.6 | 20.5 |
| Michigan | 2003 | 26.1 | 17.9 | 0.9 | 30.2 | 4.1 | 20.3 |
| Michigan | 2004 | 27.6 | 16.5 | 1.4 | 35.1 | 3.3 | 15.9 |
| Michigan | 2005 | 35.2 | 16.0 | 0.9 | 34.1 | 2.9 | 11.0 |
| Michigan | 2006 | 39.4 | 11.5 | 0.4 | 33.5 | 2.0 | 12.9 |
| Michigan | 2007 | 25.3 | 12.5 | 0.7 | 40.5 | 3.9 | 16.9 |
| Michigan | 2008 | 35.6 | 7.2 | 0.6 | 39.4 | 3.4 | 13.7 |
| Michigan | 2009 | 37.4 | 7.3 | 0.5 | 37.1 | 4.2 | 13.2 |
| Michigan | 2010 | 32.5 | 7.6 | 0.9 | 38.3 | 5.7 | 14.9 |
| Michigan | 2011 | 39.0 | 7.4 | 2.0 | 33.0 | 4.1 | 14.5 |
| Michigan | 2012 | 40.0 | 3.9 | 1.5 | 38.3 | 3.3 | 13.0 |
| Michigan | 2013 | 45.7 | 5.0 | 1.7 | 33.1 | 3.8 | 10.6 |
| Michigan | 2014 | 52.7 | 4.8 | 2.3 | 28.3 | 3.4 | 8.3 |
| Michigan | 2015 | 58.5 | 4.3 | 1.7 | 24.3 | 3.0 | 8.2 |
| Michigan | 2016 | 63.6 | 5.2 | 2.0 | 19.9 | 2.3 | 7.0 |
| Michigan | 2017 | 69.5 | 5.7 | 2.1 | 14.5 | 2.2 | 6.1 |
| Michigan | 2018 | 71.2 | 5.5 | 2.8 | 12.5 | 2.3 | 5.7 |
| Michigan | 2019 | 72.5 | 5.7 | 3.9 | 10.7 | 1.6 | 5.7 |
| Michigan | 2020 | 77.7 | 3.5 | 4.3 | 7.9 | 2.0 | 4.7 |
| Minnesota | 2000 | 9.6 | 9.6 | 6.5 | 20.3 | 9.4 | 43.7 |
| Minnesota | 2001 | 12.4 | 6.1 | 3.2 | 36.0 | 6.0 | 35.7 |
| Minnesota | 2002 | 6.9 | 10.0 | 7.0 | 35.6 | 6.7 | 32.7 |
| Minnesota | 2003 | 10.2 | 11.5 | 7.1 | 33.1 | 3.4 | 34.3 |
| Minnesota | 2004 | 9.0 | 11.7 | 4.2 | 41.8 | 4.5 | 28.9 |
| Minnesota | 2005 | 10.0 | 8.7 | 9.1 | 39.0 | 6.1 | 26.8 |
| Minnesota | 2006 | 12.8 | 9.0 | 5.6 | 41.8 | 3.9 | 25.8 |
| Minnesota | 2007 | 12.5 | 13.2 | 3.1 | 47.3 | 2.7 | 21.0 |
| Minnesota | 2008 | 13.1 | 5.8 | 4.6 | 42.7 | 4.4 | 29.4 |
| Minnesota | 2009 | 16.3 | 4.5 | 2.4 | 48.7 | 5.2 | 23.0 |
| Minnesota | 2010 | 17.0 | 5.8 | 5.0 | 44.8 | 3.2 | 23.9 |
| Minnesota | 2011 | 18.0 | 7.3 | 6.3 | 35.5 | 6.0 | 26.9 |
| Minnesota | 2012 | 21.2 | 5.1 | 11.4 | 35.0 | 2.2 | 24.9 |
| Minnesota | 2013 | 28.9 | 4.3 | 7.3 | 38.2 | 3.3 | 17.9 |
| Minnesota | 2014 | 31.8 | 3.9 | 10.7 | 33.8 | 2.1 | 17.8 |
| Minnesota | 2015 | 32.5 | 4.6 | 12.0 | 30.6 | 3.9 | 16.4 |
| Minnesota | 2016 | 38.5 | 3.7 | 17.3 | 25.0 | 3.0 | 12.2 |
| Minnesota | 2017 | 40.4 | 4.9 | 19.1 | 23.2 | 1.5 | 10.9 |
| Minnesota | 2018 | 46.3 | 3.9 | 20.1 | 15.5 | 2.3 | 12.0 |
| Minnesota | 2019 | 50.2 | 2.7 | 20.2 | 11.8 | 3.8 | 11.3 |
| Minnesota | 2020 | 63.0 | 2.9 | 16.8 | 8.7 | 2.0 | 6.6 |
| Mississippi | 2000 | 15.8 | 11.2 | 3.4 | 13.7 | 17.5 | 35.6 |
| Mississippi | 2001 | 8.1 | 13.0 | 3.7 | 35.0 | 9.5 | 30.7 |
| Mississippi | 2002 | 4.6 | 19.3 | 0.0 | 28.9 | 5.0 | 42.2 |
| Mississippi | 2003 | 11.9 | 17.5 | 4.3 | 38.5 | 7.6 | 19.8 |
| Mississippi | 2004 | 8.4 | 28.1 | 4.9 | 31.6 | 2.7 | 23.0 |
| Mississippi | 2005 | 4.1 | 22.6 | 4.8 | 37.2 | 5.4 | 23.9 |
| Mississippi | 2006 | 12.1 | 30.5 | 2.5 | 39.8 | 1.9 | 12.4 |
| Mississippi | 2007 | 18.1 | 10.5 | 2.8 | 45.9 | 7.8 | 14.0 |
| Mississippi | 2008 | 28.9 | 7.4 | 3.9 | 48.4 | 2.6 | 8.2 |
| Mississippi | 2009 | 27.5 | 11.0 | 4.4 | 38.4 | 5.9 | 12.6 |
| Mississippi | 2010 | 18.2 | 8.1 | 3.5 | 48.5 | 3.3 | 18.2 |
| Mississippi | 2011 | 15.8 | 10.9 | 3.3 | 43.5 | 11.2 | 14.7 |
| Mississippi | 2012 | 21.7 | 4.6 | 2.0 | 54.9 | 6.2 | 10.3 |
| Mississippi | 2013 | 24.4 | 7.0 | 6.0 | 41.3 | 4.2 | 16.8 |
| Mississippi | 2014 | 27.2 | 6.0 | 11.8 | 33.7 | 3.2 | 17.9 |
| Mississippi | 2015 | 31.7 | 7.0 | 19.0 | 28.3 | 1.0 | 12.9 |
| Mississippi | 2016 | 28.3 | 5.0 | 17.8 | 28.9 | 7.4 | 12.7 |
| Mississippi | 2017 | 37.6 | 5.3 | 15.7 | 20.7 | 3.6 | 17.1 |
| Mississippi | 2018 | 37.2 | 4.5 | 26.1 | 19.3 | 4.2 | 8.7 |
| Mississippi | 2019 | 49.0 | 5.0 | 21.4 | 14.0 | 3.0 | 7.6 |
| Mississippi | 2020 | 62.0 | 3.9 | 19.0 | 7.6 | 2.3 | 5.0 |
| Missouri | 2000 | 30.8 | 12.8 | 4.2 | 22.0 | 7.3 | 22.9 |
| Missouri | 2001 | 23.2 | 11.7 | 4.3 | 24.3 | 7.1 | 28.8 |
| Missouri | 2002 | 27.0 | 11.8 | 4.5 | 32.8 | 4.2 | 19.5 |
| Missouri | 2003 | 19.3 | 11.2 | 5.1 | 46.9 | 1.8 | 15.4 |
| Missouri | 2004 | 24.2 | 11.8 | 5.7 | 35.3 | 4.4 | 18.6 |
| Missouri | 2005 | 20.6 | 11.0 | 5.7 | 42.2 | 4.7 | 15.6 |
| Missouri | 2006 | 32.2 | 12.2 | 4.6 | 34.7 | 4.5 | 11.8 |
| Missouri | 2007 | 24.5 | 10.2 | 3.5 | 40.1 | 6.8 | 14.9 |
| Missouri | 2008 | 28.6 | 5.8 | 3.3 | 42.7 | 5.9 | 13.5 |
| Missouri | 2009 | 34.0 | 5.4 | 3.2 | 34.6 | 5.6 | 17.0 |
| Missouri | 2010 | 34.6 | 4.2 | 5.6 | 36.3 | 4.7 | 14.6 |
| Missouri | 2011 | 41.1 | 3.5 | 4.7 | 32.6 | 5.0 | 13.2 |
| Missouri | 2012 | 36.6 | 3.2 | 7.2 | 31.5 | 4.5 | 16.9 |
| Missouri | 2013 | 43.2 | 3.1 | 6.8 | 29.4 | 4.3 | 13.1 |
| Missouri | 2014 | 47.7 | 2.3 | 8.3 | 28.0 | 3.0 | 10.6 |
| Missouri | 2015 | 48.9 | 2.6 | 11.2 | 23.5 | 3.6 | 10.2 |
| Missouri | 2016 | 58.6 | 2.9 | 11.0 | 16.7 | 2.3 | 8.4 |
| Missouri | 2017 | 60.5 | 3.5 | 12.2 | 13.6 | 2.6 | 7.6 |
| Missouri | 2018 | 64.5 | 2.5 | 13.2 | 11.2 | 2.4 | 6.3 |
| Missouri | 2019 | 64.1 | 2.8 | 17.9 | 8.9 | 1.1 | 5.2 |
| Missouri | 2020 | 71.6 | 2.5 | 13.5 | 6.3 | 1.3 | 4.8 |
| Montana | 2000 | 6.7 | 0.0 | 2.8 | 57.9 | 0.0 | 22.8 |
| Montana | 2001 | 10.4 | 3.2 | 5.4 | 57.2 | 6.8 | 11.6 |
| Montana | 2002 | 0.0 | 4.5 | 3.6 | 55.7 | 4.5 | 28.9 |
| Montana | 2003 | 15.7 | 1.5 | 3.7 | 56.3 | 3.1 | 19.6 |
| Montana | 2004 | 24.9 | 1.9 | 1.9 | 56.4 | 1.9 | 11.0 |
| Montana | 2005 | 14.5 | 3.7 | 3.6 | 59.5 | 3.2 | 15.5 |
| Montana | 2006 | 9.3 | 3.0 | 5.9 | 53.9 | 5.7 | 22.3 |
| Montana | 2007 | 15.6 | 1.8 | 4.6 | 57.9 | 3.8 | 13.9 |
| Montana | 2008 | 20.4 | 0.0 | 3.6 | 60.1 | 0.0 | 15.1 |
| Montana | 2009 | 18.9 | 1.9 | 2.0 | 62.3 | 1.9 | 10.9 |
| Montana | 2010 | 11.6 | 1.6 | 7.8 | 56.4 | 1.3 | 21.3 |
| Montana | 2011 | 15.1 | 0.9 | 2.3 | 50.2 | 1.1 | 28.3 |
| Montana | 2012 | 23.3 | 0.0 | 5.8 | 46.1 | 2.6 | 18.7 |
| Montana | 2013 | 12.3 | 0.0 | 15.2 | 49.4 | 3.9 | 19.1 |
| Montana | 2014 | 22.9 | 0.0 | 7.6 | 38.8 | 1.6 | 29.1 |
| Montana | 2015 | 14.9 | 0.0 | 35.2 | 29.0 | 1.2 | 19.7 |
| Montana | 2016 | 29.3 | 0.0 | 25.3 | 26.3 | 1.0 | 18.1 |
| Montana | 2017 | 16.2 | 1.2 | 26.1 | 26.3 | 5.9 | 24.2 |
| Montana | 2018 | 32.9 | 1.2 | 19.1 | 23.6 | 2.7 | 20.5 |
| Montana | 2019 | 33.6 | 0.8 | 34.9 | 15.9 | 2.3 | 12.5 |
| Montana | 2020 | 36.4 | 2.0 | 29.1 | 17.0 | 2.8 | 12.6 |
| Nebraska | 2000 | 23.8 | 12.3 | 7.1 | 26.7 | 0.0 | 30.0 |
| Nebraska | 2001 | 24.0 | 4.9 | 12.0 | 28.1 | 6.2 | 24.7 |
| Nebraska | 2002 | 21.6 | 8.8 | 12.8 | 29.2 | 0.0 | 27.5 |
| Nebraska | 2003 | 12.7 | 10.5 | 3.0 | 28.8 | 3.1 | 38.3 |
| Nebraska | 2004 | 15.4 | 7.4 | 17.2 | 46.1 | 0.0 | 13.8 |
| Nebraska | 2005 | 16.9 | 2.8 | 7.3 | 52.3 | 2.2 | 17.2 |
| Nebraska | 2006 | 19.2 | 12.6 | 5.1 | 40.3 | 8.4 | 13.2 |
| Nebraska | 2007 | 9.4 | 1.7 | 6.0 | 53.2 | 5.3 | 23.2 |
| Nebraska | 2008 | 11.5 | 6.3 | 1.5 | 45.3 | 6.8 | 27.4 |
| Nebraska | 2009 | 16.1 | 1.0 | 8.3 | 56.2 | 1.4 | 16.0 |
| Nebraska | 2010 | 27.7 | 0.9 | 5.6 | 45.9 | 3.0 | 15.1 |
| Nebraska | 2011 | 10.6 | 2.7 | 16.8 | 47.9 | 4.3 | 16.2 |
| Nebraska | 2012 | 13.1 | 3.4 | 15.7 | 36.5 | 6.1 | 23.8 |
| Nebraska | 2013 | 10.5 | 1.7 | 15.9 | 42.6 | 7.6 | 19.1 |
| Nebraska | 2014 | 18.9 | 0.8 | 17.2 | 39.9 | 3.0 | 20.2 |
| Nebraska | 2015 | 20.4 | 2.0 | 26.8 | 30.5 | 4.8 | 14.6 |
| Nebraska | 2016 | 20.3 | 6.9 | 17.0 | 24.4 | 7.7 | 22.8 |
| Nebraska | 2017 | 25.1 | 0.8 | 28.3 | 25.3 | 4.0 | 15.8 |
| Nebraska | 2018 | 30.2 | 0.7 | 24.3 | 21.9 | 9.5 | 12.6 |
| Nebraska | 2019 | 28.8 | 2.7 | 28.9 | 21.3 | 4.7 | 13.1 |
| Nebraska | 2020 | 42.6 | 2.1 | 24.8 | 6.5 | 7.1 | 17.0 |
| Nevada | 2000 | 20.6 | 10.0 | 8.0 | 39.0 | 9.6 | 12.7 |
| Nevada | 2001 | 17.8 | 10.3 | 6.0 | 42.4 | 3.6 | 19.9 |
| Nevada | 2002 | 21.1 | 8.8 | 12.8 | 35.3 | 7.7 | 13.9 |
| Nevada | 2003 | 16.4 | 13.1 | 10.9 | 39.3 | 5.0 | 15.1 |
| Nevada | 2004 | 14.0 | 8.6 | 17.7 | 39.0 | 3.7 | 17.1 |
| Nevada | 2005 | 17.0 | 9.3 | 12.7 | 44.6 | 4.2 | 12.2 |
| Nevada | 2006 | 14.0 | 10.1 | 13.5 | 46.9 | 4.8 | 10.8 |
| Nevada | 2007 | 13.7 | 8.2 | 9.3 | 56.0 | 3.4 | 9.1 |
| Nevada | 2008 | 11.7 | 6.9 | 9.8 | 54.8 | 4.5 | 12.2 |
| Nevada | 2009 | 12.6 | 4.2 | 12.4 | 58.2 | 3.4 | 9.3 |
| Nevada | 2010 | 10.5 | 4.0 | 13.0 | 58.6 | 4.3 | 9.6 |
| Nevada | 2011 | 14.2 | 3.2 | 16.9 | 49.3 | 5.6 | 10.8 |
| Nevada | 2012 | 12.4 | 3.5 | 15.8 | 53.8 | 5.2 | 9.4 |
| Nevada | 2013 | 13.0 | 4.6 | 22.1 | 43.6 | 5.2 | 11.5 |
| Nevada | 2014 | 18.7 | 1.3 | 21.3 | 44.0 | 4.1 | 10.5 |
| Nevada | 2015 | 19.3 | 3.5 | 23.6 | 42.0 | 3.4 | 8.1 |
| Nevada | 2016 | 21.4 | 2.8 | 29.4 | 32.6 | 5.2 | 8.6 |
| Nevada | 2017 | 23.1 | 2.4 | 30.2 | 32.7 | 3.7 | 7.9 |
| Nevada | 2018 | 27.1 | 3.1 | 36.1 | 23.5 | 3.3 | 6.9 |
| Nevada | 2019 | 35.8 | 2.6 | 33.1 | 19.0 | 2.7 | 6.9 |
| Nevada | 2020 | 49.2 | 1.9 | 27.6 | 14.6 | 2.8 | 3.8 |
| New Hampshire | 2000 | 7.0 | 14.3 | 0.0 | 35.4 | 12.8 | 30.6 |
| New Hampshire | 2001 | 8.1 | 10.7 | 1.3 | 36.6 | 6.8 | 36.5 |
| New Hampshire | 2002 | 7.8 | 12.3 | 1.9 | 40.3 | 6.8 | 30.9 |
| New Hampshire | 2003 | 10.0 | 13.1 | 1.7 | 42.3 | 6.2 | 26.8 |
| New Hampshire | 2004 | 14.0 | 16.6 | 0.0 | 40.5 | 4.1 | 24.8 |
| New Hampshire | 2005 | 12.9 | 16.2 | 2.3 | 46.6 | 5.9 | 16.2 |
| New Hampshire | 2006 | 12.1 | 16.4 | 1.4 | 43.6 | 7.1 | 19.3 |
| New Hampshire | 2007 | 14.1 | 7.5 | 1.7 | 55.5 | 4.7 | 16.6 |
| New Hampshire | 2008 | 19.9 | 7.6 | 3.4 | 48.3 | 3.2 | 17.6 |
| New Hampshire | 2009 | 28.4 | 6.3 | 0.0 | 45.1 | 7.6 | 12.6 |
| New Hampshire | 2010 | 22.4 | 5.0 | 0.7 | 49.0 | 6.7 | 16.3 |
| New Hampshire | 2011 | 33.1 | 1.5 | 2.0 | 38.7 | 2.5 | 22.3 |
| New Hampshire | 2012 | 37.5 | 3.0 | 2.4 | 35.7 | 8.3 | 13.1 |
| New Hampshire | 2013 | 49.0 | 5.1 | 3.1 | 28.1 | 4.6 | 10.2 |
| New Hampshire | 2014 | 65.0 | 1.8 | 0.6 | 22.8 | 2.8 | 7.0 |
| New Hampshire | 2015 | 77.7 | 1.2 | 1.2 | 11.6 | 2.9 | 5.3 |
| New Hampshire | 2016 | 79.4 | 1.1 | 0.6 | 12.1 | 1.3 | 5.5 |
| New Hampshire | 2017 | 82.8 | 2.0 | 1.1 | 8.8 | 2.0 | 3.3 |
| New Hampshire | 2018 | 87.6 | 0.9 | 0.7 | 4.5 | 1.8 | 4.5 |
| New Hampshire | 2019 | 84.9 | 1.0 | 2.5 | 4.9 | 2.0 | 4.7 |
| New Hampshire | 2020 | 82.6 | 0.8 | 3.9 | 5.6 | 1.3 | 5.8 |
| New Jersey | 2000 | 47.1 | 13.4 | 0.7 | 14.9 | 4.0 | 20.0 |
| New Jersey | 2001 | 42.3 | 15.3 | 1.4 | 18.2 | 5.1 | 17.7 |
| New Jersey | 2002 | 41.2 | 16.0 | 0.8 | 17.6 | 5.3 | 19.1 |
| New Jersey | 2003 | 41.0 | 17.2 | 0.0 | 23.2 | 3.4 | 15.1 |
| New Jersey | 2004 | 32.7 | 18.8 | 1.4 | 27.0 | 4.7 | 15.4 |
| New Jersey | 2005 | 29.8 | 25.1 | 1.5 | 25.7 | 5.3 | 12.7 |
| New Jersey | 2006 | 28.4 | 19.2 | 1.0 | 32.3 | 3.5 | 15.5 |
| New Jersey | 2007 | 28.7 | 15.2 | 0.7 | 35.2 | 4.1 | 16.1 |
| New Jersey | 2008 | 29.5 | 14.8 | 0.9 | 33.4 | 4.0 | 17.4 |
| New Jersey | 2009 | 22.0 | 10.9 | 3.2 | 19.7 | 5.5 | 37.6 |
| New Jersey | 2010 | 26.4 | 13.1 | 1.3 | 38.4 | 4.8 | 15.7 |
| New Jersey | 2011 | 28.0 | 10.3 | 1.6 | 37.8 | 5.5 | 16.8 |
| New Jersey | 2012 | 44.6 | 9.1 | 1.3 | 29.1 | 4.6 | 11.4 |
| New Jersey | 2013 | 50.8 | 8.4 | 1.1 | 22.8 | 5.3 | 11.6 |
| New Jersey | 2014 | 57.7 | 5.3 | 1.2 | 22.6 | 4.4 | 8.8 |
| New Jersey | 2015 | 64.7 | 5.9 | 1.7 | 16.7 | 3.8 | 7.2 |
| New Jersey | 2016 | 73.6 | 4.6 | 1.4 | 13.3 | 2.4 | 4.6 |
| New Jersey | 2017 | 80.6 | 4.0 | 0.9 | 8.9 | 2.2 | 3.3 |
| New Jersey | 2018 | 84.5 | 3.9 | 0.9 | 5.9 | 1.8 | 3.0 |
| New Jersey | 2019 | 84.5 | 4.4 | 1.6 | 5.1 | 1.5 | 2.9 |
| New Jersey | 2020 | 85.2 | 3.6 | 1.7 | 5.3 | 1.6 | 2.5 |
| New Mexico | 2000 | 23.8 | 10.6 | 2.5 | 44.4 | 5.4 | 12.9 |
| New Mexico | 2001 | 16.8 | 12.6 | 3.8 | 45.6 | 5.4 | 15.8 |
| New Mexico | 2002 | 15.5 | 15.6 | 3.4 | 56.9 | 2.1 | 6.2 |
| New Mexico | 2003 | 14.4 | 13.6 | 7.4 | 49.8 | 1.3 | 13.2 |
| New Mexico | 2004 | 18.0 | 12.0 | 9.7 | 46.3 | 3.0 | 11.0 |
| New Mexico | 2005 | 18.0 | 13.9 | 11.6 | 45.8 | 3.1 | 7.5 |
| New Mexico | 2006 | 12.6 | 13.1 | 9.5 | 51.5 | 5.0 | 8.4 |
| New Mexico | 2007 | 13.3 | 13.5 | 9.7 | 52.1 | 2.8 | 8.7 |
| New Mexico | 2008 | 20.5 | 10.1 | 5.7 | 49.6 | 4.2 | 9.9 |
| New Mexico | 2009 | 25.9 | 11.9 | 9.3 | 40.5 | 4.9 | 7.6 |
| New Mexico | 2010 | 14.1 | 9.5 | 9.7 | 45.7 | 3.5 | 17.3 |
| New Mexico | 2011 | 25.5 | 7.1 | 10.5 | 42.1 | 3.8 | 10.9 |
| New Mexico | 2012 | 33.6 | 4.2 | 12.2 | 38.1 | 3.8 | 8.1 |
| New Mexico | 2013 | 25.4 | 4.4 | 14.4 | 44.5 | 4.7 | 6.6 |
| New Mexico | 2014 | 37.1 | 5.1 | 14.2 | 34.1 | 4.2 | 5.3 |
| New Mexico | 2015 | 41.2 | 3.3 | 16.4 | 27.2 | 5.9 | 6.0 |
| New Mexico | 2016 | 45.4 | 3.6 | 17.4 | 22.9 | 3.7 | 7.1 |
| New Mexico | 2017 | 41.2 | 4.2 | 21.2 | 23.5 | 2.7 | 7.2 |
| New Mexico | 2018 | 41.9 | 3.3 | 25.5 | 16.5 | 4.2 | 8.5 |
| New Mexico | 2019 | 50.2 | 4.1 | 25.7 | 13.4 | 2.3 | 4.4 |
| New Mexico | 2020 | 58.9 | 3.7 | 21.6 | 9.9 | 1.8 | 4.1 |
| New York | 2000 | 6.2 | 40.9 | 1.0 | 17.2 | 4.4 | 30.2 |
| New York | 2001 | 10.0 | 36.8 | 0.6 | 19.3 | 6.7 | 26.7 |
| New York | 2002 | 9.6 | 36.9 | 1.9 | 19.6 | 5.9 | 26.0 |
| New York | 2003 | 9.4 | 35.0 | 1.1 | 22.1 | 7.2 | 25.2 |
| New York | 2004 | 6.4 | 39.0 | 0.5 | 25.3 | 6.6 | 22.1 |
| New York | 2005 | 6.2 | 36.7 | 1.1 | 28.8 | 5.2 | 21.9 |
| New York | 2006 | 16.2 | 27.7 | 1.8 | 34.2 | 4.5 | 15.7 |
| New York | 2007 | 20.4 | 22.0 | 1.3 | 37.2 | 4.5 | 14.5 |
| New York | 2008 | 22.3 | 16.1 | 1.1 | 39.0 | 4.8 | 16.7 |
| New York | 2009 | 29.1 | 14.1 | 1.6 | 35.7 | 5.7 | 13.8 |
| New York | 2010 | 25.1 | 13.6 | 1.6 | 43.3 | 4.4 | 12.0 |
| New York | 2011 | 26.6 | 12.3 | 1.9 | 40.6 | 4.8 | 13.7 |
| New York | 2012 | 34.0 | 9.9 | 2.0 | 36.2 | 5.6 | 12.4 |
| New York | 2013 | 38.9 | 10.6 | 1.7 | 31.1 | 4.8 | 12.8 |
| New York | 2014 | 48.1 | 8.6 | 2.5 | 26.5 | 4.9 | 9.5 |
| New York | 2015 | 56.1 | 7.8 | 1.4 | 22.1 | 4.2 | 8.3 |
| New York | 2016 | 65.3 | 7.6 | 2.1 | 16.2 | 2.7 | 6.2 |
| New York | 2017 | 70.4 | 8.1 | 2.1 | 12.3 | 2.3 | 4.8 |
| New York | 2018 | 68.9 | 8.9 | 2.0 | 12.5 | 2.8 | 4.9 |
| New York | 2019 | 72.3 | 8.3 | 2.7 | 10.1 | 2.4 | 4.2 |
| New York | 2020 | 79.0 | 6.2 | 2.3 | 7.1 | 1.8 | 3.7 |
| North Carolina | 2000 | 20.6 | 15.1 | 0.2 | 42.9 | 2.6 | 18.5 |
| North Carolina | 2001 | 22.5 | 13.3 | 0.8 | 45.5 | 3.6 | 14.3 |
| North Carolina | 2002 | 16.5 | 14.0 | 1.5 | 48.7 | 2.7 | 16.4 |
| North Carolina | 2003 | 17.8 | 16.8 | 1.0 | 50.4 | 1.9 | 12.0 |
| North Carolina | 2004 | 20.2 | 15.1 | 1.6 | 48.6 | 1.8 | 12.6 |
| North Carolina | 2005 | 19.0 | 18.7 | 1.9 | 49.1 | 1.8 | 9.5 |
| North Carolina | 2006 | 16.5 | 19.9 | 0.9 | 52.3 | 1.6 | 8.8 |
| North Carolina | 2007 | 16.1 | 12.3 | 1.1 | 57.8 | 2.5 | 10.1 |
| North Carolina | 2008 | 17.3 | 8.1 | 0.9 | 58.2 | 3.1 | 12.4 |
| North Carolina | 2009 | 22.8 | 9.0 | 0.7 | 55.6 | 1.7 | 10.2 |
| North Carolina | 2010 | 21.6 | 7.3 | 1.2 | 56.1 | 3.4 | 10.6 |
| North Carolina | 2011 | 22.2 | 8.6 | 1.5 | 54.2 | 3.2 | 10.3 |
| North Carolina | 2012 | 26.5 | 8.8 | 2.3 | 47.8 | 2.6 | 11.9 |
| North Carolina | 2013 | 27.7 | 8.8 | 2.3 | 46.3 | 3.2 | 11.7 |
| North Carolina | 2014 | 36.8 | 8.0 | 1.9 | 39.5 | 2.8 | 11.0 |
| North Carolina | 2015 | 42.8 | 8.7 | 2.3 | 35.6 | 3.6 | 7.0 |
| North Carolina | 2016 | 51.3 | 9.2 | 3.0 | 27.0 | 2.3 | 7.2 |
| North Carolina | 2017 | 66.0 | 7.4 | 3.0 | 15.8 | 2.9 | 4.8 |
| North Carolina | 2018 | 66.8 | 8.2 | 4.2 | 12.6 | 2.2 | 5.9 |
| North Carolina | 2019 | 69.9 | 7.8 | 5.6 | 10.1 | 1.6 | 5.0 |
| North Carolina | 2020 | 78.1 | 5.7 | 4.9 | 6.8 | 1.4 | 3.1 |
| North Dakota | 2000 | 28.6 | 21.4 | 0.0 | 14.3 | 0.0 | 35.7 |
| North Dakota | 2001 | 27.9 | 0.0 | 10.7 | 47.1 | 0.0 | 14.3 |
| North Dakota | 2002 | 49.4 | 0.0 | 5.9 | 38.8 | 0.0 | 5.9 |
| North Dakota | 2003 | 33.3 | 0.0 | 12.1 | 13.6 | 4.5 | 27.3 |
| North Dakota | 2004 | 31.3 | 0.0 | 0.0 | 39.6 | 0.0 | 22.9 |
| North Dakota | 2005 | 25.0 | 0.0 | 0.0 | 50.0 | 8.3 | 16.7 |
| North Dakota | 2006 | 25.0 | 10.7 | 0.0 | 7.1 | 14.3 | 35.7 |
| North Dakota | 2007 | 9.7 | 0.0 | 0.0 | 32.3 | 9.7 | 41.9 |
| North Dakota | 2008 | 18.5 | 0.0 | 2.9 | 31.9 | 17.4 | 29.3 |
| North Dakota | 2009 | 23.5 | 0.0 | 4.9 | 19.4 | 17.3 | 34.9 |
| North Dakota | 2010 | 9.8 | 0.0 | 0.0 | 54.5 | 9.8 | 21.2 |
| North Dakota | 2011 | 20.5 | 0.0 | 6.3 | 26.8 | 6.3 | 40.2 |
| North Dakota | 2012 | 19.0 | 0.0 | 6.3 | 38.1 | 0.0 | 31.7 |
| North Dakota | 2013 | 13.5 | 6.0 | 5.0 | 42.0 | 0.0 | 33.5 |
| North Dakota | 2014 | 27.5 | 0.0 | 7.5 | 36.6 | 0.0 | 26.1 |
| North Dakota | 2015 | 19.3 | 0.0 | 16.7 | 18.7 | 11.5 | 33.8 |
| North Dakota | 2016 | 38.4 | 0.0 | 10.3 | 25.9 | 0.0 | 25.3 |
| North Dakota | 2017 | 26.6 | 1.8 | 25.2 | 21.9 | 1.6 | 22.8 |
| North Dakota | 2018 | 21.1 | 0.0 | 21.3 | 33.2 | 0.0 | 24.4 |
| North Dakota | 2019 | 20.6 | 1.2 | 22.2 | 32.6 | 1.4 | 20.8 |
| North Dakota | 2020 | 43.0 | 1.1 | 21.9 | 14.5 | 0.0 | 19.5 |
| Ohio | 2000 | 25.3 | 15.7 | 0.8 | 28.2 | 6.0 | 23.9 |
| Ohio | 2001 | 26.9 | 15.4 | 0.4 | 29.6 | 3.9 | 23.8 |
| Ohio | 2002 | 27.0 | 15.0 | 1.1 | 31.4 | 5.2 | 20.0 |
| Ohio | 2003 | 23.7 | 15.9 | 0.9 | 36.9 | 2.7 | 19.7 |
| Ohio | 2004 | 26.4 | 17.5 | 1.5 | 35.9 | 3.9 | 14.5 |
| Ohio | 2005 | 26.6 | 16.7 | 1.8 | 34.3 | 4.0 | 16.6 |
| Ohio | 2006 | 24.3 | 20.1 | 0.9 | 36.5 | 3.2 | 14.8 |
| Ohio | 2007 | 26.4 | 16.4 | 0.6 | 41.0 | 3.3 | 12.2 |
| Ohio | 2008 | 30.7 | 12.1 | 0.6 | 38.3 | 3.9 | 14.1 |
| Ohio | 2009 | 34.3 | 11.0 | 0.8 | 39.2 | 3.3 | 11.3 |
| Ohio | 2010 | 37.7 | 7.4 | 1.0 | 39.3 | 4.2 | 10.5 |
| Ohio | 2011 | 37.9 | 8.1 | 1.4 | 37.8 | 3.8 | 11.0 |
| Ohio | 2012 | 49.1 | 7.3 | 1.2 | 28.8 | 3.2 | 10.4 |
| Ohio | 2013 | 57.7 | 6.7 | 1.5 | 22.0 | 2.9 | 9.3 |
| Ohio | 2014 | 66.9 | 5.3 | 1.1 | 17.8 | 2.6 | 6.3 |
| Ohio | 2015 | 70.6 | 4.7 | 1.5 | 13.9 | 2.4 | 6.8 |
| Ohio | 2016 | 73.9 | 5.9 | 2.3 | 10.8 | 2.0 | 5.1 |
| Ohio | 2017 | 77.9 | 5.9 | 3.1 | 8.0 | 1.3 | 3.8 |
| Ohio | 2018 | 77.8 | 5.7 | 4.5 | 6.6 | 1.4 | 4.0 |
| Ohio | 2019 | 79.8 | 5.4 | 5.2 | 4.3 | 1.3 | 4.0 |
| Ohio | 2020 | 84.3 | 3.9 | 4.2 | 3.6 | 0.8 | 3.2 |
| Oklahoma | 2000 | 17.1 | 5.1 | 9.9 | 38.2 | 4.0 | 25.7 |
| Oklahoma | 2001 | 11.6 | 7.2 | 8.6 | 48.8 | 4.9 | 18.8 |
| Oklahoma | 2002 | 13.3 | 6.2 | 8.2 | 50.2 | 3.2 | 18.9 |
| Oklahoma | 2003 | 13.3 | 6.2 | 12.2 | 51.7 | 3.6 | 13.0 |
| Oklahoma | 2004 | 11.6 | 6.1 | 10.8 | 53.6 | 2.5 | 15.4 |
| Oklahoma | 2005 | 12.9 | 4.0 | 13.3 | 54.2 | 2.2 | 13.3 |
| Oklahoma | 2006 | 10.0 | 4.7 | 7.9 | 59.0 | 3.2 | 15.3 |
| Oklahoma | 2007 | 11.8 | 6.6 | 6.4 | 64.7 | 2.4 | 8.1 |
| Oklahoma | 2008 | 14.2 | 4.1 | 4.8 | 61.0 | 5.6 | 10.3 |
| Oklahoma | 2009 | 24.6 | 1.8 | 8.3 | 50.0 | 4.1 | 11.2 |
| Oklahoma | 2010 | 17.3 | 2.7 | 12.0 | 54.5 | 3.5 | 10.1 |
| Oklahoma | 2011 | 14.7 | 3.2 | 12.3 | 52.1 | 6.3 | 11.4 |
| Oklahoma | 2012 | 18.4 | 3.2 | 13.8 | 49.5 | 4.5 | 10.6 |
| Oklahoma | 2013 | 15.2 | 2.2 | 17.9 | 48.5 | 4.6 | 11.6 |
| Oklahoma | 2014 | 13.8 | 1.2 | 17.6 | 50.6 | 3.2 | 13.5 |
| Oklahoma | 2015 | 19.1 | 1.9 | 25.6 | 38.2 | 4.6 | 10.6 |
| Oklahoma | 2016 | 19.5 | 1.9 | 30.7 | 33.8 | 2.5 | 11.6 |
| Oklahoma | 2017 | 21.9 | 4.5 | 31.8 | 26.8 | 3.6 | 11.5 |
| Oklahoma | 2018 | 22.4 | 3.6 | 40.2 | 19.1 | 3.1 | 11.6 |
| Oklahoma | 2019 | 23.5 | 4.0 | 44.0 | 15.5 | 3.7 | 9.2 |
| Oklahoma | 2020 | 30.6 | 3.6 | 44.7 | 9.9 | 3.2 | 8.0 |
| Oregon | 2000 | 20.7 | 6.9 | 7.9 | 26.3 | 4.2 | 32.9 |
| Oregon | 2001 | 24.6 | 5.3 | 6.2 | 38.0 | 7.8 | 17.6 |
| Oregon | 2002 | 19.6 | 5.4 | 8.6 | 44.3 | 4.6 | 17.6 |
| Oregon | 2003 | 15.9 | 2.1 | 7.2 | 51.3 | 3.0 | 20.1 |
| Oregon | 2004 | 19.0 | 4.9 | 7.7 | 47.3 | 1.2 | 19.9 |
| Oregon | 2005 | 17.6 | 5.9 | 7.9 | 51.5 | 2.7 | 14.0 |
| Oregon | 2006 | 19.4 | 4.0 | 4.1 | 54.4 | 3.6 | 14.1 |
| Oregon | 2007 | 28.1 | 5.4 | 2.6 | 45.6 | 2.7 | 15.5 |
| Oregon | 2008 | 28.6 | 2.0 | 4.0 | 45.8 | 3.0 | 16.1 |
| Oregon | 2009 | 32.7 | 1.0 | 5.8 | 45.8 | 3.5 | 11.1 |
| Oregon | 2010 | 24.7 | 2.2 | 7.0 | 47.4 | 3.7 | 15.0 |
| Oregon | 2011 | 32.8 | 1.6 | 9.1 | 43.1 | 1.6 | 11.8 |
| Oregon | 2012 | 34.4 | 1.7 | 7.9 | 41.5 | 2.6 | 11.8 |
| Oregon | 2013 | 31.5 | 1.7 | 13.9 | 35.4 | 2.2 | 15.3 |
| Oregon | 2014 | 31.9 | 2.0 | 16.4 | 31.9 | 4.3 | 13.5 |
| Oregon | 2015 | 28.5 | 1.7 | 17.3 | 36.4 | 3.4 | 12.7 |
| Oregon | 2016 | 32.1 | 1.5 | 24.4 | 27.4 | 1.5 | 13.0 |
| Oregon | 2017 | 40.6 | 2.0 | 22.2 | 19.5 | 2.6 | 13.1 |
| Oregon | 2018 | 43.6 | 2.1 | 26.4 | 16.1 | 1.9 | 9.7 |
| Oregon | 2019 | 39.2 | 2.7 | 33.2 | 12.8 | 0.8 | 11.3 |
| Oregon | 2020 | 54.2 | 2.1 | 26.1 | 8.2 | 1.6 | 7.8 |
| Pennsylvania | 2000 | 36.0 | 16.4 | 0.4 | 17.0 | 3.5 | 25.6 |
| Pennsylvania | 2001 | 41.6 | 13.8 | 0.6 | 18.4 | 3.6 | 21.0 |
| Pennsylvania | 2002 | 34.8 | 19.8 | 0.5 | 20.6 | 4.1 | 20.2 |
| Pennsylvania | 2003 | 36.7 | 15.7 | 0.8 | 24.9 | 4.9 | 17.0 |
| Pennsylvania | 2004 | 32.4 | 16.1 | 1.6 | 30.2 | 3.7 | 15.6 |
| Pennsylvania | 2005 | 29.3 | 16.9 | 0.9 | 32.4 | 3.4 | 17.0 |
| Pennsylvania | 2006 | 28.8 | 22.7 | 1.0 | 29.5 | 2.3 | 15.2 |
| Pennsylvania | 2007 | 24.2 | 17.5 | 0.7 | 39.3 | 2.5 | 15.7 |
| Pennsylvania | 2008 | 30.4 | 15.4 | 1.2 | 34.6 | 3.7 | 14.5 |
| Pennsylvania | 2009 | 32.5 | 11.2 | 0.5 | 34.4 | 5.3 | 15.8 |
| Pennsylvania | 2010 | 26.2 | 11.9 | 2.0 | 39.3 | 4.8 | 15.8 |
| Pennsylvania | 2011 | 34.8 | 11.4 | 1.4 | 33.3 | 4.3 | 14.7 |
| Pennsylvania | 2012 | 37.8 | 9.3 | 1.9 | 32.4 | 4.1 | 14.6 |
| Pennsylvania | 2013 | 42.4 | 7.2 | 1.9 | 31.2 | 3.9 | 13.4 |
| Pennsylvania | 2014 | 47.8 | 7.6 | 1.7 | 27.1 | 3.9 | 11.9 |
| Pennsylvania | 2015 | 57.4 | 6.1 | 2.5 | 22.2 | 2.8 | 9.0 |
| Pennsylvania | 2016 | 69.0 | 5.4 | 1.9 | 16.2 | 2.4 | 5.1 |
| Pennsylvania | 2017 | 75.9 | 5.7 | 2.0 | 10.1 | 1.8 | 4.5 |
| Pennsylvania | 2018 | 76.2 | 7.1 | 2.8 | 7.3 | 1.9 | 4.6 |
| Pennsylvania | 2019 | 76.6 | 6.6 | 3.7 | 6.7 | 1.2 | 5.2 |
| Pennsylvania | 2020 | 81.5 | 4.3 | 4.5 | 5.4 | 1.0 | 3.3 |
| Rhode Island | 2000 | 5.5 | 27.4 | 0.0 | 22.2 | 2.7 | 42.2 |
| Rhode Island | 2001 | 3.1 | 26.6 | 2.0 | 20.2 | 4.1 | 44.0 |
| Rhode Island | 2002 | 9.6 | 25.3 | 1.1 | 23.6 | 4.3 | 36.1 |
| Rhode Island | 2003 | 9.7 | 26.8 | 0.7 | 22.7 | 3.0 | 37.0 |
| Rhode Island | 2004 | 11.2 | 20.7 | 0.0 | 24.2 | 4.9 | 38.9 |
| Rhode Island | 2005 | 10.1 | 25.6 | 0.0 | 29.9 | 5.4 | 28.4 |
| Rhode Island | 2006 | 8.2 | 27.3 | 1.8 | 36.7 | 7.1 | 18.9 |
| Rhode Island | 2007 | 5.0 | 21.3 | 0.0 | 37.5 | 8.2 | 28.1 |
| Rhode Island | 2008 | 9.5 | 15.2 | 1.7 | 30.5 | 11.1 | 32.0 |
| Rhode Island | 2009 | 7.4 | 18.7 | 1.4 | 38.3 | 10.7 | 23.4 |
| Rhode Island | 2010 | 9.5 | 17.4 | 1.2 | 40.1 | 12.2 | 19.5 |
| Rhode Island | 2011 | 10.9 | 12.0 | 1.6 | 44.7 | 12.7 | 18.0 |
| Rhode Island | 2012 | 22.0 | 14.2 | 4.3 | 41.7 | 4.3 | 13.0 |
| Rhode Island | 2013 | 39.9 | 11.4 | 0.8 | 29.3 | 4.2 | 14.4 |
| Rhode Island | 2014 | 54.8 | 6.6 | 0.8 | 22.2 | 5.3 | 10.2 |
| Rhode Island | 2015 | 54.9 | 7.4 | 2.3 | 23.2 | 4.2 | 7.9 |
| Rhode Island | 2016 | 61.0 | 10.8 | 2.2 | 18.0 | 1.9 | 6.2 |
| Rhode Island | 2017 | 65.8 | 7.3 | 0.9 | 18.6 | 1.9 | 5.4 |
| Rhode Island | 2018 | 70.4 | 7.5 | 2.3 | 14.3 | 2.6 | 2.9 |
| Rhode Island | 2019 | 69.6 | 13.2 | 2.0 | 8.0 | 2.4 | 4.7 |
| Rhode Island | 2020 | 74.4 | 9.3 | 2.3 | 9.1 | 1.6 | 3.4 |
| South Carolina | 2000 | 17.8 | 19.4 | 3.5 | 28.6 | 6.3 | 24.3 |
| South Carolina | 2001 | 8.4 | 19.3 | 2.6 | 46.6 | 4.9 | 18.1 |
| South Carolina | 2002 | 17.8 | 21.1 | 2.2 | 31.0 | 6.4 | 21.4 |
| South Carolina | 2003 | 14.9 | 25.9 | 3.8 | 36.4 | 4.5 | 14.1 |
| South Carolina | 2004 | 17.2 | 20.1 | 3.3 | 40.3 | 3.0 | 16.1 |
| South Carolina | 2005 | 13.3 | 22.4 | 2.4 | 43.6 | 2.7 | 15.6 |
| South Carolina | 2006 | 12.0 | 20.7 | 1.9 | 44.7 | 4.2 | 16.5 |
| South Carolina | 2007 | 12.7 | 18.9 | 2.3 | 43.8 | 5.3 | 17.1 |
| South Carolina | 2008 | 16.2 | 16.4 | 2.5 | 46.9 | 4.8 | 13.2 |
| South Carolina | 2009 | 21.5 | 12.0 | 4.7 | 44.3 | 5.5 | 12.1 |
| South Carolina | 2010 | 18.2 | 9.1 | 2.3 | 47.9 | 5.4 | 16.9 |
| South Carolina | 2011 | 17.3 | 10.4 | 3.5 | 50.4 | 3.9 | 14.6 |
| South Carolina | 2012 | 19.0 | 9.1 | 5.2 | 45.9 | 3.1 | 17.6 |
| South Carolina | 2013 | 23.6 | 7.6 | 7.0 | 45.1 | 2.8 | 13.9 |
| South Carolina | 2014 | 26.0 | 4.5 | 8.1 | 47.0 | 4.8 | 9.5 |
| South Carolina | 2015 | 33.3 | 6.9 | 8.4 | 38.0 | 5.8 | 7.5 |
| South Carolina | 2016 | 39.2 | 7.0 | 9.9 | 29.4 | 5.3 | 9.2 |
| South Carolina | 2017 | 51.4 | 7.6 | 10.6 | 20.9 | 3.4 | 5.9 |
| South Carolina | 2018 | 54.6 | 6.8 | 11.5 | 17.3 | 2.2 | 7.5 |
| South Carolina | 2019 | 59.8 | 5.6 | 12.1 | 14.2 | 2.6 | 5.8 |
| South Carolina | 2020 | 73.1 | 4.9 | 9.3 | 7.7 | 1.1 | 3.8 |
| South Dakota | 2000 | 13.2 | 0.0 | 0.0 | 6.6 | 0.0 | 75.0 |
| South Dakota | 2001 | 18.6 | 4.3 | 4.3 | 26.7 | 13.7 | 28.0 |
| South Dakota | 2002 | 56.8 | 0.0 | 6.8 | 15.9 | 0.0 | 20.5 |
| South Dakota | 2003 | 22.5 | 0.0 | 6.3 | 25.8 | 0.0 | 45.4 |
| South Dakota | 2004 | 17.9 | 4.8 | 0.0 | 33.0 | 4.2 | 37.9 |
| South Dakota | 2005 | 23.9 | 6.0 | 8.5 | 38.5 | 17.9 | 2.6 |
| South Dakota | 2006 | 2.7 | 0.0 | 3.2 | 67.1 | 10.8 | 16.2 |
| South Dakota | 2007 | 24.5 | 7.5 | 8.3 | 34.4 | 0.0 | 25.2 |
| South Dakota | 2008 | 17.6 | 6.9 | 2.8 | 55.7 | 5.0 | 12.0 |
| South Dakota | 2009 | 31.4 | 0.0 | 2.0 | 47.9 | 0.0 | 18.6 |
| South Dakota | 2010 | 15.7 | 0.0 | 4.8 | 58.6 | 11.0 | 10.0 |
| South Dakota | 2011 | 20.8 | 3.6 | 9.9 | 41.3 | 1.9 | 22.5 |
| South Dakota | 2012 | 14.6 | 0.0 | 10.0 | 34.9 | 7.3 | 30.7 |
| South Dakota | 2013 | 13.9 | 0.0 | 17.3 | 54.8 | 3.8 | 10.2 |
| South Dakota | 2014 | 14.4 | 0.0 | 21.7 | 36.7 | 3.3 | 23.9 |
| South Dakota | 2015 | 23.6 | 0.0 | 20.4 | 20.8 | 3.6 | 31.5 |
| South Dakota | 2016 | 27.1 | 0.0 | 22.9 | 34.6 | 3.1 | 10.9 |
| South Dakota | 2017 | 29.4 | 1.4 | 29.8 | 15.6 | 1.5 | 22.4 |
| South Dakota | 2018 | 32.2 | 7.4 | 20.7 | 19.6 | 1.8 | 18.3 |
| South Dakota | 2019 | 30.4 | 2.7 | 36.9 | 13.3 | 3.6 | 13.3 |
| South Dakota | 2020 | 48.8 | 2.5 | 28.2 | 10.1 | 1.2 | 9.1 |
| Tennessee | 2000 | 9.6 | 18.7 | 3.8 | 30.5 | 8.2 | 28.9 |
| Tennessee | 2001 | 5.6 | 16.6 | 1.6 | 38.5 | 9.2 | 28.3 |
| Tennessee | 2002 | 8.6 | 18.7 | 3.9 | 37.8 | 9.4 | 21.6 |
| Tennessee | 2003 | 14.0 | 15.4 | 2.8 | 41.9 | 5.5 | 20.2 |
| Tennessee | 2004 | 13.8 | 16.6 | 2.5 | 47.2 | 3.9 | 16.0 |
| Tennessee | 2005 | 13.6 | 16.7 | 2.8 | 47.1 | 4.8 | 14.9 |
| Tennessee | 2006 | 9.8 | 18.9 | 2.0 | 51.1 | 5.2 | 13.0 |
| Tennessee | 2007 | 12.5 | 17.7 | 2.7 | 48.0 | 6.8 | 12.1 |
| Tennessee | 2008 | 15.6 | 10.7 | 2.5 | 48.7 | 7.2 | 15.2 |
| Tennessee | 2009 | 13.4 | 11.1 | 2.6 | 52.2 | 6.3 | 14.3 |
| Tennessee | 2010 | 10.7 | 9.0 | 4.5 | 57.6 | 4.1 | 13.9 |
| Tennessee | 2011 | 9.8 | 8.8 | 5.3 | 55.8 | 5.1 | 15.1 |
| Tennessee | 2012 | 13.3 | 6.3 | 4.4 | 54.3 | 5.4 | 16.2 |
| Tennessee | 2013 | 17.1 | 6.7 | 6.7 | 51.5 | 4.2 | 13.8 |
| Tennessee | 2014 | 24.7 | 4.5 | 5.2 | 47.6 | 4.6 | 13.4 |
| Tennessee | 2015 | 31.5 | 5.9 | 6.0 | 41.8 | 3.5 | 11.2 |
| Tennessee | 2016 | 38.8 | 6.1 | 7.5 | 37.4 | 3.7 | 6.6 |
| Tennessee | 2017 | 46.2 | 6.3 | 11.4 | 25.9 | 3.6 | 6.6 |
| Tennessee | 2018 | 55.0 | 4.1 | 13.8 | 16.8 | 2.8 | 7.5 |
| Tennessee | 2019 | 62.3 | 4.7 | 14.1 | 11.3 | 1.5 | 6.1 |
| Tennessee | 2020 | 74.7 | 3.1 | 10.5 | 6.5 | 1.4 | 3.7 |
| Texas | 2000 | 21.5 | 22.6 | 3.6 | 24.6 | 4.8 | 22.7 |
| Texas | 2001 | 24.8 | 21.3 | 4.0 | 27.3 | 3.3 | 19.2 |
| Texas | 2002 | 23.8 | 20.5 | 3.8 | 31.6 | 4.0 | 16.2 |
| Texas | 2003 | 18.4 | 22.8 | 5.5 | 33.7 | 3.6 | 16.0 |
| Texas | 2004 | 20.5 | 23.6 | 5.5 | 31.4 | 3.9 | 15.1 |
| Texas | 2005 | 18.7 | 21.9 | 8.4 | 34.1 | 3.0 | 13.9 |
| Texas | 2006 | 17.6 | 24.4 | 5.7 | 38.3 | 3.3 | 10.6 |
| Texas | 2007 | 19.2 | 22.2 | 4.5 | 36.3 | 6.0 | 11.9 |
| Texas | 2008 | 22.5 | 18.4 | 5.3 | 35.7 | 4.1 | 14.0 |
| Texas | 2009 | 26.3 | 14.7 | 5.6 | 34.3 | 3.8 | 15.3 |
| Texas | 2010 | 23.9 | 13.8 | 7.7 | 35.2 | 4.3 | 15.2 |
| Texas | 2011 | 25.2 | 14.8 | 8.1 | 32.0 | 3.8 | 16.0 |
| Texas | 2012 | 26.4 | 14.4 | 9.9 | 29.8 | 4.0 | 15.4 |
| Texas | 2013 | 26.1 | 13.4 | 15.2 | 25.8 | 4.2 | 15.3 |
| Texas | 2014 | 29.3 | 12.9 | 14.7 | 23.7 | 4.7 | 14.8 |
| Texas | 2015 | 32.7 | 13.5 | 16.8 | 21.7 | 4.1 | 11.1 |
| Texas | 2016 | 31.6 | 13.9 | 18.5 | 20.0 | 4.0 | 11.9 |
| Texas | 2017 | 32.7 | 15.5 | 18.6 | 18.5 | 3.8 | 10.9 |
| Texas | 2018 | 35.1 | 15.5 | 22.1 | 14.5 | 3.0 | 9.8 |
| Texas | 2019 | 37.4 | 13.7 | 25.8 | 10.9 | 2.2 | 9.9 |
| Texas | 2020 | 44.2 | 13.0 | 25.6 | 8.3 | 2.0 | 6.9 |
| Utah | 2000 | 28.6 | 7.5 | 5.0 | 46.0 | 2.5 | 10.4 |
| Utah | 2001 | 21.7 | 6.6 | 5.6 | 51.1 | 2.1 | 12.8 |
| Utah | 2002 | 15.0 | 6.4 | 8.2 | 56.1 | 4.0 | 10.4 |
| Utah | 2003 | 17.0 | 7.1 | 11.9 | 53.8 | 2.0 | 8.2 |
| Utah | 2004 | 10.4 | 6.9 | 12.2 | 60.1 | 1.5 | 8.9 |
| Utah | 2005 | 21.0 | 5.8 | 8.3 | 57.3 | 1.8 | 5.5 |
| Utah | 2006 | 20.5 | 4.2 | 10.5 | 54.7 | 2.8 | 7.1 |
| Utah | 2007 | 18.5 | 4.3 | 8.5 | 52.5 | 3.2 | 13.0 |
| Utah | 2008 | 23.0 | 3.0 | 8.3 | 54.7 | 3.4 | 7.6 |
| Utah | 2009 | 24.4 | 3.9 | 9.0 | 48.3 | 4.6 | 9.8 |
| Utah | 2010 | 25.9 | 2.2 | 8.1 | 48.0 | 3.1 | 12.8 |
| Utah | 2011 | 27.1 | 1.6 | 8.8 | 46.5 | 5.5 | 10.5 |
| Utah | 2012 | 25.0 | 3.1 | 11.5 | 44.6 | 4.5 | 11.3 |
| Utah | 2013 | 31.3 | 1.4 | 12.0 | 40.4 | 4.1 | 10.8 |
| Utah | 2014 | 29.9 | 1.2 | 13.9 | 43.0 | 3.3 | 8.6 |
| Utah | 2015 | 30.2 | 1.6 | 16.5 | 37.1 | 4.1 | 10.5 |
| Utah | 2016 | 39.4 | 1.2 | 13.2 | 32.9 | 4.1 | 9.3 |
| Utah | 2017 | 36.2 | 1.6 | 19.5 | 29.4 | 4.5 | 8.6 |
| Utah | 2018 | 37.9 | 2.2 | 22.7 | 26.7 | 3.8 | 6.8 |
| Utah | 2019 | 40.8 | 1.8 | 23.6 | 25.2 | 2.4 | 6.3 |
| Utah | 2020 | 47.1 | 1.8 | 19.8 | 21.1 | 2.7 | 7.4 |
| Vermont | 2000 | 27.6 | 10.0 | 0.0 | 30.7 | 6.3 | 25.4 |
| Vermont | 2001 | 26.8 | 7.3 | 0.0 | 34.1 | 4.9 | 26.8 |
| Vermont | 2002 | 24.4 | 11.1 | 4.4 | 40.0 | 4.4 | 15.6 |
| Vermont | 2003 | 21.3 | 1.7 | 1.6 | 50.8 | 5.0 | 19.7 |
| Vermont | 2004 | 35.0 | 6.0 | 6.0 | 41.5 | 2.0 | 9.5 |
| Vermont | 2005 | 13.9 | 8.4 | 0.0 | 55.1 | 4.4 | 18.3 |
| Vermont | 2006 | 24.0 | 9.8 | 2.9 | 49.2 | 4.3 | 8.5 |
| Vermont | 2007 | 25.4 | 8.0 | 0.0 | 51.0 | 1.6 | 14.1 |
| Vermont | 2008 | 24.9 | 5.6 | 1.4 | 51.2 | 2.9 | 14.0 |
| Vermont | 2009 | 18.8 | 0.0 | 1.8 | 49.1 | 12.1 | 18.2 |
| Vermont | 2010 | 15.3 | 0.0 | 0.0 | 55.0 | 6.5 | 23.2 |
| Vermont | 2011 | 25.1 | 6.5 | 3.7 | 41.7 | 2.6 | 20.4 |
| Vermont | 2012 | 25.3 | 0.0 | 4.3 | 51.0 | 1.4 | 18.0 |
| Vermont | 2013 | 41.4 | 2.3 | 1.1 | 36.3 | 6.6 | 12.4 |
| Vermont | 2014 | 60.3 | 1.2 | 5.1 | 18.2 | 1.2 | 14.0 |
| Vermont | 2015 | 58.2 | 5.2 | 0.0 | 21.9 | 3.2 | 11.6 |
| Vermont | 2016 | 63.8 | 5.0 | 2.5 | 19.5 | 3.4 | 5.9 |
| Vermont | 2017 | 65.9 | 3.8 | 1.5 | 19.7 | 1.5 | 7.6 |
| Vermont | 2018 | 76.7 | 4.1 | 0.7 | 8.9 | 0.7 | 8.9 |
| Vermont | 2019 | 78.2 | 6.9 | 1.5 | 7.8 | 0.8 | 4.7 |
| Vermont | 2020 | 73.9 | 8.0 | 2.7 | 8.0 | 2.1 | 5.3 |
| Virginia | 2000 | 24.0 | 11.8 | 1.4 | 30.4 | 3.6 | 28.3 |
| Virginia | 2001 | 28.1 | 9.6 | 0.2 | 38.8 | 4.9 | 18.3 |
| Virginia | 2002 | 29.2 | 11.7 | 0.9 | 39.1 | 3.4 | 15.8 |
| Virginia | 2003 | 26.3 | 10.6 | 1.2 | 39.8 | 4.0 | 18.2 |
| Virginia | 2004 | 25.0 | 11.7 | 0.4 | 45.0 | 2.8 | 15.1 |
| Virginia | 2005 | 19.5 | 15.3 | 1.4 | 43.5 | 3.7 | 16.7 |
| Virginia | 2006 | 22.9 | 15.9 | 1.0 | 40.2 | 4.2 | 15.7 |
| Virginia | 2007 | 24.6 | 9.9 | 1.1 | 44.0 | 5.5 | 14.9 |
| Virginia | 2008 | 27.6 | 6.6 | 1.6 | 46.6 | 3.8 | 13.8 |
| Virginia | 2009 | 30.1 | 4.9 | 1.2 | 45.5 | 4.3 | 14.0 |
| Virginia | 2010 | 25.9 | 7.0 | 1.1 | 47.7 | 5.3 | 13.1 |
| Virginia | 2011 | 23.0 | 6.3 | 1.2 | 49.6 | 5.0 | 15.0 |
| Virginia | 2012 | 29.6 | 3.8 | 1.7 | 43.2 | 4.6 | 17.0 |
| Virginia | 2013 | 39.2 | 5.1 | 1.9 | 36.2 | 5.2 | 12.4 |
| Virginia | 2014 | 43.2 | 4.9 | 3.3 | 32.9 | 4.3 | 11.3 |
| Virginia | 2015 | 52.6 | 6.2 | 3.4 | 24.5 | 3.0 | 10.3 |
| Virginia | 2016 | 62.2 | 4.9 | 3.5 | 18.1 | 3.6 | 7.8 |
| Virginia | 2017 | 68.5 | 5.3 | 3.2 | 13.8 | 2.1 | 7.2 |
| Virginia | 2018 | 70.2 | 4.8 | 3.3 | 12.2 | 1.7 | 7.8 |
| Virginia | 2019 | 72.5 | 4.3 | 6.1 | 10.0 | 1.4 | 5.7 |
| Virginia | 2020 | 78.4 | 2.9 | 6.1 | 7.0 | 1.1 | 4.6 |
| Washington | 2000 | 15.2 | 19.3 | 9.7 | 26.8 | 4.4 | 24.6 |
| Washington | 2001 | 15.8 | 14.2 | 8.6 | 35.3 | 3.8 | 22.3 |
| Washington | 2002 | 18.8 | 12.0 | 9.6 | 35.3 | 3.8 | 20.5 |
| Washington | 2003 | 14.5 | 10.9 | 12.9 | 37.8 | 4.7 | 19.2 |
| Washington | 2004 | 12.8 | 11.2 | 10.4 | 47.3 | 3.6 | 14.7 |
| Washington | 2005 | 13.6 | 8.3 | 11.4 | 50.3 | 3.3 | 13.1 |
| Washington | 2006 | 12.4 | 9.2 | 8.8 | 53.4 | 3.1 | 13.0 |
| Washington | 2007 | 13.1 | 8.5 | 9.7 | 50.9 | 4.4 | 13.4 |
| Washington | 2008 | 13.6 | 5.9 | 6.9 | 54.8 | 4.4 | 14.3 |
| Washington | 2009 | 15.0 | 6.4 | 10.9 | 49.5 | 3.6 | 14.5 |
| Washington | 2010 | 15.2 | 3.9 | 9.1 | 51.4 | 4.3 | 16.1 |
| Washington | 2011 | 21.6 | 4.1 | 11.1 | 45.8 | 3.6 | 13.8 |
| Washington | 2012 | 25.2 | 2.4 | 12.3 | 42.6 | 4.3 | 13.2 |
| Washington | 2013 | 29.2 | 4.5 | 15.3 | 34.9 | 4.1 | 12.0 |
| Washington | 2014 | 37.9 | 2.4 | 14.5 | 29.5 | 3.2 | 12.6 |
| Washington | 2015 | 35.8 | 2.7 | 21.4 | 26.2 | 2.9 | 11.0 |
| Washington | 2016 | 36.3 | 3.5 | 20.6 | 27.2 | 2.9 | 9.5 |
| Washington | 2017 | 39.8 | 3.1 | 22.9 | 21.9 | 2.1 | 10.2 |
| Washington | 2018 | 47.6 | 3.6 | 24.1 | 14.6 | 2.1 | 7.9 |
| Washington | 2019 | 54.0 | 2.2 | 24.0 | 11.2 | 1.5 | 7.1 |
| Washington | 2020 | 60.4 | 2.2 | 22.4 | 8.4 | 1.4 | 5.2 |
| West Virginia | 2000 | 13.5 | 7.1 | 0.0 | 32.0 | 16.5 | 30.0 |
| West Virginia | 2001 | 23.5 | 4.3 | 0.0 | 49.4 | 6.1 | 16.2 |
| West Virginia | 2002 | 21.3 | 4.7 | 0.0 | 59.6 | 6.2 | 8.1 |
| West Virginia | 2003 | 19.3 | 9.4 | 0.4 | 58.3 | 4.5 | 8.1 |
| West Virginia | 2004 | 24.1 | 5.3 | 0.3 | 53.2 | 5.6 | 11.6 |
| West Virginia | 2005 | 25.8 | 5.2 | 0.6 | 53.3 | 4.1 | 11.0 |
| West Virginia | 2006 | 16.7 | 7.1 | 0.6 | 63.5 | 3.1 | 9.0 |
| West Virginia | 2007 | 25.8 | 4.9 | 0.3 | 58.5 | 5.2 | 5.4 |
| West Virginia | 2008 | 27.8 | 3.5 | 0.5 | 55.2 | 5.3 | 7.7 |
| West Virginia | 2009 | 28.3 | 3.6 | 0.9 | 52.5 | 7.0 | 7.8 |
| West Virginia | 2010 | 25.4 | 2.8 | 3.0 | 61.1 | 2.8 | 4.9 |
| West Virginia | 2011 | 21.4 | 2.6 | 2.7 | 64.5 | 4.5 | 4.3 |
| West Virginia | 2012 | 26.8 | 2.6 | 2.8 | 56.7 | 4.4 | 6.6 |
| West Virginia | 2013 | 41.4 | 3.4 | 3.6 | 43.8 | 2.9 | 5.0 |
| West Virginia | 2014 | 42.8 | 1.6 | 3.3 | 42.7 | 5.4 | 4.2 |
| West Virginia | 2015 | 49.3 | 2.5 | 5.4 | 35.4 | 3.0 | 4.4 |
| West Virginia | 2016 | 63.9 | 3.3 | 7.6 | 19.7 | 2.8 | 2.6 |
| West Virginia | 2017 | 69.6 | 2.7 | 8.4 | 15.5 | 1.8 | 2.0 |
| West Virginia | 2018 | 68.0 | 2.4 | 13.6 | 12.3 | 1.4 | 2.3 |
| West Virginia | 2019 | 68.5 | 2.9 | 18.6 | 6.7 | 0.8 | 2.5 |
| West Virginia | 2020 | 80.3 | 0.8 | 12.0 | 4.0 | 1.0 | 1.9 |
| Wisconsin | 2000 | 26.9 | 14.7 | 2.6 | 28.2 | 4.7 | 22.9 |
| Wisconsin | 2001 | 21.5 | 13.0 | 0.9 | 39.0 | 4.7 | 20.8 |
| Wisconsin | 2002 | 18.2 | 13.0 | 0.7 | 42.2 | 4.3 | 21.7 |
| Wisconsin | 2003 | 21.6 | 18.1 | 0.6 | 40.3 | 5.2 | 14.2 |
| Wisconsin | 2004 | 18.0 | 17.5 | 1.6 | 39.7 | 6.7 | 16.5 |
| Wisconsin | 2005 | 20.9 | 17.5 | 1.5 | 44.0 | 2.4 | 13.7 |
| Wisconsin | 2006 | 16.8 | 14.9 | 1.4 | 48.6 | 3.8 | 14.5 |
| Wisconsin | 2007 | 16.4 | 14.6 | 2.3 | 48.5 | 3.5 | 14.7 |
| Wisconsin | 2008 | 23.5 | 9.5 | 0.4 | 48.1 | 3.7 | 14.8 |
| Wisconsin | 2009 | 27.4 | 7.8 | 1.6 | 43.1 | 6.0 | 13.9 |
| Wisconsin | 2010 | 28.6 | 6.3 | 1.5 | 44.3 | 5.5 | 13.8 |
| Wisconsin | 2011 | 31.5 | 7.3 | 2.7 | 40.8 | 4.4 | 13.4 |
| Wisconsin | 2012 | 39.5 | 4.0 | 2.2 | 36.4 | 5.1 | 12.7 |
| Wisconsin | 2013 | 40.5 | 6.1 | 2.5 | 35.7 | 5.3 | 9.9 |
| Wisconsin | 2014 | 45.8 | 4.5 | 4.0 | 32.8 | 4.1 | 8.8 |
| Wisconsin | 2015 | 48.3 | 4.3 | 3.2 | 29.4 | 6.2 | 8.6 |
| Wisconsin | 2016 | 59.0 | 3.9 | 3.7 | 23.5 | 3.7 | 6.2 |
| Wisconsin | 2017 | 62.7 | 5.9 | 5.0 | 16.7 | 3.3 | 6.4 |
| Wisconsin | 2018 | 63.1 | 6.1 | 4.6 | 15.3 | 3.1 | 7.7 |
| Wisconsin | 2019 | 67.7 | 5.8 | 5.7 | 11.1 | 3.0 | 6.7 |
| Wisconsin | 2020 | 75.7 | 4.4 | 4.4 | 7.7 | 2.8 | 5.0 |
| Wyoming | 2000 | 0.0 | 0.0 | 13.6 | 36.4 | 9.1 | 40.9 |
| Wyoming | 2001 | 20.0 | 19.0 | 11.4 | 15.9 | 0.0 | 33.7 |
| Wyoming | 2002 | 16.1 | 2.9 | 8.6 | 48.2 | 4.7 | 10.6 |
| Wyoming | 2003 | 10.9 | 0.0 | 18.6 | 16.6 | 5.7 | 37.8 |
| Wyoming | 2004 | 4.9 | 14.3 | 16.3 | 35.8 | 4.9 | 21.4 |
| Wyoming | 2005 | 4.8 | 8.0 | 52.8 | 20.8 | 4.8 | 8.8 |
| Wyoming | 2006 | 3.7 | 0.0 | 19.7 | 31.5 | 10.3 | 29.3 |
| Wyoming | 2007 | 14.0 | 10.8 | 9.3 | 30.4 | 6.6 | 28.7 |
| Wyoming | 2008 | 24.4 | 8.4 | 4.1 | 43.5 | 4.7 | 13.4 |
| Wyoming | 2009 | 9.5 | 4.3 | 8.7 | 50.1 | 1.7 | 24.1 |
| Wyoming | 2010 | 15.0 | 3.6 | 4.8 | 57.0 | 1.8 | 15.5 |
| Wyoming | 2011 | 15.4 | 0.0 | 3.4 | 47.1 | 4.4 | 27.2 |
| Wyoming | 2012 | 17.0 | 0.0 | 8.6 | 37.6 | 8.8 | 24.9 |
| Wyoming | 2013 | 9.7 | 1.0 | 12.3 | 50.0 | 8.0 | 17.9 |
| Wyoming | 2014 | 27.1 | 0.0 | 18.1 | 33.6 | 5.9 | 14.3 |
| Wyoming | 2015 | 16.0 | 0.0 | 19.7 | 38.5 | 8.2 | 17.7 |
| Wyoming | 2016 | 21.2 | 0.0 | 19.4 | 26.9 | 15.1 | 17.4 |
| Wyoming | 2017 | 29.8 | 1.4 | 18.3 | 33.6 | 1.5 | 15.4 |
| Wyoming | 2018 | 23.7 | 3.2 | 28.8 | 34.5 | 4.9 | 5.0 |
| Wyoming | 2019 | 36.3 | 1.3 | 24.5 | 21.1 | 5.2 | 11.6 |
| Wyoming | 2020 | 38.2 | 2.2 | 25.4 | 22.4 | 0.0 | 11.8 |
| United States | 2000 | 20.9 | 18.0 | 3.9 | 24.5 | 5.1 | 27.4 |
| United States | 2001 | 19.9 | 17.5 | 3.4 | 29.3 | 4.9 | 24.8 |
| United States | 2002 | 19.5 | 16.7 | 4.6 | 32.3 | 4.3 | 22.5 |
| United States | 2003 | 18.5 | 17.1 | 5.5 | 34.7 | 4.0 | 20.0 |
| United States | 2004 | 17.7 | 16.6 | 5.7 | 37.6 | 3.8 | 18.4 |
| United States | 2005 | 17.0 | 17.1 | 6.5 | 38.9 | 3.7 | 16.6 |
| United States | 2006 | 18.4 | 17.3 | 5.1 | 40.2 | 3.7 | 15.1 |
| United States | 2007 | 17.5 | 14.5 | 4.7 | 43.8 | 4.1 | 15.2 |
| United States | 2008 | 20.3 | 10.7 | 4.3 | 44.4 | 4.6 | 15.5 |
| United States | 2009 | 22.9 | 8.9 | 5.2 | 43.2 | 4.4 | 15.3 |
| United States | 2010 | 21.2 | 8.1 | 5.8 | 45.1 | 4.5 | 15.2 |
| United States | 2011 | 23.0 | 8.0 | 6.3 | 43.0 | 4.4 | 15.2 |
| United States | 2012 | 27.3 | 6.9 | 7.1 | 39.0 | 4.3 | 15.3 |
| United States | 2013 | 32.6 | 6.6 | 8.8 | 34.0 | 4.2 | 13.7 |
| United States | 2014 | 39.6 | 5.6 | 8.7 | 30.7 | 3.8 | 11.5 |
| United States | 2015 | 45.7 | 5.5 | 9.9 | 25.6 | 3.6 | 9.6 |
| United States | 2016 | 53.6 | 5.8 | 9.8 | 20.1 | 2.9 | 7.7 |
| United States | 2017 | 58.7 | 6.2 | 10.3 | 15.6 | 2.5 | 6.7 |
| United States | 2018 | 60.2 | 5.9 | 12.2 | 12.7 | 2.5 | 6.6 |
| United States | 2019 | 63.2 | 5.6 | 13.3 | 10.1 | 1.9 | 5.9 |
| United States | 2020 | 70.4 | 4.4 | 12.1 | 7.3 | 1.5 | 4.3 |
