# Supplementary material for: Estimated Number of Injection-Involved Overdose Deaths in US States From 2000 to 2020: Secondary Analysis of Surveillance Data
Source: JMIR Public Health Surveill. 2024 Apr 5;10:e49527. doi: 10.2196/49527 (PMC11031697; doi:10.2196/49527)
Supplement: Multimedia Appendix 3 [file publichealth_v10i1e49527_app3.docx]

|  |  |  |  | Estimated Injection-involved Overdose Deaths | |
| --- | --- | --- | --- | --- | --- |
| **State** | **Year** | **Population** | **Overdose Deaths** | **n (95% CI)** | **Rate per 100,000 (95% CI)** |
| Alabama | 2000 | 3,329,900 | 192 | 31 (22-41) | 0.9 (0.7-1.2) |
| Alabama | 2001 | 3,347,225 | 212 | 36 (28-45) | 1.1 (0.8-1.3) |
| Alabama | 2002 | 3,363,499 | 207 | 34 (26-44) | 1.0 (0.8-1.3) |
| Alabama | 2003 | 3,390,408 | 192 | 30 (22-39) | 0.9 (0.7-1.1) |
| Alabama | 2004 | 3,417,067 | 280 | 51 (41-63) | 1.5 (1.2-1.8) |
| Alabama | 2005 | 3,452,576 | 281 | 44 (35-54) | 1.3 (1.0-1.6) |
| Alabama | 2006 | 3,502,183 | 390 | 31 (18-67) | 0.9 (0.5-1.9) |
| Alabama | 2007 | 3,540,544 | 503 | Data not reported to TEDS-A |  |
| Alabama | 2008 | 3,583,279 | 602 | 88 (56-121) | 2.5 (1.6-3.4) |
| Alabama | 2009 | 3,623,746 | 625 | 111 (79-146) | 3.1 (2.2-4.0) |
| Alabama | 2010 | 3,655,075 | 545 | 107 (85-135) | 2.9 (2.3-3.7) |
| Alabama | 2011 | 3,675,689 | 550 | 108 (74-152) | 2.9 (2.0-4.1) |
| Alabama | 2012 | 3,699,663 | 555 | 158 (121-194) | 4.3 (3.3-5.2) |
| Alabama | 2013 | 3,721,314 | 597 | 196 (157-234) | 5.3 (4.2-6.3) |
| Alabama | 2014 | 3,737,520 | 721 | 311 (257-362) | 8.3 (6.9-9.7) |
| Alabama | 2015 | 3,751,644 | 731 | 328 (287-373) | 8.8 (7.6-9.9) |
| Alabama | 2016 | 3,766,363 | 753 | 357 (310-407) | 9.5 (8.2-10.8) |
| Alabama | 2017 | 3,781,412 | 833 | 369 (324-409) | 9.8 (8.6-10.8) |
| Alabama | 2018 | 3,799,029 | 767 | 307 (279-335) | 8.1 (7.3-8.8) |
| Alabama | 2019 | 3,819,238 | 767 | 324 (296-355) | 8.5 (7.7-9.3) |
| Alabama | 2020 | 3,834,249 | 1,023 | 456 (407-513) | 11.9 (10.6-13.4) |
| Alaska | 2000 | 437,348 | 47 | Suppressed |  |
| Alaska | 2001 | 444,943 | 61 | Suppressed |  |
| Alaska | 2002 | 453,855 | 84 | Suppressed |  |
| Alaska | 2003 | 461,571 | 80 | Suppressed |  |
| Alaska | 2004 | 472,951 | 81 | Data not reported to TEDS-A |  |
| Alaska | 2005 | 481,642 | 77 | Suppressed |  |
| Alaska | 2006 | 489,722 | 76 | Suppressed |  |
| Alaska | 2007 | 495,956 | 72 | Suppressed |  |
| Alaska | 2008 | 504,331 | 124 | 41 (23-61) | 8.1 (4.5-12.1) |
| Alaska | 2009 | 512,544 | 129 | 41 (25-58) | 8.0 (4.8-11.2) |
| Alaska | 2010 | 525,960 | 83 | 26 (17-36) | 4.9 (3.1-6.8) |
| Alaska | 2011 | 533,709 | 104 | 38 (23-50) | 7.2 (4.3-9.4) |
| Alaska | 2012 | 542,313 | 128 | 45 (31-62) | 8.3 (5.7-11.4) |
| Alaska | 2013 | 549,149 | 104 | 38 (26-55) | 7.0 (4.7-10.0) |
| Alaska | 2014 | 550,083 | 124 | 45 (30-63) | 8.1 (5.5-11.4) |
| Alaska | 2015 | 552,063 | 121 | 51 (40-65) | 9.2 (7.2-11.8) |
| Alaska | 2016 | 555,432 | 127 | 55 (43-69) | 9.9 (7.7-12.5) |
| Alaska | 2017 | 555,254 | 146 | 69 (53-87) | 12.3 (9.6-15.7) |
| Alaska | 2018 | 553,435 | 108 | 47 (38-59) | 8.6 (6.8-10.6) |
| Alaska | 2019 | 553,161 | 131 | 58 (48-70) | 10.5 (8.8-12.7) |
| Alaska | 2020 | 552,427 | 158 | 83 (68-100) | 15.1 (12.3-18.1) |
| Arizona | 2000 | 3,787,172 | 517 | Suppressed |  |
| Arizona | 2001 | 3,874,462 | 537 | 73 (58-104) | 1.9 (1.5-2.7) |
| Arizona | 2002 | 3,968,317 | 612 | Suppressed |  |
| Arizona | 2003 | 4,056,693 | 665 | Suppressed |  |
| Arizona | 2004 | 4,167,950 | 729 | 108 (77-188) | 2.6 (1.9-4.5) |
| Arizona | 2005 | 4,309,909 | 780 | 104 (78-176) | 2.4 (1.8-4.1) |
| Arizona | 2006 | 4,454,274 | 862 | 145 (112-213) | 3.3 (2.5-4.8) |
| Arizona | 2007 | 4,559,786 | 925 | 135 (102-206) | 3.0 (2.2-4.5) |
| Arizona | 2008 | 4,651,711 | 820 | 134 (104-188) | 2.9 (2.2-4.1) |
| Arizona | 2009 | 4,715,811 | 983 | 154 (129-211) | 3.3 (2.7-4.5) |
| Arizona | 2010 | 4,780,903 | 1,083 | 187 (155-235) | 3.9 (3.2-4.9) |
| Arizona | 2011 | 4,857,125 | 1,063 | 194 (164-240) | 4.0 (3.4-5.0) |
| Arizona | 2012 | 4,939,075 | 1,125 | 198 (167-243) | 4.0 (3.4-4.9) |
| Arizona | 2013 | 5,015,910 | 1,209 | 282 (248-332) | 5.6 (4.9-6.6) |
| Arizona | 2014 | 5,108,215 | 1,204 | 313 (276-366) | 6.1 (5.4-7.2) |
| Arizona | 2015 | 5,203,045 | 1,264 | 344 (303-401) | 6.6 (5.8-7.7) |
| Arizona | 2016 | 5,308,398 | 1,370 | 394 (355-443) | 7.4 (6.7-8.4) |
| Arizona | 2017 | 5,409,363 | 1,519 | 473 (434-521) | 8.7 (8.0-9.6) |
| Arizona | 2018 | 5,525,571 | 1,653 | 600 (565-640) | 10.9 (10.2-11.6) |
| Arizona | 2019 | 5,650,116 | 1,877 | 696 (599-811) | 12.3 (10.6-14.4) |
| Arizona | 2020 | 5,774,978 | 2,483 | 779 (539-1,249) | 13.5 (9.3-21.6) |
| Arkansas | 2000 | 1,998,210 | 135 | 36 (30-42) | 1.8 (1.5-2.1) |
| Arkansas | 2001 | 2,011,965 | 122 | 35 (27-41) | 1.7 (1.4-2.1) |
| Arkansas | 2002 | 2,027,129 | 170 | 59 (45-70) | 2.9 (2.2-3.5) |
| Arkansas | 2003 | 2,045,237 | 182 | 62 (50-72) | 3.0 (2.4-3.5) |
| Arkansas | 2004 | 2,066,520 | 225 | Data not reported to TEDS-A |  |
| Arkansas | 2005 | 2,091,310 | 269 | 64 (46-79) | 3.1 (2.2-3.8) |
| Arkansas | 2006 | 2,123,919 | 277 | 69 (51-86) | 3.2 (2.4-4.0) |
| Arkansas | 2007 | 2,145,913 | 296 | 71 (51-86) | 3.3 (2.4-4.0) |
| Arkansas | 2008 | 2,168,829 | 353 | 102 (82-114) | 4.7 (3.8-5.3) |
| Arkansas | 2009 | 2,188,957 | 347 | 107 (78-124) | 4.9 (3.6-5.7) |
| Arkansas | 2010 | 2,210,348 | 345 | 101 (73-125) | 4.6 (3.3-5.7) |
| Arkansas | 2011 | 2,229,331 | 353 | 94 (70-109) | 4.2 (3.1-4.9) |
| Arkansas | 2012 | 2,241,551 | 369 | 98 (72-118) | 4.4 (3.2-5.3) |
| Arkansas | 2013 | 2,251,221 | 313 | 93 (72-108) | 4.1 (3.2-4.8) |
| Arkansas | 2014 | 2,261,321 | 350 | 98 (77-127) | 4.3 (3.4-5.6) |
| Arkansas | 2015 | 2,272,853 | 390 | 119 (94-145) | 5.2 (4.1-6.4) |
| Arkansas | 2016 | 2,285,361 | 396 | 121 (99-143) | 5.3 (4.3-6.2) |
| Arkansas | 2017 | 2,297,903 | 438 | 148 (119-174) | 6.4 (5.2-7.6) |
| Arkansas | 2018 | 2,308,535 | 438 | 170 (140-193) | 7.4 (6.1-8.4) |
| Arkansas | 2019 | 2,319,668 | 379 | 157 (132-182) | 6.8 (5.7-7.9) |
| Arkansas | 2020 | 2,330,808 | 540 | 248 (192-299) | 10.7 (8.2-12.8) |
| California | 2000 | 24,720,888 | 1,921 | 565 (541-597) | 2.3 (2.2-2.4) |
| California | 2001 | 25,153,992 | 1,216 | 370 (356-389) | 1.5 (1.4-1.5) |
| California | 2002 | 25,506,701 | 2,955 | 731 (697-777) | 2.9 (2.7-3.0) |
| California | 2003 | 25,848,565 | 3,080 | 755 (719-801) | 2.9 (2.8-3.1) |
| California | 2004 | 26,156,079 | 3,105 | 689 (652-737) | 2.6 (2.5-2.8) |
| California | 2005 | 26,422,378 | 3,189 | 625 (588-673) | 2.4 (2.2-2.5) |
| California | 2006 | 26,650,318 | 3,452 | 704 (667-752) | 2.6 (2.5-2.8) |
| California | 2007 | 26,914,691 | 3,812 | 715 (677-767) | 2.7 (2.5-2.9) |
| California | 2008 | 27,282,716 | 3,833 | 782 (744-831) | 2.9 (2.7-3.0) |
| California | 2009 | 27,666,728 | 4,019 | 851 (808-903) | 3.1 (2.9-3.3) |
| California | 2010 | 28,038,943 | 4,011 | 824 (780-879) | 2.9 (2.8-3.1) |
| California | 2011 | 28,391,169 | 4,143 | 838 (793-893) | 3.0 (2.8-3.1) |
| California | 2012 | 28,750,033 | 4,012 | 825 (785-874) | 2.9 (2.7-3.0) |
| California | 2013 | 29,094,983 | 4,430 | 994 (947-1,051) | 3.4 (3.3-3.6) |
| California | 2014 | 29,450,726 | 4,498 | 1,051 (1,007-1,106) | 3.6 (3.4-3.8) |
| California | 2015 | 29,785,477 | 4,631 | 1,104 (1,055-1,164) | 3.7 (3.5-3.9) |
| California | 2016 | 30,060,643 | 4,615 | 1,175 (1,126-1,234) | 3.9 (3.7-4.1) |
| California | 2017 | 30,287,695 | 4,837 | 1,318 (1,265-1,380) | 4.4 (4.2-4.6) |
| California | 2018 | 30,462,986 | 5,317 | 1,588 (1,532-1,653) | 5.2 (5.0-5.4) |
| California | 2019 | 30,556,506 | 6,148 | 2,073 (1,996-2,161) | 6.8 (6.5-7.1) |
| California | 2020 | 30,576,844 | 8,778 | 3,475 (3,343-3,620) | 11.4 (10.9-11.8) |
| Colorado | 2000 | 3,220,245 | 346 | 97 (85-112) | 3.0 (2.6-3.5) |
| Colorado | 2001 | 3,299,040 | 405 | 81 (66-105) | 2.4 (2.0-3.2) |
| Colorado | 2002 | 3,352,133 | 442 | 99 (86-119) | 3.0 (2.6-3.5) |
| Colorado | 2003 | 3,384,135 | 494 | 100 (87-117) | 2.9 (2.6-3.5) |
| Colorado | 2004 | 3,428,644 | 514 | 88 (76-106) | 2.6 (2.2-3.1) |
| Colorado | 2005 | 3,475,489 | 597 | 124 (108-147) | 3.6 (3.1-4.2) |
| Colorado | 2006 | 3,548,591 | 621 | 110 (95-130) | 3.1 (2.7-3.7) |
| Colorado | 2007 | 3,614,434 | 726 | 140 (122-167) | 3.9 (3.4-4.6) |
| Colorado | 2008 | 3,686,441 | 740 | 135 (117-161) | 3.7 (3.2-4.4) |
| Colorado | 2009 | 3,754,982 | 759 | 198 (175-225) | 5.3 (4.7-6.0) |
| Colorado | 2010 | 3,820,259 | 646 | 159 (142-179) | 4.2 (3.7-4.7) |
| Colorado | 2011 | 3,889,068 | 831 | 226 (202-253) | 5.8 (5.2-6.5) |
| Colorado | 2012 | 3,957,199 | 801 | 202 (183-226) | 5.1 (4.6-5.7) |
| Colorado | 2013 | 4,029,749 | 836 | 247 (227-271) | 6.1 (5.6-6.7) |
| Colorado | 2014 | 4,104,487 | 890 | 267 (244-296) | 6.5 (6.0-7.2) |
| Colorado | 2015 | 4,196,016 | 861 | 254 (230-284) | 6.0 (5.5-6.8) |
| Colorado | 2016 | 4,279,740 | 934 | 308 (286-335) | 7.2 (6.7-7.8) |
| Colorado | 2017 | 4,353,202 | 1,006 | 338 (315-364) | 7.8 (7.2-8.4) |
| Colorado | 2018 | 4,432,929 | 990 | 328 (306-356) | 7.4 (6.9-8.0) |
| Colorado | 2019 | 4,501,813 | 1,067 | 372 (345-403) | 8.3 (7.7-8.9) |
| Colorado | 2020 | 4,557,684 | 1,461 | 553 (513-599) | 12.1 (11.3-13.1) |
| Connecticut | 2000 | 2,569,535 | 317 | Data not reported to TEDS-A |  |
| Connecticut | 2001 | 2,586,985 | 306 | Data not reported to TEDS-A |  |
| Connecticut | 2002 | 2,609,872 | 347 | Data not reported to TEDS-A |  |
| Connecticut | 2003 | 2,633,221 | 305 | Data not reported to TEDS-A |  |
| Connecticut | 2004 | 2,647,115 | 329 | Data not reported to TEDS-A |  |
| Connecticut | 2005 | 2,662,922 | 290 | 79 (74-84) | 3.0 (2.8-3.2) |
| Connecticut | 2006 | 2,678,088 | 404 | 96 (90-104) | 3.6 (3.4-3.9) |
| Connecticut | 2007 | 2,693,786 | 430 | 130 (124-138) | 4.8 (4.6-5.1) |
| Connecticut | 2008 | 2,718,953 | 380 | 113 (108-120) | 4.2 (4.0-4.4) |
| Connecticut | 2009 | 2,740,968 | 392 | 116 (110-124) | 4.2 (4.0-4.5) |
| Connecticut | 2010 | 2,764,774 | 363 | 90 (84-97) | 3.2 (3.0-3.5) |
| Connecticut | 2011 | 2,783,911 | 401 | 101 (96-107) | 3.6 (3.4-3.8) |
| Connecticut | 2012 | 2,800,510 | 436 | 121 (115-128) | 4.3 (4.1-4.6) |
| Connecticut | 2013 | 2,813,054 | 581 | 193 (184-204) | 6.9 (6.5-7.3) |
| Connecticut | 2014 | 2,822,669 | 620 | 237 (228-250) | 8.4 (8.1-8.8) |
| Connecticut | 2015 | 2,826,387 | 794 | 325 (313-340) | 11.5 (11.1-12.0) |
| Connecticut | 2016 | 2,827,529 | 965 | 433 (418-450) | 15.3 (14.8-15.9) |
| Connecticut | 2017 | 2,831,595 | 1,068 | 478 (461-497) | 16.9 (16.3-17.5) |
| Connecticut | 2018 | 2,838,500 | 1,065 | 458 (440-481) | 16.1 (15.5-16.9) |
| Connecticut | 2019 | 2,838,742 | 1,212 | 498 (475-523) | 17.5 (16.7-18.4) |
| Connecticut | 2020 | 2,838,054 | 1,364 | 543 (510-578) | 19.1 (18.0-20.4) |
| Delaware | 2000 | 591,459 | 54 | 10 (8-16) | 1.7 (1.3-2.6) |
| Delaware | 2001 | 599,661 | 63 | 10 (7-16) | 1.6 (1.2-2.6) |
| Delaware | 2002 | 609,223 | 84 | 13 (10-22) | 2.2 (1.6-3.7) |
| Delaware | 2003 | 619,958 | 75 | 13 (11-18) | 2.1 (1.7-2.9) |
| Delaware | 2004 | 631,172 | 63 | 11 (8-14) | 1.7 (1.3-2.2) |
| Delaware | 2005 | 643,162 | 62 | 9 (8-15) | 1.5 (1.2-2.4) |
| Delaware | 2006 | 655,539 | 76 | 17 (14-20) | 2.5 (2.1-3.1) |
| Delaware | 2007 | 666,594 | 95 | 18 (15-23) | 2.7 (2.2-3.4) |
| Delaware | 2008 | 677,758 | 124 | 22 (17-30) | 3.2 (2.6-4.4) |
| Delaware | 2009 | 685,517 | 133 | 25 (21-33) | 3.7 (3.0-4.8) |
| Delaware | 2010 | 694,117 | 144 | 30 (25-39) | 4.4 (3.5-5.7) |
| Delaware | 2011 | 702,746 | 154 | 30 (25-37) | 4.3 (3.6-5.3) |
| Delaware | 2012 | 711,290 | 141 | 39 (33-46) | 5.4 (4.6-6.5) |
| Delaware | 2013 | 720,622 | 165 | 44 (37-55) | 6.1 (5.1-7.7) |
| Delaware | 2014 | 729,567 | 187 | 61 (54-72) | 8.4 (7.4-9.9) |
| Delaware | 2015 | 738,143 | 196 | 78 (71-91) | 10.6 (9.6-12.3) |
| Delaware | 2016 | 745,946 | 281 | 124 (111-142) | 16.6 (14.9-19.0) |
| Delaware | 2017 | 753,777 | 337 | 154 (141-168) | 20.4 (18.7-22.3) |
| Delaware | 2018 | 762,831 | 400 | 182 (170-195) | 23.8 (22.3-25.6) |
| Delaware | 2019 | 772,405 | 435 | 179 (166-194) | 23.2 (21.5-25.1) |
| Delaware | 2020 | 782,153 | 444 | 175 (159-192) | 22.3 (20.3-24.6) |
| District of Columbia | 2000 | 457,543 | 76 | Suppressed |  |
| District of Columbia | 2001 | 459,879 | 92 | Suppressed |  |
| District of Columbia | 2002 | 459,336 | 61 | Suppressed |  |
| District of Columbia | 2003 | 457,099 | 101 | Suppressed |  |
| District of Columbia | 2004 | 457,998 | 86 | Data not reported to TEDS-A |  |
| District of Columbia | 2005 | 459,949 | 77 | Data not reported to TEDS-A |  |
| District of Columbia | 2006 | 465,030 | 100 | Data not reported to TEDS-A |  |
| District of Columbia | 2007 | 470,278 | 61 | Suppressed |  |
| District of Columbia | 2008 | 477,979 | 56 | Suppressed |  |
| District of Columbia | 2009 | 490,130 | 26 | Data not reported to TEDS-A |  |
| District of Columbia | 2010 | 504,016 | 78 | Suppressed |  |
| District of Columbia | 2011 | 516,370 | 86 | Suppressed |  |
| District of Columbia | 2012 | 528,072 | 76 | Suppressed |  |
| District of Columbia | 2013 | 539,546 | 101 | Suppressed |  |
| District of Columbia | 2014 | 547,800 | 96 | Suppressed |  |
| District of Columbia | 2015 | 557,837 | 125 | Suppressed |  |
| District of Columbia | 2016 | 565,995 | 269 | Suppressed |  |
| District of Columbia | 2017 | 572,258 | 310 | Suppressed |  |
| District of Columbia | 2018 | 577,444 | 254 | Suppressed |  |
| District of Columbia | 2019 | 580,301 | 310 | Suppressed |  |
| District of Columbia | 2020 | 583,228 | 421 | Suppressed |  |
| Florida | 2000 | 12,392,635 | 1,145 | 329 (305-355) | 2.7 (2.5-2.9) |
| Florida | 2001 | 12,642,527 | 1,714 | 461 (420-508) | 3.6 (3.3-4.0) |
| Florida | 2002 | 12,914,746 | 1,898 | 516 (478-561) | 4.0 (3.7-4.3) |
| Florida | 2003 | 13,183,209 | 2,036 | 547 (502-598) | 4.1 (3.8-4.5) |
| Florida | 2004 | 13,524,584 | 2,233 | Data not reported to TEDS-A |  |
| Florida | 2005 | 13,873,860 | 2,344 | 412 (351-484) | 3.0 (2.5-3.5) |
| Florida | 2006 | 14,144,078 | 2,555 | 406 (355-473) | 2.9 (2.5-3.3) |
| Florida | 2007 | 14,336,744 | 2,756 | 423 (362-499) | 3.0 (2.5-3.5) |
| Florida | 2008 | 14,508,933 | 2,930 | 470 (417-536) | 3.2 (2.9-3.7) |
| Florida | 2009 | 14,655,361 | 3,062 | 570 (511-639) | 3.9 (3.5-4.4) |
| Florida | 2010 | 14,848,458 | 3,033 | 603 (542-674) | 4.1 (3.7-4.5) |
| Florida | 2011 | 15,055,836 | 2,889 | 654 (604-707) | 4.3 (4.0-4.7) |
| Florida | 2012 | 15,293,175 | 2,584 | 722 (660-788) | 4.7 (4.3-5.2) |
| Florida | 2013 | 15,528,132 | 2,458 | 900 (829-972) | 5.8 (5.3-6.3) |
| Florida | 2014 | 15,798,828 | 2,622 | 1,072 (984-1,164) | 6.8 (6.2-7.4) |
| Florida | 2015 | 16,114,774 | 3,207 | 1,452 (1,343-1,569) | 9.0 (8.3-9.7) |
| Florida | 2016 | 16,463,604 | 4,703 | 2,339 (2,173-2,514) | 14.2 (13.2-15.3) |
| Florida | 2017 | 16,772,222 | 5,069 | 2,601 (2,465-2,742) | 15.5 (14.7-16.4) |
| Florida | 2018 | 17,028,792 | 4,685 | 2,322 (2,233-2,414) | 13.6 (13.1-14.2) |
| Florida | 2019 | 17,258,089 | 5,247 | 2,660 (2,488-2,844) | 15.4 (14.4-16.5) |
| Florida | 2020 | 17,482,580 | 7,202 | 3,767 (3,475-4,073) | 21.5 (19.9-23.3) |
| Georgia | 2000 | 6,050,727 | 355 | 25 (16-42) | 0.4 (0.3-0.7) |
| Georgia | 2001 | 6,161,648 | 501 | 56 (42-78) | 0.9 (0.7-1.3) |
| Georgia | 2002 | 6,258,472 | 552 | 78 (60-104) | 1.2 (1.0-1.7) |
| Georgia | 2003 | 6,344,083 | 593 | 71 (54-96) | 1.1 (0.8-1.5) |
| Georgia | 2004 | 6,460,397 | 661 | 75 (56-102) | 1.2 (0.9-1.6) |
| Georgia | 2005 | 6,572,318 | 723 | 82 (65-102) | 1.3 (1.0-1.6) |
| Georgia | 2006 | 6,749,799 | 822 | 106 (87-129) | 1.6 (1.3-1.9) |
| Georgia | 2007 | 6,893,739 | 908 | 102 (82-127) | 1.5 (1.2-1.8) |
| Georgia | 2008 | 7,025,746 | 917 | 119 (95-150) | 1.7 (1.3-2.1) |
| Georgia | 2009 | 7,135,065 | 993 | 140 (113-172) | 2.0 (1.6-2.4) |
| Georgia | 2010 | 7,222,411 | 1,039 | 170 (141-203) | 2.3 (2.0-2.8) |
| Georgia | 2011 | 7,317,045 | 1,049 | 174 (145-207) | 2.4 (2.0-2.8) |
| Georgia | 2012 | 7,418,788 | 1,045 | 160 (136-190) | 2.2 (1.8-2.6) |
| Georgia | 2013 | 7,491,410 | 1,092 | 219 (190-252) | 2.9 (2.5-3.4) |
| Georgia | 2014 | 7,581,730 | 1,202 | 340 (303-380) | 4.5 (4.0-5.0) |
| Georgia | 2015 | 7,684,073 | 1,286 | 448 (404-494) | 5.8 (5.3-6.4) |
| Georgia | 2016 | 7,797,409 | 1,384 | 473 (418-528) | 6.1 (5.4-6.8) |
| Georgia | 2017 | 7,903,220 | 1,528 | 576 (515-637) | 7.3 (6.5-8.1) |
| Georgia | 2018 | 8,009,933 | 1,395 | 506 (453-562) | 6.3 (5.7-7.0) |
| Georgia | 2019 | 8,122,621 | 1,394 | 490 (436-552) | 6.0 (5.4-6.8) |
| Georgia | 2020 | 8,210,067 | 1,902 | 773 (683-868) | 9.4 (8.3-10.6) |
| Hawaii | 2000 | 918,167 | 63 | 8 (5-17) | 0.9 (0.5-1.9) |
| Hawaii | 2001 | 931,815 | 76 | 10 (5-20) | 1.0 (0.5-2.1) |
| Hawaii | 2002 | 946,013 | 77 | 12 (7-23) | 1.3 (0.8-2.4) |
| Hawaii | 2003 | 956,635 | 92 | 13 (8-24) | 1.4 (0.8-2.5) |
| Hawaii | 2004 | 975,466 | 102 | 18 (11-31) | 1.8 (1.1-3.1) |
| Hawaii | 2005 | 994,232 | 126 | 19 (11-34) | 1.9 (1.1-3.5) |
| Hawaii | 2006 | 1,010,418 | 85 | 11 (5-23) | 1.1 (0.5-2.3) |
| Hawaii | 2007 | 1,015,468 | 136 | 25 (14-45) | 2.4 (1.4-4.4) |
| Hawaii | 2008 | 1,031,119 | 122 | 12 (6-27) | 1.1 (0.6-2.6) |
| Hawaii | 2009 | 1,043,921 | 134 | 13 (6-27) | 1.3 (0.5-2.5) |
| Hawaii | 2010 | 1,060,126 | 150 | 22 (11-38) | 2.1 (1.0-3.6) |
| Hawaii | 2011 | 1,073,598 | 179 | 28 (15-49) | 2.6 (1.4-4.6) |
| Hawaii | 2012 | 1,087,699 | 153 | 23 (13-39) | 2.1 (1.2-3.6) |
| Hawaii | 2013 | 1,099,924 | 158 | 34 (19-53) | 3.1 (1.7-4.8) |
| Hawaii | 2014 | 1,107,217 | 157 | 34 (22-53) | 3.1 (1.9-4.8) |
| Hawaii | 2015 | 1,114,576 | 168 | 33 (21-47) | 3.0 (1.9-4.2) |
| Hawaii | 2016 | 1,121,290 | 190 | 42 (28-63) | 3.8 (2.5-5.7) |
| Hawaii | 2017 | 1,120,403 | 200 | 33 (21-57) | 2.9 (1.9-5.1) |
| Hawaii | 2018 | 1,120,053 | 211 | 43 (27-69) | 3.8 (2.4-6.1) |
| Hawaii | 2019 | 1,116,196 | 241 | Suppressed |  |
| Hawaii | 2020 | 1,111,188 | 273 | Suppressed |  |
| Idaho | 2000 | 929,000 | 63 | 7 (5-15) | 0.8 (0.6-1.6) |
| Idaho | 2001 | 946,817 | 97 | 21 (16-29) | 2.2 (1.7-3.1) |
| Idaho | 2002 | 964,386 | 116 | Suppressed |  |
| Idaho | 2003 | 984,139 | 100 | Suppressed |  |
| Idaho | 2004 | 1,007,110 | 95 | Suppressed |  |
| Idaho | 2005 | 1,033,590 | 107 | 22 (14-33) | 2.1 (1.3-3.2) |
| Idaho | 2006 | 1,063,916 | 127 | 32 (23-47) | 3.0 (2.1-4.4) |
| Idaho | 2007 | 1,090,081 | 126 | Suppressed |  |
| Idaho | 2008 | 1,111,973 | 145 | 33 (20-51) | 2.9 (1.8-4.6) |
| Idaho | 2009 | 1,128,363 | 170 | 46 (34-63) | 4.1 (3.0-5.6) |
| Idaho | 2010 | 1,141,569 | 178 | 67 (50-88) | 5.9 (4.4-7.7) |
| Idaho | 2011 | 1,155,369 | 192 | 58 (42-78) | 5.0 (3.7-6.7) |
| Idaho | 2012 | 1,167,926 | 181 | 52 (40-65) | 4.5 (3.5-5.6) |
| Idaho | 2013 | 1,183,066 | 206 | 56 (40-79) | 4.7 (3.4-6.7) |
| Idaho | 2014 | 1,200,934 | 210 | 69 (48-97) | 5.7 (4.0-8.1) |
| Idaho | 2015 | 1,218,559 | 214 | 78 (54-107) | 6.4 (4.5-8.7) |
| Idaho | 2016 | 1,245,890 | 241 | Suppressed |  |
| Idaho | 2017 | 1,276,702 | 234 | Suppressed |  |
| Idaho | 2018 | 1,306,940 | 247 | Suppressed |  |
| Idaho | 2019 | 1,340,944 | 262 | Suppressed |  |
| Idaho | 2020 | 1,375,870 | 284 | Data not reported to TEDS-A |  |
| Illinois | 2000 | 9,189,217 | 856 | 64 (51-83) | 0.7 (0.6-0.9) |
| Illinois | 2001 | 9,244,828 | 883 | 72 (57-95) | 0.8 (0.6-1.0) |
| Illinois | 2002 | 9,287,194 | 976 | 83 (66-106) | 0.9 (0.7-1.1) |
| Illinois | 2003 | 9,330,459 | 835 | 61 (48-79) | 0.7 (0.5-0.8) |
| Illinois | 2004 | 9,378,174 | 1,023 | 75 (61-94) | 0.8 (0.7-1.0) |
| Illinois | 2005 | 9,412,585 | 1,057 | 96 (78-121) | 1.0 (0.8-1.3) |
| Illinois | 2006 | 9,462,709 | 1,360 | 210 (189-237) | 2.2 (2.0-2.5) |
| Illinois | 2007 | 9,525,732 | 1,181 | 133 (116-159) | 1.4 (1.2-1.7) |
| Illinois | 2008 | 9,593,637 | 1,346 | 165 (148-191) | 1.7 (1.5-2.0) |
| Illinois | 2009 | 9,658,372 | 1,370 | 194 (174-220) | 2.0 (1.8-2.3) |
| Illinois | 2010 | 9,717,263 | 1,281 | 239 (221-261) | 2.5 (2.3-2.7) |
| Illinois | 2011 | 9,775,099 | 1,395 | 235 (217-259) | 2.4 (2.2-2.6) |
| Illinois | 2012 | 9,823,144 | 1,612 | 290 (264-329) | 3.0 (2.7-3.3) |
| Illinois | 2013 | 9,870,318 | 1,564 | 457 (416-512) | 4.6 (4.2-5.2) |
| Illinois | 2014 | 9,891,872 | 1,693 | 554 (506-620) | 5.6 (5.1-6.3) |
| Illinois | 2015 | 9,896,700 | 1,821 | 671 (633-719) | 6.8 (6.4-7.3) |
| Illinois | 2016 | 9,890,300 | 2,402 | 882 (813-969) | 8.9 (8.2-9.8) |
| Illinois | 2017 | 9,882,838 | 2,765 | 1,034 (973-1,108) | 10.5 (9.8-11.2) |
| Illinois | 2018 | 9,867,336 | 2,708 | 914 (859-977) | 9.3 (8.7-9.9) |
| Illinois | 2019 | 9,849,705 | 2,778 | 894 (836-961) | 9.1 (8.5-9.8) |
| Illinois | 2020 | 9,809,562 | 3,532 | 1,063 (971-1,169) | 10.8 (9.9-11.9) |
| Indiana | 2000 | 4,516,877 | 214 | 43 (38-48) | 1.0 (0.8-1.1) |
| Indiana | 2001 | 4,548,233 | 276 | 51 (43-60) | 1.1 (1.0-1.3) |
| Indiana | 2002 | 4,575,153 | 289 | 39 (31-47) | 0.8 (0.7-1.0) |
| Indiana | 2003 | 4,614,078 | 426 | 84 (72-97) | 1.8 (1.6-2.1) |
| Indiana | 2004 | 4,646,726 | 529 | 89 (76-103) | 1.9 (1.6-2.2) |
| Indiana | 2005 | 4,684,718 | 601 | 120 (102-139) | 2.6 (2.2-3.0) |
| Indiana | 2006 | 4,729,562 | 719 | 152 (134-172) | 3.2 (2.8-3.6) |
| Indiana | 2007 | 4,770,105 | 763 | 150 (129-173) | 3.2 (2.7-3.6) |
| Indiana | 2008 | 4,813,312 | 817 | 203 (178-229) | 4.2 (3.7-4.8) |
| Indiana | 2009 | 4,849,621 | 891 | 255 (222-289) | 5.3 (4.6-6.0) |
| Indiana | 2010 | 4,884,363 | 921 | 210 (180-244) | 4.3 (3.7-5.0) |
| Indiana | 2011 | 4,918,599 | 968 | 200 (175-230) | 4.1 (3.5-4.7) |
| Indiana | 2012 | 4,948,883 | 997 | 254 (225-287) | 5.1 (4.6-5.8) |
| Indiana | 2013 | 4,984,077 | 1,056 | 347 (317-379) | 7.0 (6.4-7.6) |
| Indiana | 2014 | 5,013,367 | 1,165 | 404 (372-438) | 8.1 (7.4-8.7) |
| Indiana | 2015 | 5,032,455 | 1,241 | 496 (458-538) | 9.9 (9.1-10.7) |
| Indiana | 2016 | 5,061,086 | 1,518 | 645 (594-701) | 12.7 (11.7-13.8) |
| Indiana | 2017 | 5,088,163 | 1,843 | 810 (748-878) | 15.9 (14.7-17.3) |
| Indiana | 2018 | 5,126,077 | 1,619 | 750 (706-800) | 14.6 (13.8-15.6) |
| Indiana | 2019 | 5,161,635 | 1,689 | 791 (740-847) | 15.3 (14.3-16.4) |
| Indiana | 2020 | 5,188,514 | 2,305 | 1,250 (1,161-1,344) | 24.1 (22.4-25.9) |
| Iowa | 2000 | 2,195,730 | 71 | 11 (8-15) | 0.5 (0.4-0.7) |
| Iowa | 2001 | 2,203,396 | 87 | 10 (7-16) | 0.5 (0.3-0.7) |
| Iowa | 2002 | 2,210,549 | 90 | 14 (10-20) | 0.6 (0.5-0.9) |
| Iowa | 2003 | 2,221,897 | 93 | 14 (10-20) | 0.6 (0.5-0.9) |
| Iowa | 2004 | 2,234,927 | 120 | 22 (17-28) | 1.0 (0.8-1.3) |
| Iowa | 2005 | 2,245,966 | 140 | 21 (16-28) | 0.9 (0.7-1.2) |
| Iowa | 2006 | 2,260,941 | 183 | 31 (23-42) | 1.4 (1.0-1.9) |
| Iowa | 2007 | 2,275,580 | 201 | 32 (24-43) | 1.4 (1.0-1.9) |
| Iowa | 2008 | 2,291,076 | 209 | 39 (27-53) | 1.7 (1.2-2.3) |
| Iowa | 2009 | 2,305,901 | 205 | 52 (40-64) | 2.3 (1.7-2.8) |
| Iowa | 2010 | 2,322,568 | 250 | 58 (47-70) | 2.5 (2.0-3.0) |
| Iowa | 2011 | 2,340,403 | 248 | 56 (44-71) | 2.4 (1.9-3.0) |
| Iowa | 2012 | 2,351,952 | 256 | 69 (57-82) | 2.9 (2.4-3.5) |
| Iowa | 2013 | 2,367,379 | 270 | 75 (63-89) | 3.2 (2.7-3.7) |
| Iowa | 2014 | 2,381,687 | 261 | 76 (66-90) | 3.2 (2.8-3.8) |
| Iowa | 2015 | 2,392,391 | 307 | 98 (85-114) | 4.1 (3.6-4.8) |
| Iowa | 2016 | 2,401,985 | 313 | 110 (96-124) | 4.6 (4.0-5.2) |
| Iowa | 2017 | 2,411,759 | 341 | 119 (102-138) | 4.9 (4.2-5.7) |
| Iowa | 2018 | 2,420,098 | 282 | 96 (85-109) | 4.0 (3.5-4.5) |
| Iowa | 2019 | 2,431,591 | 348 | 123 (108-139) | 5.1 (4.4-5.7) |
| Iowa | 2020 | 2,438,002 | 428 | 171 (150-194) | 7.0 (6.1-8.0) |
| Kansas | 2000 | 1,979,794 | 103 | 23 (17-28) | 1.1 (0.9-1.4) |
| Kansas | 2001 | 1,991,239 | 136 | 34 (25-44) | 1.7 (1.3-2.2) |
| Kansas | 2002 | 2,004,119 | 170 | 48 (33-62) | 2.4 (1.7-3.1) |
| Kansas | 2003 | 2,015,157 | 184 | 35 (25-49) | 1.8 (1.2-2.4) |
| Kansas | 2004 | 2,028,917 | 221 | 60 (46-80) | 3.0 (2.3-3.9) |
| Kansas | 2005 | 2,040,610 | 237 | 47 (33-65) | 2.3 (1.6-3.2) |
| Kansas | 2006 | 2,057,654 | 252 | 57 (41-77) | 2.8 (2.0-3.7) |
| Kansas | 2007 | 2,072,780 | 265 | 61 (43-81) | 2.9 (2.1-3.9) |
| Kansas | 2008 | 2,093,387 | 218 | 53 (40-66) | 2.5 (1.9-3.1) |
| Kansas | 2009 | 2,110,863 | 300 | 104 (84-117) | 4.9 (4.0-5.5) |
| Kansas | 2010 | 2,130,739 | 262 | 67 (52-87) | 3.2 (2.4-4.1) |
| Kansas | 2011 | 2,142,970 | 279 | 79 (61-97) | 3.7 (2.8-4.5) |
| Kansas | 2012 | 2,159,429 | 316 | 110 (86-131) | 5.1 (4.0-6.1) |
| Kansas | 2013 | 2,169,775 | 325 | 108 (80-132) | 5.0 (3.7-6.1) |
| Kansas | 2014 | 2,179,145 | 330 | 111 (77-142) | 5.1 (3.6-6.5) |
| Kansas | 2015 | 2,189,582 | 327 | 110 (81-136) | 5.0 (3.7-6.2) |
| Kansas | 2016 | 2,195,387 | 309 | 108 (90-126) | 4.9 (4.1-5.7) |
| Kansas | 2017 | 2,198,480 | 329 | 112 (91-133) | 5.1 (4.1-6.1) |
| Kansas | 2018 | 2,206,155 | 340 | 129 (103-152) | 5.8 (4.7-6.9) |
| Kansas | 2019 | 2,211,182 | 398 | 171 (127-201) | 7.7 (5.8-9.1) |
| Kansas | 2020 | 2,217,059 | 474 | 222 (178-265) | 10.0 (8.0-11.9) |
| Kentucky | 2000 | 3,054,037 | 236 | Suppressed |  |
| Kentucky | 2001 | 3,074,027 | 343 | 50 (38-64) | 1.6 (1.2-2.1) |
| Kentucky | 2002 | 3,094,624 | 419 | 68 (52-85) | 2.2 (1.7-2.8) |
| Kentucky | 2003 | 3,118,685 | 555 | 75 (60-92) | 2.4 (1.9-3.0) |
| Kentucky | 2004 | 3,147,642 | 518 | 76 (59-98) | 2.4 (1.9-3.1) |
| Kentucky | 2005 | 3,178,722 | 630 | 85 (70-102) | 2.7 (2.2-3.2) |
| Kentucky | 2006 | 3,207,944 | 723 | 106 (88-127) | 3.3 (2.7-4.0) |
| Kentucky | 2007 | 3,240,384 | 701 | 103 (87-121) | 3.2 (2.7-3.7) |
| Kentucky | 2008 | 3,267,877 | 756 | 124 (106-145) | 3.8 (3.2-4.4) |
| Kentucky | 2009 | 3,295,364 | 767 | 161 (139-187) | 4.9 (4.2-5.7) |
| Kentucky | 2010 | 3,324,912 | 998 | 186 (164-214) | 5.6 (4.9-6.4) |
| Kentucky | 2011 | 3,348,853 | 1,050 | 235 (206-268) | 7.0 (6.2-8.0) |
| Kentucky | 2012 | 3,369,097 | 1,073 | 303 (267-339) | 9.0 (7.9-10.1) |
| Kentucky | 2013 | 3,389,443 | 1,019 | 356 (326-391) | 10.5 (9.6-11.5) |
| Kentucky | 2014 | 3,401,903 | 1,073 | 437 (403-475) | 12.8 (11.9-14.0) |
| Kentucky | 2015 | 3,415,881 | 1,265 | 605 (566-646) | 17.7 (16.6-18.9) |
| Kentucky | 2016 | 3,427,691 | 1,413 | 674 (629-721) | 19.7 (18.4-21.0) |
| Kentucky | 2017 | 3,444,627 | 1,562 | 804 (756-854) | 23.3 (21.9-24.8) |
| Kentucky | 2018 | 3,456,256 | 1,313 | 661 (617-708) | 19.1 (17.8-20.5) |
| Kentucky | 2019 | 3,468,077 | 1,377 | 667 (619-716) | 19.2 (17.9-20.7) |
| Kentucky | 2020 | 3,475,334 | 2,075 | 1,104 (1,025-1,185) | 31.8 (29.5-34.1) |
| Louisiana | 2000 | 3,254,215 | 246 | 29 (21-37) | 0.9 (0.7-1.2) |
| Louisiana | 2001 | 3,273,688 | 254 | 38 (30-46) | 1.1 (0.9-1.4) |
| Louisiana | 2002 | 3,302,448 | 359 | 50 (41-63) | 1.5 (1.3-1.9) |
| Louisiana | 2003 | 3,332,972 | 526 | 84 (70-101) | 2.5 (2.1-3.0) |
| Louisiana | 2004 | 3,369,507 | 529 | 81 (66-96) | 2.4 (2.0-2.8) |
| Louisiana | 2005 | 3,398,674 | 650 | 91 (73-109) | 2.7 (2.2-3.2) |
| Louisiana | 2006 | 3,223,886 | 693 | 99 (74-120) | 3.1 (2.3-3.7) |
| Louisiana | 2007 | 3,278,939 | 798 | 110 (89-131) | 3.4 (2.7-4.0) |
| Louisiana | 2008 | 3,326,858 | 640 | 100 (83-118) | 3.0 (2.5-3.5) |
| Louisiana | 2009 | 3,377,420 | 581 | 113 (100-126) | 3.3 (3.0-3.7) |
| Louisiana | 2010 | 3,426,794 | 587 | 131 (119-144) | 3.8 (3.5-4.2) |
| Louisiana | 2011 | 3,459,913 | 596 | 154 (139-169) | 4.5 (4.0-4.9) |
| Louisiana | 2012 | 3,487,895 | 560 | 191 (174-208) | 5.5 (5.0-6.0) |
| Louisiana | 2013 | 3,513,228 | 807 | 322 (293-351) | 9.2 (8.4-10.0) |
| Louisiana | 2014 | 3,531,723 | 775 | 295 (263-328) | 8.4 (7.5-9.3) |
| Louisiana | 2015 | 3,551,471 | 857 | 355 (309-404) | 10.0 (8.7-11.4) |
| Louisiana | 2016 | 3,566,136 | 983 | 436 (378-495) | 12.2 (10.6-13.9) |
| Louisiana | 2017 | 3,565,731 | 1,106 | 502 (441-559) | 14.1 (12.4-15.7) |
| Louisiana | 2018 | 3,566,132 | 1,135 | 548 (482-613) | 15.4 (13.5-17.2) |
| Louisiana | 2019 | 3,568,379 | 1,260 | 604 (532-679) | 16.9 (14.9-19.0) |
| Louisiana | 2020 | 3,564,038 | 1,889 | 847 (718-986) | 23.8 (20.2-27.7) |
| Maine | 2000 | 975,665 | 59 | 13 (11-18) | 1.4 (1.1-1.8) |
| Maine | 2001 | 985,604 | 84 | 15 (13-19) | 1.6 (1.3-1.9) |
| Maine | 2002 | 997,365 | 139 | 31 (26-37) | 3.1 (2.6-3.7) |
| Maine | 2003 | 1,009,727 | 131 | 29 (25-34) | 2.9 (2.4-3.3) |
| Maine | 2004 | 1,018,897 | 135 | 29 (24-35) | 2.8 (2.4-3.4) |
| Maine | 2005 | 1,026,748 | 162 | 30 (25-36) | 2.9 (2.4-3.5) |
| Maine | 2006 | 1,034,674 | 157 | 26 (22-32) | 2.6 (2.1-3.1) |
| Maine | 2007 | 1,040,855 | 159 | 30 (26-35) | 2.9 (2.5-3.3) |
| Maine | 2008 | 1,048,305 | 160 | 31 (26-37) | 3.0 (2.5-3.5) |
| Maine | 2009 | 1,051,644 | 173 | 30 (26-35) | 2.9 (2.5-3.4) |
| Maine | 2010 | 1,054,243 | 135 | 29 (26-34) | 2.8 (2.5-3.2) |
| Maine | 2011 | 1,059,325 | 156 | 33 (28-39) | 3.1 (2.6-3.7) |
| Maine | 2012 | 1,062,933 | 149 | 39 (35-44) | 3.7 (3.3-4.2) |
| Maine | 2013 | 1,066,439 | 171 | 45 (40-53) | 4.3 (3.7-4.9) |
| Maine | 2014 | 1,071,433 | 215 | 81 (74-88) | 7.5 (6.9-8.3) |
| Maine | 2015 | 1,072,605 | 269 | 122 (112-132) | 11.4 (10.5-12.3) |
| Maine | 2016 | 1,077,640 | 353 | 161 (146-177) | 14.9 (13.6-16.4) |
| Maine | 2017 | 1,082,884 | 423 | 203 (186-221) | 18.8 (17.2-20.4) |
| Maine | 2018 | 1,089,658 | 344 | 154 (138-173) | 14.1 (12.6-15.9) |
| Maine | 2019 | 1,096,160 | 371 | 178 (162-197) | 16.2 (14.8-18.0) |
| Maine | 2020 | 1,101,973 | 495 | 229 (202-257) | 20.8 (18.3-23.3) |
| Maryland | 2000 | 3,954,073 | 614 | 42 (37-47) | 1.0 (0.9-1.2) |
| Maryland | 2001 | 4,008,139 | 626 | 36 (32-40) | 0.9 (0.8-1.0) |
| Maryland | 2002 | 4,065,035 | 716 | 55 (51-59) | 1.3 (1.2-1.5) |
| Maryland | 2003 | 4,116,628 | 771 | 68 (63-74) | 1.6 (1.5-1.8) |
| Maryland | 2004 | 4,163,485 | 669 | 85 (79-95) | 2.0 (1.9-2.3) |
| Maryland | 2005 | 4,209,413 | 650 | 97 (90-106) | 2.3 (2.1-2.5) |
| Maryland | 2006 | 4,249,611 | 752 | 131 (121-143) | 3.1 (2.9-3.4) |
| Maryland | 2007 | 4,283,845 | 758 | 137 (128-148) | 3.2 (3.0-3.5) |
| Maryland | 2008 | 4,325,751 | 684 | 123 (114-134) | 2.8 (2.6-3.1) |
| Maryland | 2009 | 4,376,757 | 739 | 168 (158-181) | 3.8 (3.6-4.1) |
| Maryland | 2010 | 4,436,123 | 641 | 139 (128-152) | 3.1 (2.9-3.4) |
| Maryland | 2011 | 4,490,429 | 700 | 160 (150-171) | 3.6 (3.3-3.8) |
| Maryland | 2012 | 4,540,807 | 828 | 204 (191-218) | 4.5 (4.2-4.8) |
| Maryland | 2013 | 4,580,592 | 885 | 243 (229-260) | 5.3 (5.0-5.7) |
| Maryland | 2014 | 4,612,729 | 1,063 | 350 (330-374) | 7.6 (7.2-8.1) |
| Maryland | 2015 | 4,641,577 | 1,274 | 448 (435-464) | 9.7 (9.4-10.0) |
| Maryland | 2016 | 4,660,365 | 2,036 | 830 (804-860) | 17.8 (17.2-18.5) |
| Maryland | 2017 | 4,682,945 | 2,242 | 948 (925-972) | 20.2 (19.7-20.8) |
| Maryland | 2018 | 4,700,723 | 2,313 | 951 (929-973) | 20.2 (19.8-20.7) |
| Maryland | 2019 | 4,716,722 | 2,358 | 855 (826-885) | 18.1 (17.5-18.8) |
| Maryland | 2020 | 4,721,883 | 2,757 | Data not reported to TEDS-A |  |
| Massachusetts | 2000 | 4,859,770 | 461 | 44 (41-48) | 0.9 (0.8-1.0) |
| Massachusetts | 2001 | 4,892,606 | 685 | 69 (64-75) | 1.4 (1.3-1.5) |
| Massachusetts | 2002 | 4,914,554 | 704 | 77 (71-84) | 1.6 (1.4-1.7) |
| Massachusetts | 2003 | 4,929,193 | 816 | 99 (91-108) | 2.0 (1.8-2.2) |
| Massachusetts | 2004 | 4,932,740 | 659 | 79 (73-86) | 1.6 (1.5-1.7) |
| Massachusetts | 2005 | 4,939,150 | 776 | 88 (83-95) | 1.8 (1.7-1.9) |
| Massachusetts | 2006 | 4,959,882 | 970 | 150 (143-160) | 3.0 (2.9-3.2) |
| Massachusetts | 2007 | 4,991,802 | 920 | 164 (156-174) | 3.3 (3.1-3.5) |
| Massachusetts | 2008 | 5,039,240 | 784 | 130 (123-139) | 2.6 (2.4-2.7) |
| Massachusetts | 2009 | 5,094,678 | 813 | 148 (141-157) | 2.9 (2.8-3.1) |
| Massachusetts | 2010 | 5,148,908 | 735 | 165 (157-174) | 3.2 (3.1-3.4) |
| Massachusetts | 2011 | 5,204,033 | 852 | 228 (219-239) | 4.4 (4.2-4.6) |
| Massachusetts | 2012 | 5,260,557 | 857 | 304 (295-316) | 5.8 (5.6-6.0) |
| Massachusetts | 2013 | 5,316,712 | 1,080 | 364 (352-378) | 6.8 (6.6-7.1) |
| Massachusetts | 2014 | 5,371,646 | 1,288 | 693 (679-709) | 12.9 (12.6-13.2) |
| Massachusetts | 2015 | 5,411,190 | 1,721 | 1,071 (1,052-1,092) | 19.8 (19.4-20.2) |
| Massachusetts | 2016 | 5,448,399 | 2,220 | 1,441 (1,413-1,471) | 26.5 (25.9-27.0) |
| Massachusetts | 2017 | 5,489,197 | 2,163 | 1,377 (1,349-1,407) | 25.1 (24.6-25.6) |
| Massachusetts | 2018 | 5,519,764 | 2,236 | 1,408 (1,378-1,440) | 25.5 (25.0-26.1) |
| Massachusetts | 2019 | 5,541,268 | 2,203 | 1,345 (1,313-1,378) | 24.3 (23.7-24.9) |
| Massachusetts | 2020 | 5,552,051 | 2,298 | 1,375 (1,335-1,416) | 24.8 (24.0-25.5) |
| Michigan | 2000 | 7,356,336 | 554 | 164 (154-177) | 2.2 (2.1-2.4) |
| Michigan | 2001 | 7,397,810 | 614 | 156 (145-172) | 2.1 (2.0-2.3) |
| Michigan | 2002 | 7,431,400 | 741 | 175 (163-189) | 2.4 (2.2-2.5) |
| Michigan | 2003 | 7,472,072 | 752 | 167 (156-182) | 2.2 (2.1-2.4) |
| Michigan | 2004 | 7,502,001 | 853 | 204 (189-223) | 2.7 (2.5-3.0) |
| Michigan | 2005 | 7,519,298 | 972 | 287 (269-310) | 3.8 (3.6-4.1) |
| Michigan | 2006 | 7,532,533 | 1,166 | 364 (344-387) | 4.8 (4.6-5.1) |
| Michigan | 2007 | 7,531,221 | 1,226 | 276 (257-299) | 3.7 (3.4-4.0) |
| Michigan | 2008 | 7,528,010 | 1,227 | 372 (351-397) | 4.9 (4.7-5.3) |
| Michigan | 2009 | 7,528,988 | 1,415 | 448 (422-476) | 5.9 (5.6-6.3) |
| Michigan | 2010 | 7,543,391 | 1,380 | 407 (384-439) | 5.4 (5.1-5.8) |
| Michigan | 2011 | 7,581,132 | 1,376 | 469 (445-496) | 6.2 (5.9-6.5) |
| Michigan | 2012 | 7,625,204 | 1,301 | 461 (436-490) | 6.0 (5.7-6.4) |
| Michigan | 2013 | 7,665,060 | 1,546 | 602 (570-636) | 7.9 (7.4-8.3) |
| Michigan | 2014 | 7,701,423 | 1,748 | 735 (703-772) | 9.5 (9.1-10.0) |
| Michigan | 2015 | 7,724,786 | 1,967 | 900 (865-936) | 11.6 (11.2-12.1) |
| Michigan | 2016 | 7,759,193 | 2,333 | 1,157 (1,115-1,200) | 14.9 (14.4-15.5) |
| Michigan | 2017 | 7,795,358 | 2,678 | 1,350 (1,301-1,402) | 17.3 (16.7-18.0) |
| Michigan | 2018 | 7,823,696 | 2,580 | 1,230 (1,185-1,277) | 15.7 (15.1-16.3) |
| Michigan | 2019 | 7,840,488 | 2,369 | 1,100 (1,058-1,143) | 14.0 (13.5-14.6) |
| Michigan | 2020 | 7,839,742 | 2,742 | 1,295 (1,237-1,355) | 16.5 (15.8-17.3) |
| Minnesota | 2000 | 3,643,977 | 128 | 17 (13-22) | 0.5 (0.4-0.6) |
| Minnesota | 2001 | 3,691,535 | 182 | 29 (21-38) | 0.8 (0.6-1.0) |
| Minnesota | 2002 | 3,730,140 | 198 | 26 (19-36) | 0.7 (0.5-1.0) |
| Minnesota | 2003 | 3,769,885 | 243 | 38 (31-47) | 1.0 (0.8-1.3) |
| Minnesota | 2004 | 3,805,767 | 247 | 34 (27-42) | 0.9 (0.7-1.1) |
| Minnesota | 2005 | 3,839,041 | 275 | 40 (33-49) | 1.1 (0.9-1.3) |
| Minnesota | 2006 | 3,881,174 | 306 | 44 (36-54) | 1.1 (0.9-1.4) |
| Minnesota | 2007 | 3,922,129 | 337 | 53 (45-62) | 1.4 (1.2-1.6) |
| Minnesota | 2008 | 3,962,839 | 380 | 59 (51-68) | 1.5 (1.3-1.7) |
| Minnesota | 2009 | 3,997,100 | 415 | 76 (67-86) | 1.9 (1.7-2.1) |
| Minnesota | 2010 | 4,027,612 | 393 | 72 (63-82) | 1.8 (1.6-2.0) |
| Minnesota | 2011 | 4,065,623 | 510 | 92 (82-104) | 2.3 (2.0-2.6) |
| Minnesota | 2012 | 4,099,798 | 478 | 100 (89-114) | 2.4 (2.2-2.8) |
| Minnesota | 2013 | 4,135,747 | 514 | 126 (114-140) | 3.0 (2.8-3.4) |
| Minnesota | 2014 | 4,169,635 | 513 | 136 (124-150) | 3.3 (3.0-3.6) |
| Minnesota | 2015 | 4,198,281 | 578 | 158 (144-175) | 3.8 (3.4-4.2) |
| Minnesota | 2016 | 4,232,500 | 669 | 214 (198-232) | 5.1 (4.7-5.5) |
| Minnesota | 2017 | 4,269,222 | 728 | 246 (228-266) | 5.8 (5.3-6.2) |
| Minnesota | 2018 | 4,305,672 | 634 | 222 (206-241) | 5.2 (4.8-5.6) |
| Minnesota | 2019 | 4,336,841 | 784 | 280 (259-302) | 6.5 (6.0-7.0) |
| Minnesota | 2020 | 4,356,123 | 1,035 | 413 (379-450) | 9.5 (8.7-10.3) |
| Mississippi | 2000 | 2,074,000 | 110 | 18 (12-25) | 0.9 (0.6-1.2) |
| Mississippi | 2001 | 2,084,576 | 167 | 28 (20-37) | 1.4 (1.0-1.8) |
| Mississippi | 2002 | 2,095,533 | 181 | 26 (20-33) | 1.2 (1.0-1.6) |
| Mississippi | 2003 | 2,108,865 | 205 | 40 (30-52) | 1.9 (1.4-2.5) |
| Mississippi | 2004 | 2,128,600 | 221 | 32 (23-43) | 1.5 (1.1-2.0) |
| Mississippi | 2005 | 2,145,073 | 242 | 29 (20-43) | 1.3 (0.9-2.0) |
| Mississippi | 2006 | 2,147,988 | 335 | 58 (35-80) | 2.7 (1.6-3.7) |
| Mississippi | 2007 | 2,167,179 | 311 | 54 (31-84) | 2.5 (1.4-3.9) |
| Mississippi | 2008 | 2,187,234 | 302 | Suppressed |  |
| Mississippi | 2009 | 2,200,235 | 318 | Data not reported to TEDS-A |  |
| Mississippi | 2010 | 2,217,043 | 331 | 66 (38-100) | 3.0 (1.7-4.5) |
| Mississippi | 2011 | 2,231,379 | 299 | 55 (36-77) | 2.4 (1.6-3.4) |
| Mississippi | 2012 | 2,242,590 | 310 | 79 (56-103) | 3.5 (2.5-4.6) |
| Mississippi | 2013 | 2,254,012 | 313 | 82 (51-107) | 3.6 (2.3-4.7) |
| Mississippi | 2014 | 2,260,108 | 333 | 89 (62-123) | 4.0 (2.8-5.4) |
| Mississippi | 2015 | 2,263,446 | 348 | 106 (79-133) | 4.7 (3.5-5.9) |
| Mississippi | 2016 | 2,268,992 | 350 | 97 (77-125) | 4.3 (3.4-5.5) |
| Mississippi | 2017 | 2,275,824 | 354 | 125 (104-147) | 5.5 (4.6-6.5) |
| Mississippi | 2018 | 2,275,216 | 309 | 116 (97-139) | 5.1 (4.3-6.1) |
| Mississippi | 2019 | 2,278,243 | 393 | 156 (128-188) | 6.8 (5.6-8.3) |
| Mississippi | 2020 | 2,273,653 | 582 | 256 (202-318) | 11.3 (8.9-14.0) |
| Missouri | 2000 | 4,178,902 | 299 | 88 (76-102) | 2.1 (1.8-2.5) |
| Missouri | 2001 | 4,214,567 | 346 | 86 (75-99) | 2.0 (1.8-2.4) |
| Missouri | 2002 | 4,250,312 | 412 | 125 (108-142) | 2.9 (2.5-3.3) |
| Missouri | 2003 | 4,287,476 | 515 | 125 (103-150) | 2.9 (2.4-3.5) |
| Missouri | 2004 | 4,326,785 | 538 | 144 (125-164) | 3.3 (2.9-3.8) |
| Missouri | 2005 | 4,367,322 | 600 | 163 (143-186) | 3.7 (3.3-4.3) |
| Missouri | 2006 | 4,414,380 | 731 | 228 (203-258) | 5.2 (4.6-5.9) |
| Missouri | 2007 | 4,456,266 | 704 | 184 (161-210) | 4.1 (3.6-4.7) |
| Missouri | 2008 | 4,494,971 | 758 | 228 (206-254) | 5.1 (4.6-5.7) |
| Missouri | 2009 | 4,534,485 | 852 | 291 (267-317) | 6.4 (5.9-7.0) |
| Missouri | 2010 | 4,572,091 | 987 | 351 (322-382) | 7.7 (7.0-8.4) |
| Missouri | 2011 | 4,596,992 | 953 | 372 (339-406) | 8.1 (7.4-8.8) |
| Missouri | 2012 | 4,620,893 | 932 | 356 (329-386) | 7.7 (7.1-8.4) |
| Missouri | 2013 | 4,644,830 | 1,016 | 425 (392-459) | 9.1 (8.4-9.9) |
| Missouri | 2014 | 4,665,371 | 1,052 | 479 (448-512) | 10.3 (9.6-11.0) |
| Missouri | 2015 | 4,684,914 | 1,051 | 481 (451-514) | 10.3 (9.6-11.0) |
| Missouri | 2016 | 4,704,359 | 1,362 | 691 (651-732) | 14.7 (13.8-15.6) |
| Missouri | 2017 | 4,727,436 | 1,360 | 671 (631-715) | 14.2 (13.3-15.1) |
| Missouri | 2018 | 4,746,878 | 1,599 | 815 (772-859) | 17.2 (16.3-18.1) |
| Missouri | 2019 | 4,765,772 | 1,575 | 772 (723-822) | 16.2 (15.2-17.2) |
| Missouri | 2020 | 4,780,119 | 1,859 | 921 (856-987) | 19.3 (17.9-20.6) |
| Montana | 2000 | 673,706 | 41 | 7 (4-11) | 1.1 (0.6-1.6) |
| Montana | 2001 | 679,843 | 56 | 16 (10-21) | 2.3 (1.4-3.1) |
| Montana | 2002 | 686,895 | 75 | 12 (7-19) | 1.7 (1.0-2.7) |
| Montana | 2003 | 696,618 | 92 | 27 (20-35) | 3.9 (2.9-5.0) |
| Montana | 2004 | 708,010 | 97 | 33 (21-43) | 4.7 (3.0-6.1) |
| Montana | 2005 | 718,417 | 95 | 26 (18-35) | 3.7 (2.4-4.8) |
| Montana | 2006 | 730,762 | 95 | 23 (15-30) | 3.1 (2.1-4.1) |
| Montana | 2007 | 741,571 | 123 | 32 (23-40) | 4.3 (3.0-5.4) |
| Montana | 2008 | 752,601 | 132 | 37 (24-49) | 4.9 (3.2-6.5) |
| Montana | 2009 | 760,307 | 140 | 45 (33-55) | 5.9 (4.4-7.3) |
| Montana | 2010 | 767,330 | 115 | 32 (24-39) | 4.1 (3.1-5.0) |
| Montana | 2011 | 774,388 | 138 | 36 (26-45) | 4.7 (3.4-5.8) |
| Montana | 2012 | 781,251 | 116 | 41 (31-48) | 5.3 (4.0-6.2) |
| Montana | 2013 | 789,704 | 134 | 38 (29-48) | 4.8 (3.6-6.0) |
| Montana | 2014 | 797,051 | 124 | 37 (26-49) | 4.7 (3.2-6.1) |
| Montana | 2015 | 804,802 | 138 | 44 (32-55) | 5.5 (4.0-6.9) |
| Montana | 2016 | 814,043 | 118 | 46 (32-57) | 5.6 (3.9-7.0) |
| Montana | 2017 | 824,381 | 118 | 35 (23-50) | 4.3 (2.8-6.0) |
| Montana | 2018 | 832,608 | 124 | Suppressed |  |
| Montana | 2019 | 841,235 | 143 | Suppressed |  |
| Montana | 2020 | 850,894 | 160 | Suppressed |  |
| Nebraska | 2000 | 1,263,440 | 44 | Suppressed |  |
| Nebraska | 2001 | 1,271,529 | 64 | Suppressed |  |
| Nebraska | 2002 | 1,280,578 | 65 | Suppressed |  |
| Nebraska | 2003 | 1,291,199 | 54 | Suppressed |  |
| Nebraska | 2004 | 1,301,010 | 58 | Suppressed |  |
| Nebraska | 2005 | 1,312,579 | 84 | Suppressed |  |
| Nebraska | 2006 | 1,322,595 | 88 | Suppressed |  |
| Nebraska | 2007 | 1,331,494 | 79 | Suppressed |  |
| Nebraska | 2008 | 1,342,591 | 93 | 18 (10-29) | 1.4 (0.7-2.2) |
| Nebraska | 2009 | 1,356,140 | 105 | Suppressed |  |
| Nebraska | 2010 | 1,369,846 | 113 | 34 (20-47) | 2.5 (1.4-3.4) |
| Nebraska | 2011 | 1,380,019 | 129 | 35 (26-45) | 2.5 (1.9-3.3) |
| Nebraska | 2012 | 1,391,097 | 142 | 36 (25-45) | 2.6 (1.8-3.3) |
| Nebraska | 2013 | 1,401,117 | 117 | 28 (18-36) | 2.0 (1.3-2.6) |
| Nebraska | 2014 | 1,412,432 | 122 | 37 (26-51) | 2.6 (1.8-3.6) |
| Nebraska | 2015 | 1,421,281 | 125 | 44 (34-55) | 3.1 (2.4-3.9) |
| Nebraska | 2016 | 1,432,477 | 120 | 37 (29-48) | 2.6 (2.0-3.4) |
| Nebraska | 2017 | 1,440,821 | 150 | 50 (36-66) | 3.5 (2.5-4.6) |
| Nebraska | 2018 | 1,448,931 | 137 | 55 (42-69) | 3.8 (2.9-4.7) |
| Nebraska | 2019 | 1,456,538 | 158 | 64 (48-77) | 4.4 (3.3-5.3) |
| Nebraska | 2020 | 1,462,537 | 209 | 94 (69-115) | 6.4 (4.7-7.9) |
| Nevada | 2000 | 1,502,723 | 273 | 74 (59-112) | 5.0 (4.0-7.5) |
| Nevada | 2001 | 1,563,691 | 255 | 61 (47-91) | 3.9 (3.0-5.8) |
| Nevada | 2002 | 1,620,975 | 319 | 94 (76-131) | 5.8 (4.7-8.1) |
| Nevada | 2003 | 1,679,887 | 337 | 71 (58-94) | 4.2 (3.5-5.6) |
| Nevada | 2004 | 1,754,908 | 372 | 75 (59-106) | 4.3 (3.4-6.0) |
| Nevada | 2005 | 1,820,548 | 448 | 104 (83-143) | 5.7 (4.5-7.9) |
| Nevada | 2006 | 1,888,255 | 455 | 87 (69-128) | 4.6 (3.7-6.8) |
| Nevada | 2007 | 1,947,019 | 495 | 84 (66-126) | 4.3 (3.4-6.5) |
| Nevada | 2008 | 1,991,009 | 510 | 80 (55-126) | 4.0 (2.8-6.3) |
| Nevada | 2009 | 2,018,624 | 533 | 85 (63-128) | 4.2 (3.1-6.3) |
| Nevada | 2010 | 2,039,128 | 574 | 94 (69-148) | 4.6 (3.4-7.3) |
| Nevada | 2011 | 2,055,237 | 630 | 122 (91-179) | 5.9 (4.4-8.7) |
| Nevada | 2012 | 2,089,026 | 598 | 109 (81-165) | 5.2 (3.9-7.9) |
| Nevada | 2013 | 2,120,334 | 610 | 117 (83-176) | 5.5 (3.9-8.3) |
| Nevada | 2014 | 2,158,331 | 539 | 121 (92-175) | 5.6 (4.3-8.1) |
| Nevada | 2015 | 2,200,396 | 610 | 133 (99-202) | 6.0 (4.5-9.2) |
| Nevada | 2016 | 2,243,667 | 659 | 137 (97-208) | 6.1 (4.3-9.3) |
| Nevada | 2017 | 2,289,815 | 673 | 151 (122-200) | 6.6 (5.3-8.7) |
| Nevada | 2018 | 2,341,736 | 684 | 186 (138-265) | 7.9 (5.9-11.3) |
| Nevada | 2019 | 2,396,041 | 642 | 203 (149-286) | 8.5 (6.2-12.0) |
| Nevada | 2020 | 2,440,679 | 817 | 292 (185-440) | 11.9 (7.6-18.0) |
| New Hampshire | 2000 | 929,530 | 45 | 4 (3-9) | 0.5 (0.3-0.9) |
| New Hampshire | 2001 | 943,640 | 77 | 7 (4-13) | 0.7 (0.4-1.4) |
| New Hampshire | 2002 | 956,346 | 107 | 10 (6-17) | 1.0 (0.7-1.8) |
| New Hampshire | 2003 | 968,428 | 126 | 15 (11-24) | 1.6 (1.2-2.5) |
| New Hampshire | 2004 | 980,878 | 123 | 18 (14-26) | 1.9 (1.4-2.6) |
| New Hampshire | 2005 | 991,089 | 141 | 21 (17-30) | 2.1 (1.7-3.0) |
| New Hampshire | 2006 | 1,003,220 | 147 | 21 (15-30) | 2.0 (1.5-3.0) |
| New Hampshire | 2007 | 1,011,622 | 183 | 26 (18-38) | 2.5 (1.8-3.8) |
| New Hampshire | 2008 | 1,019,877 | 125 | 23 (18-30) | 2.2 (1.8-3.0) |
| New Hampshire | 2009 | 1,025,252 | 168 | 42 (35-53) | 4.1 (3.5-5.1) |
| New Hampshire | 2010 | 1,030,880 | 160 | 39 (32-47) | 3.7 (3.1-4.6) |
| New Hampshire | 2011 | 1,039,174 | 209 | 66 (54-80) | 6.3 (5.2-7.7) |
| New Hampshire | 2012 | 1,048,284 | 173 | 66 (57-79) | 6.3 (5.5-7.6) |
| New Hampshire | 2013 | 1,055,375 | 202 | 89 (79-105) | 8.5 (7.5-9.9) |
| New Hampshire | 2014 | 1,066,317 | 334 | 185 (165-206) | 17.4 (15.5-19.4) |
| New Hampshire | 2015 | 1,072,499 | 418 | 255 (227-283) | 23.7 (21.2-26.4) |
| New Hampshire | 2016 | 1,080,995 | 479 | 286 (262-314) | 26.5 (24.2-29.0) |
| New Hampshire | 2017 | 1,089,892 | 466 | 264 (240-291) | 24.2 (22.0-26.7) |
| New Hampshire | 2018 | 1,097,019 | 449 | 260 (228-292) | 23.7 (20.7-26.7) |
| New Hampshire | 2019 | 1,104,998 | 407 | 246 (214-281) | 22.3 (19.4-25.4) |
| New Hampshire | 2020 | 1,113,141 | 391 | Suppressed |  |
| New Jersey | 2000 | 6,341,736 | 639 | 155 (145-169) | 2.4 (2.3-2.7) |
| New Jersey | 2001 | 6,389,833 | 698 | 155 (143-172) | 2.4 (2.2-2.7) |
| New Jersey | 2002 | 6,436,052 | 806 | 177 (166-193) | 2.8 (2.6-3.0) |
| New Jersey | 2003 | 6,475,388 | 720 | 160 (150-175) | 2.5 (2.3-2.7) |
| New Jersey | 2004 | 6,505,510 | 582 | 118 (109-131) | 1.8 (1.7-2.0) |
| New Jersey | 2005 | 6,530,096 | 815 | 154 (143-170) | 2.4 (2.2-2.6) |
| New Jersey | 2006 | 6,555,276 | 842 | 149 (139-165) | 2.3 (2.1-2.5) |
| New Jersey | 2007 | 6,586,862 | 716 | 124 (115-136) | 1.9 (1.7-2.1) |
| New Jersey | 2008 | 6,634,724 | 696 | 132 (124-142) | 2.0 (1.9-2.1) |
| New Jersey | 2009 | 6,686,918 | 261 | 44 (42-47) | 0.7 (0.6-0.7) |
| New Jersey | 2010 | 6,737,746 | 861 | 175 (165-187) | 2.6 (2.4-2.8) |
| New Jersey | 2011 | 6,780,856 | 1,000 | 220 (209-235) | 3.3 (3.1-3.5) |
| New Jersey | 2012 | 6,816,749 | 1,215 | 380 (364-399) | 5.6 (5.3-5.8) |
| New Jersey | 2013 | 6,845,977 | 1,288 | 443 (427-462) | 6.5 (6.2-6.7) |
| New Jersey | 2014 | 6,868,271 | 1,249 | 463 (441-489) | 6.7 (6.4-7.1) |
| New Jersey | 2015 | 6,885,593 | 1,449 | 599 (575-626) | 8.7 (8.4-9.1) |
| New Jersey | 2016 | 6,901,215 | 2,050 | 990 (960-1,024) | 14.4 (13.9-14.8) |
| New Jersey | 2017 | 6,923,660 | 2,673 | 1,319 (1,280-1,362) | 19.1 (18.5-19.7) |
| New Jersey | 2018 | 6,937,685 | 2,895 | 1,392 (1,351-1,435) | 20.1 (19.5-20.7) |
| New Jersey | 2019 | 6,947,683 | 2,799 | 1,234 (1,195-1,275) | 17.8 (17.2-18.4) |
| New Jersey | 2020 | 6,947,836 | 2,827 | 1,175 (1,130-1,223) | 16.9 (16.3-17.6) |
| New Mexico | 2000 | 1,313,072 | 263 | 73 (54-111) | 5.6 (4.1-8.4) |
| New Mexico | 2001 | 1,328,286 | 253 | Suppressed |  |
| New Mexico | 2002 | 1,352,530 | 287 | Suppressed |  |
| New Mexico | 2003 | 1,376,797 | 356 | Suppressed |  |
| New Mexico | 2004 | 1,402,624 | 312 | Suppressed |  |
| New Mexico | 2005 | 1,429,662 | 368 | 85 (62-134) | 6.0 (4.3-9.4) |
| New Mexico | 2006 | 1,457,012 | 415 | 75 (53-129) | 5.1 (3.6-8.9) |
| New Mexico | 2007 | 1,481,345 | 453 | 86 (61-139) | 5.8 (4.1-9.4) |
| New Mexico | 2008 | 1,499,448 | 515 | 129 (92-195) | 8.6 (6.1-13.0) |
| New Mexico | 2009 | 1,521,332 | 425 | 122 (85-173) | 8.0 (5.6-11.4) |
| New Mexico | 2010 | 1,546,080 | 469 | Suppressed |  |
| New Mexico | 2011 | 1,563,328 | 528 | Suppressed |  |
| New Mexico | 2012 | 1,574,164 | 486 | 222 (173-285) | 14.1 (11.0-18.1) |
| New Mexico | 2013 | 1,583,383 | 453 | 164 (122-228) | 10.3 (7.7-14.4) |
| New Mexico | 2014 | 1,586,200 | 545 | 270 (190-372) | 17.1 (12.0-23.4) |
| New Mexico | 2015 | 1,590,635 | 498 | Suppressed |  |
| New Mexico | 2016 | 1,597,519 | 492 | Suppressed |  |
| New Mexico | 2017 | 1,603,795 | 489 | Suppressed |  |
| New Mexico | 2018 | 1,611,312 | 531 | Suppressed |  |
| New Mexico | 2019 | 1,622,425 | 594 | Suppressed |  |
| New Mexico | 2020 | 1,633,828 | 778 | Data not reported to TEDS-A |  |
| New York | 2000 | 14,314,406 | 762 | 55 (50-61) | 0.4 (0.4-0.4) |
| New York | 2001 | 14,410,413 | 1,070 | 100 (93-108) | 0.7 (0.6-0.7) |
| New York | 2002 | 14,485,568 | 913 | 87 (82-94) | 0.6 (0.6-0.7) |
| New York | 2003 | 14,556,433 | 955 | 81 (75-88) | 0.6 (0.5-0.6) |
| New York | 2004 | 14,597,502 | 822 | 59 (54-66) | 0.4 (0.4-0.5) |
| New York | 2005 | 14,618,154 | 933 | 65 (60-72) | 0.4 (0.4-0.5) |
| New York | 2006 | 14,646,854 | 1,665 | 208 (197-222) | 1.4 (1.3-1.5) |
| New York | 2007 | 14,721,386 | 1,696 | 246 (235-259) | 1.7 (1.6-1.8) |
| New York | 2008 | 14,840,266 | 1,673 | 253 (242-266) | 1.7 (1.6-1.8) |
| New York | 2009 | 14,964,140 | 1,581 | 313 (301-326) | 2.1 (2.0-2.2) |
| New York | 2010 | 15,080,135 | 1,541 | 293 (282-306) | 1.9 (1.9-2.0) |
| New York | 2011 | 15,203,551 | 1,938 | 395 (382-410) | 2.6 (2.5-2.7) |
| New York | 2012 | 15,308,682 | 2,094 | 530 (515-547) | 3.5 (3.4-3.6) |
| New York | 2013 | 15,389,001 | 2,293 | 637 (620-655) | 4.1 (4.0-4.3) |
| New York | 2014 | 15,446,010 | 2,280 | 779 (760-799) | 5.0 (4.9-5.2) |
| New York | 2015 | 15,474,398 | 2,738 | 1,040 (1,018-1,064) | 6.7 (6.6-6.9) |
| New York | 2016 | 15,484,821 | 3,622 | 1,544 (1,513-1,576) | 10.0 (9.8-10.2) |
| New York | 2017 | 15,479,237 | 3,905 | 1,736 (1,702-1,772) | 11.2 (11.0-11.4) |
| New York | 2018 | 15,469,684 | 3,682 | 1,535 (1,502-1,570) | 9.9 (9.7-10.2) |
| New York | 2019 | 15,431,237 | 3,604 | 1,473 (1,438-1,510) | 9.5 (9.3-9.8) |
| New York | 2020 | 15,348,422 | 4,950 | 2,082 (2,025-2,142) | 13.6 (13.2-14.0) |
| North Carolina | 2000 | 6,113,988 | 485 | 135 (114-160) | 2.2 (1.9-2.6) |
| North Carolina | 2001 | 6,206,340 | 545 | 143 (119-170) | 2.3 (1.9-2.7) |
| North Carolina | 2002 | 6,291,750 | 634 | 128 (104-159) | 2.0 (1.6-2.5) |
| North Carolina | 2003 | 6,361,663 | 753 | 160 (132-191) | 2.5 (2.1-3.0) |
| North Carolina | 2004 | 6,467,987 | 836 | 190 (162-225) | 2.9 (2.5-3.5) |
| North Carolina | 2005 | 6,582,922 | 984 | 338 (292-382) | 5.1 (4.4-5.8) |
| North Carolina | 2006 | 6,750,877 | 1,044 | 347 (299-396) | 5.1 (4.4-5.9) |
| North Carolina | 2007 | 6,898,869 | 1,065 | 195 (164-230) | 2.8 (2.4-3.3) |
| North Carolina | 2008 | 7,057,348 | 1,177 | 211 (180-250) | 3.0 (2.5-3.5) |
| North Carolina | 2009 | 7,176,611 | 1,176 | 268 (236-299) | 3.7 (3.3-4.2) |
| North Carolina | 2010 | 7,293,118 | 1,082 | 257 (227-287) | 3.5 (3.1-3.9) |
| North Carolina | 2011 | 7,377,031 | 1,249 | 303 (276-330) | 4.1 (3.7-4.5) |
| North Carolina | 2012 | 7,471,022 | 1,285 | 350 (321-382) | 4.7 (4.3-5.1) |
| North Carolina | 2013 | 7,565,192 | 1,250 | 365 (336-394) | 4.8 (4.4-5.2) |
| North Carolina | 2014 | 7,652,739 | 1,353 | 482 (443-524) | 6.3 (5.8-6.8) |
| North Carolina | 2015 | 7,748,812 | 1,554 | 617 (577-659) | 8.0 (7.4-8.5) |
| North Carolina | 2016 | 7,865,840 | 1,946 | 893 (856-934) | 11.4 (10.9-11.9) |
| North Carolina | 2017 | 7,972,827 | 2,403 | 1,277 (1,224-1,331) | 16.0 (15.3-16.7) |
| North Carolina | 2018 | 8,086,829 | 2,252 | 1,153 (1,088-1,222) | 14.3 (13.5-15.1) |
| North Carolina | 2019 | 8,196,830 | 2,259 | 1,096 (1,028-1,166) | 13.4 (12.5-14.2) |
| North Carolina | 2020 | 8,294,423 | 3,129 | 1,529 (1,411-1,654) | 18.4 (17.0-19.9) |
| North Dakota | 2000 | 481,546 | 14 | Suppressed |  |
| North Dakota | 2001 | 482,949 | 14 | Suppressed |  |
| North Dakota | 2002 | 485,071 | 17 | Suppressed |  |
| North Dakota | 2003 | 488,411 | 22 | Suppressed |  |
| North Dakota | 2004 | 495,577 | 16 | Suppressed |  |
| North Dakota | 2005 | 497,970 | 12 | Suppressed |  |
| North Dakota | 2006 | 502,091 | 14 | Suppressed |  |
| North Dakota | 2007 | 505,559 | 31 | Suppressed |  |
| North Dakota | 2008 | 510,107 | 46 | Suppressed |  |
| North Dakota | 2009 | 516,294 | 27 | Suppressed |  |
| North Dakota | 2010 | 524,329 | 22 | Suppressed |  |
| North Dakota | 2011 | 532,742 | 16 | Suppressed |  |
| North Dakota | 2012 | 544,834 | 21 | Suppressed |  |
| North Dakota | 2013 | 559,598 | 20 | Suppressed |  |
| North Dakota | 2014 | 569,986 | 43 | Suppressed |  |
| North Dakota | 2015 | 581,151 | 61 | Suppressed |  |
| North Dakota | 2016 | 580,427 | 75 | 28 (21-35) | 4.8 (3.7-6.0) |
| North Dakota | 2017 | 580,106 | 68 | Suppressed |  |
| North Dakota | 2018 | 581,538 | 69 | Suppressed |  |
| North Dakota | 2019 | 583,140 | 82 | Suppressed |  |
| North Dakota | 2020 | 583,680 | 110 | Suppressed |  |
| Ohio | 2000 | 8,476,958 | 555 | 137 (123-157) | 1.6 (1.4-1.8) |
| Ohio | 2001 | 8,509,281 | 729 | 185 (169-212) | 2.2 (2.0-2.5) |
| Ohio | 2002 | 8,542,215 | 920 | 234 (215-256) | 2.7 (2.5-3.0) |
| Ohio | 2003 | 8,585,215 | 765 | 179 (165-198) | 2.1 (1.9-2.3) |
| Ohio | 2004 | 8,616,183 | 1,121 | 294 (269-326) | 3.4 (3.1-3.8) |
| Ohio | 2005 | 8,643,526 | 1,229 | 312 (290-337) | 3.6 (3.4-3.9) |
| Ohio | 2006 | 8,676,385 | 1,497 | 349 (325-377) | 4.0 (3.7-4.3) |
| Ohio | 2007 | 8,710,121 | 1,583 | 402 (375-432) | 4.6 (4.3-5.0) |
| Ohio | 2008 | 8,746,423 | 1,713 | 486 (457-520) | 5.6 (5.2-5.9) |
| Ohio | 2009 | 8,780,845 | 1,226 | 380 (356-408) | 4.3 (4.1-4.7) |
| Ohio | 2010 | 8,816,270 | 1,819 | 615 (586-648) | 7.0 (6.6-7.4) |
| Ohio | 2011 | 8,851,127 | 2,012 | 684 (653-720) | 7.7 (7.4-8.1) |
| Ohio | 2012 | 8,881,842 | 2,115 | 874 (838-912) | 9.8 (9.4-10.3) |
| Ohio | 2013 | 8,925,091 | 2,337 | 1,081 (1,036-1,129) | 12.1 (11.6-12.7) |
| Ohio | 2014 | 8,963,027 | 2,736 | 1,381 (1,327-1,440) | 15.4 (14.8-16.1) |
| Ohio | 2015 | 8,990,212 | 3,295 | 1,732 (1,667-1,801) | 19.3 (18.5-20.0) |
| Ohio | 2016 | 9,020,824 | 4,320 | 2,334 (2,247-2,426) | 25.9 (24.9-26.9) |
| Ohio | 2017 | 9,056,569 | 5,087 | 2,726 (2,616-2,843) | 30.1 (28.9-31.4) |
| Ohio | 2018 | 9,085,308 | 3,965 | 2,123 (2,025-2,225) | 23.4 (22.3-24.5) |
| Ohio | 2019 | 9,115,104 | 4,230 | 2,189 (2,051-2,335) | 24.0 (22.5-25.6) |
| Ohio | 2020 | 9,124,576 | 5,179 | 2,688 (2,485-2,903) | 29.5 (27.2-31.8) |
| Oklahoma | 2000 | 2,562,518 | 233 | 63 (48-80) | 2.4 (1.9-3.1) |
| Oklahoma | 2001 | 2,581,882 | 262 | 63 (48-80) | 2.4 (1.9-3.1) |
| Oklahoma | 2002 | 2,604,119 | 237 | 59 (44-73) | 2.2 (1.7-2.8) |
| Oklahoma | 2003 | 2,620,933 | 387 | 109 (85-135) | 4.1 (3.3-5.1) |
| Oklahoma | 2004 | 2,643,627 | 467 | 112 (88-143) | 4.2 (3.3-5.4) |
| Oklahoma | 2005 | 2,663,281 | 474 | 116 (87-146) | 4.4 (3.3-5.5) |
| Oklahoma | 2006 | 2,699,329 | 555 | 119 (83-153) | 4.4 (3.1-5.7) |
| Oklahoma | 2007 | 2,730,021 | 653 | 126 (85-169) | 4.6 (3.1-6.2) |
| Oklahoma | 2008 | 2,758,359 | 556 | 112 (80-146) | 4.1 (2.9-5.3) |
| Oklahoma | 2009 | 2,794,861 | 749 | 221 (159-279) | 7.9 (5.7-10.0) |
| Oklahoma | 2010 | 2,828,389 | 709 | 188 (138-233) | 6.6 (4.9-8.2) |
| Oklahoma | 2011 | 2,852,425 | 698 | 177 (132-219) | 6.2 (4.6-7.7) |
| Oklahoma | 2012 | 2,878,225 | 763 | 229 (176-271) | 8.0 (6.1-9.4) |
| Oklahoma | 2013 | 2,904,395 | 779 | 231 (185-277) | 7.9 (6.4-9.5) |
| Oklahoma | 2014 | 2,923,874 | 773 | 207 (159-255) | 7.1 (5.5-8.7) |
| Oklahoma | 2015 | 2,948,683 | 711 | 246 (202-291) | 8.4 (6.8-9.9) |
| Oklahoma | 2016 | 2,965,187 | 809 | 291 (240-337) | 9.8 (8.1-11.4) |
| Oklahoma | 2017 | 2,974,460 | 768 | 259 (216-304) | 8.7 (7.3-10.2) |
| Oklahoma | 2018 | 2,987,492 | 714 | 267 (230-307) | 8.9 (7.7-10.3) |
| Oklahoma | 2019 | 3,006,753 | 638 | 248 (216-285) | 8.3 (7.2-9.5) |
| Oklahoma | 2020 | 3,027,263 | 750 | 289 (245-336) | 9.5 (8.1-11.1) |
| Oregon | 2000 | 2,582,197 | 206 | 56 (51-62) | 2.2 (2.0-2.4) |
| Oregon | 2001 | 2,619,274 | 236 | 69 (63-77) | 2.6 (2.4-2.9) |
| Oregon | 2002 | 2,662,691 | 316 | 82 (74-96) | 3.1 (2.8-3.6) |
| Oregon | 2003 | 2,697,125 | 348 | 78 (68-93) | 2.9 (2.5-3.4) |
| Oregon | 2004 | 2,722,677 | 363 | 87 (78-99) | 3.2 (2.9-3.6) |
| Oregon | 2005 | 2,763,879 | 383 | 87 (78-100) | 3.1 (2.8-3.6) |
| Oregon | 2006 | 2,813,880 | 482 | 109 (98-123) | 3.9 (3.5-4.4) |
| Oregon | 2007 | 2,860,256 | 497 | 142 (132-155) | 5.0 (4.6-5.4) |
| Oregon | 2008 | 2,903,084 | 445 | 125 (116-138) | 4.3 (4.0-4.7) |
| Oregon | 2009 | 2,942,406 | 497 | 157 (146-169) | 5.3 (5.0-5.8) |
| Oregon | 2010 | 2,971,558 | 498 | 133 (123-145) | 4.5 (4.1-4.9) |
| Oregon | 2011 | 3,007,857 | 528 | 170 (159-184) | 5.7 (5.3-6.1) |
| Oregon | 2012 | 3,038,820 | 487 | 164 (153-176) | 5.4 (5.0-5.8) |
| Oregon | 2013 | 3,065,969 | 452 | 147 (137-160) | 4.8 (4.5-5.2) |
| Oregon | 2014 | 3,104,968 | 518 | 168 (155-185) | 5.4 (5.0-6.0) |
| Oregon | 2015 | 3,154,693 | 504 | Data not reported to TEDS-A |  |
| Oregon | 2016 | 3,222,280 | 503 | Data not reported to TEDS-A |  |
| Oregon | 2017 | 3,274,381 | 527 | Data not reported to TEDS-A |  |
| Oregon | 2018 | 3,314,659 | 544 | Data not reported to TEDS-A |  |
| Oregon | 2019 | 3,351,301 | 611 | Data not reported to TEDS-A |  |
| Oregon | 2020 | 3,380,729 | 794 | Data not reported to TEDS-A |  |
| Pennsylvania | 2000 | 9,365,323 | 1,134 | 306 (290-325) | 3.3 (3.1-3.5) |
| Pennsylvania | 2001 | 9,393,134 | 944 | 288 (273-305) | 3.1 (2.9-3.3) |
| Pennsylvania | 2002 | 9,436,096 | 1,082 | 294 (277-313) | 3.1 (2.9-3.3) |
| Pennsylvania | 2003 | 9,491,388 | 1,368 | 408 (387-432) | 4.3 (4.1-4.6) |
| Pennsylvania | 2004 | 9,537,597 | 1,530 | 413 (394-435) | 4.3 (4.1-4.6) |
| Pennsylvania | 2005 | 9,590,197 | 1,598 | 395 (373-423) | 4.1 (3.9-4.4) |
| Pennsylvania | 2006 | 9,660,031 | 1,628 | 390 (370-416) | 4.0 (3.8-4.3) |
| Pennsylvania | 2007 | 9,724,363 | 1,714 | 384 (362-413) | 4.0 (3.7-4.2) |
| Pennsylvania | 2008 | 9,791,281 | 1,829 | 490 (465-522) | 5.0 (4.8-5.3) |
| Pennsylvania | 2009 | 9,861,929 | 1,887 | 522 (493-557) | 5.3 (5.0-5.6) |
| Pennsylvania | 2010 | 9,924,925 | 1,902 | 473 (444-508) | 4.8 (4.5-5.1) |
| Pennsylvania | 2011 | 9,983,032 | 2,277 | 706 (666-753) | 7.1 (6.7-7.5) |
| Pennsylvania | 2012 | 10,028,672 | 2,354 | 838 (793-894) | 8.4 (7.9-8.9) |
| Pennsylvania | 2013 | 10,060,988 | 2,415 | 971 (923-1,029) | 9.7 (9.2-10.2) |
| Pennsylvania | 2014 | 10,088,516 | 2,716 | 1,213 (1,157-1,288) | 12.0 (11.5-12.8) |
| Pennsylvania | 2015 | 10,099,448 | 3,249 | 1,623 (1,538-1,727) | 16.1 (15.2-17.1) |
| Pennsylvania | 2016 | 10,112,196 | 4,611 | 2,573 (2,440-2,727) | 25.4 (24.1-27.0) |
| Pennsylvania | 2017 | 10,129,130 | 5,366 | 3,069 (2,906-3,240) | 30.3 (28.7-32.0) |
| Pennsylvania | 2018 | 10,156,049 | 4,398 | 2,519 (2,388-2,659) | 24.8 (23.5-26.2) |
| Pennsylvania | 2019 | 10,163,064 | 4,361 | 2,427 (2,294-2,569) | 23.9 (22.6-25.3) |
| Pennsylvania | 2020 | 10,162,497 | 5,141 | 2,877 (2,684-3,077) | 28.3 (26.4-30.3) |
| Rhode Island | 2000 | 802,203 | 73 | 6 (5-9) | 0.8 (0.6-1.1) |
| Rhode Island | 2001 | 808,846 | 107 | 8 (5-11) | 1.0 (0.6-1.4) |
| Rhode Island | 2002 | 817,305 | 99 | 10 (8-12) | 1.2 (1.0-1.5) |
| Rhode Island | 2003 | 823,267 | 145 | 15 (12-18) | 1.8 (1.5-2.2) |
| Rhode Island | 2004 | 828,351 | 105 | Suppressed |  |
| Rhode Island | 2005 | 825,984 | 156 | 16 (13-21) | 1.9 (1.6-2.5) |
| Rhode Island | 2006 | 825,748 | 172 | 16 (13-23) | 1.9 (1.5-2.7) |
| Rhode Island | 2007 | 823,660 | 135 | 8 (7-14) | 1.0 (0.8-1.6) |
| Rhode Island | 2008 | 825,205 | 183 | 18 (14-24) | 2.1 (1.7-2.9) |
| Rhode Island | 2009 | 827,744 | 159 | 15 (12-25) | 1.8 (1.4-3.0) |
| Rhode Island | 2010 | 830,501 | 166 | Suppressed |  |
| Rhode Island | 2011 | 833,282 | 190 | 22 (19-32) | 2.6 (2.2-3.9) |
| Rhode Island | 2012 | 837,360 | 195 | 40 (36-49) | 4.8 (4.3-5.9) |
| Rhode Island | 2013 | 840,699 | 241 | 80 (74-90) | 9.5 (8.8-10.7) |
| Rhode Island | 2014 | 844,036 | 247 | 109 (100-120) | 12.9 (11.8-14.2) |
| Rhode Island | 2015 | 846,199 | 309 | 127 (115-142) | 15.0 (13.6-16.8) |
| Rhode Island | 2016 | 848,635 | 325 | 144 (133-157) | 16.9 (15.6-18.6) |
| Rhode Island | 2017 | 849,612 | 319 | 138 (124-152) | 16.2 (14.6-17.9) |
| Rhode Island | 2018 | 853,279 | 317 | 147 (133-162) | 17.2 (15.6-19.0) |
| Rhode Island | 2019 | 854,235 | 306 | 133 (119-148) | 15.6 (14.0-17.4) |
| Rhode Island | 2020 | 855,276 | 395 | 182 (162-203) | 21.2 (18.9-23.7) |
| South Carolina | 2000 | 3,013,582 | 252 | 38 (27-52) | 1.2 (0.9-1.7) |
| South Carolina | 2001 | 3,048,861 | 239 | 34 (25-45) | 1.1 (0.8-1.5) |
| South Carolina | 2002 | 3,087,264 | 215 | 43 (34-52) | 1.4 (1.1-1.7) |
| South Carolina | 2003 | 3,126,512 | 281 | 48 (37-59) | 1.5 (1.2-1.9) |
| South Carolina | 2004 | 3,181,810 | 351 | 61 (45-79) | 1.9 (1.4-2.5) |
| South Carolina | 2005 | 3,233,209 | 416 | 64 (49-83) | 2.0 (1.5-2.6) |
| South Carolina | 2006 | 3,307,805 | 546 | 72 (51-95) | 2.2 (1.6-2.9) |
| South Carolina | 2007 | 3,379,920 | 536 | 66 (43-92) | 1.9 (1.3-2.7) |
| South Carolina | 2008 | 3,454,880 | 562 | 89 (66-114) | 2.6 (1.9-3.3) |
| South Carolina | 2009 | 3,510,143 | 609 | 128 (94-159) | 3.7 (2.7-4.5) |
| South Carolina | 2010 | 3,556,327 | 673 | 111 (80-146) | 3.1 (2.2-4.1) |
| South Carolina | 2011 | 3,596,472 | 612 | 119 (91-145) | 3.3 (2.5-4.0) |
| South Carolina | 2012 | 3,642,366 | 586 | 112 (81-141) | 3.1 (2.2-3.9) |
| South Carolina | 2013 | 3,688,450 | 619 | 154 (114-188) | 4.2 (3.1-5.1) |
| South Carolina | 2014 | 3,742,946 | 695 | Data not reported to TEDS-A |  |
| South Carolina | 2015 | 3,803,612 | 759 | 210 (142-297) | 5.5 (3.7-7.8) |
| South Carolina | 2016 | 3,864,117 | 870 | 278 (224-334) | 7.2 (5.8-8.6) |
| South Carolina | 2017 | 3,922,137 | 1,003 | 408 (366-451) | 10.4 (9.3-11.5) |
| South Carolina | 2018 | 3,983,114 | 1,121 | 464 (421-509) | 11.6 (10.6-12.8) |
| South Carolina | 2019 | 4,044,029 | 1,122 | 459 (414-506) | 11.3 (10.2-12.5) |
| South Carolina | 2020 | 4,100,115 | 1,730 | 487 (392-607) | 11.9 (9.6-14.8) |
| South Dakota | 2000 | 553,163 | 19 | Suppressed |  |
| South Dakota | 2001 | 557,177 | 23 | Suppressed |  |
| South Dakota | 2002 | 561,326 | 22 | Suppressed |  |
| South Dakota | 2003 | 566,403 | 24 | Suppressed |  |
| South Dakota | 2004 | 573,592 | 42 | Suppressed |  |
| South Dakota | 2005 | 579,017 | 39 | 12 (6-19) | 2.1 (1.0-3.3) |
| South Dakota | 2006 | 585,701 | 37 | 3 (2-8) | 0.6 (0.3-1.3) |
| South Dakota | 2007 | 592,776 | 31 | 6 (2-11) | 1.1 (0.4-1.8) |
| South Dakota | 2008 | 599,276 | 54 | 13 (6-21) | 2.2 (1.1-3.5) |
| South Dakota | 2009 | 605,863 | 49 | 13 (5-22) | 2.2 (0.9-3.6) |
| South Dakota | 2010 | 612,978 | 42 | 8 (4-13) | 1.4 (0.7-2.1) |
| South Dakota | 2011 | 619,681 | 56 | 14 (9-20) | 2.3 (1.4-3.2) |
| South Dakota | 2012 | 628,263 | 41 | 8 (6-12) | 1.3 (1.0-1.9) |
| South Dakota | 2013 | 634,868 | 55 | 11 (7-18) | 1.7 (1.1-2.9) |
| South Dakota | 2014 | 639,829 | 63 | 15 (11-21) | 2.3 (1.7-3.3) |
| South Dakota | 2015 | 643,267 | 64 | 17 (11-24) | 2.7 (1.7-3.7) |
| South Dakota | 2016 | 649,904 | 68 | 22 (16-30) | 3.4 (2.5-4.6) |
| South Dakota | 2017 | 657,624 | 73 | 24 (17-30) | 3.6 (2.6-4.6) |
| South Dakota | 2018 | 662,664 | 55 | 21 (15-26) | 3.1 (2.3-3.9) |
| South Dakota | 2019 | 669,310 | 83 | 31 (24-40) | 4.6 (3.6-5.9) |
| South Dakota | 2020 | 674,238 | 81 | 37 (26-49) | 5.5 (3.8-7.2) |
| Tennessee | 2000 | 4,304,034 | 388 | Suppressed |  |
| Tennessee | 2001 | 4,343,211 | 418 | Suppressed |  |
| Tennessee | 2002 | 4,381,061 | 484 | Suppressed |  |
| Tennessee | 2003 | 4,422,951 | 655 | Suppressed |  |
| Tennessee | 2004 | 4,477,466 | 756 | Suppressed |  |
| Tennessee | 2005 | 4,541,731 | 865 | Suppressed |  |
| Tennessee | 2006 | 4,618,600 | 968 | Suppressed |  |
| Tennessee | 2007 | 4,692,980 | 972 | Suppressed |  |
| Tennessee | 2008 | 4,753,057 | 923 | Suppressed |  |
| Tennessee | 2009 | 4,811,332 | 960 | 284 (207-325) | 5.9 (4.3-6.7) |
| Tennessee | 2010 | 4,860,752 | 1,071 | 315 (262-361) | 6.5 (5.4-7.4) |
| Tennessee | 2011 | 4,908,607 | 1,080 | 288 (240-334) | 5.9 (4.9-6.8) |
| Tennessee | 2012 | 4,963,555 | 1,123 | 338 (290-389) | 6.8 (5.8-7.8) |
| Tennessee | 2013 | 5,004,253 | 1,180 | 414 (363-467) | 8.3 (7.2-9.3) |
| Tennessee | 2014 | 5,048,839 | 1,263 | 493 (434-551) | 9.8 (8.6-10.9) |
| Tennessee | 2015 | 5,095,287 | 1,447 | 615 (546-683) | 12.1 (10.7-13.4) |
| Tennessee | 2016 | 5,147,093 | 1,625 | 753 (674-834) | 14.6 (13.1-16.2) |
| Tennessee | 2017 | 5,206,931 | 1,767 | 856 (782-934) | 16.4 (15.0-17.9) |
| Tennessee | 2018 | 5,267,805 | 1,812 | 925 (853-1,001) | 17.6 (16.2-19.0) |
| Tennessee | 2019 | 5,319,349 | 2,078 | 1,031 (947-1,120) | 19.4 (17.8-21.1) |
| Tennessee | 2020 | 5,373,433 | 3,021 | 1,587 (1,446-1,734) | 29.5 (26.9-32.3) |
| Texas | 2000 | 15,038,198 | 1,006 | 290 (263-326) | 1.9 (1.7-2.2) |
| Texas | 2001 | 15,339,435 | 1,354 | 417 (378-461) | 2.7 (2.5-3.0) |
| Texas | 2002 | 15,629,953 | 1,566 | 484 (446-531) | 3.1 (2.9-3.4) |
| Texas | 2003 | 15,897,951 | 1,723 | 465 (423-516) | 2.9 (2.7-3.2) |
| Texas | 2004 | 16,185,764 | 1,772 | 511 (471-561) | 3.2 (2.9-3.5) |
| Texas | 2005 | 16,487,153 | 1,878 | 495 (450-549) | 3.0 (2.7-3.3) |
| Texas | 2006 | 16,912,782 | 2,149 | 537 (487-598) | 3.2 (2.9-3.5) |
| Texas | 2007 | 17,266,111 | 2,155 | 545 (495-605) | 3.2 (2.9-3.5) |
| Texas | 2008 | 17,633,122 | 2,028 | 566 (524-613) | 3.2 (3.0-3.5) |
| Texas | 2009 | 18,008,854 | 2,374 | 719 (669-774) | 4.0 (3.7-4.3) |
| Texas | 2010 | 18,363,978 | 2,364 | 674 (618-735) | 3.7 (3.4-4.0) |
| Texas | 2011 | 18,711,018 | 2,560 | 719 (660-787) | 3.8 (3.5-4.2) |
| Texas | 2012 | 19,091,847 | 2,426 | 723 (668-787) | 3.8 (3.5-4.1) |
| Texas | 2013 | 19,424,479 | 2,424 | 723 (665-791) | 3.7 (3.4-4.1) |
| Texas | 2014 | 19,819,120 | 2,571 | 837 (773-910) | 4.2 (3.9-4.6) |
| Texas | 2015 | 20,225,833 | 2,563 | 923 (858-1,008) | 4.6 (4.2-5.0) |
| Texas | 2016 | 20,593,255 | 2,803 | 994 (921-1,082) | 4.8 (4.5-5.3) |
| Texas | 2017 | 20,925,237 | 2,966 | 1,062 (988-1,152) | 5.1 (4.7-5.5) |
| Texas | 2018 | 21,241,878 | 2,979 | 1,102 (1,021-1,197) | 5.2 (4.8-5.6) |
| Texas | 2019 | 21,580,017 | 3,111 | 1,189 (1,103-1,288) | 5.5 (5.1-6.0) |
| Texas | 2020 | 21,925,627 | 4,116 | 1,677 (1,548-1,823) | 7.6 (7.1-8.3) |
| Utah | 2000 | 1,522,816 | 210 | 66 (58-77) | 4.3 (3.8-5.1) |
| Utah | 2001 | 1,556,896 | 208 | 53 (46-66) | 3.4 (3.0-4.3) |
| Utah | 2002 | 1,591,298 | 285 | 55 (45-74) | 3.4 (2.8-4.6) |
| Utah | 2003 | 1,619,654 | 345 | 74 (61-98) | 4.5 (3.8-6.0) |
| Utah | 2004 | 1,649,809 | 354 | 65 (52-85) | 3.9 (3.2-5.2) |
| Utah | 2005 | 1,689,831 | 431 | 105 (87-132) | 6.2 (5.1-7.8) |
| Utah | 2006 | 1,735,550 | 438 | 99 (82-123) | 5.7 (4.7-7.1) |
| Utah | 2007 | 1,782,250 | 502 | 98 (85-119) | 5.5 (4.8-6.7) |
| Utah | 2008 | 1,825,771 | 466 | 109 (94-130) | 6.0 (5.2-7.1) |
| Utah | 2009 | 1,865,568 | 476 | 112 (99-132) | 6.0 (5.3-7.1) |
| Utah | 2010 | 1,901,360 | 427 | 103 (89-122) | 5.4 (4.7-6.4) |
| Utah | 2011 | 1,932,666 | 500 | 125 (109-148) | 6.5 (5.6-7.6) |
| Utah | 2012 | 1,965,293 | 595 | 138 (119-168) | 7.0 (6.1-8.6) |
| Utah | 2013 | 2,000,946 | 589 | 157 (137-184) | 7.8 (6.9-9.2) |
| Utah | 2014 | 2,034,062 | 594 | 148 (128-179) | 7.3 (6.3-8.8) |
| Utah | 2015 | 2,072,463 | 643 | 179 (157-211) | 8.7 (7.6-10.2) |
| Utah | 2016 | 2,122,960 | 628 | 198 (179-226) | 9.3 (8.4-10.6) |
| Utah | 2017 | 2,175,478 | 647 | 216 (193-248) | 9.9 (8.9-11.4) |
| Utah | 2018 | 2,224,991 | 617 | 214 (194-241) | 9.6 (8.7-10.8) |
| Utah | 2019 | 2,273,443 | 567 | 196 (178-219) | 8.6 (7.9-9.7) |
| Utah | 2020 | 2,320,603 | 619 | 217 (193-250) | 9.4 (8.3-10.8) |
| Vermont | 2000 | 462,069 | 36 | 9 (7-14) | 1.9 (1.5-3.1) |
| Vermont | 2001 | 466,183 | 41 | 10 (8-13) | 2.1 (1.7-2.8) |
| Vermont | 2002 | 471,001 | 45 | 12 (10-14) | 2.5 (2.1-3.1) |
| Vermont | 2003 | 475,140 | 65 | 15 (12-20) | 3.2 (2.6-4.1) |
| Vermont | 2004 | 478,852 | 50 | 17 (14-21) | 3.5 (2.9-4.4) |
| Vermont | 2005 | 482,282 | 53 | 12 (10-14) | 2.4 (2.0-2.9) |
| Vermont | 2006 | 486,161 | 73 | 23 (18-29) | 4.7 (3.6-6.0) |
| Vermont | 2007 | 488,786 | 64 | 17 (13-22) | 3.5 (2.7-4.4) |
| Vermont | 2008 | 491,551 | 73 | 20 (16-24) | 4.1 (3.3-4.9) |
| Vermont | 2009 | 494,367 | 55 | 12 (10-17) | 2.5 (2.0-3.5) |
| Vermont | 2010 | 497,102 | 62 | 16 (12-20) | 3.2 (2.4-4.0) |
| Vermont | 2011 | 500,193 | 83 | 23 (18-28) | 4.5 (3.6-5.5) |
| Vermont | 2012 | 501,447 | 70 | 21 (18-25) | 4.3 (3.6-5.1) |
| Vermont | 2013 | 503,499 | 93 | 36 (32-41) | 7.1 (6.3-8.1) |
| Vermont | 2014 | 504,152 | 83 | 40 (37-43) | 7.9 (7.3-8.6) |
| Vermont | 2015 | 505,693 | 99 | 44 (41-50) | 8.8 (8.1-9.9) |
| Vermont | 2016 | 506,098 | 125 | 63 (58-68) | 12.5 (11.5-13.5) |
| Vermont | 2017 | 507,986 | 134 | 70 (64-76) | 13.7 (12.6-14.9) |
| Vermont | 2018 | 509,172 | 153 | 82 (75-88) | 16.1 (14.8-17.3) |
| Vermont | 2019 | 509,721 | 132 | 71 (63-80) | 13.9 (12.3-15.7) |
| Vermont | 2020 | 510,181 | 189 | 93 (82-104) | 18.3 (16.1-20.4) |
| Virginia | 2000 | 5,364,397 | 407 | 60 (45-82) | 1.1 (0.8-1.5) |
| Virginia | 2001 | 5,443,813 | 452 | 86 (68-110) | 1.6 (1.2-2.0) |
| Virginia | 2002 | 5,515,626 | 504 | 100 (82-123) | 1.8 (1.5-2.2) |
| Virginia | 2003 | 5,584,723 | 543 | 116 (103-130) | 2.1 (1.8-2.3) |
| Virginia | 2004 | 5,673,617 | 550 | 128 (115-143) | 2.3 (2.0-2.5) |
| Virginia | 2005 | 5,760,835 | 574 | 110 (97-127) | 1.9 (1.7-2.2) |
| Virginia | 2006 | 5,847,357 | 619 | 130 (113-151) | 2.2 (1.9-2.6) |
| Virginia | 2007 | 5,916,614 | 694 | 153 (133-178) | 2.6 (2.3-3.0) |
| Virginia | 2008 | 5,995,135 | 709 | 175 (156-197) | 2.9 (2.6-3.3) |
| Virginia | 2009 | 6,080,805 | 681 | 177 (158-200) | 2.9 (2.6-3.3) |
| Virginia | 2010 | 6,169,030 | 552 | 141 (126-164) | 2.3 (2.0-2.7) |
| Virginia | 2011 | 6,244,884 | 785 | 190 (169-216) | 3.0 (2.7-3.5) |
| Virginia | 2012 | 6,324,832 | 728 | 220 (199-245) | 3.5 (3.1-3.9) |
| Virginia | 2013 | 6,391,126 | 843 | 311 (284-342) | 4.9 (4.4-5.4) |
| Virginia | 2014 | 6,449,501 | 974 | 369 (335-408) | 5.7 (5.2-6.3) |
| Virginia | 2015 | 6,498,749 | 1,037 | 449 (410-492) | 6.9 (6.3-7.6) |
| Virginia | 2016 | 6,545,777 | 1,395 | 642 (590-698) | 9.8 (9.0-10.7) |
| Virginia | 2017 | 6,598,050 | 1,498 | 712 (654-773) | 10.8 (9.9-11.7) |
| Virginia | 2018 | 6,640,878 | 1,437 | 642 (590-697) | 9.7 (8.9-10.5) |
| Virginia | 2019 | 6,687,953 | 1,537 | 674 (619-735) | 10.1 (9.2-11.0) |
| Virginia | 2020 | 6,724,143 | 2,226 | 990 (888-1,101) | 14.7 (13.2-16.4) |
| Washington | 2000 | 4,394,151 | 551 | 131 (118-150) | 3.0 (2.7-3.4) |
| Washington | 2001 | 4,468,195 | 491 | 112 (102-130) | 2.5 (2.3-2.9) |
| Washington | 2002 | 4,534,694 | 636 | 156 (141-179) | 3.5 (3.1-3.9) |
| Washington | 2003 | 4,589,238 | 691 | 145 (130-171) | 3.2 (2.8-3.7) |
| Washington | 2004 | 4,657,894 | 801 | 150 (134-174) | 3.2 (2.9-3.7) |
| Washington | 2005 | 4,733,415 | 847 | 157 (141-179) | 3.3 (3.0-3.8) |
| Washington | 2006 | 4,833,827 | 880 | 159 (140-188) | 3.3 (2.9-3.9) |
| Washington | 2007 | 4,912,005 | 951 | 166 (147-194) | 3.4 (3.0-4.0) |
| Washington | 2008 | 5,001,929 | 994 | 167 (150-191) | 3.3 (3.0-3.8) |
| Washington | 2009 | 5,093,023 | 977 | 183 (166-207) | 3.6 (3.3-4.1) |
| Washington | 2010 | 5,160,144 | 909 | 166 (150-187) | 3.2 (2.9-3.6) |
| Washington | 2011 | 5,240,936 | 991 | 228 (208-256) | 4.4 (4.0-4.9) |
| Washington | 2012 | 5,309,617 | 978 | 255 (234-279) | 4.8 (4.4-5.3) |
| Washington | 2013 | 5,372,149 | 963 | 282 (262-308) | 5.3 (4.9-5.7) |
| Washington | 2014 | 5,454,820 | 972 | 335 (313-361) | 6.1 (5.7-6.6) |
| Washington | 2015 | 5,551,936 | 1,086 | 358 (333-389) | 6.4 (6.0-7.0) |
| Washington | 2016 | 5,665,071 | 1,089 | 365 (349-384) | 6.5 (6.2-6.8) |
| Washington | 2017 | 5,776,295 | 1,161 | 400 (374-430) | 6.9 (6.5-7.5) |
| Washington | 2018 | 5,868,970 | 1,156 | Data not reported to TEDS-A |  |
| Washington | 2019 | 5,953,000 | 1,243 | Data not reported to TEDS-A |  |
| Washington | 2020 | 6,027,818 | 1,705 | Data not reported to TEDS-A |  |
| West Virginia | 2000 | 1,405,959 | 112 | Data not reported to TEDS-A |  |
| West Virginia | 2001 | 1,406,174 | 204 | Data not reported to TEDS-A |  |
| West Virginia | 2002 | 1,411,845 | 225 | Data not reported to TEDS-A |  |
| West Virginia | 2003 | 1,419,835 | 257 | Suppressed |  |
| West Virginia | 2004 | 1,424,582 | 332 | Suppressed |  |
| West Virginia | 2005 | 1,430,061 | 183 | Suppressed |  |
| West Virginia | 2006 | 1,437,275 | 359 | Suppressed |  |
| West Virginia | 2007 | 1,443,391 | 397 | Suppressed |  |
| West Virginia | 2008 | 1,450,100 | 454 | Suppressed |  |
| West Virginia | 2009 | 1,458,739 | 226 | 81 (63-96) | 5.5 (4.3-6.6) |
| West Virginia | 2010 | 1,467,020 | 507 | 182 (137-226) | 12.4 (9.3-15.4) |
| West Virginia | 2011 | 1,470,982 | 632 | 199 (133-260) | 13.5 (9.1-17.6) |
| West Virginia | 2012 | 1,472,927 | 555 | 190 (128-257) | 12.9 (8.7-17.5) |
| West Virginia | 2013 | 1,472,243 | 568 | 252 (191-314) | 17.1 (13.0-21.3) |
| West Virginia | 2014 | 1,469,636 | 624 | 269 (220-325) | 18.3 (14.9-22.1) |
| West Virginia | 2015 | 1,464,943 | 720 | 334 (258-421) | 22.8 (17.6-28.7) |
| West Virginia | 2016 | 1,457,990 | 882 | 514 (453-574) | 35.3 (31.1-39.3) |
| West Virginia | 2017 | 1,449,042 | 972 | 579 (497-656) | 40.0 (34.3-45.3) |
| West Virginia | 2018 | 1,440,834 | 851 | 481 (383-576) | 33.4 (26.6-40.0) |
| West Virginia | 2019 | 1,434,824 | 865 | 460 (341-576) | 32.1 (23.7-40.1) |
| West Virginia | 2020 | 1,428,520 | 1,328 | Suppressed |  |
| Wisconsin | 2000 | 4,003,559 | 244 | 47 (32-71) | 1.2 (0.8-1.8) |
| Wisconsin | 2001 | 4,039,242 | 262 | 51 (37-74) | 1.3 (0.9-1.8) |
| Wisconsin | 2002 | 4,079,847 | 338 | 53 (39-76) | 1.3 (1.0-1.9) |
| Wisconsin | 2003 | 4,120,698 | 379 | 71 (55-98) | 1.7 (1.3-2.4) |
| Wisconsin | 2004 | 4,159,383 | 433 | 69 (52-100) | 1.7 (1.2-2.4) |
| Wisconsin | 2005 | 4,196,300 | 511 | 91 (70-120) | 2.2 (1.7-2.9) |
| Wisconsin | 2006 | 4,228,870 | 588 | 88 (70-115) | 2.1 (1.7-2.7) |
| Wisconsin | 2007 | 4,261,874 | 611 | 89 (70-121) | 2.1 (1.7-2.8) |
| Wisconsin | 2008 | 4,295,423 | 596 | 111 (91-145) | 2.6 (2.1-3.4) |
| Wisconsin | 2009 | 4,326,853 | 621 | 137 (114-168) | 3.2 (2.6-3.9) |
| Wisconsin | 2010 | 4,353,537 | 627 | 152 (127-186) | 3.5 (2.9-4.3) |
| Wisconsin | 2011 | 4,379,398 | 690 | 186 (163-220) | 4.3 (3.7-5.0) |
| Wisconsin | 2012 | 4,403,862 | 689 | 224 (200-261) | 5.1 (4.5-5.9) |
| Wisconsin | 2013 | 4,428,871 | 850 | 300 (268-340) | 6.8 (6.0-7.7) |
| Wisconsin | 2014 | 4,450,687 | 847 | 306 (269-357) | 6.9 (6.0-8.0) |
| Wisconsin | 2015 | 4,467,318 | 875 | 337 (301-388) | 7.6 (6.7-8.7) |
| Wisconsin | 2016 | 4,486,270 | 1,063 | 519 (468-577) | 11.6 (10.4-12.9) |
| Wisconsin | 2017 | 4,509,211 | 1,170 | 576 (527-629) | 12.8 (11.7-13.9) |
| Wisconsin | 2018 | 4,533,253 | 1,072 | 505 (466-553) | 11.1 (10.3-12.2) |
| Wisconsin | 2019 | 4,556,646 | 1,193 | 571 (522-627) | 12.5 (11.5-13.8) |
| Wisconsin | 2020 | 4,574,131 | 1,523 | 771 (694-857) | 16.9 (15.2-18.7) |
| Wyoming | 2000 | 365,526 | 22 | Suppressed |  |
| Wyoming | 2001 | 368,445 | 21 | Suppressed |  |
| Wyoming | 2002 | 374,522 | 34 | Suppressed |  |
| Wyoming | 2003 | 379,271 | 29 | 4 (3-7) | 1.1 (0.7-1.7) |
| Wyoming | 2004 | 385,132 | 44 | Suppressed |  |
| Wyoming | 2005 | 390,135 | 25 | Suppressed |  |
| Wyoming | 2006 | 397,142 | 54 | Suppressed |  |
| Wyoming | 2007 | 406,116 | 62 | Suppressed |  |
| Wyoming | 2008 | 414,532 | 69 | 21 (12-29) | 5.1 (2.9-7.0) |
| Wyoming | 2009 | 424,891 | 60 | 11 (7-16) | 2.6 (1.6-3.8) |
| Wyoming | 2010 | 429,038 | 85 | 16 (10-28) | 3.8 (2.4-6.6) |
| Wyoming | 2011 | 432,071 | 83 | 16 (8-23) | 3.7 (1.9-5.3) |
| Wyoming | 2012 | 440,175 | 95 | Suppressed |  |
| Wyoming | 2013 | 444,814 | 97 | Suppressed |  |
| Wyoming | 2014 | 444,623 | 109 | Suppressed |  |
| Wyoming | 2015 | 446,645 | 95 | Suppressed |  |
| Wyoming | 2016 | 446,168 | 96 | Suppressed |  |
| Wyoming | 2017 | 443,645 | 69 | Suppressed |  |
| Wyoming | 2018 | 444,371 | 65 | Suppressed |  |
| Wyoming | 2019 | 446,539 | 79 | Suppressed |  |
| Wyoming | 2020 | 449,237 | 99 | Suppressed |  |
