# Supplementary figures and images for: Estimated Number of Injection-Involved Overdose Deaths in US States From 2000 to 2020: Secondary Analysis of Surveillance Data
Source: JMIR Public Health Surveill. 2024 Apr 5;10:e49527. doi: 10.2196/49527 (PMC11031697; doi:10.2196/49527)

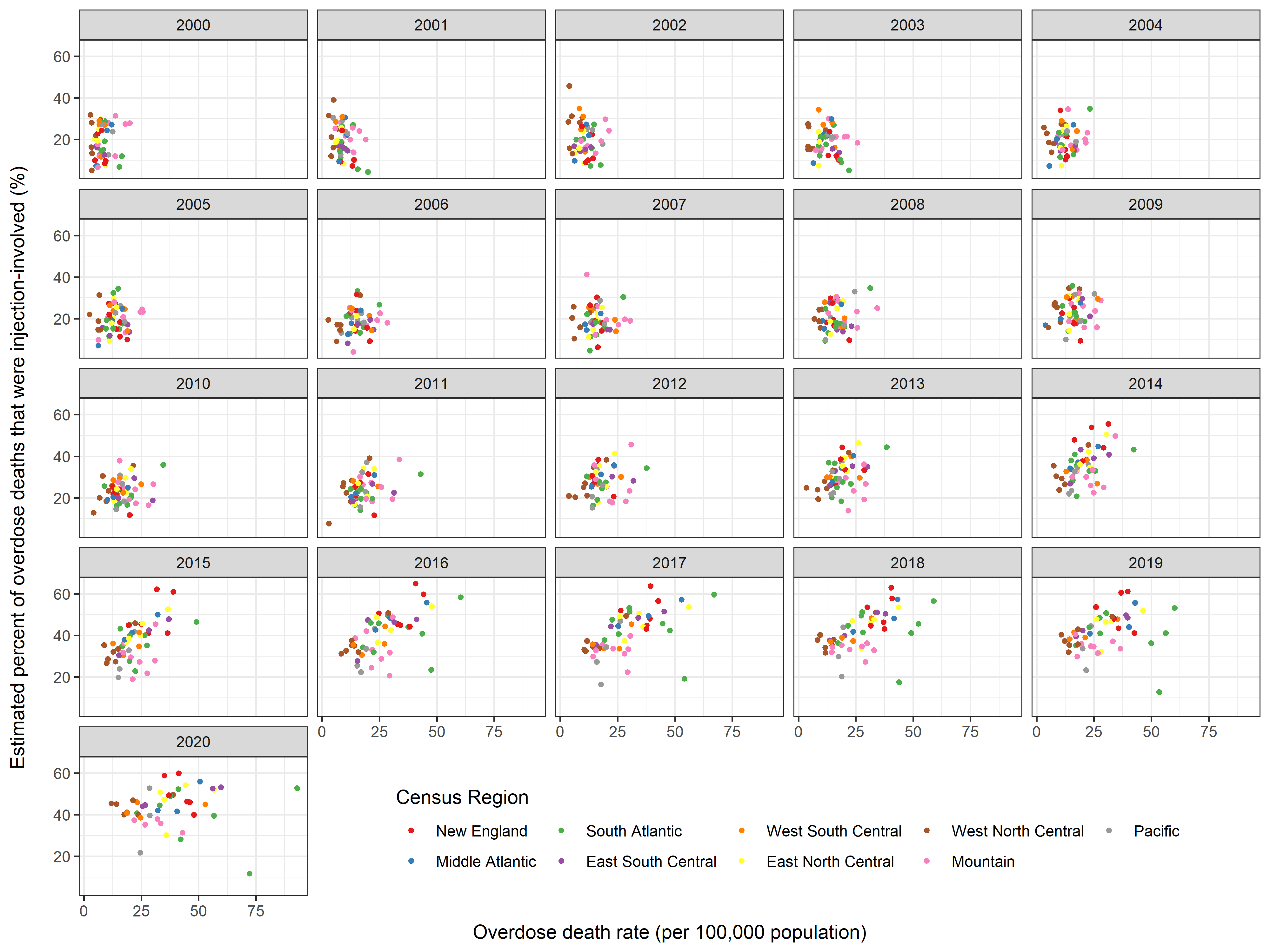

Supplement: Multimedia Appendix 4 [file publichealth_v10i1e49527_app4.png]
